# Supplementary figures and images for: A negative feedback loop is critical for recovery of RpoS after stress in Escherichia coli (part 1 of 2)
Source: PLoS Genet. 2024 Mar 11;20(3):e1011059. doi: 10.1371/journal.pgen.1011059 (PMC10957080; doi:10.1371/journal.pgen.1011059)

Supplemental Fig S1

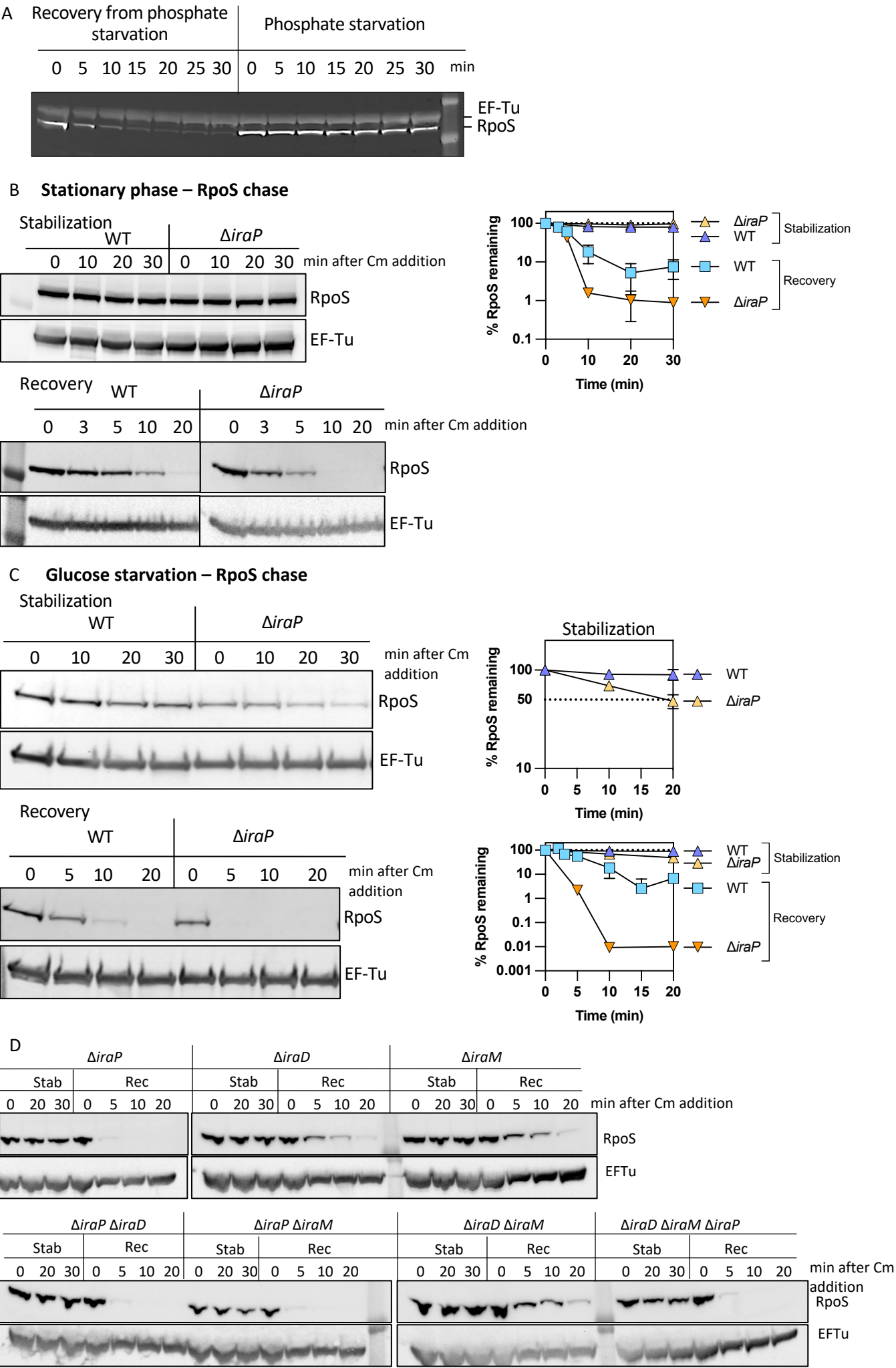

Supplement: S1 Fig — A) Western blots showing RpoS accumulation (no chloramphenicol) after phosphate was added to the MG1655 strain (recovery) or when incubation was continued without phosphate addition. Results are plotted in Fig 1D. B) Western blot against RpoS and the loading control EF-Tu showing RpoS degradation (chase) during (stabilization) and after exit from stationary phase (recovery) in MG1655 and ΔiraP (SB151) strains. Cells were grown in MOPS minimal glucose medium overnight to reach stationary phase. Stationary-phase cells were diluted 5-fold back into fresh medium and chloramphenicol was added after 2 minutes. Samples were taken and treated as described for Fig 1. The graph on the right corresponds to quantification of RpoS degradation (n > 3). C) Western blot against RpoS and the loading control EF-Tu showing RpoS degradation (chase) during and after glucose starvation in MG1655 and ΔiraP (SB151) strains. Samples were taken and treated as described for Fig 1. The graphs on the right and at the bottom correspond to quantification of RpoS degradation (n > 3). D. Stationary Phase, RpoS chase in ira mutants. Western blot of RpoS and the loading control EF-Tu showing RpoS degradation after Chloramphenicol addition (chase) during (Stab, for stabilization) and after exit from stationary phase (Rec for recovery) as in B, but in the following isogenic derivatives of MG1655, carrying different combinations of ira mutant alleles. Strains used: ΔiraP (SB151); ΔiraD (SB364); ΔiraM (SB539); ΔiraP ΔiraD (SB365); ΔiraP ΔiraM (SB540); ΔiraD ΔiraP (SB541); ΔiraD ΔiraM ΔiraP (SB542). Quantitation of triplicates is shown in Fig 2D (stabilization) and 2E (recovery). (PDF) [file pgen.1011059.s001.pdf]

Supplemental Fig S2

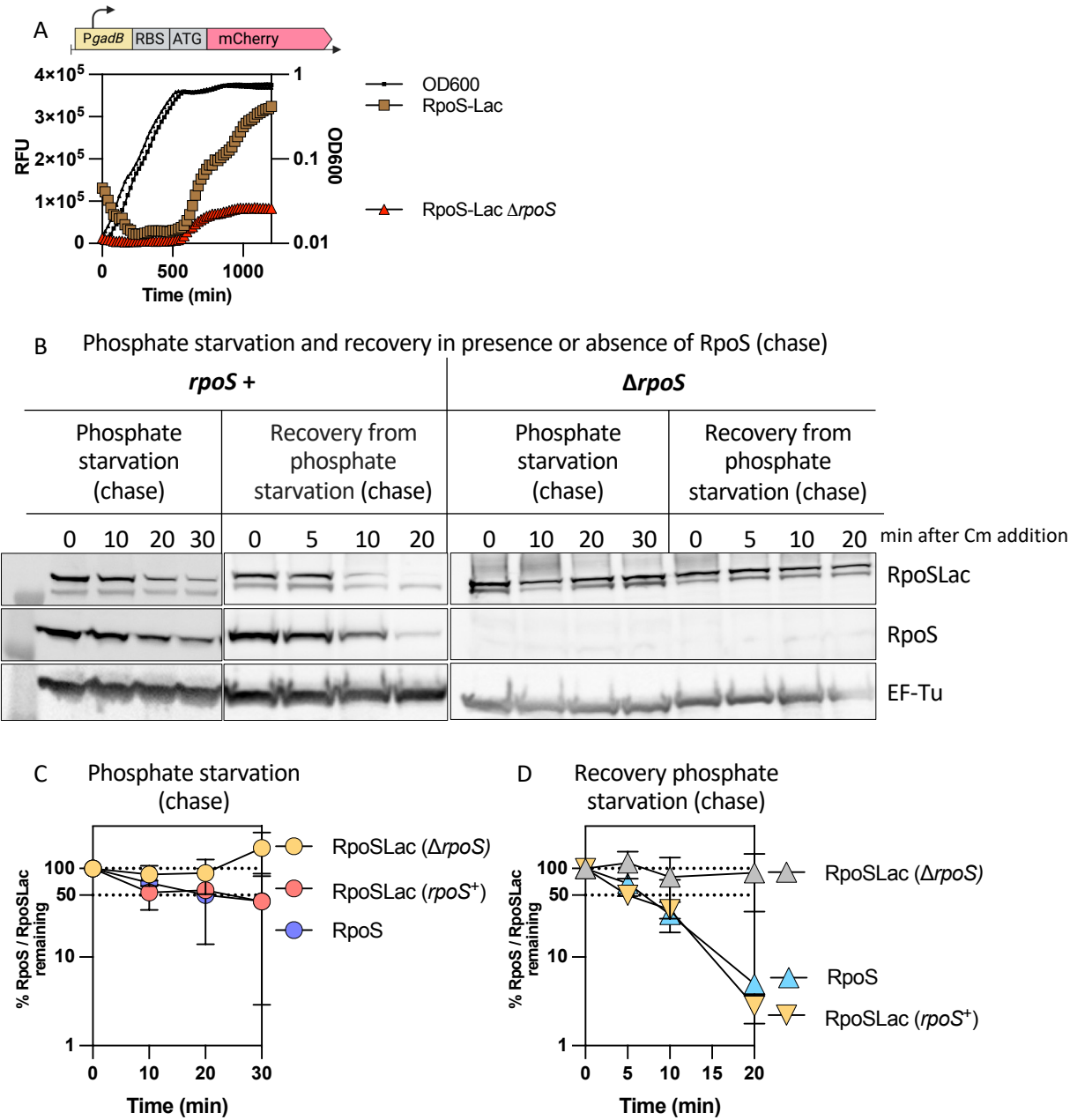

Supplement: S2 Fig — A) The RpoS-Lac fusion protein is inactive for transcription. The plasmid pSB23 bearing a transcriptional fusion between the RpoS-dependent promoter PgadB and mCherry (full description of the fusions and method of measurement described in S4 Fig) was introduced in strains containing RpoS-Lac in the presence (SG30013) and absence of RpoS (INH28). The loss of the mCherry signal upon deletion of rpoS confirms that the fusion protein is not able to activate an RpoS-dependent promoter. B) Western blot showing RpoS and RpoS-Lac stabilization and degradation during phosphate starvation and recovery in the strains containing the RpoS-Lac translational fusion in the presence of RpoS (strain SG30013), and in the absence of RpoS (INH28). The protocol is as in Fig 1, with chloramphenicol added to stop translation. C) Quantitation of RpoS and RpoS-Lac half-life during phosphate starvation from Western Blot as shown in S2B Fig (n = 3). (D) Quantitation of RpoS and RpoS-Lac half-life during recovery from phosphate starvation, from Western Blot as shown in S2B Fig (n = 3). (PDF) [file pgen.1011059.s002.pdf]

Supplemental Fig S3

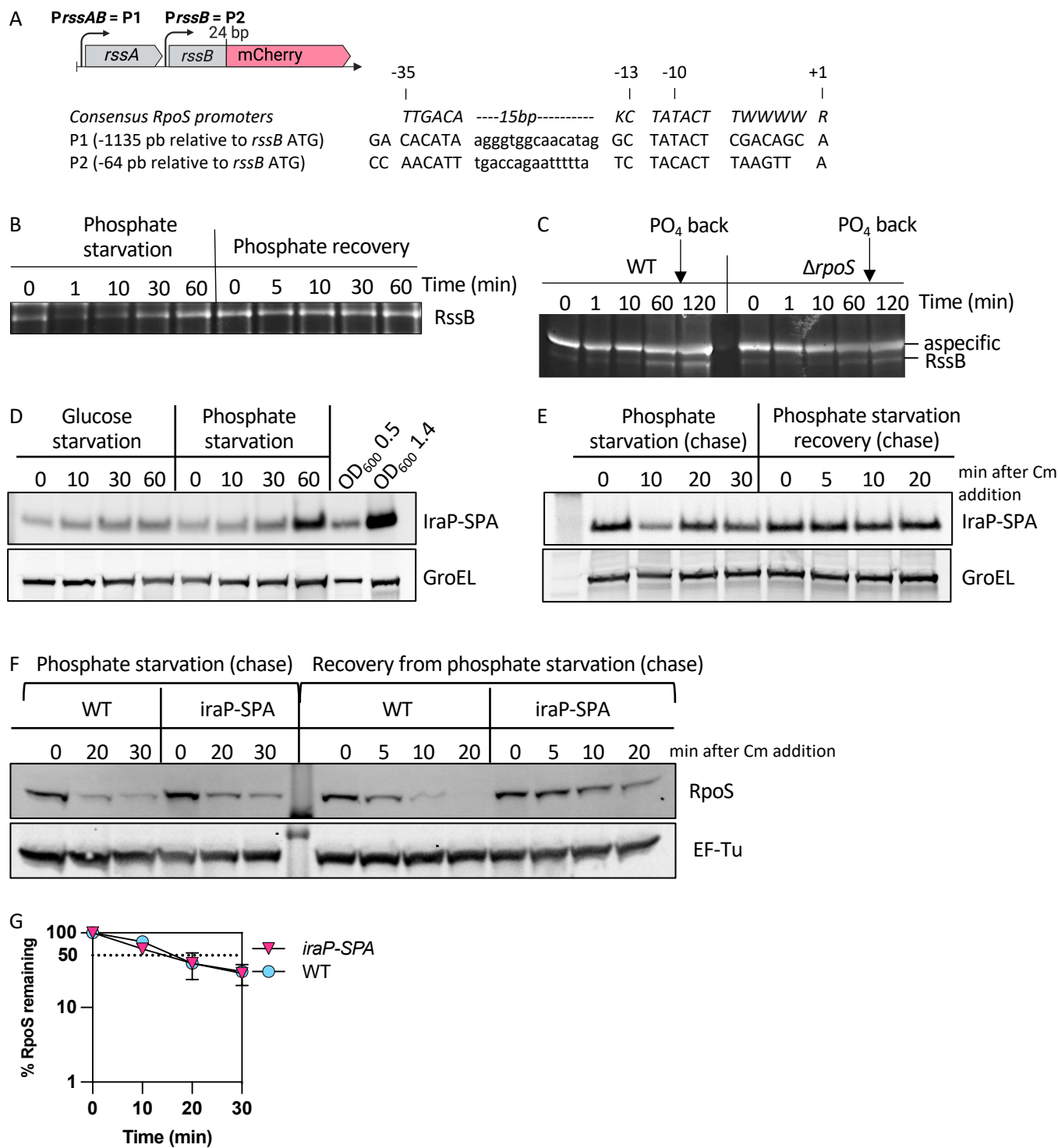

Supplement: S3 Fig — A) P1 and P2 rssB promoter sequences and fusion schematics. 1597pb upstream of the rssB coding gene and the first 24 bp of the rssB gene were fused to mCherry. The start point for the P1 promoter is located 1135pb upstream of the first rssB codon and 139 nt upstream of the first rssA codon; P2 is located 64 bp upstream of the first rssB gene codon. Note that the position of the P2 start was determined from dRNA-seq data determined by Thomason et al [46] and differs from the start site previously identified for P2. B) Western Blot of RssB levels during phosphate starvation and after phosphate was added back (phosphate recovery) in MG1655 (WT). C) Western Blot of RssB levels during phosphate starvation (1–60’ samples) and one hour after phosphate was added back (120’ sample) in WT and ΔrpoS (AB165) strains. D) IraP-SPA levels during glucose starvation, phosphate starvation (0, 10, 30 and 60-minute samples for each), exponential phase (OD600 = 0.5) and stationary phase (OD600 = 1.4). Western blot against FLAG-tag of samples from a strain in which a SPA tag was inserted at the C-terminus of IraP in the chromosome (strain SB212) and against the loading control GroEL. Phosphate and glucose starvation follow the protocol as described in Figs 1 and 2. Note that we were unable to detect untagged IraP by western blot. E) Stability of IraP-SPA protein during and after phosphate starvation. Chase experiment in the strain SB212 containing iraP-SPA at the iraP locus and Western Blot against Flag-tag detecting the SPA tag of IraP. Samples were taken from cells undergoing phosphate starvation; chloramphenicol was added to start the chase as described in Figs 1 and 2. F) Effect of IraP-SPA tag on RpoS stabilization and recovery. RpoS chase during and after phosphate starvation in strains WT (MG1655) and SB212 containing iraP-SPA at the iraP locus, following the protocol as described in Fig 1A. G) RpoS chase during phosphate starvation in strains WT (MG1655) and SB212 containing iraP [file pgen.1011059.s003.pdf]

Supplemental Fig S4

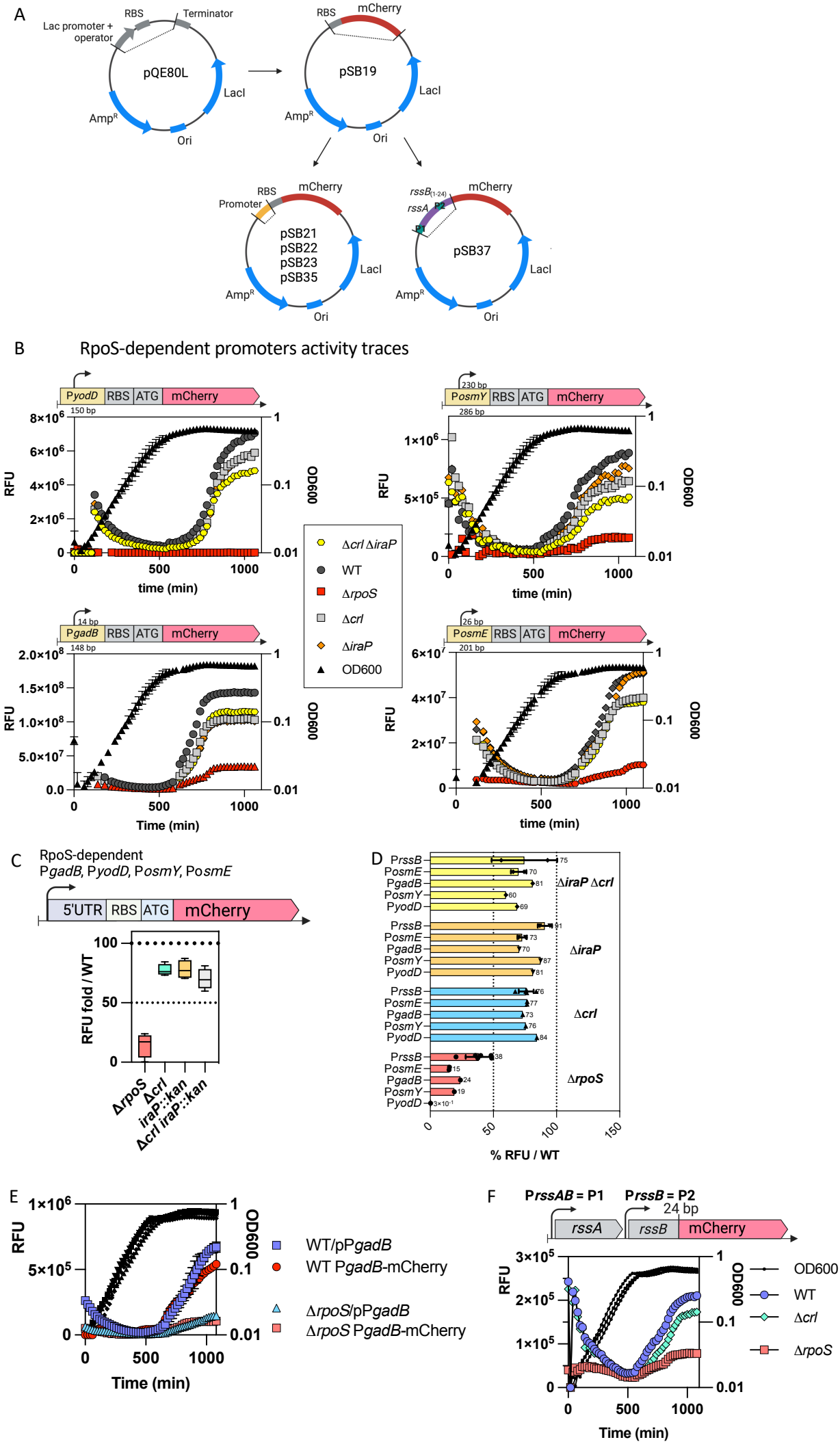

Supplement: S4 Fig — A) Plasmid maps for construction of transcriptional fusions. The empty vector pQE80L was used as the backbone to construct the pSB19 plasmid by Gibson assembly, replacing the Lac promoter/operator and the RBS by the coding sequence of mCherry following a consensus RBS sequence (see dotted lines for both pQE80L and pSB19). The promoter regions of yodD, osmY, gadB and osmE or the 1658 bp upstream of rssB were then introduced into pSB19, obtaining the plasmids pSB21, pSB22, pSB23, pSB38 and pSB37 respectively. B) The activity of the transcriptional mCherry fusions of the RpoS-dependent promoters of yodD (pSB21, top left panel), osmY (pSB22, top right panel), gadB (pSB23, bottom left panel) and osmE (pSB38, bottom right panel) in the WT, ΔrpoS (AB165), Δcrl (INH24), iraP::kan (AB006) and iraP::kan Δcrl (INH26) strains. Strains were grown from exponential to stationary phase in a microplate reader in MOPS minimal medium that measured mCherry fluorescence and OD600 every 20 minutes. The transcriptional fusion construct is shown above each graph (see Material and methods for details). C) Comparison of relative average expression of the transcriptional fusions of the four RpoS-dependent promoters to WT, after 16 hours of growth, from data as in S4B (n > 3). D) Relative expression of the four RpoS-dependent transcriptional fusions in the ΔrpoS (AB165), Δcrl (SB147), ΔiraP (SB151) and ΔiraP Δcrl (INH26) strains, with WT (MG1655) set to 100, during stationary phase. E) Comparison of the activity of the gadB promoter fused to mCherry, expressed either from the pQE80L-derived plasmid pSB23 or from the same fusion expressed as a single chromosomal copy, in WT (MG1655; blue symbols) and ΔrpoS (AB165; red symbols) strains. Strains were grown and the RFU measured as described in Fig 4A. F) Fluorescence over time of the translational fusion contained on pSB37, carrying the upstream region of rssB fused to mCherry, as shown in S4 Fig. The fusion extends 1597bp upstream of the rssB cod [file pgen.1011059.s004.pdf]

Supplemental Fig S6

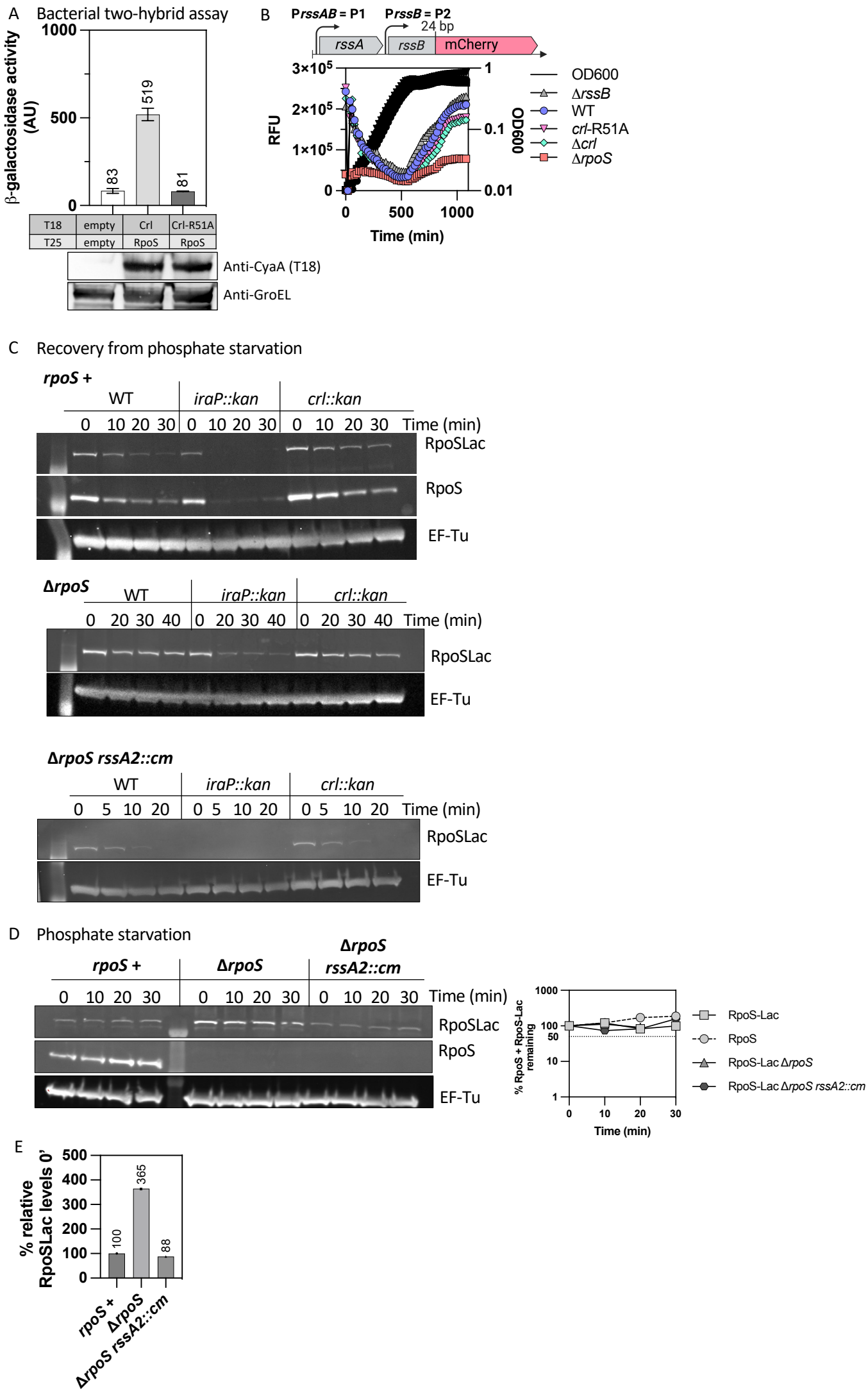

Supplement: S6 Fig — A) Bacterial two-hybrid experiment showing the interaction between RpoS and Crl. A plasmid expressing the T25 domain of adenylate cyclase was fused to the rpoS coding gene at its 5’ end and a plasmid expressing the T18 domain was fused to the wild-type crl or crl-R51A coding gene at its 5’ end. Western Blot detecting the T18 domain of the adenylate cyclase (below graph) shows similar production of T18-Crl and T18-Crl-R51A in the cya+ MG1655 strain. B) Fluorescence over time of the translational rssB fusion between mCherry contained on the pSB37 plasmid in WT, ΔrpoS (AB165), ΔrssB (SB94), Δcrl (SB147) and crl-R51A (SB148) strains, grown from exponential to stationary phase in a microplate reader that measured mCherry fluorescence and OD600 every 20 minutes. C) Western blot against RpoS showing RpoS and RpoS-Lac recovery accumulation from phosphate starvation in WT, iraP::kan and crl::kan strains. rpoS+ strains: SG300013 (crl+ iraP+), SB179 (iraP-) and SB180 (crl-). ΔrpoS strains: INH28 (crl+), SB175 (iraP-) and SB176 (crl-). ΔrpoS rssA2::cm strains: SB150 (crl+), SB173 (iraP-) and SB174 (crl-). D) Western blot showing RpoS and RpoS-Lac accumulation during phosphate starvation in the crl+ iraP+ strains used in C. Protocol is as in Fig 1. E) Relative RpoS-Lac levels from the Western Blot in S6D Fig at 0’ time points (pre-phosphate starvation). Values are represented as percentage relative to RpoS-Lac levels in the rpoS+ strain, set to 100. (PDF) [file pgen.1011059.s006.pdf]

Supplemental Fig S7

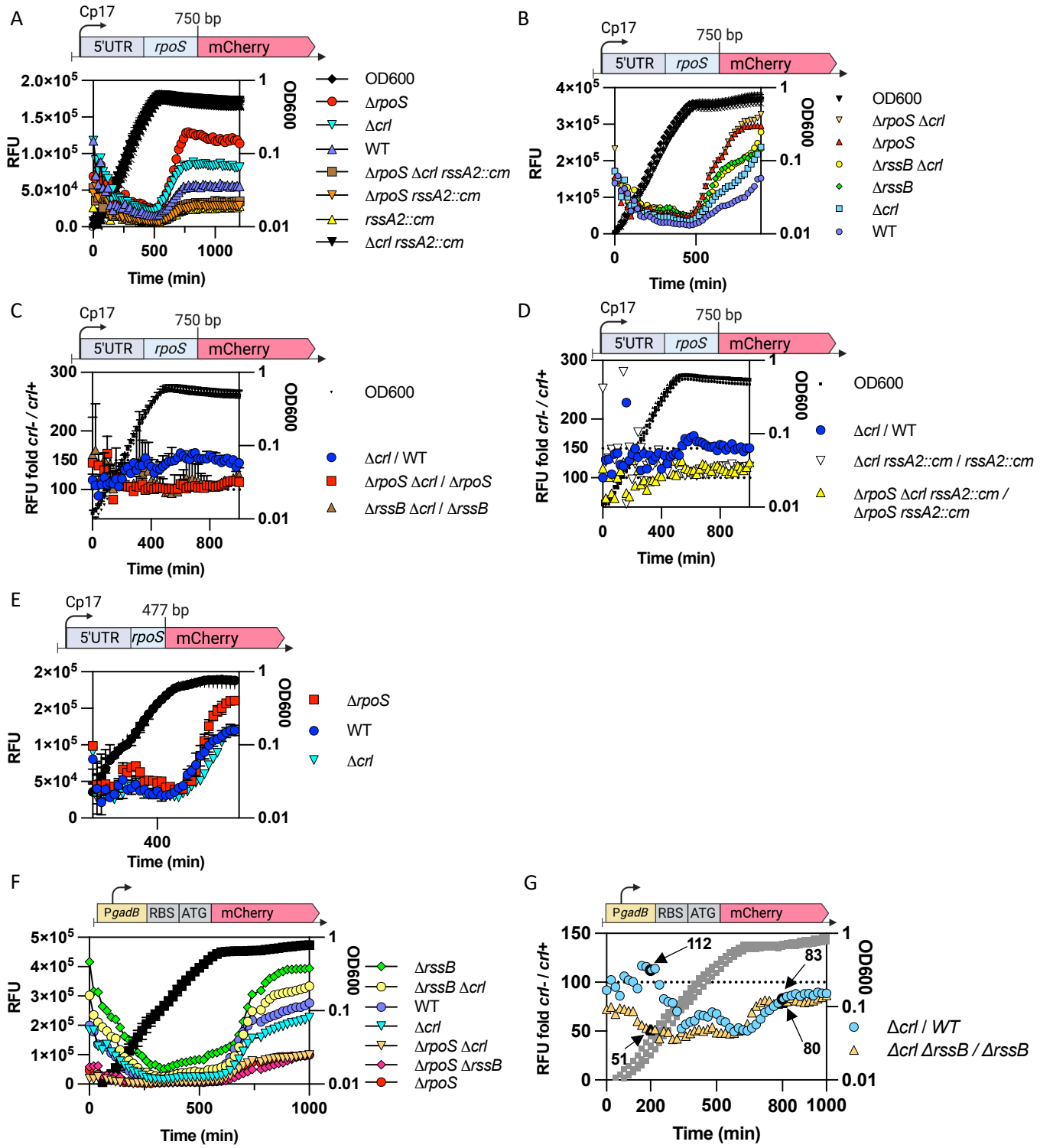

Supplement: S7 Fig — A) The fluorescence of the degradable RpoS-mCherry fusion in WT (NM801), ΔrpoS (SB238), rssA2::cm (SB226), Δcrl (SB228), ΔrpoS rssA2::cm (SB281), ΔrpoS Δcrl rssA2::cm (SB280) and Δcrl rssA2::cm (SB282) strains was measured as described in Fig 4A. B) The fluorescence of the degradable RpoS-mCherry fusion in WT (NM801), ΔrpoS (SB238), ΔrpoS Δcrl (SB239), Δcrl (SB228), ΔrssB Δcrl (SB283), and ΔrssB (SB225) strains was measured as described in Fig 4A. C) Ratios between RFU values in strains crl-/ crl+ in WT, rpoS- and rssB- strains, from the data shown in S7B Fig. WT is set to 100. D) Full graphs of the ratios between the strains deleted of crl (Δcrl (SB228), Δcrl ΔrpoS rssA2::cm (SB280) and Δcrl rssA2::cm (SB282)) and the corresponding crl+ strains (WT (NM801), ΔrpoS rssA2::cm (SB281) and rssA2::cm (SB226)). Fluorescence and data analysis were performed as described in Fig 4A, with data during growth from S7A Fig. WT is set to 100. E) The fluorescence of the non-degradable RpoS-mCherry fusion in WT (NM802), ΔrpoS (SB439) and Δcrl (SB437) strains was measured as described in Fig 4A. F) The fluorescence of the chromosomally-inserted PgadB-mCherry transcriptional fusion from WT (SB66), ΔrpoS (SB67), ΔrssB (SB242), Δcrl (SB243), ΔrpoS ΔrssB (SB298), ΔrssB Δcrl (SB297) and Δcrl ΔrpoS (SB296) strains was measured as described in Fig 4A. G) The effect of the absence of Crl on PgadB-mCherry. mCherry fluorescence of the strains WT (SB66), ΔrssB (SB242), Δcrl (SB243) and ΔrssB Δcrl (SB297), all containing the chromosomally-inserted PgadB-mCherry fusion was measured over time as described in Fig 4A. Using the data from rpoS+ strains in S7F Fig, the RFU ratios during growth between the strains containing deletion of crl compared to crl+, in WT or ΔrssB strains was calculated, with the value in the crl+ strain across growth set to 100. The arrows indicate the ratios at times 200 and 800 minutes. (PDF) [file pgen.1011059.s007.pdf]

Supplemental Fig S8

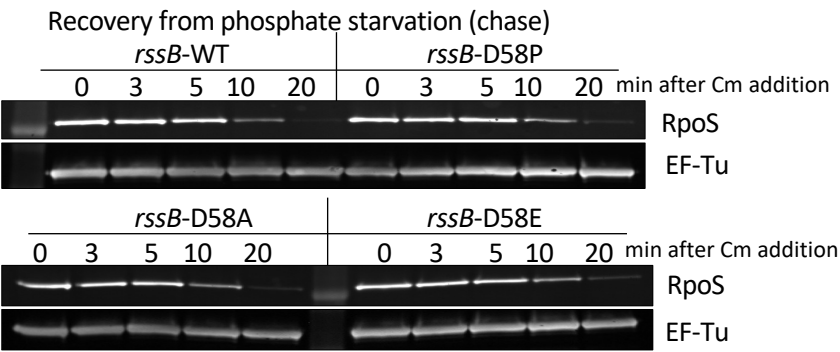

Supplement: S8 Fig — Western Blots against RpoS and the loading control EF-Tu showing RpoS degradation after phosphate starvation of the strains WT, rssB-D58E (SB192), rssB-D58A (SB190) and rssB-D58P (SB198). Primary data for Fig 7B. (PDF) [file pgen.1011059.s008.pdf]

Supplemental Fig S9

A Chase – phosphate starvation

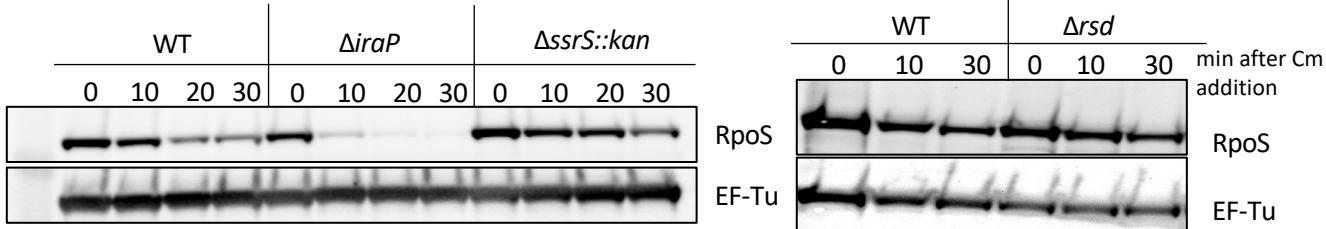

B Chase – recovery from phosphate starvation

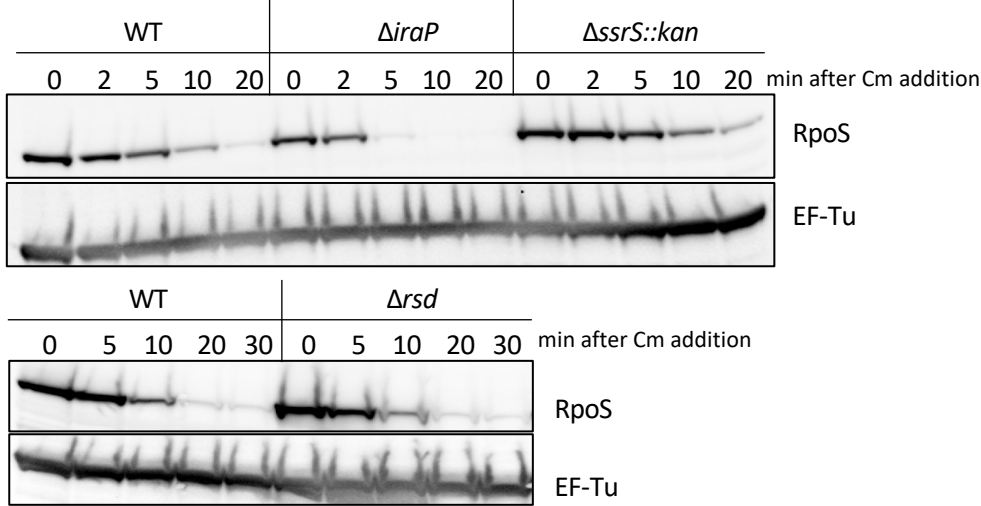

Supplement: S9 Fig — Primary data for Fig 8. A) Western blot against RpoS and the loading control EF-Tu showing RpoS stabilization during phosphate starvation in MG1655, ΔiraP (SB151), ΔssrS::kan (SB470) and Δrsd (SB505) strains. Samples were taken and treated as described in Fig 1A, chloramphenicol was added to stop translation. B) Western blot against RpoS and the loading control EF-Tu showing RpoS degradation during recovery from phosphate starvation in MG1655, ΔiraP (SB151), ΔssrS::kan (SB470) and Δrsd (SB505) strains. Samples were taken and treated as described in Fig 1A, chloramphenicol was added to stop translation. (PDF) [file pgen.1011059.s009.pdf]

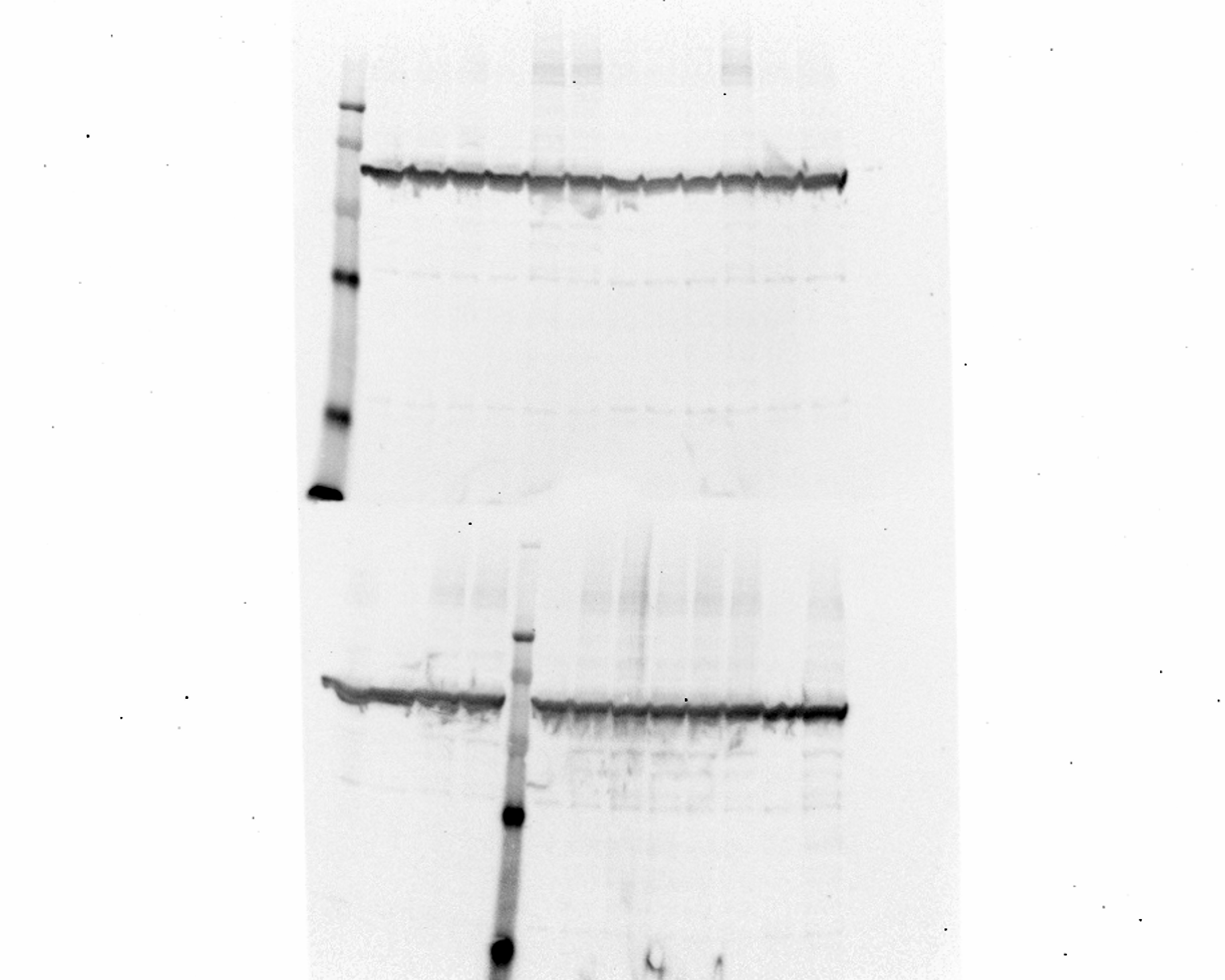

Supplement: S1 Data — (ZIP) [file pgen.1011059.s014.zip › SIdata/Figure 3 + S2/Fig3+S2B+S2C+S2D_WB RpoS+RpoSLac chase phosphate starvation+recovery/lmbchemidoc 2023-10-06 16h21m06s(DyLight 800).tif]

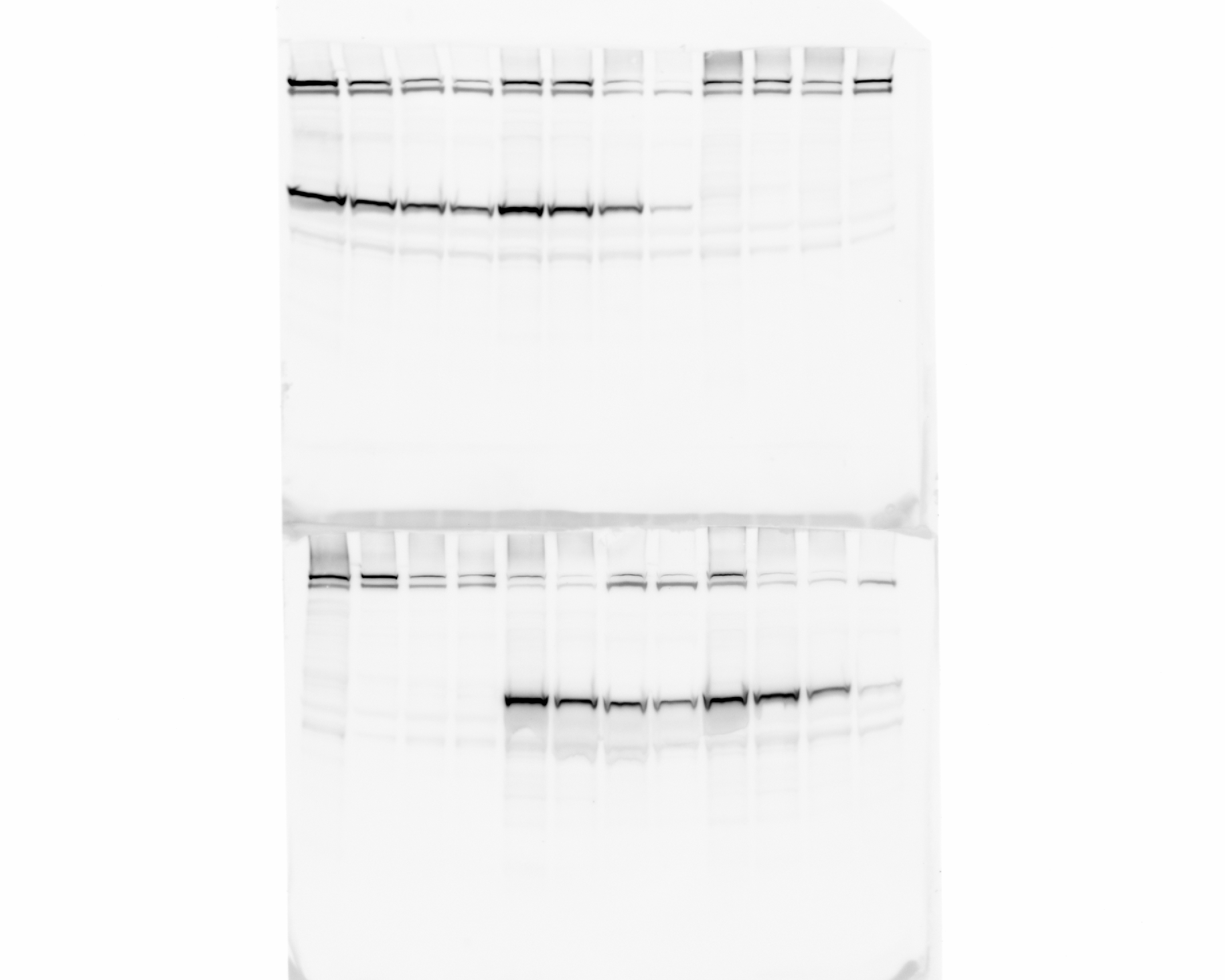

Supplement: S1 Data — (ZIP) [file pgen.1011059.s014.zip › SIdata/Figure 3 + S2/Fig3+S2B+S2C+S2D_WB RpoS+RpoSLac chase phosphate starvation+recovery/lmbchemidoc 2023-10-04 15h19m30s(StarBright B700).tif]

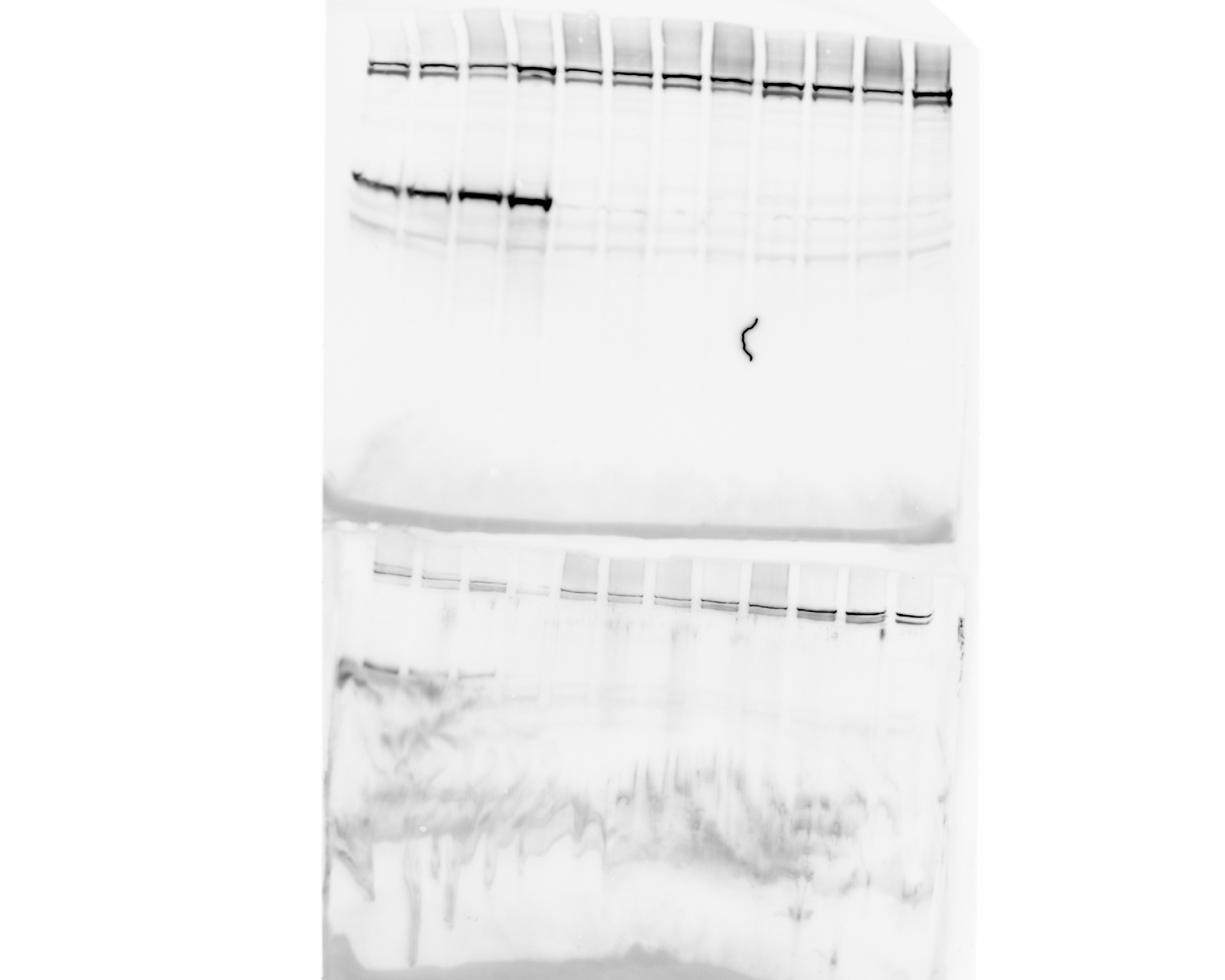

Supplement: S1 Data — (ZIP) [file pgen.1011059.s014.zip › SIdata/Figure 3 + S2/Fig3+S2B+S2C+S2D_WB RpoS+RpoSLac chase phosphate starvation+recovery/lmbchemidoc 2023-10-04 15h20m57s(StarBright B700).tif]

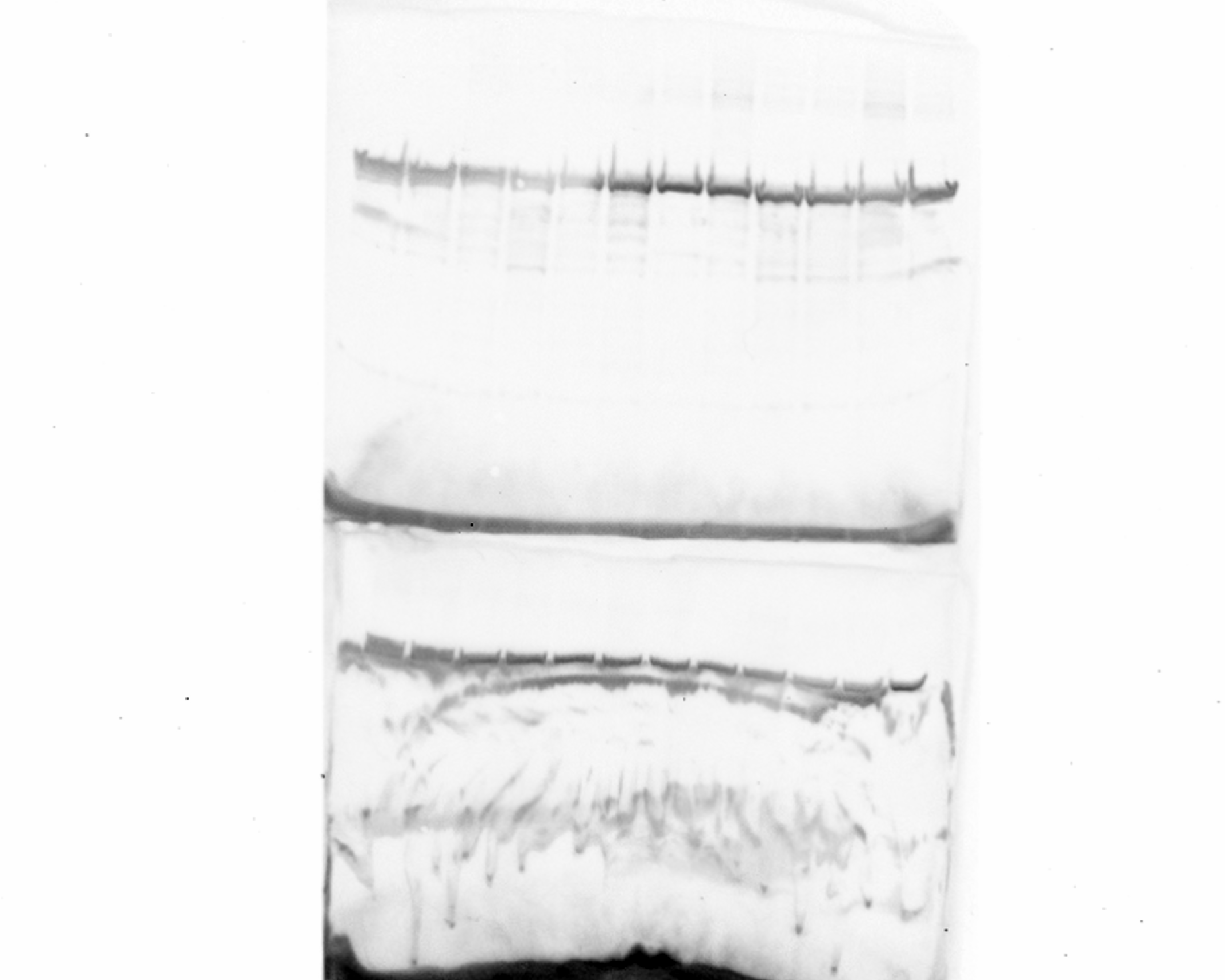

Supplement: S1 Data — (ZIP) [file pgen.1011059.s014.zip › SIdata/Figure 3 + S2/Fig3+S2B+S2C+S2D_WB RpoS+RpoSLac chase phosphate starvation+recovery/lmbchemidoc 2023-10-04 15h20m57s(DyLight 800).tif]

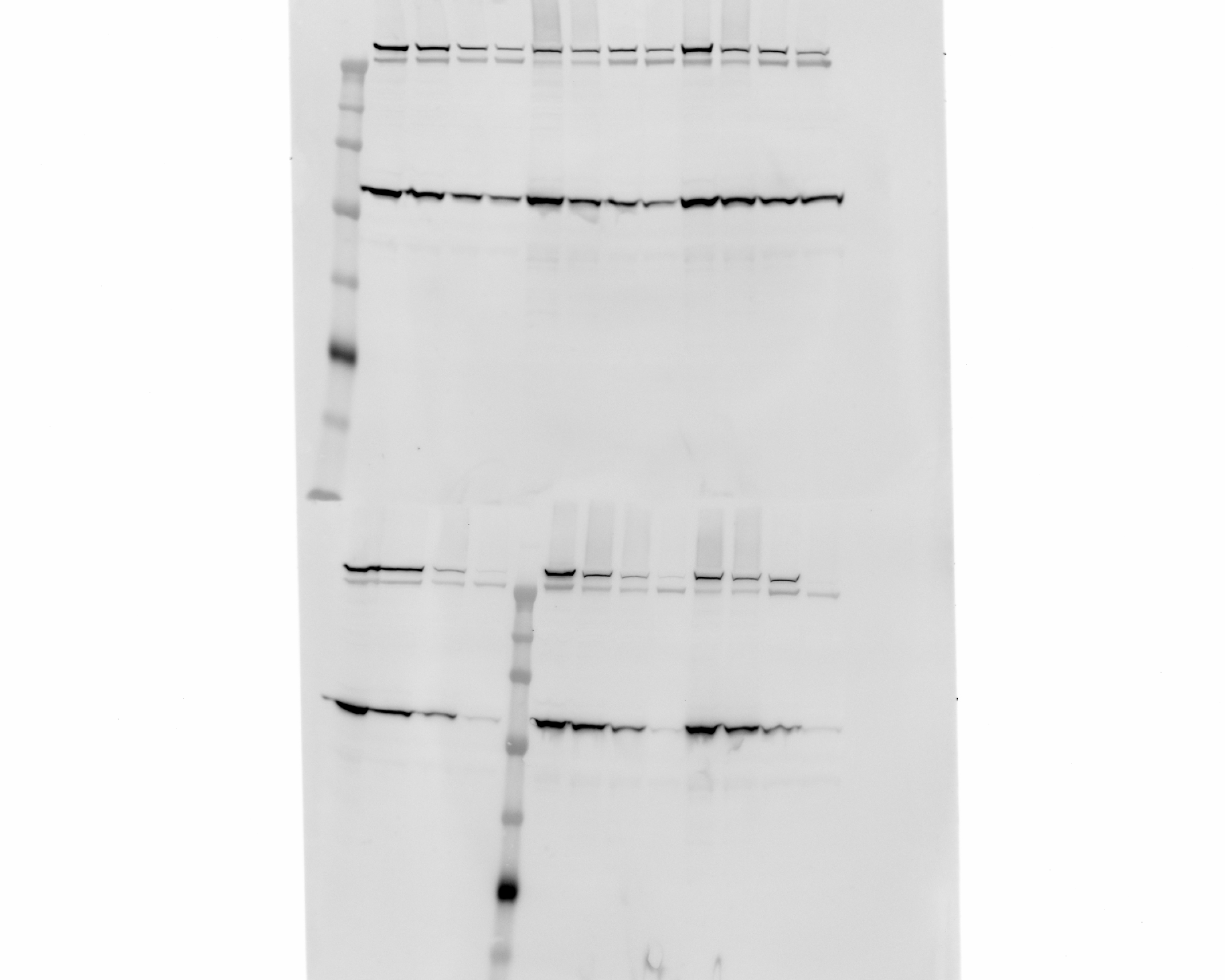

Supplement: S1 Data — (ZIP) [file pgen.1011059.s014.zip › SIdata/Figure 3 + S2/Fig3+S2B+S2C+S2D_WB RpoS+RpoSLac chase phosphate starvation+recovery/lmbchemidoc 2023-10-06 16h21m06s(StarBright B700).tif]

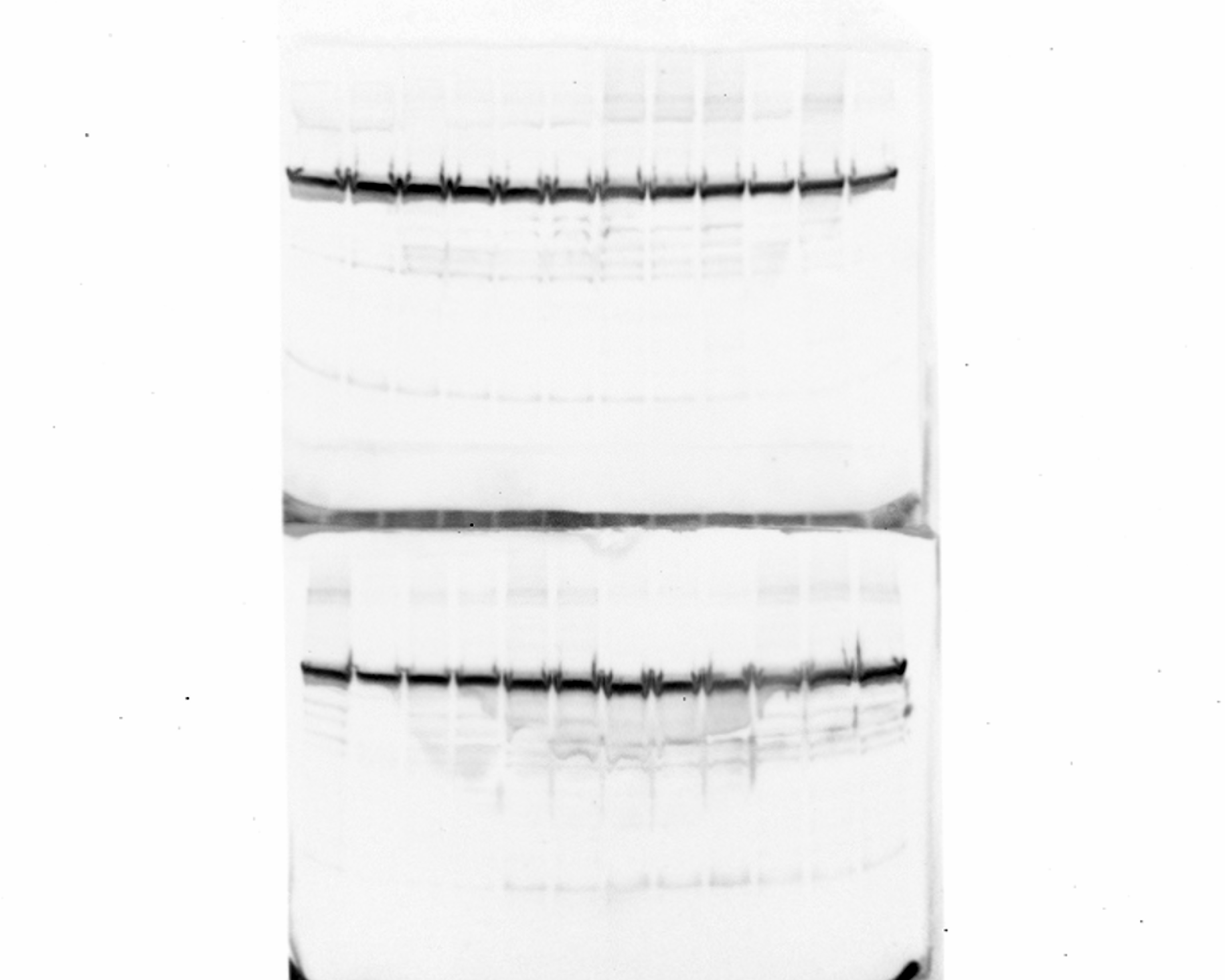

Supplement: S1 Data — (ZIP) [file pgen.1011059.s014.zip › SIdata/Figure 3 + S2/Fig3+S2B+S2C+S2D_WB RpoS+RpoSLac chase phosphate starvation+recovery/lmbchemidoc 2023-10-04 15h19m30s(DyLight 800).tif]

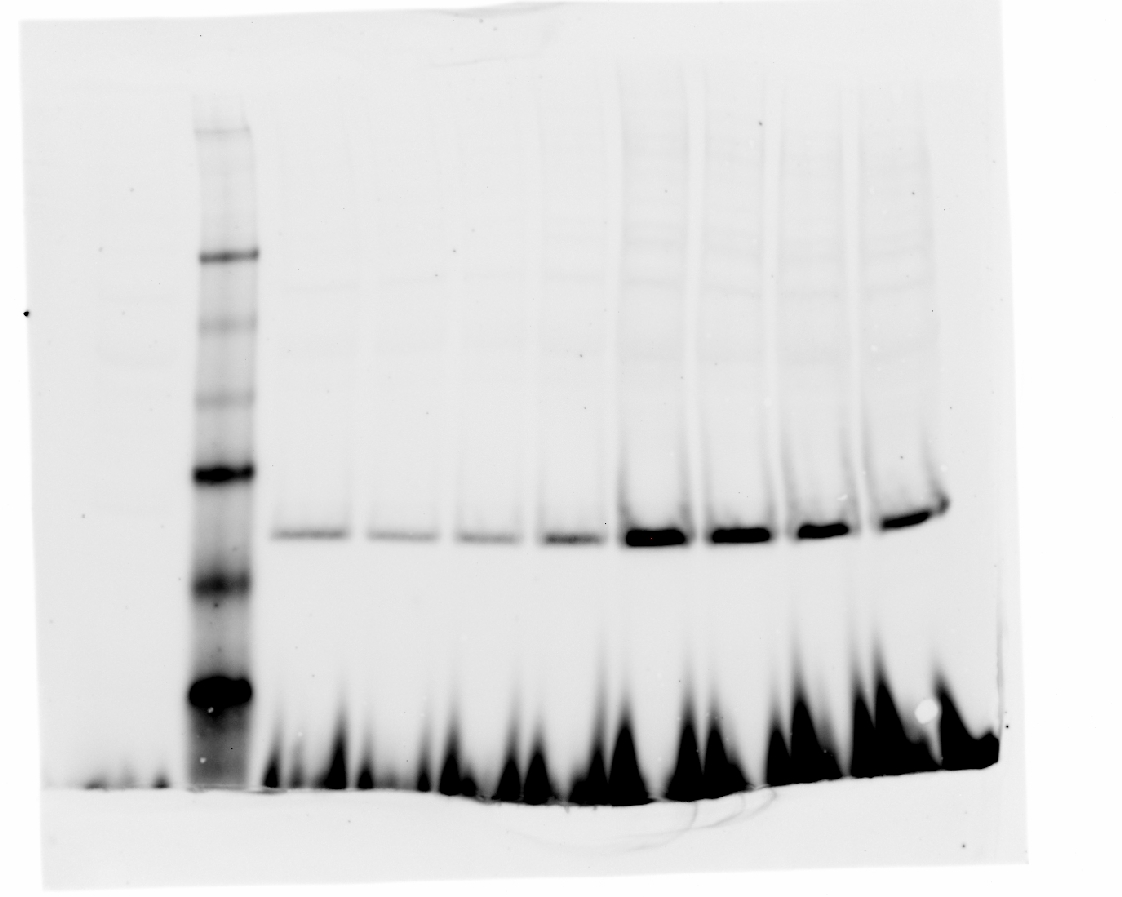

Supplement: S1 Data — (ZIP) [file pgen.1011059.s014.zip › SIdata/Figure 4 + S3/4C+S3D_WB IraPSPA/Flag.jpg]

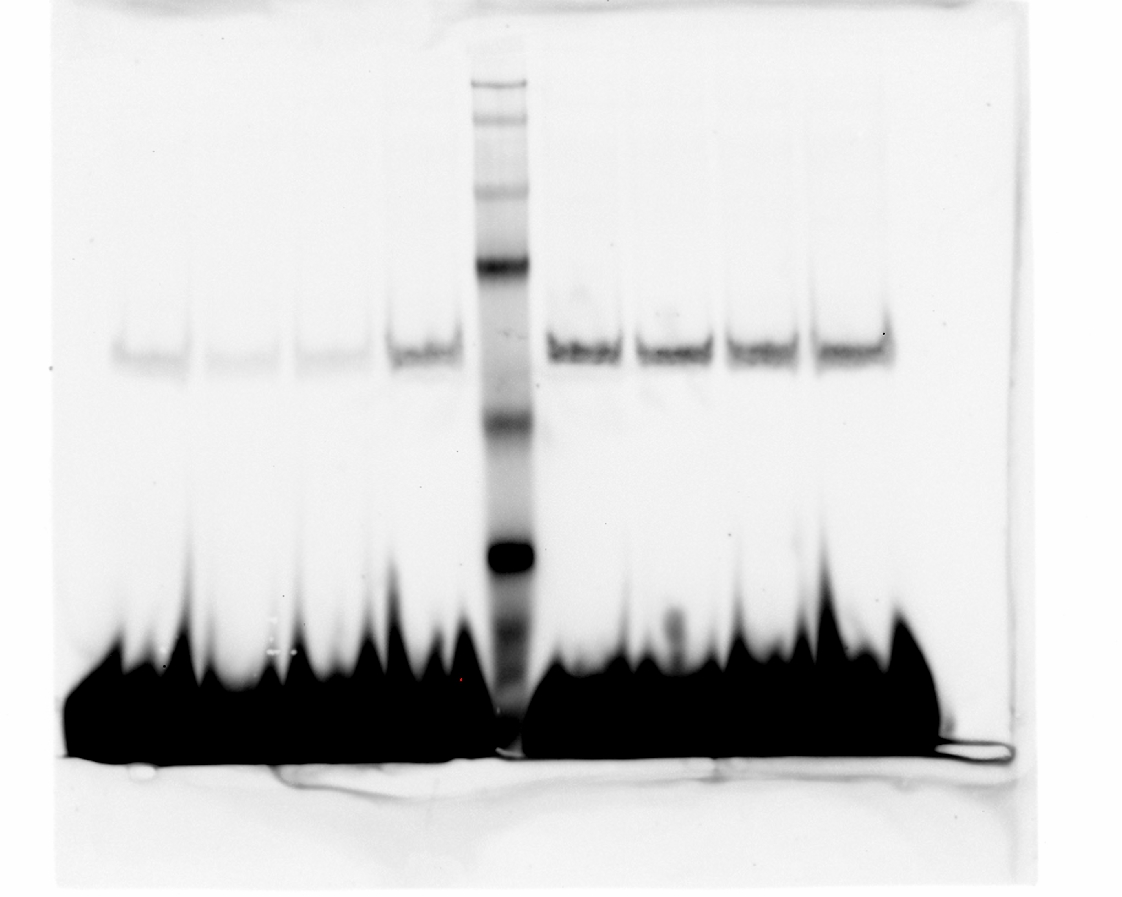

Supplement: S1 Data — (ZIP) [file pgen.1011059.s014.zip › SIdata/Figure 4 + S3/4C+S3D_WB IraPSPA/lmbchemidoc 2021-06-01 14h29m25s(DyLight 800).jpg]

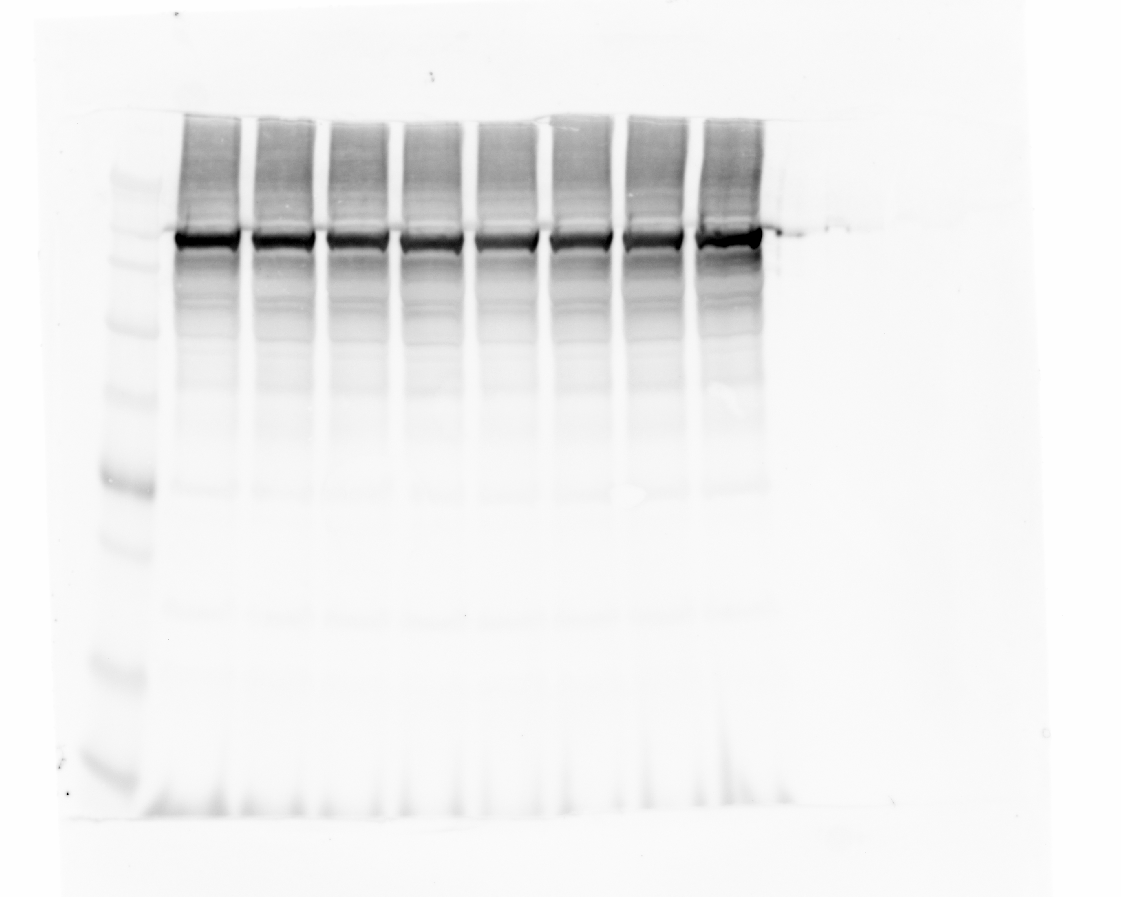

Supplement: S1 Data — (ZIP) [file pgen.1011059.s014.zip › SIdata/Figure 4 + S3/4D+S3E_WB IraPSPAChase/lmbchemidoc 2023-09-11 16h29m13s(StarBright B700).tif]

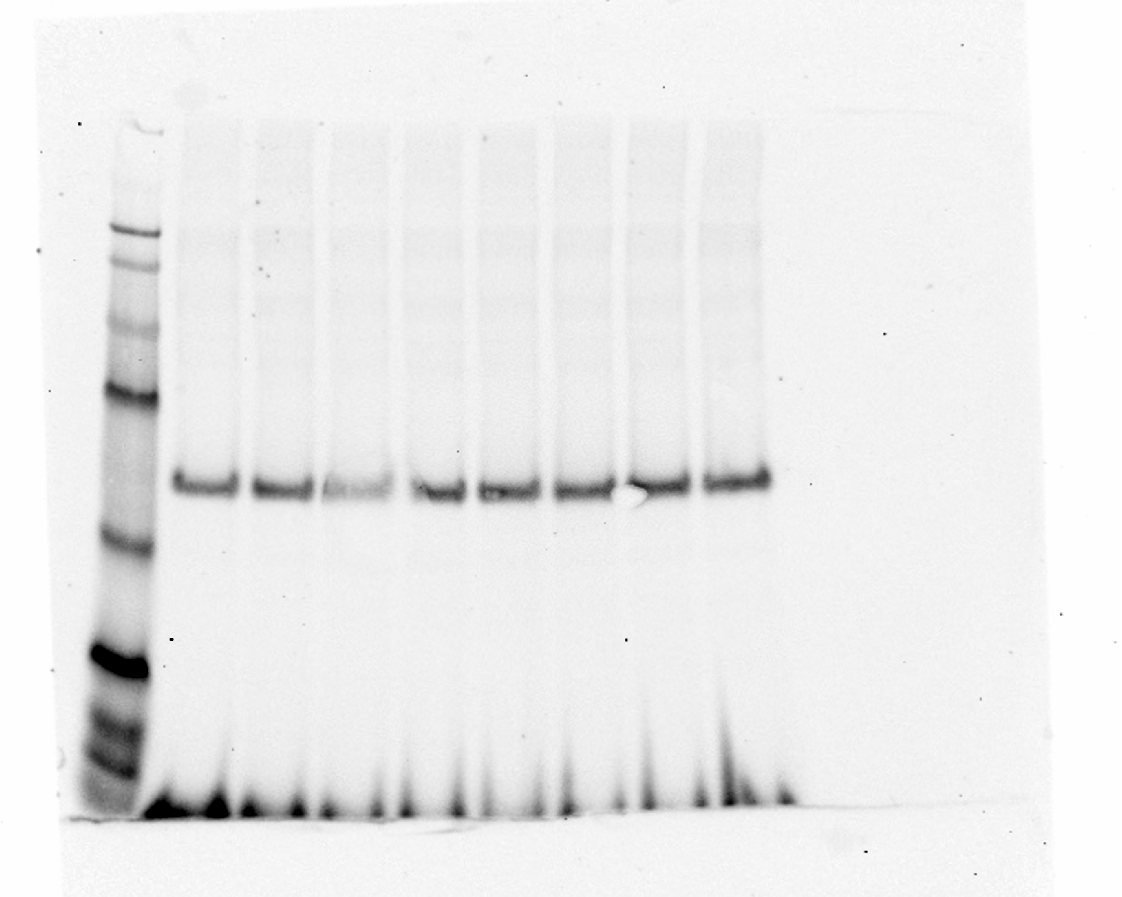

Supplement: S1 Data — (ZIP) [file pgen.1011059.s014.zip › SIdata/Figure 4 + S3/4D+S3E_WB IraPSPAChase/lmbchemidoc 2023-09-11 16h29m13s(DyLight 800).tif]

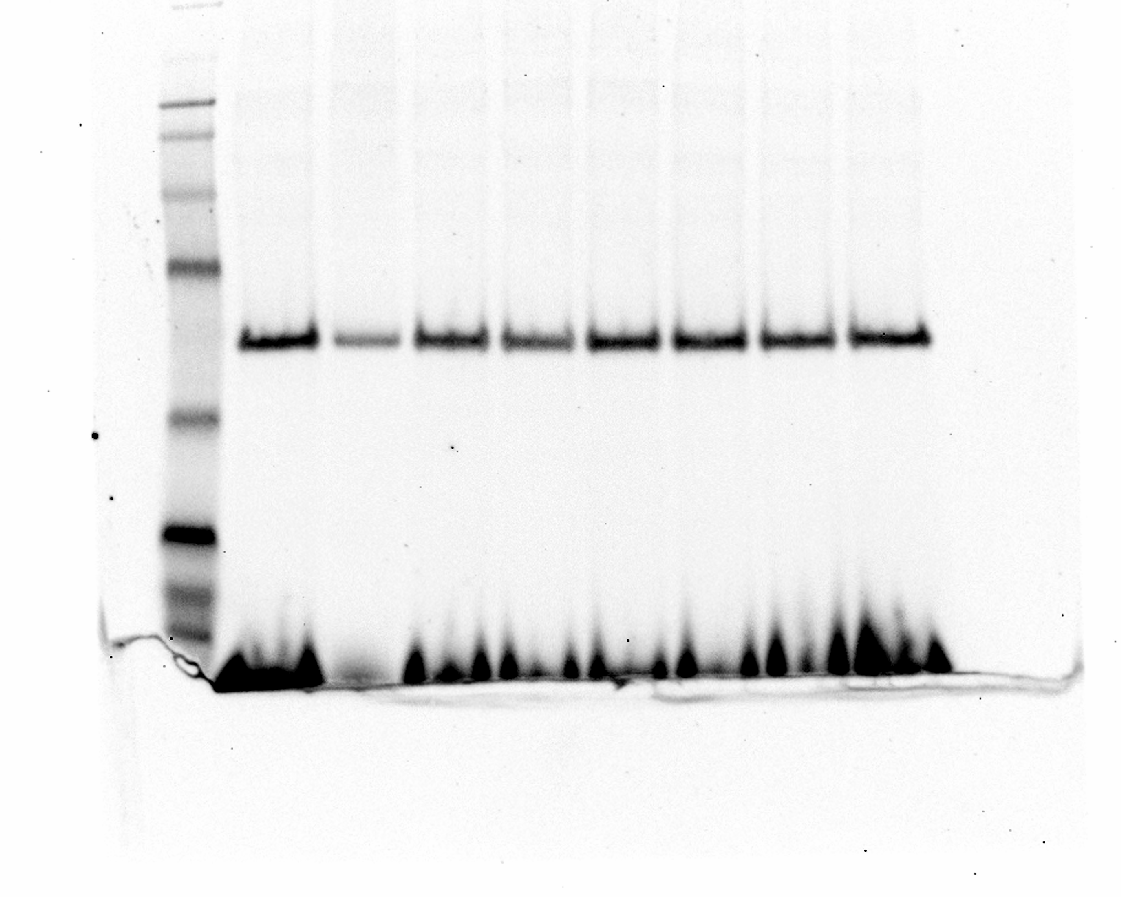

Supplement: S1 Data — (ZIP) [file pgen.1011059.s014.zip › SIdata/Figure 4 + S3/4D+S3E_WB IraPSPAChase/lmbchemidoc 2023-09-13 15h58m46s(DyLight 800).tif]

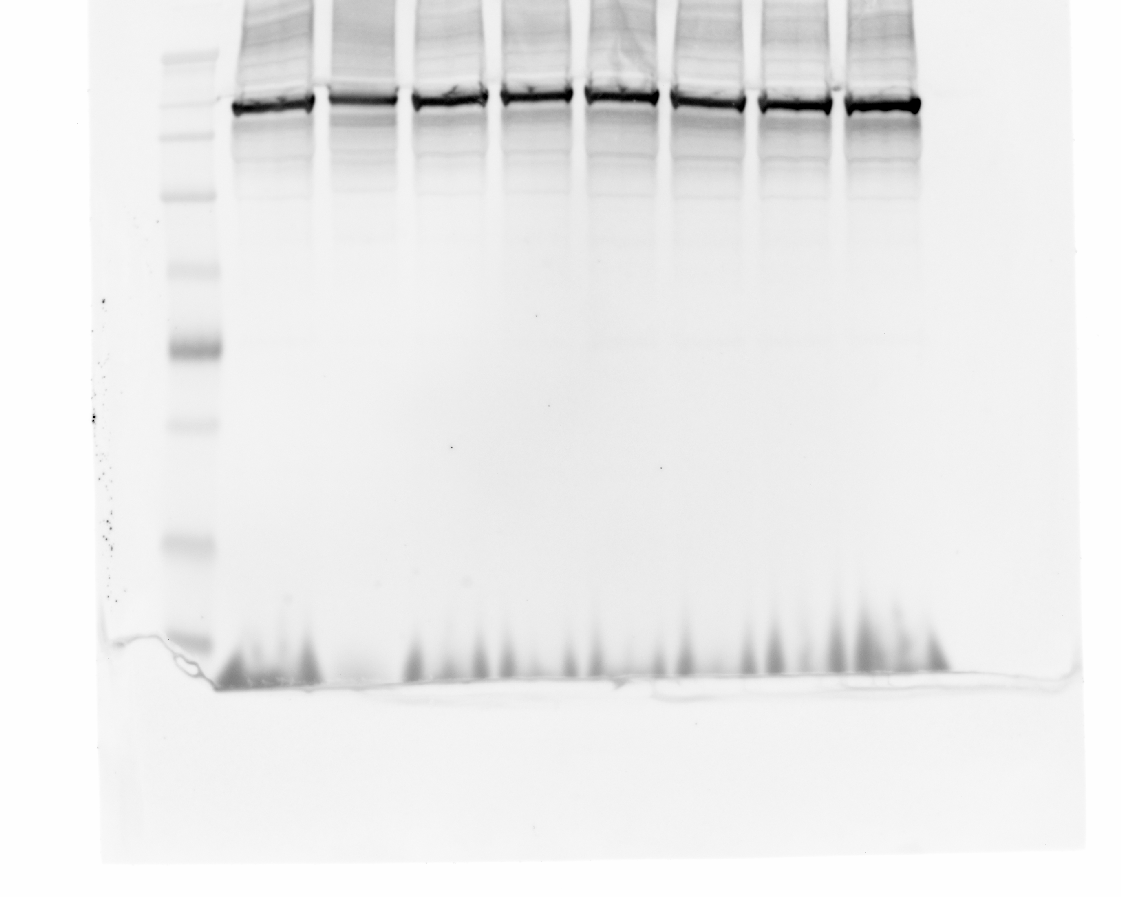

Supplement: S1 Data — (ZIP) [file pgen.1011059.s014.zip › SIdata/Figure 4 + S3/4D+S3E_WB IraPSPAChase/lmbchemidoc 2023-09-13 15h58m46s(StarBright B700).tif]

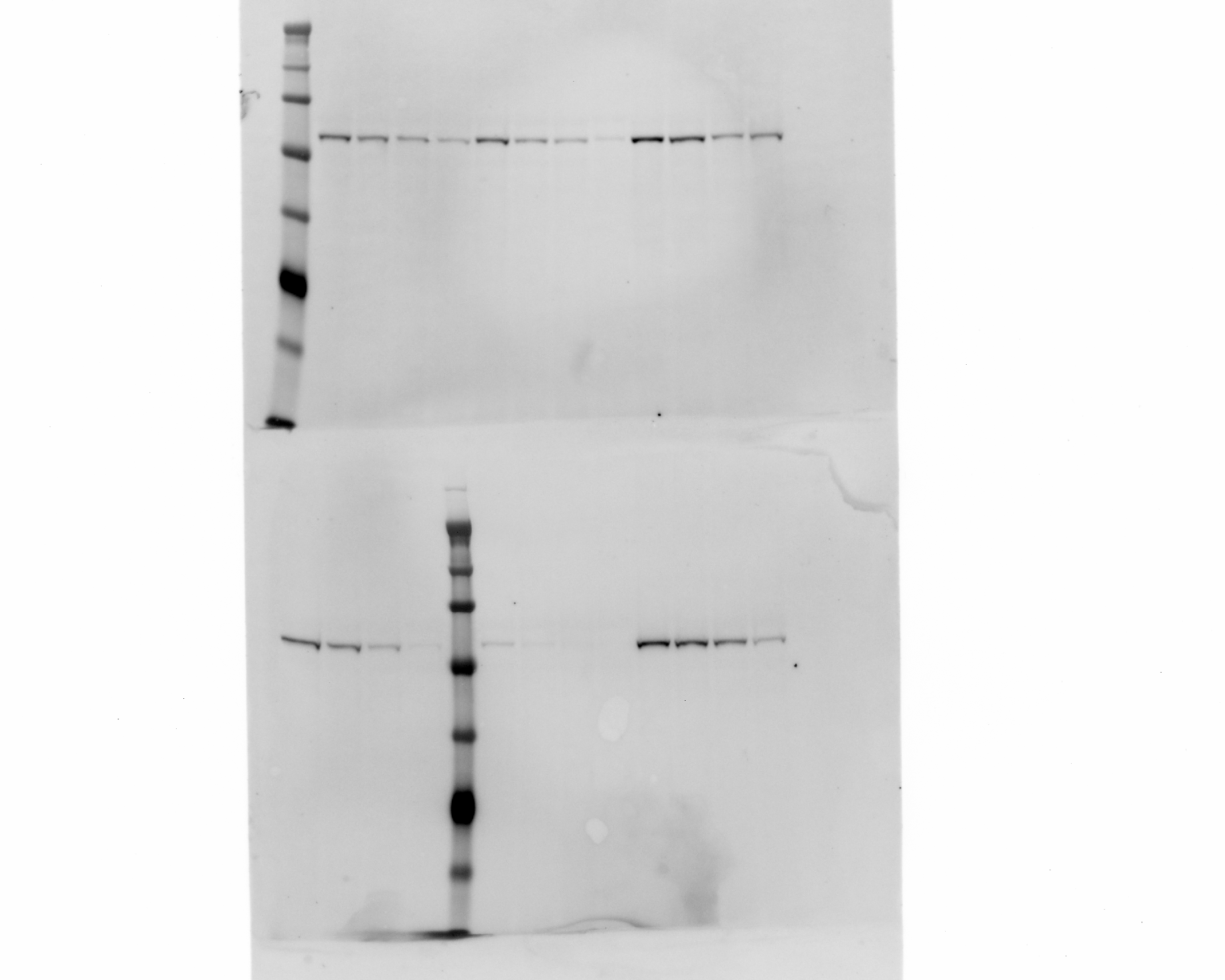

Supplement: S1 Data — (ZIP) [file pgen.1011059.s014.zip › SIdata/Figure 4 + S3/4E+S3F_WBRpoS/lmbchemidoc 2023-09-11 16h30m50s(StarBright B700).tif]

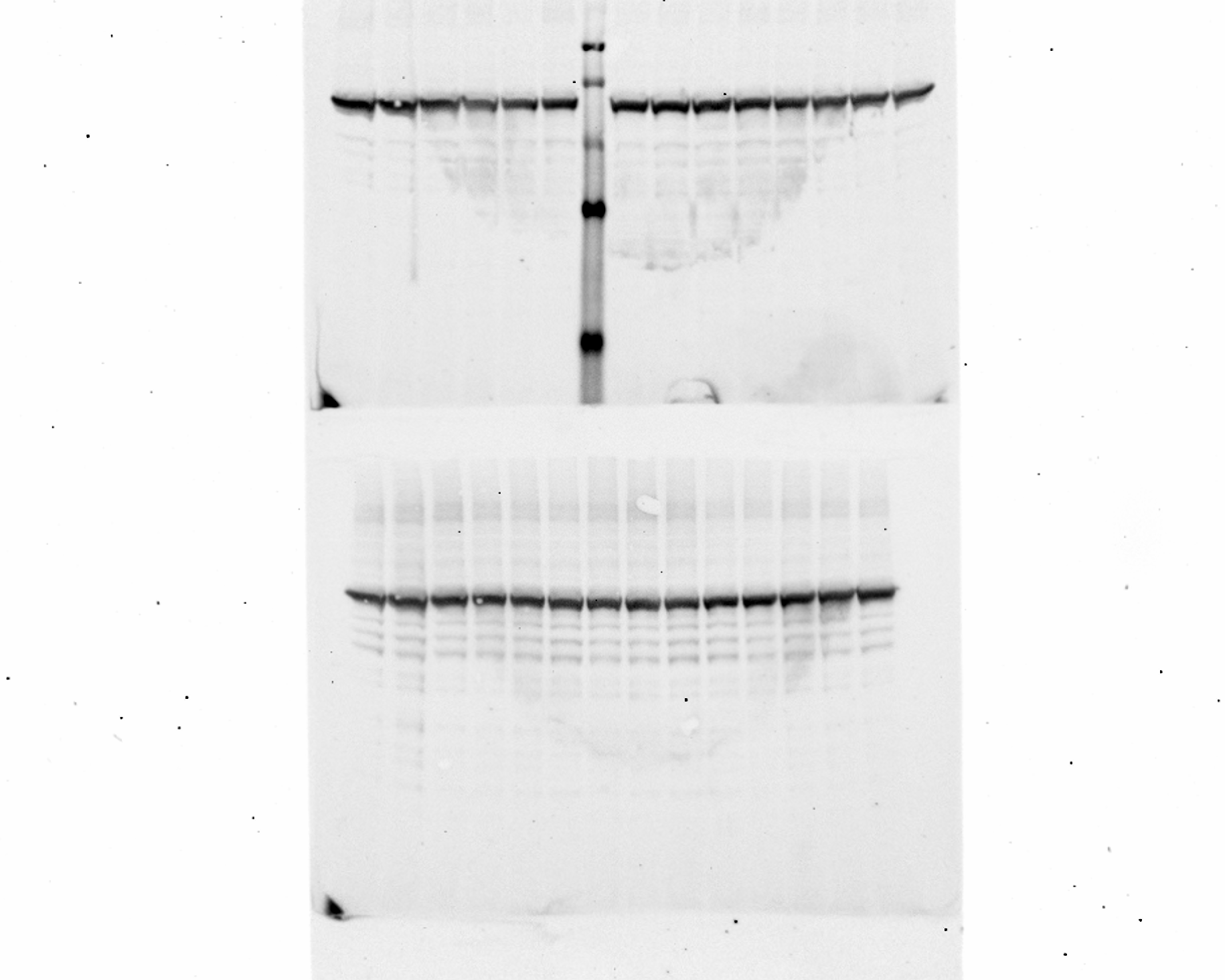

Supplement: S1 Data — (ZIP) [file pgen.1011059.s014.zip › SIdata/Figure 4 + S3/4E+S3F_WBRpoS/lmbchemidoc 2023-09-13 16h01m20s(DyLight 800).tif]

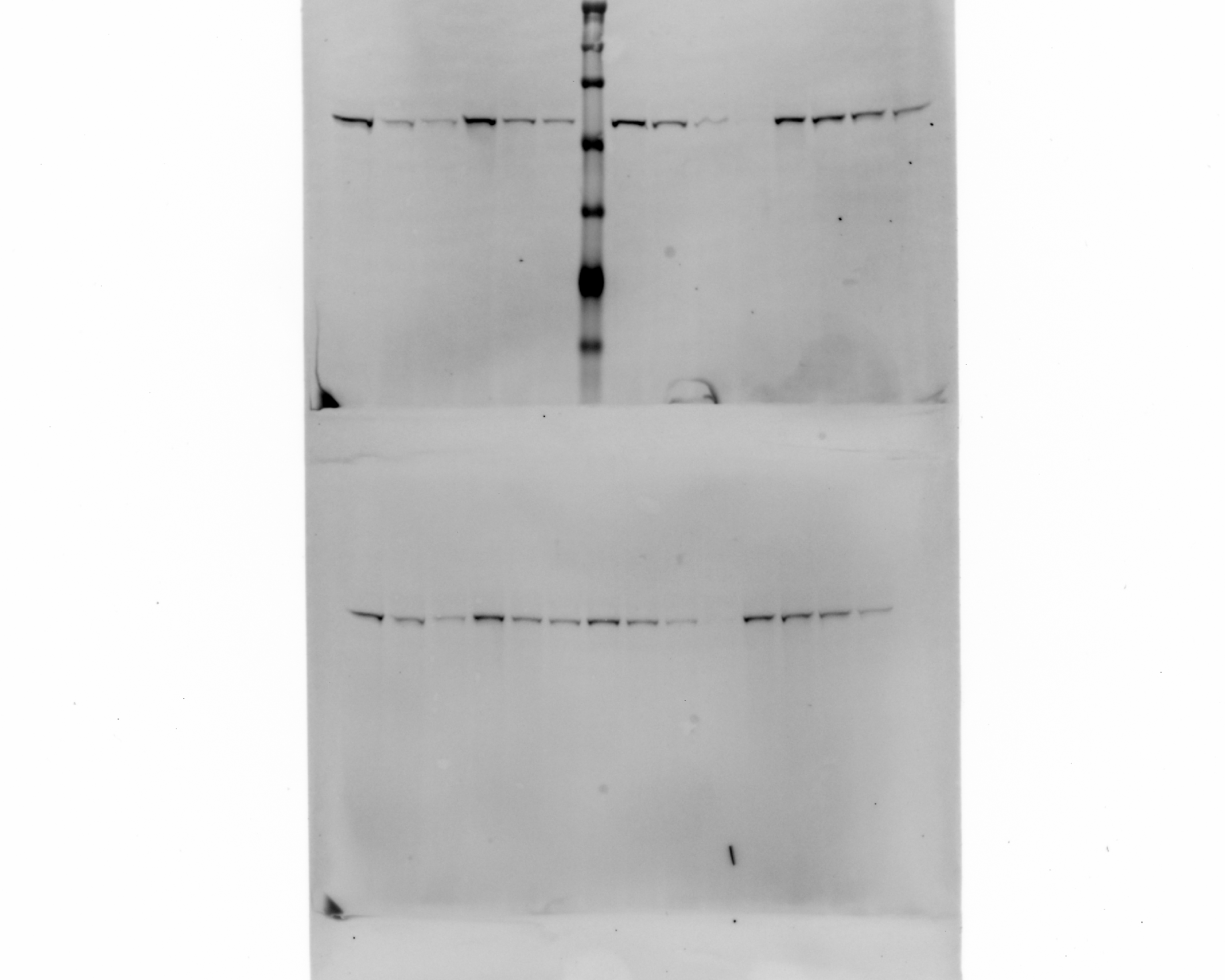

Supplement: S1 Data — (ZIP) [file pgen.1011059.s014.zip › SIdata/Figure 4 + S3/4E+S3F_WBRpoS/lmbchemidoc 2023-09-13 16h01m20s(StarBright B700).tif]

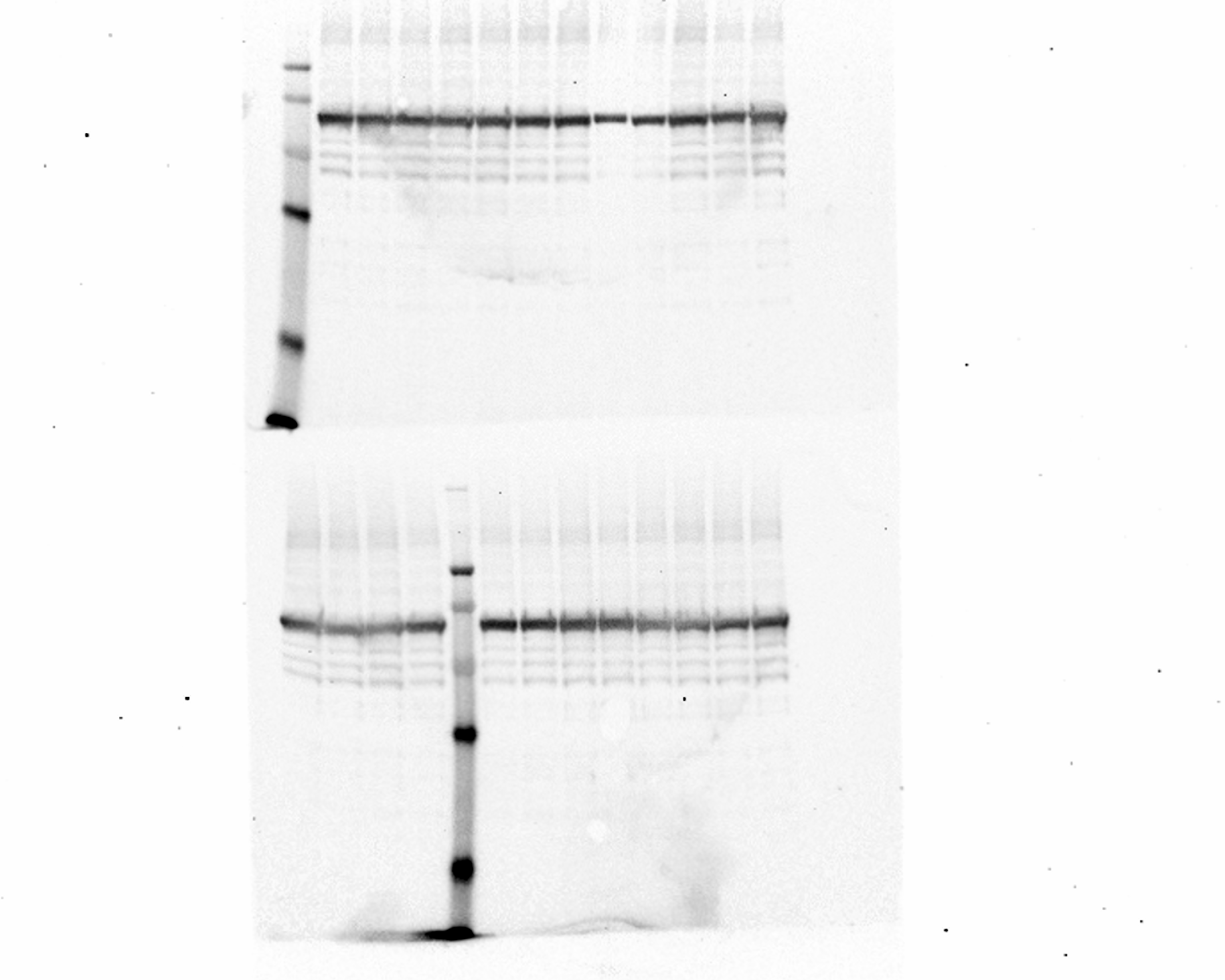

Supplement: S1 Data — (ZIP) [file pgen.1011059.s014.zip › SIdata/Figure 4 + S3/4E+S3F_WBRpoS/lmbchemidoc 2023-09-11 16h30m50s(DyLight 800).tif]

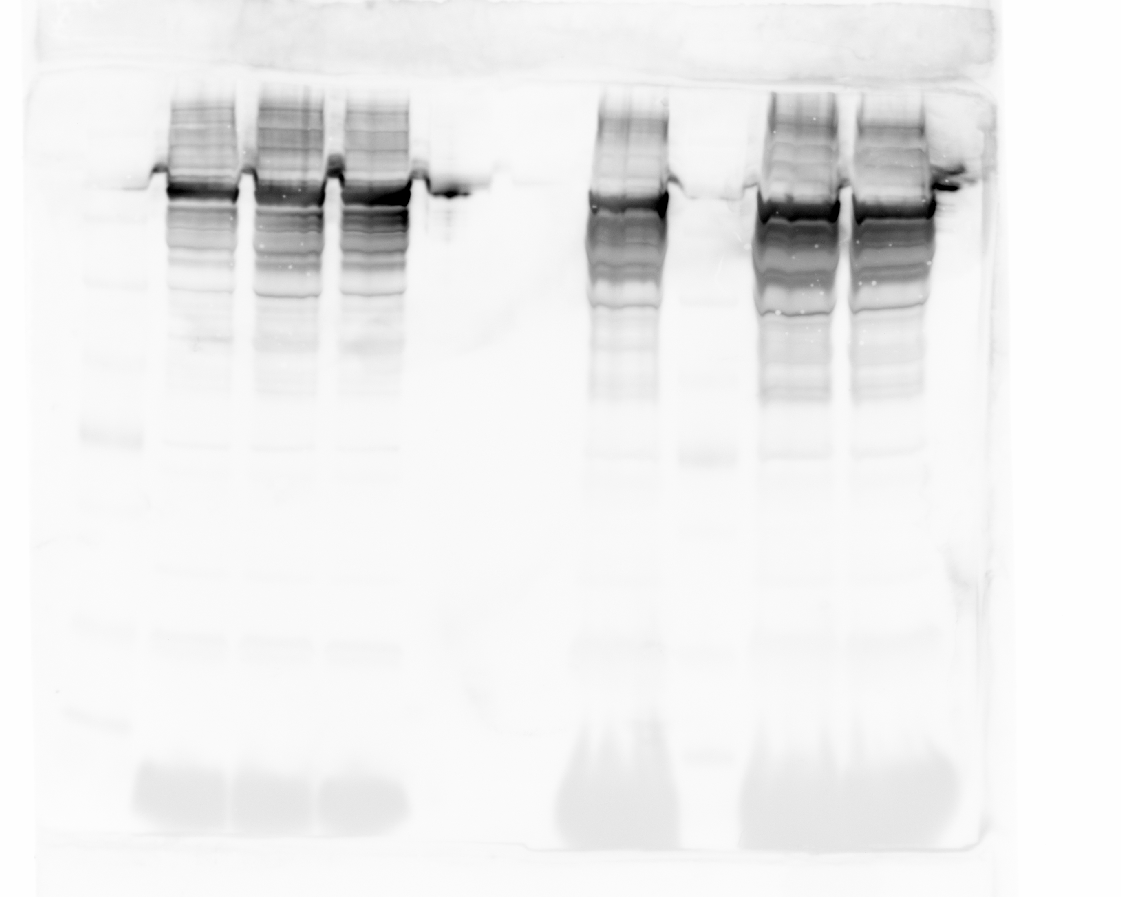

Supplement: S1 Data — (ZIP) [file pgen.1011059.s014.zip › SIdata/Figure 5 + S5 + S6/S6A - WB anti-T18crl/lmbchemidoc 2023-09-21 16h13m31s(StarBright B700).tif]

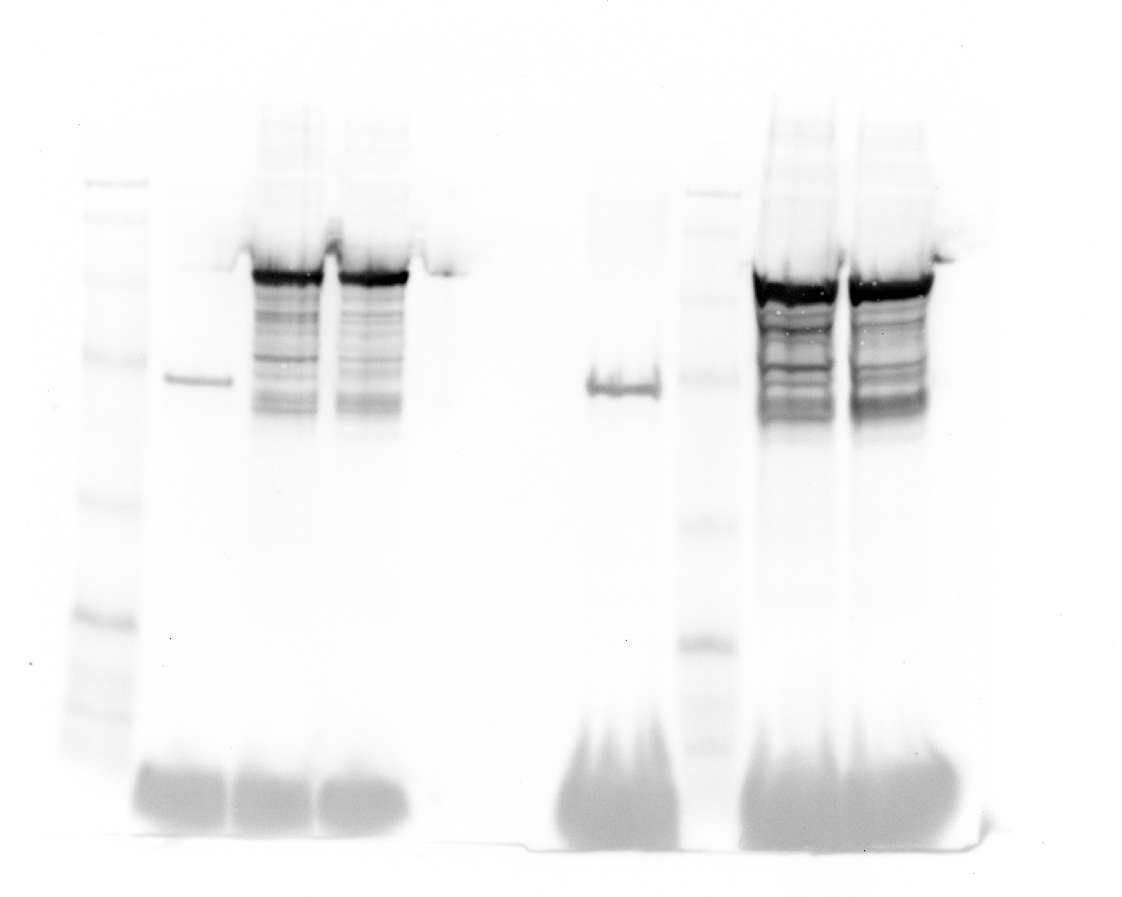

Supplement: S1 Data — (ZIP) [file pgen.1011059.s014.zip › SIdata/Figure 5 + S5 + S6/S6A - WB anti-T18crl/lmbchemidoc 2023-09-21 16h13m31s(DyLight 800).tif]

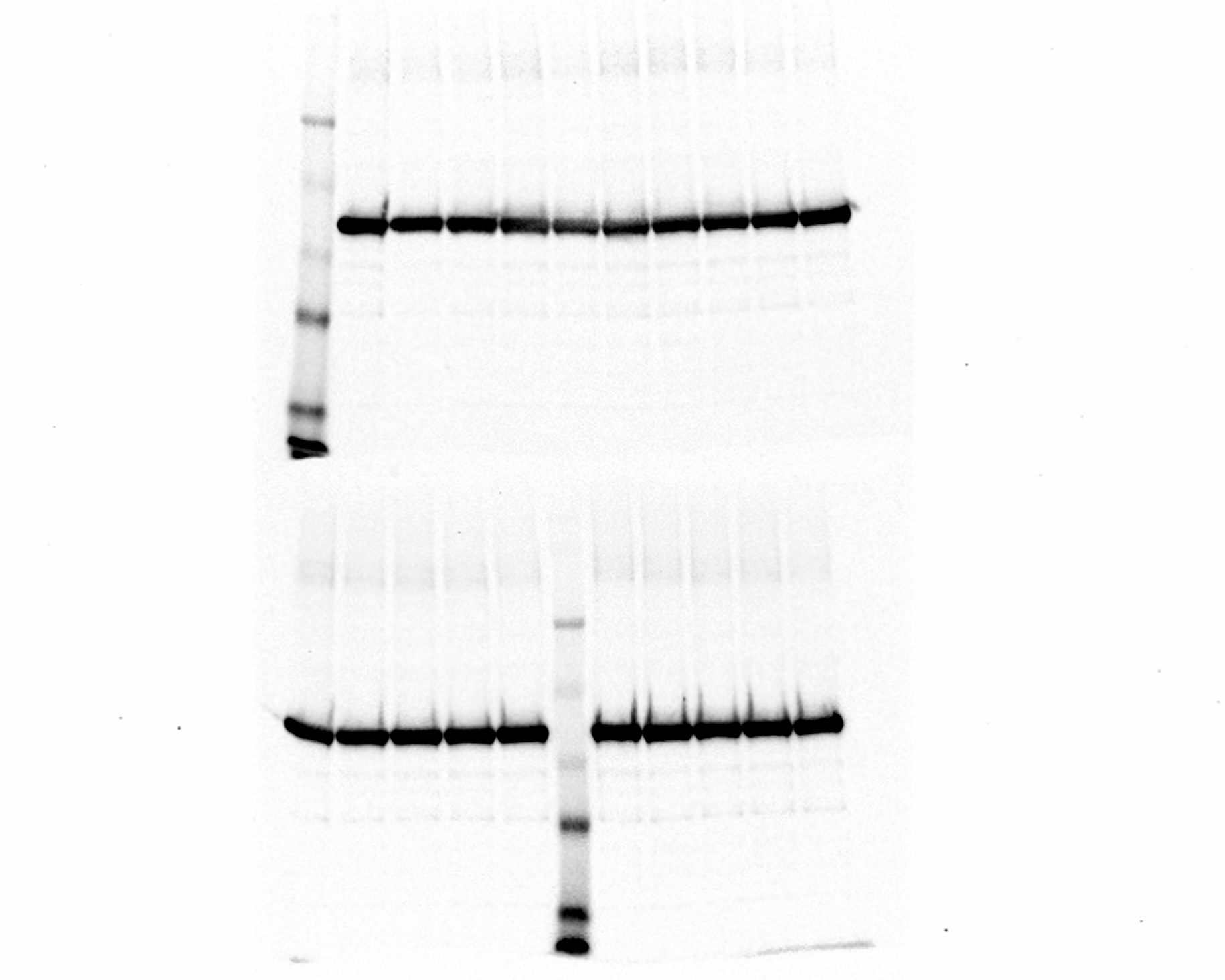

Supplement: S1 Data — (ZIP) [file pgen.1011059.s014.zip › SIdata/Figure 7 + S8/Fig7+S8_WB/Set 2/lmbchemidoc 2021-05-21 16h17m14s(DyLight 800).tif]

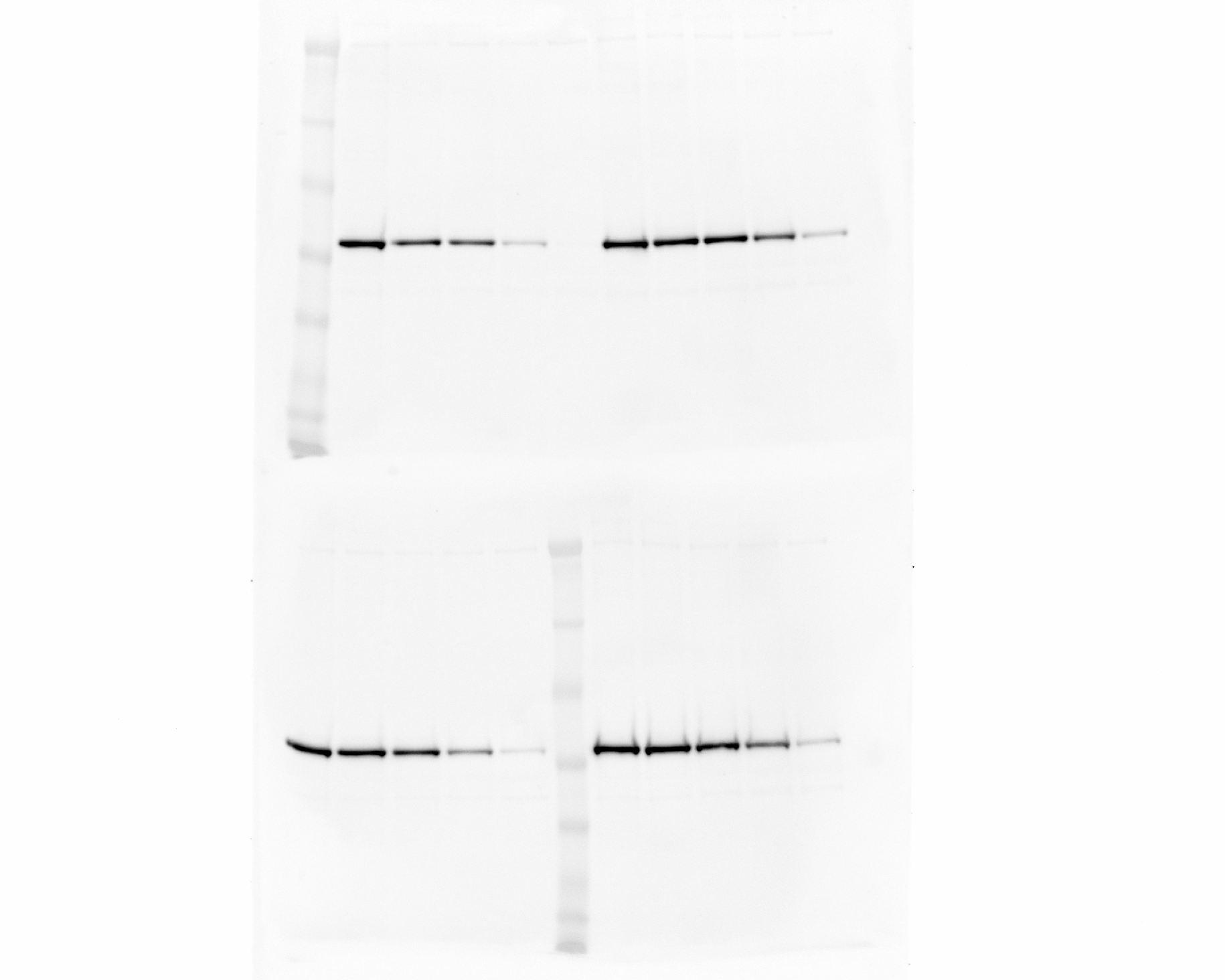

Supplement: S1 Data — (ZIP) [file pgen.1011059.s014.zip › SIdata/Figure 7 + S8/Fig7+S8_WB/Set 2/lmbchemidoc 2021-05-21 16h17m14s(StarBright B700).tif]

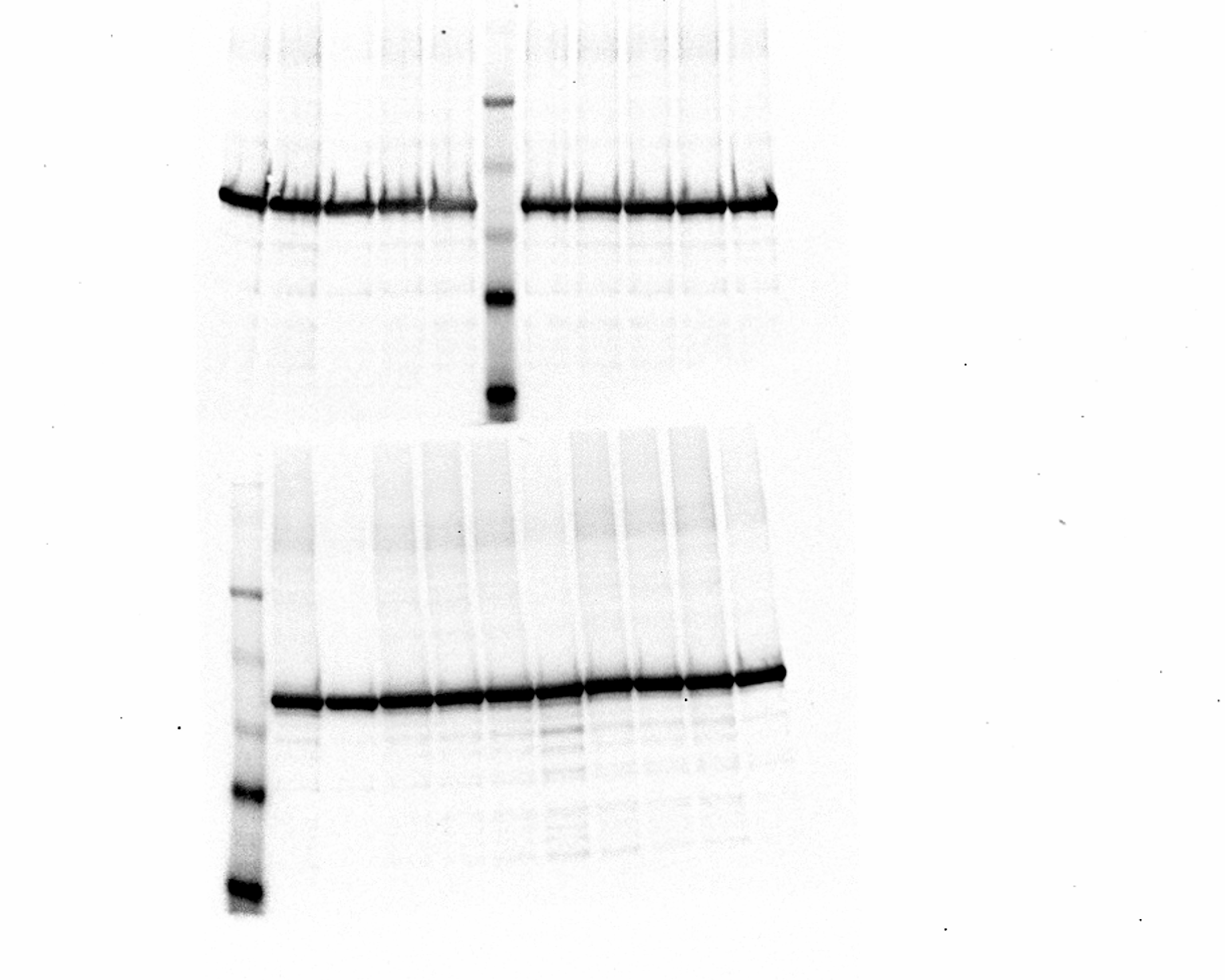

Supplement: S1 Data — (ZIP) [file pgen.1011059.s014.zip › SIdata/Figure 7 + S8/Fig7+S8_WB/Set 3/lmbchemidoc 2021-05-26 18h23m50s(DyLight 800).tif]

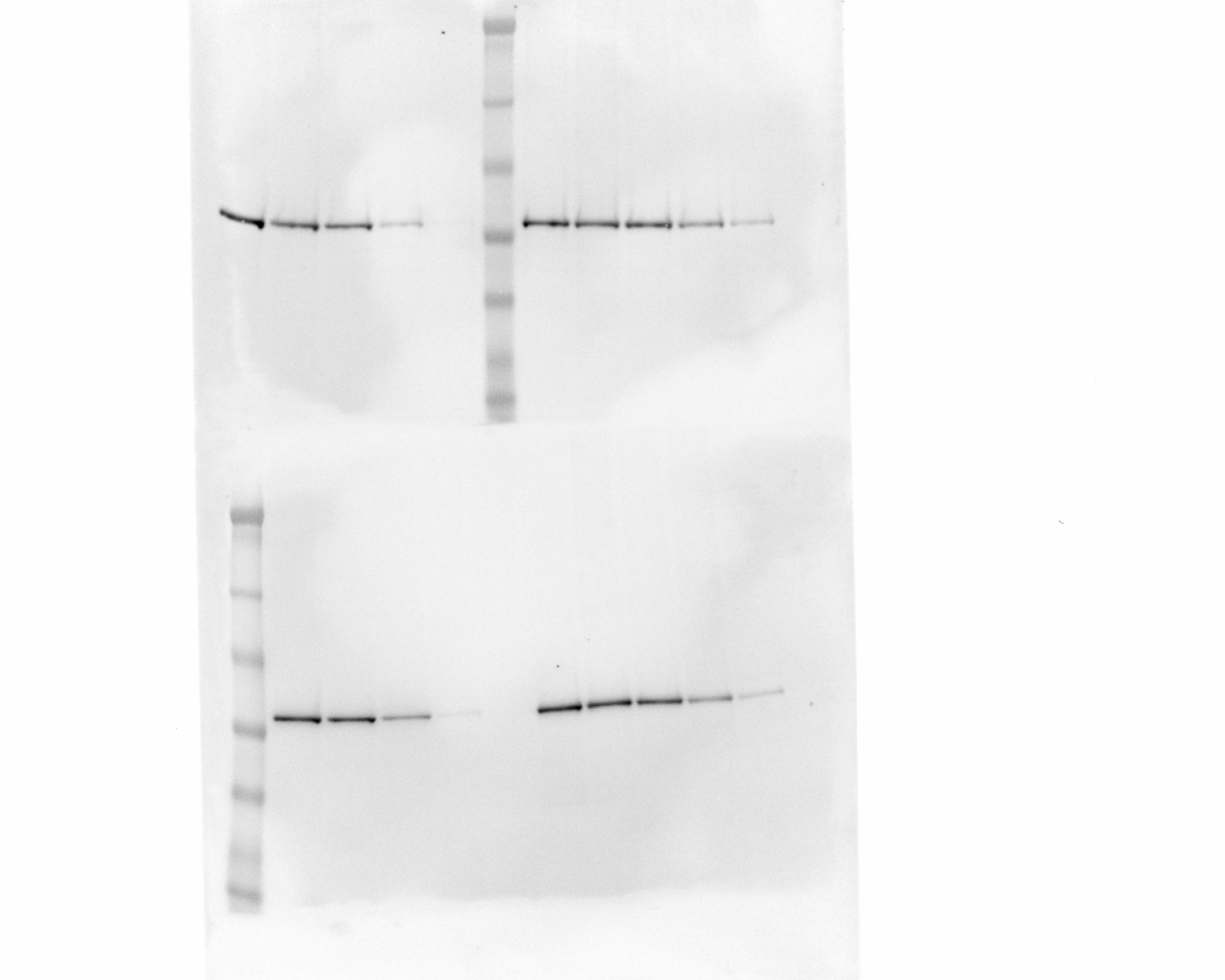

Supplement: S1 Data — (ZIP) [file pgen.1011059.s014.zip › SIdata/Figure 7 + S8/Fig7+S8_WB/Set 3/lmbchemidoc 2021-05-26 18h23m50s(StarBright B700).tif]

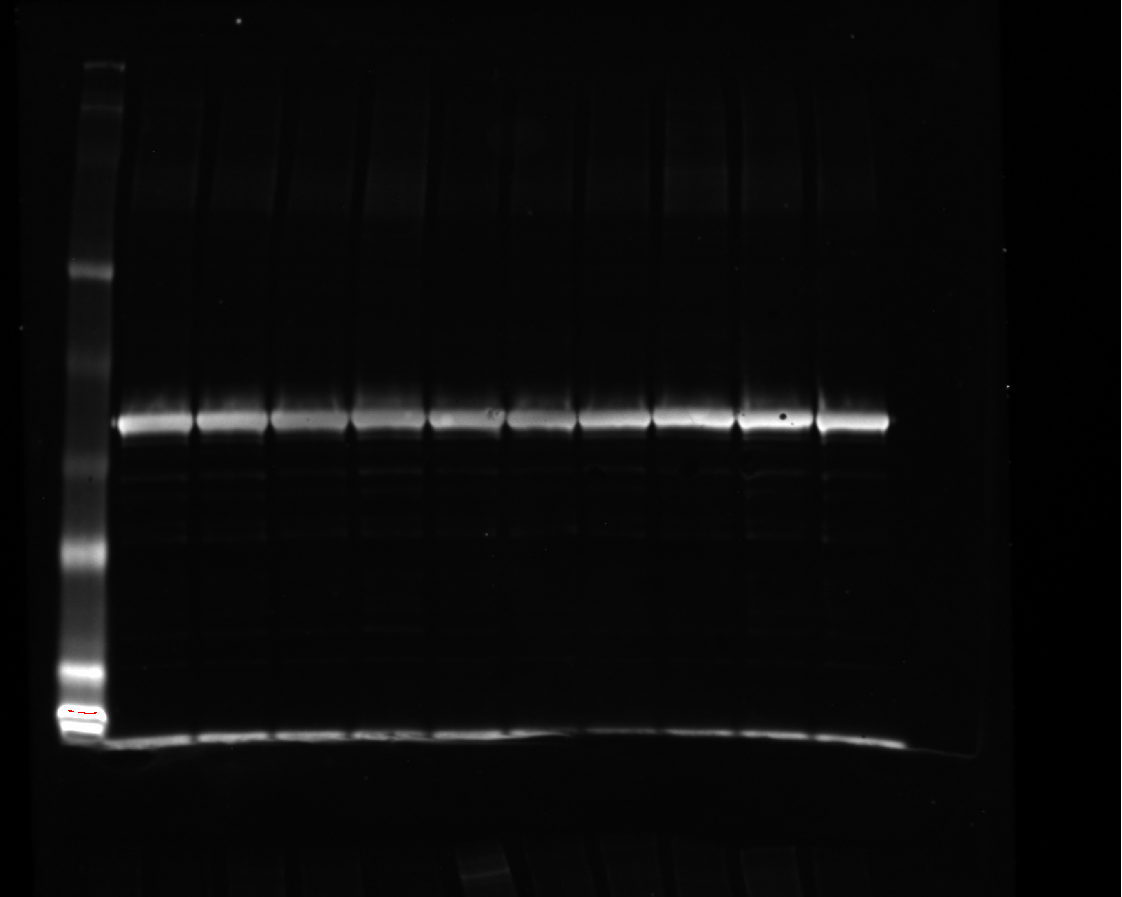

Supplement: S1 Data — (ZIP) [file pgen.1011059.s014.zip › SIdata/Figure 7 + S8/Fig7+S8_WB/Set 1/lmbchemidoc 2021-04-16 13h53m09s(DyLight 800).tif]

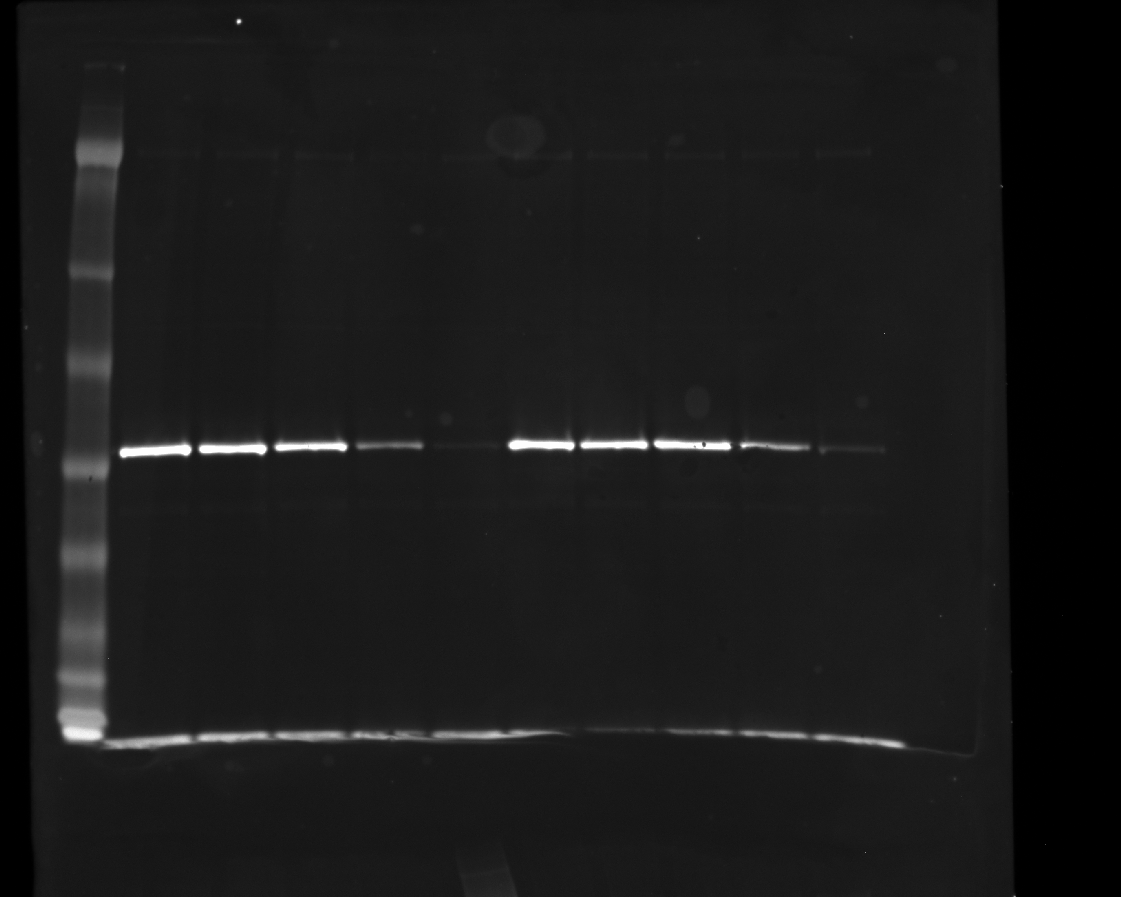

Supplement: S1 Data — (ZIP) [file pgen.1011059.s014.zip › SIdata/Figure 7 + S8/Fig7+S8_WB/Set 1/lmbchemidoc 2021-04-16 13h55m37s(StarBright B700).tif]

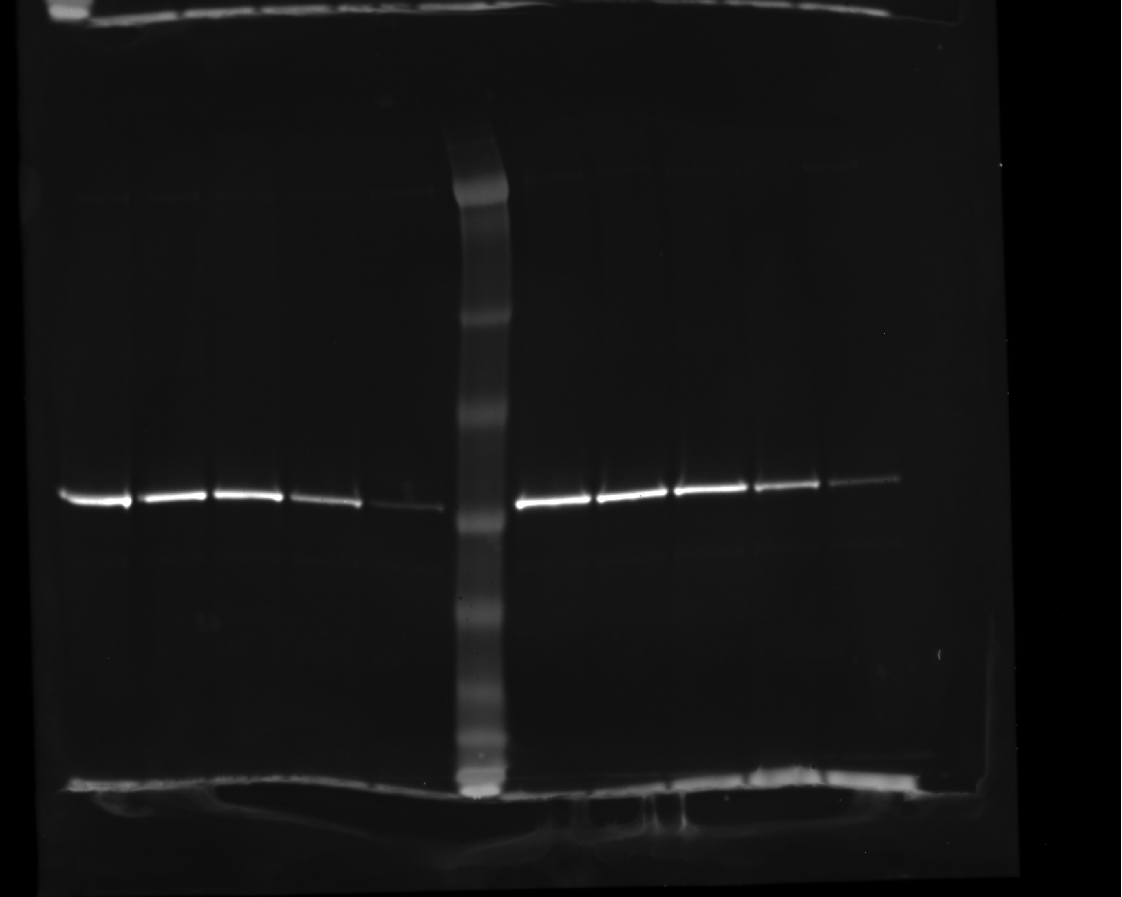

Supplement: S1 Data — (ZIP) [file pgen.1011059.s014.zip › SIdata/Figure 7 + S8/Fig7+S8_WB/Set 1/lmbchemidoc 2021-04-16 13h57m56s(StarBright B700).tif]

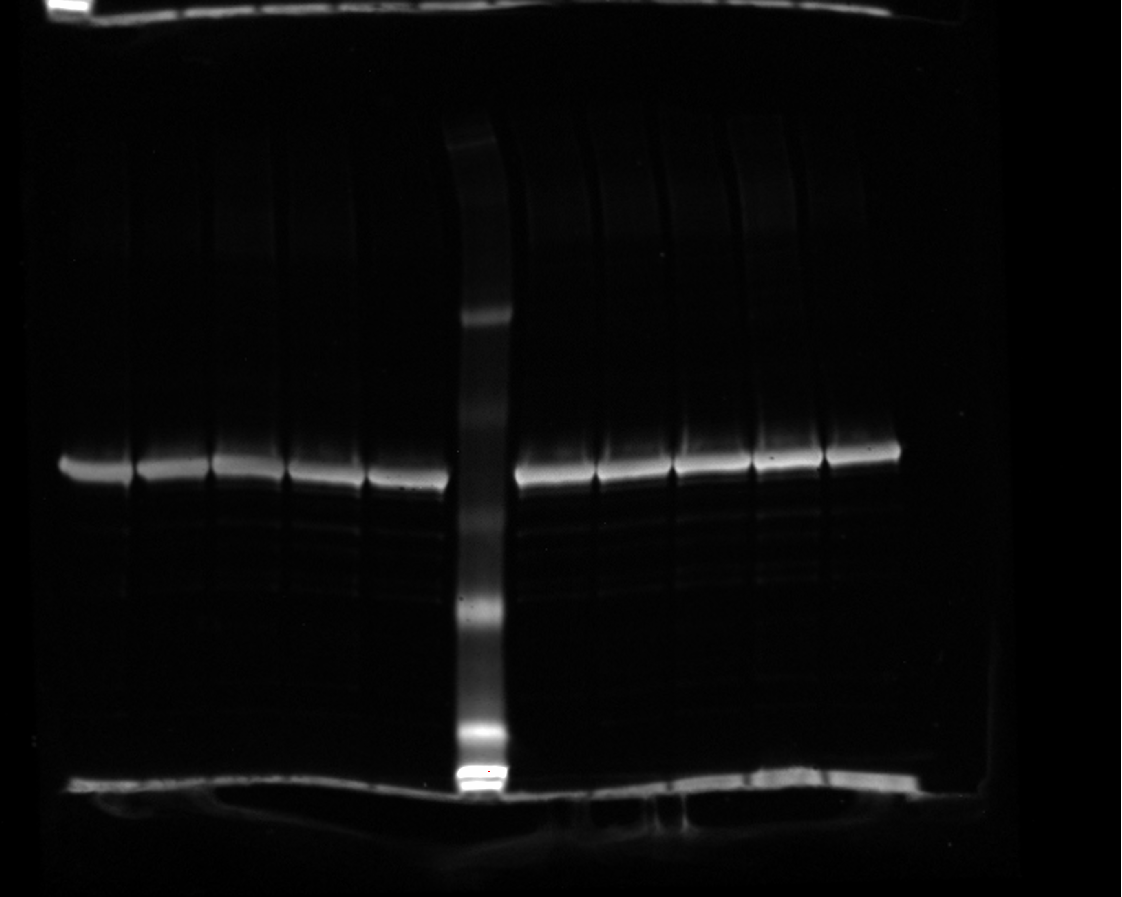

Supplement: S1 Data — (ZIP) [file pgen.1011059.s014.zip › SIdata/Figure 7 + S8/Fig7+S8_WB/Set 1/lmbchemidoc 2021-04-16 13h57m56s(DyLight 800).tif]

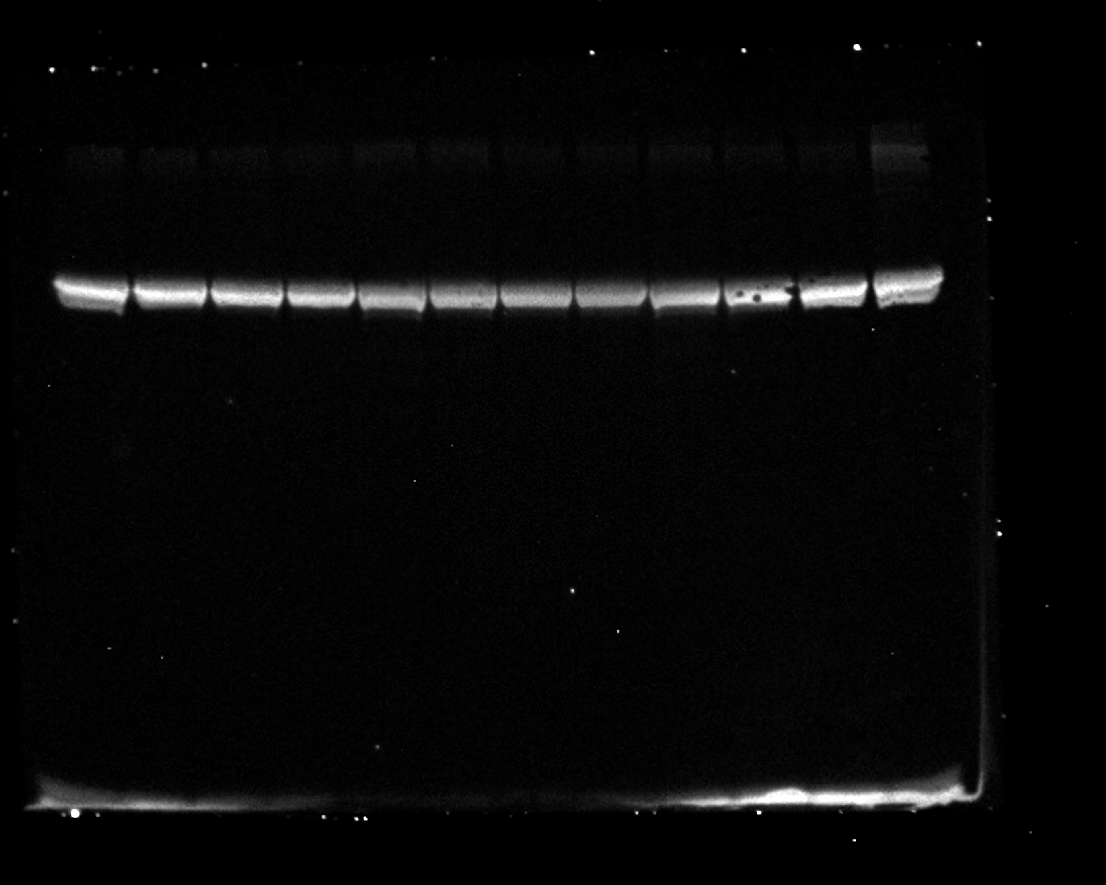

Supplement: S1 Data — (ZIP) [file pgen.1011059.s014.zip › SIdata/Figure 4 + S3/4BC+S3BC_WB RssB/Set 5/lmbchemidoc 2021-02-12 18h54m40s(DyLight 800).tif]

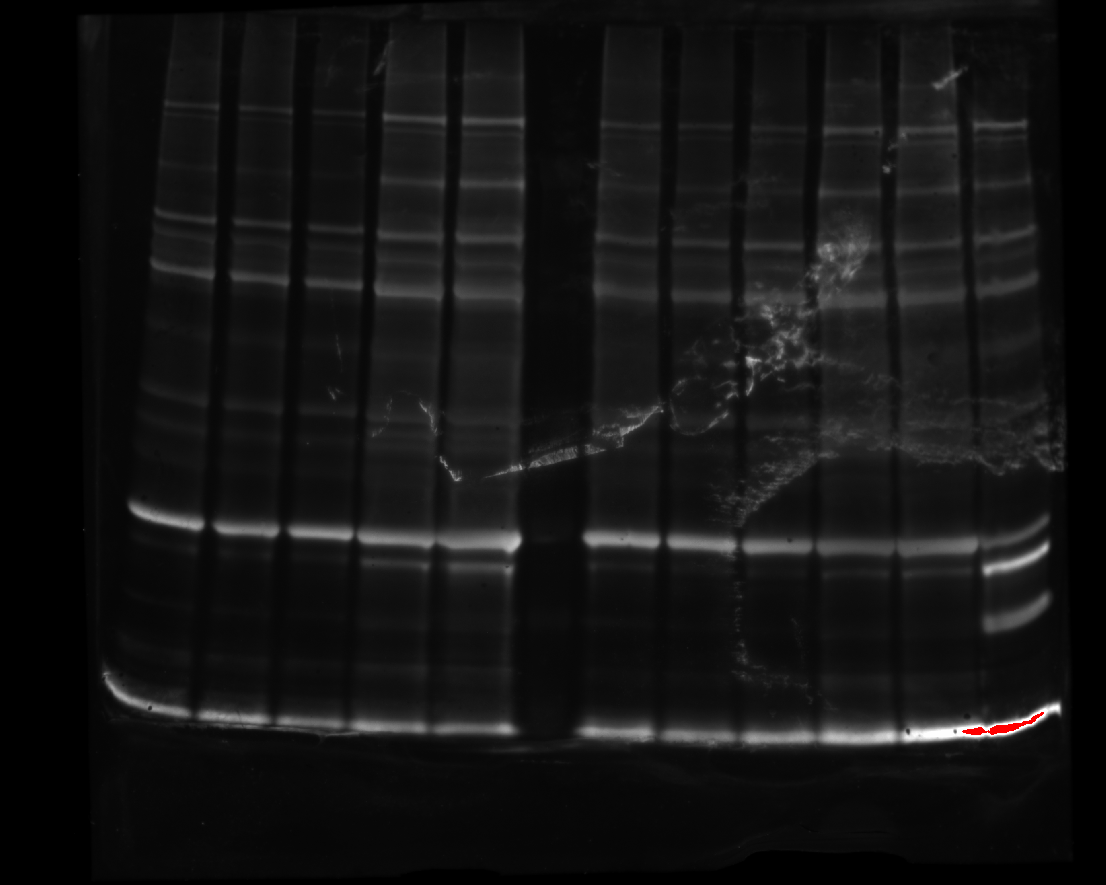

Supplement: S1 Data — (ZIP) [file pgen.1011059.s014.zip › SIdata/Figure 4 + S3/4BC+S3BC_WB RssB/Set 5/lmbchemidoc 2021-02-12 18h47m32s(StarBright B700).tif]

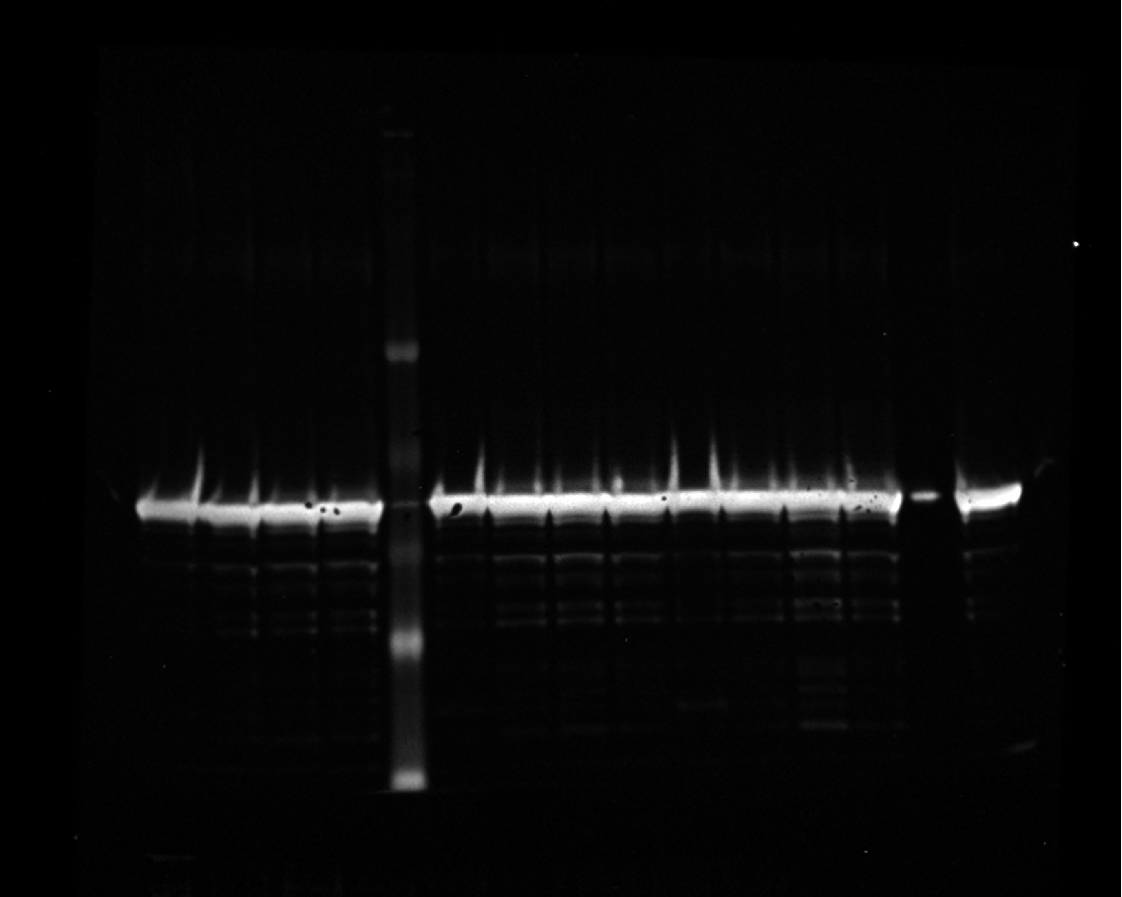

Supplement: S1 Data — (ZIP) [file pgen.1011059.s014.zip › SIdata/Figure 4 + S3/4BC+S3BC_WB RssB/Set 2/10sec(DyLight 800).tif]

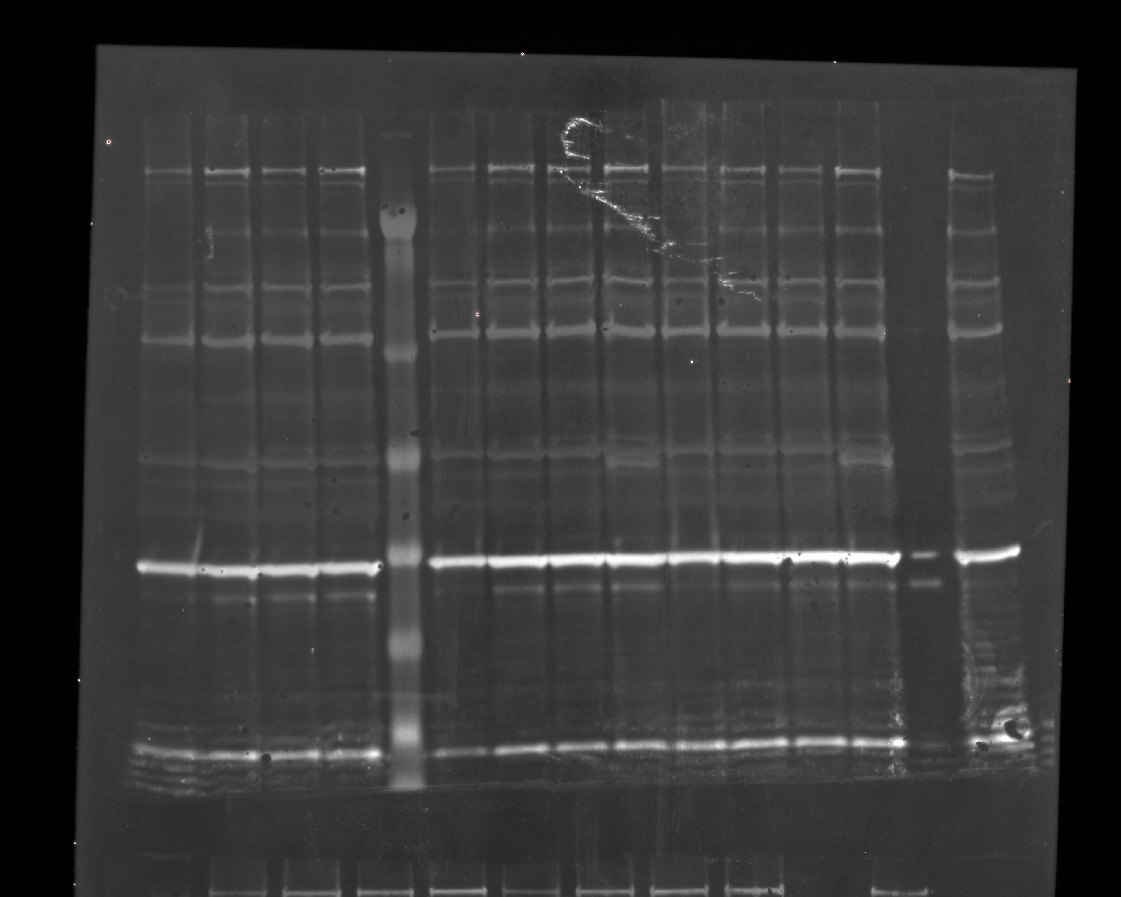

Supplement: S1 Data — (ZIP) [file pgen.1011059.s014.zip › SIdata/Figure 4 + S3/4BC+S3BC_WB RssB/Set 2/30sec(StarBright B700).tif]

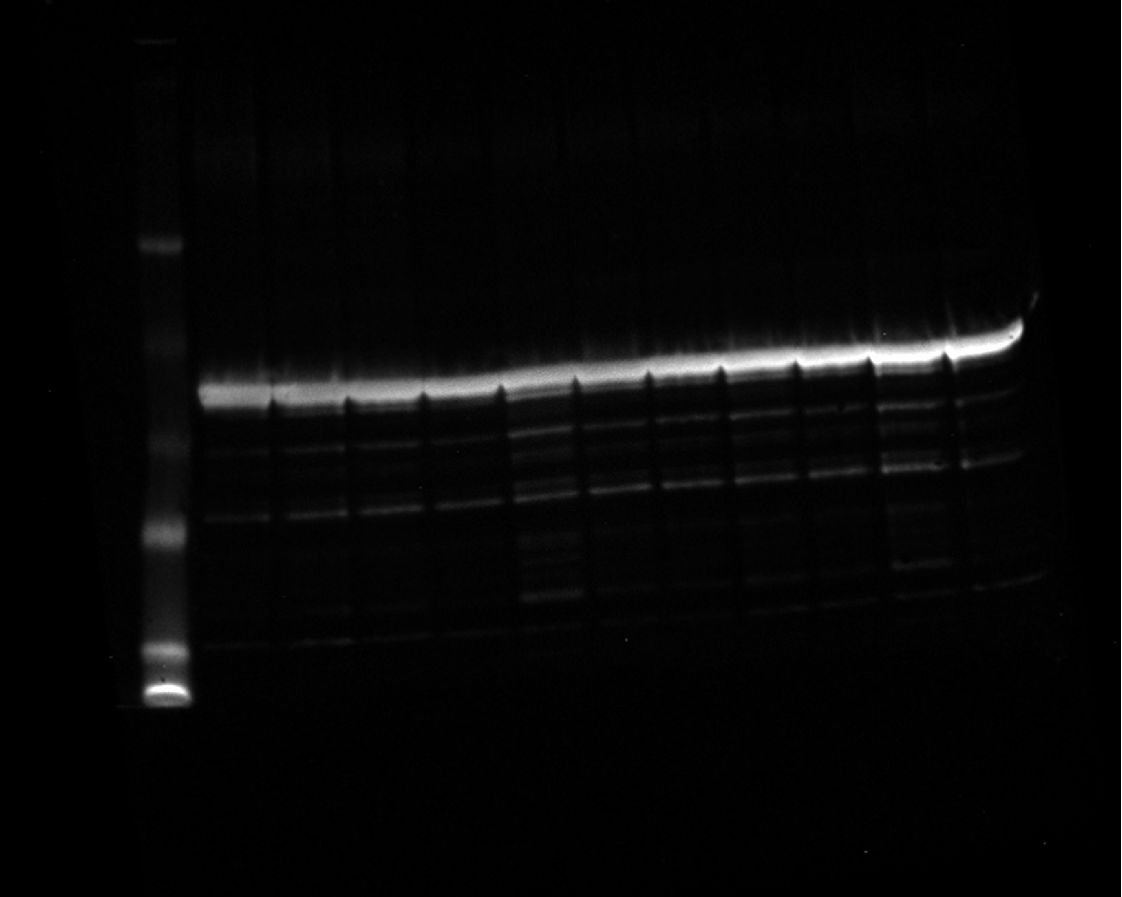

Supplement: S1 Data — (ZIP) [file pgen.1011059.s014.zip › SIdata/Figure 4 + S3/4BC+S3BC_WB RssB/Set 3/lmbchemidoc 2021-01-22 17h30m57s(DyLight 800).tif]

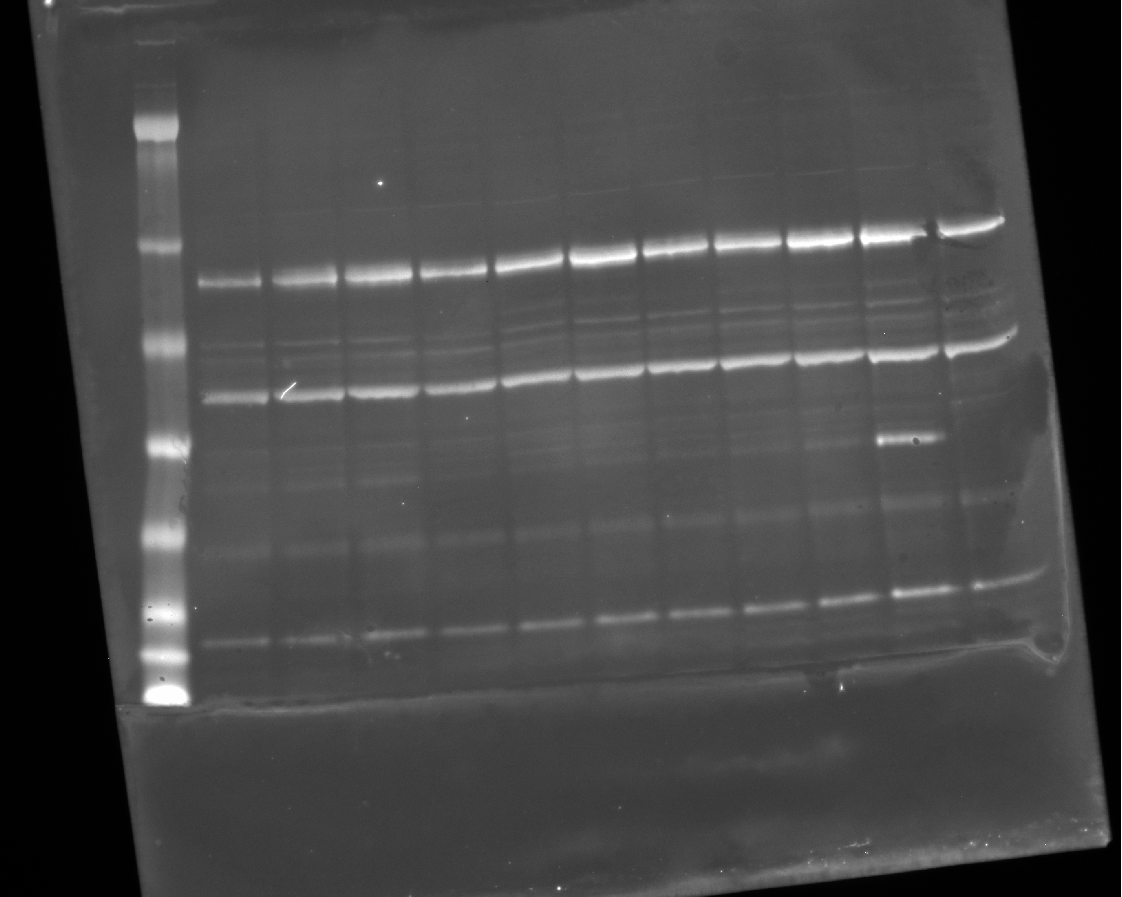

Supplement: S1 Data — (ZIP) [file pgen.1011059.s014.zip › SIdata/Figure 4 + S3/4BC+S3BC_WB RssB/Set 3/lmbchemidoc 2021-01-22 17h31m50s(StarBright B700).tif]

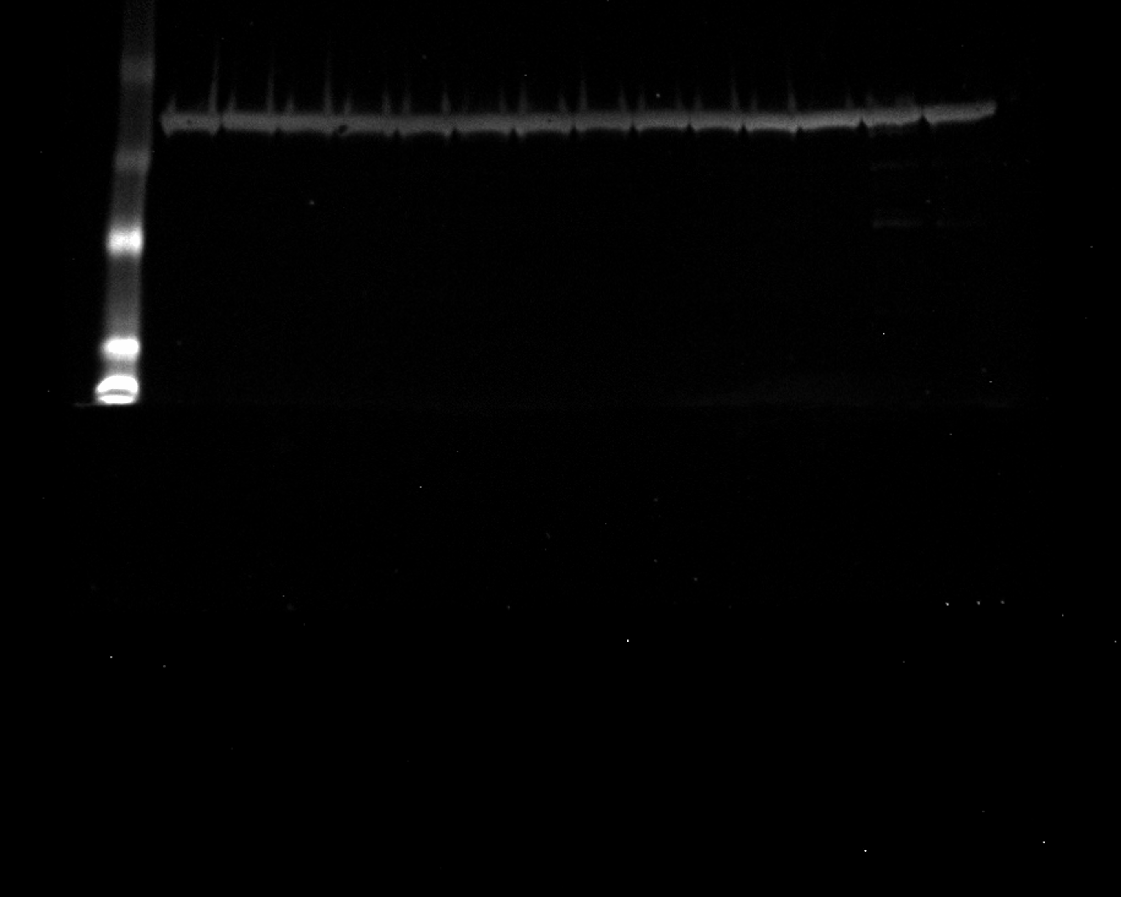

Supplement: S1 Data — (ZIP) [file pgen.1011059.s014.zip › SIdata/Figure 4 + S3/4BC+S3BC_WB RssB/Set 4/lmbchemidoc 2021-01-29 17h41m54s(DyLight 800).tif]

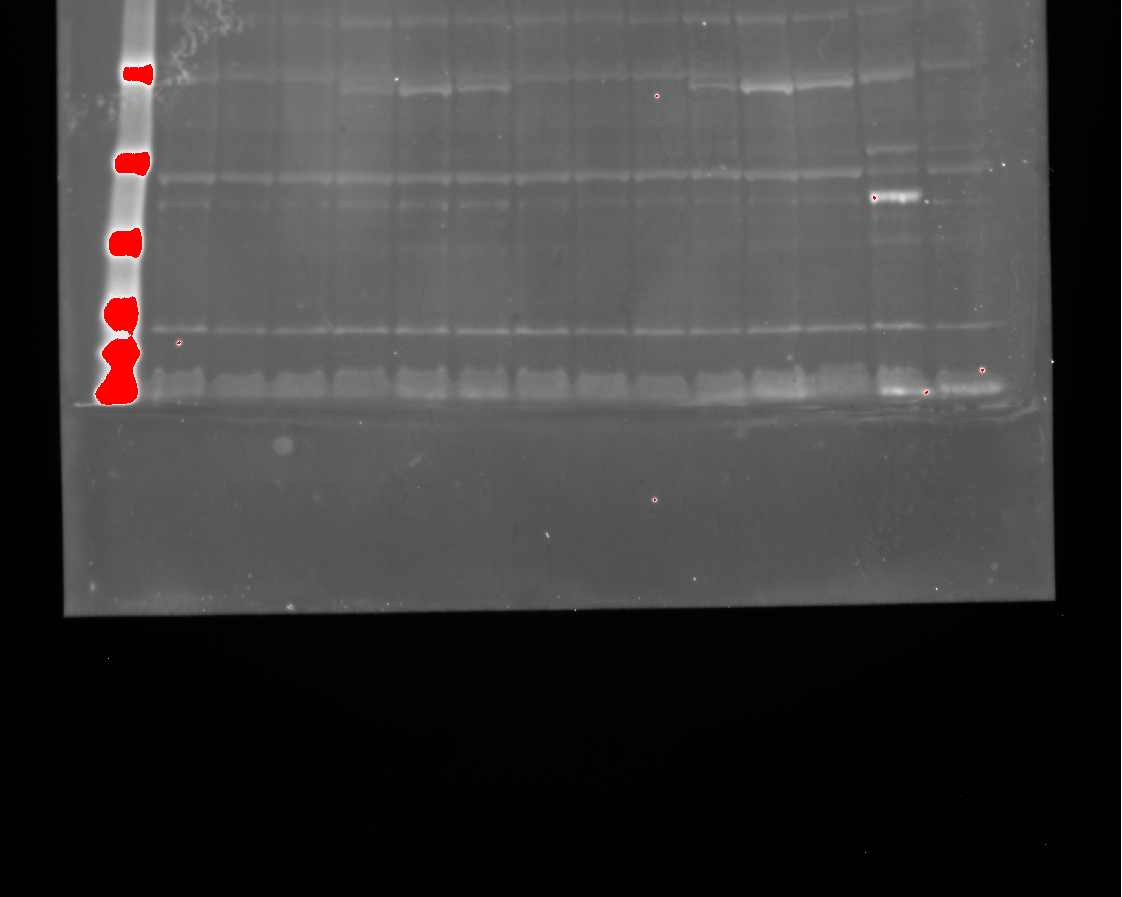

Supplement: S1 Data — (ZIP) [file pgen.1011059.s014.zip › SIdata/Figure 4 + S3/4BC+S3BC_WB RssB/Set 4/lmbchemidoc 2021-01-29 17h41m54s(StarBright B700).tif]

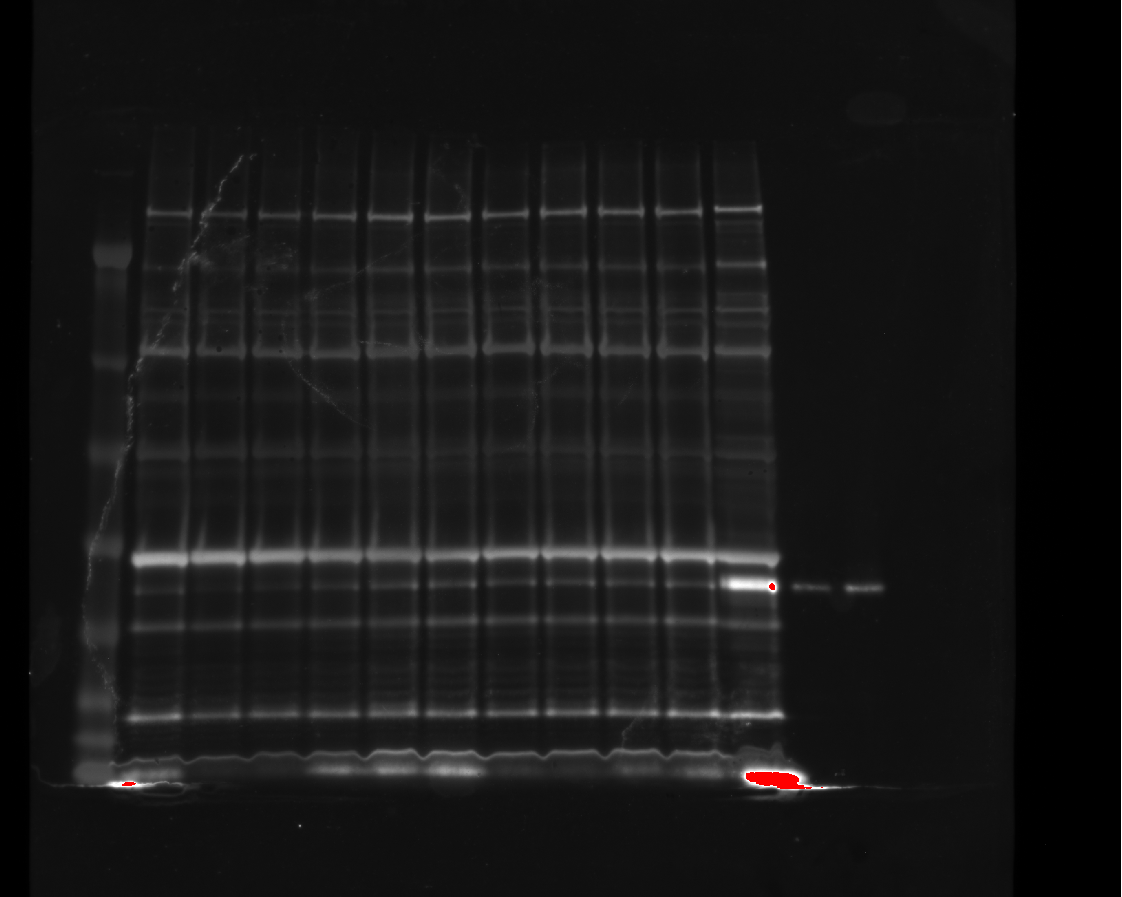

Supplement: S1 Data — (ZIP) [file pgen.1011059.s014.zip › SIdata/Figure 4 + S3/4BC+S3BC_WB RssB/Set 1/15(StarBright B700).tif]

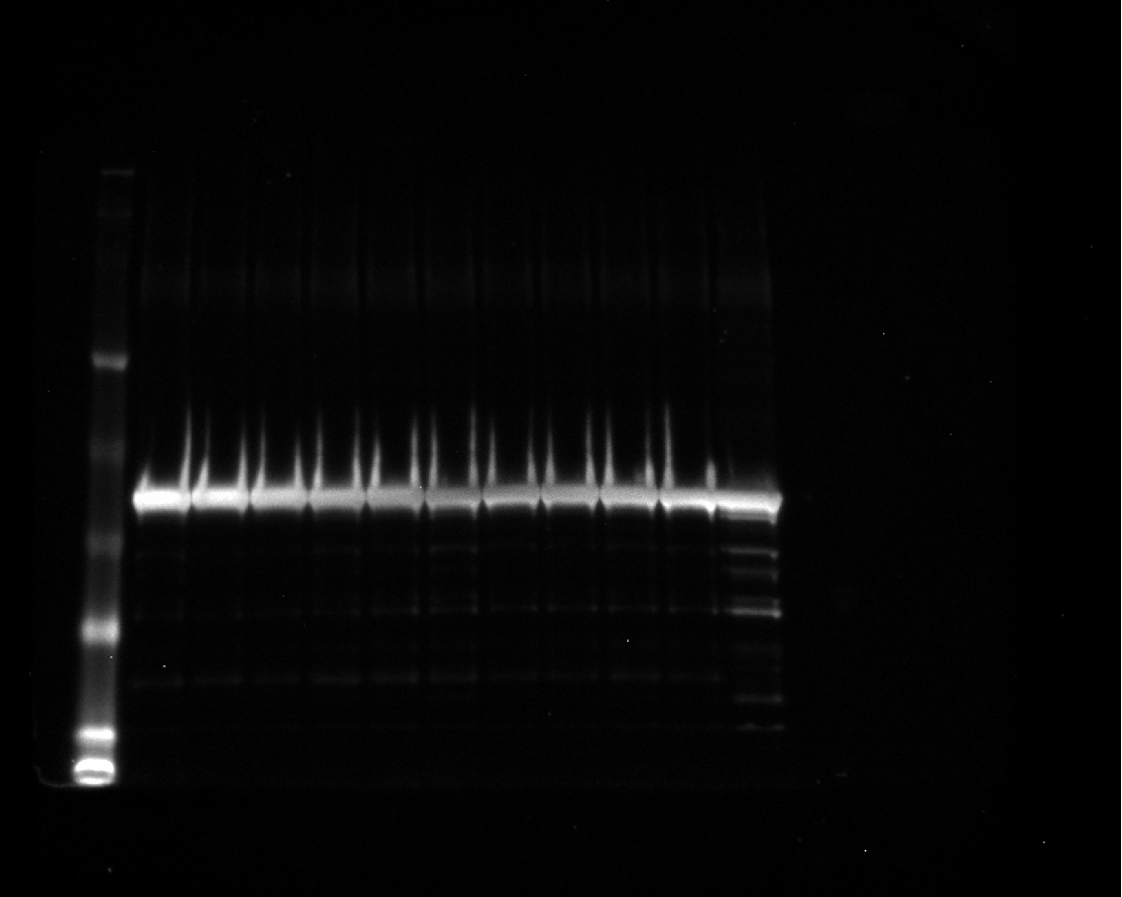

Supplement: S1 Data — (ZIP) [file pgen.1011059.s014.zip › SIdata/Figure 4 + S3/4BC+S3BC_WB RssB/Set 1/5(DyLight 800).tif]

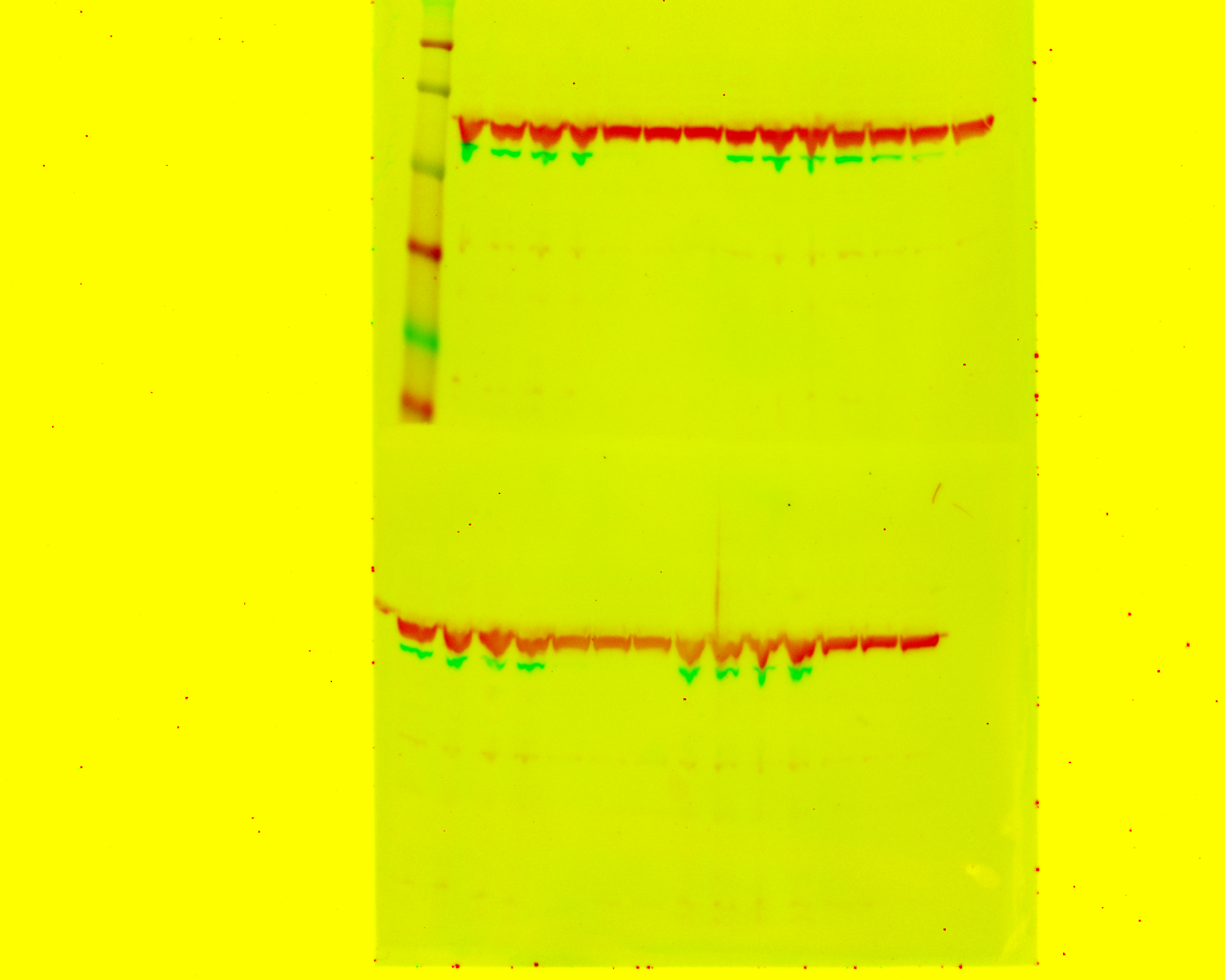

Supplement: S1 Data — (ZIP) [file pgen.1011059.s014.zip › SIdata/Figure 2 + S1/2E+2F+S1D_WB Stat phase+recovery RpoS/Set 2/lmbchemidoc 2023-12-23 13h58m46s(Composite).tif]

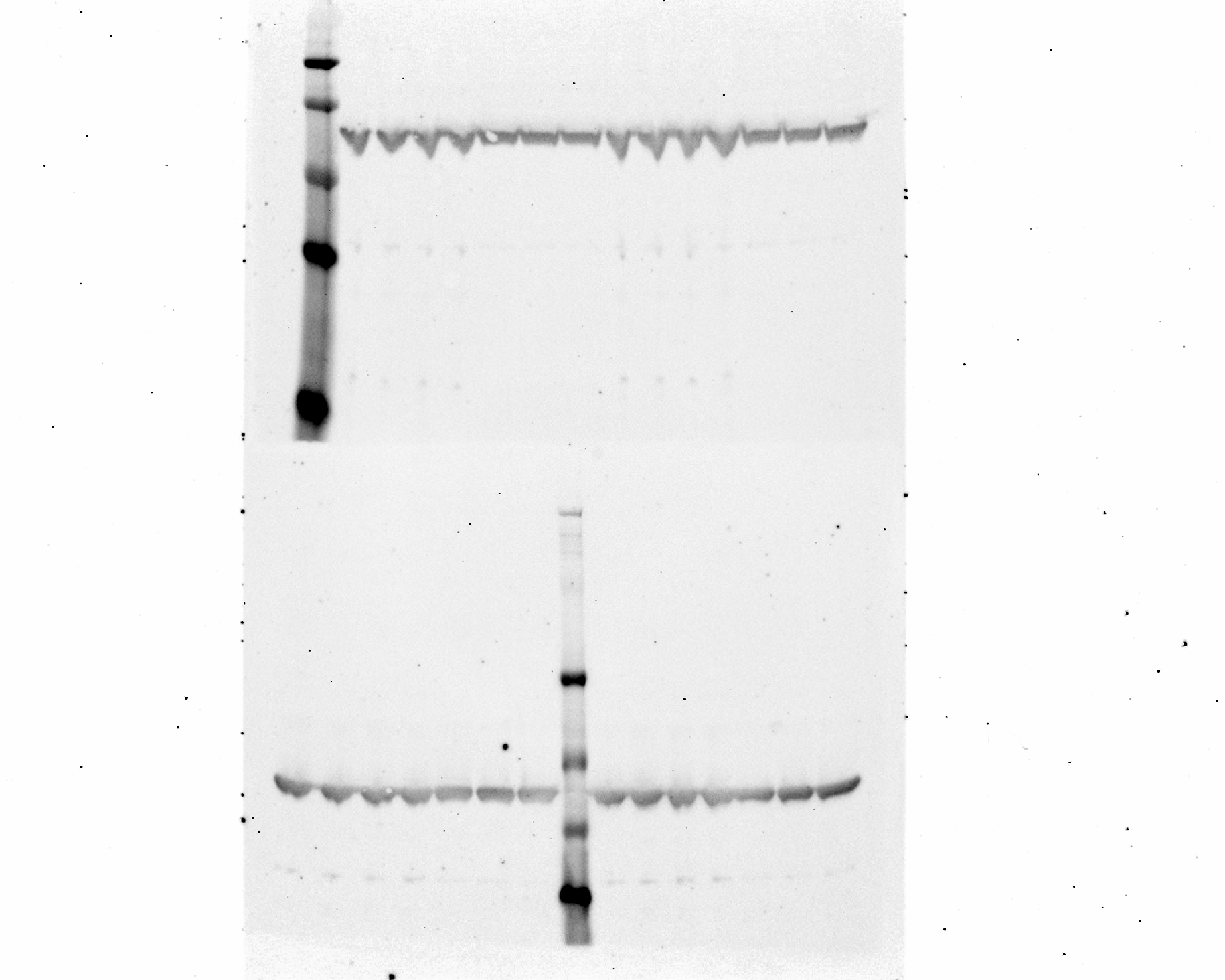

Supplement: S1 Data — (ZIP) [file pgen.1011059.s014.zip › SIdata/Figure 2 + S1/2E+2F+S1D_WB Stat phase+recovery RpoS/Set 2/lmbchemidoc 2023-12-23 13h56m15s(DyLight 800).tif]

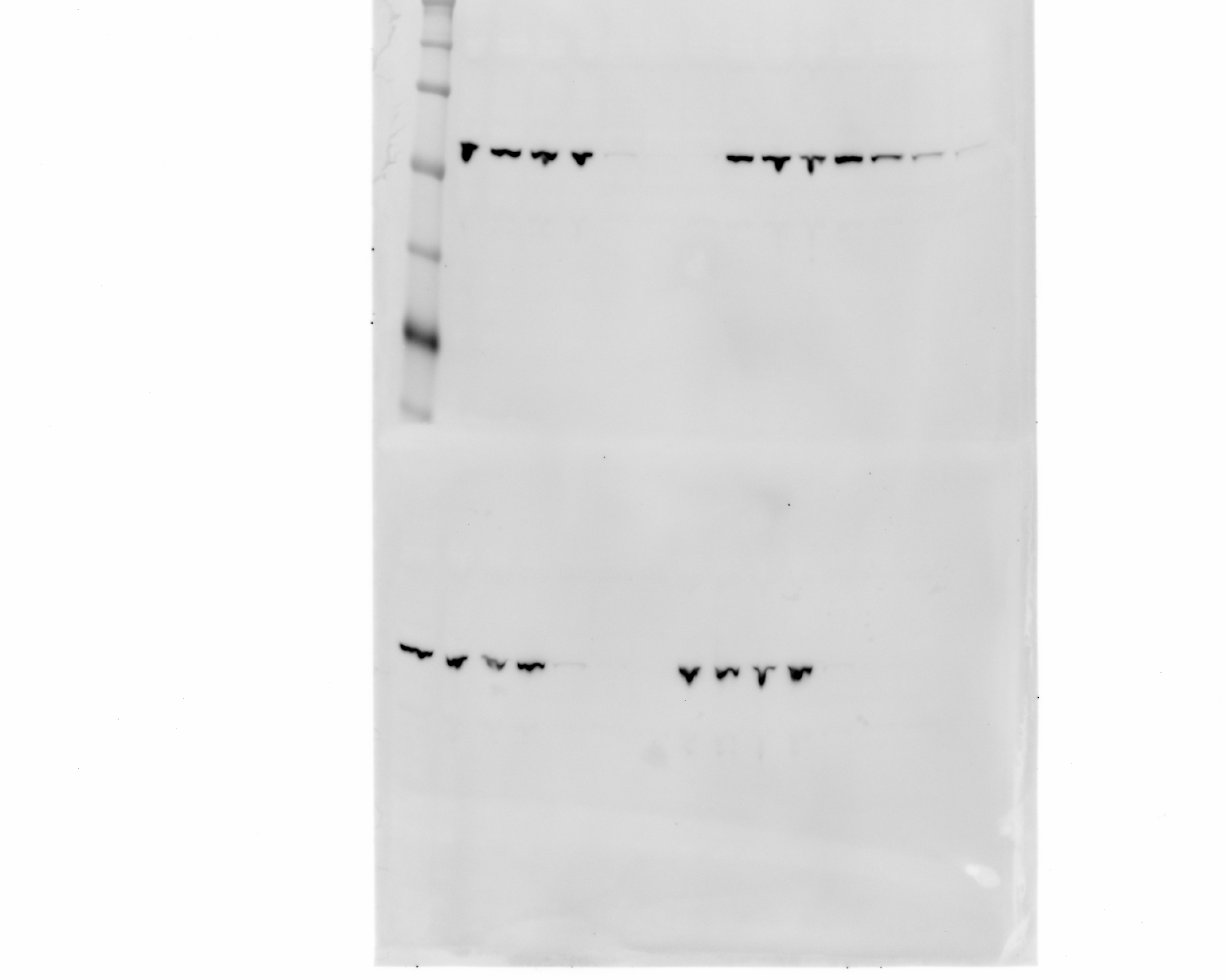

Supplement: S1 Data — (ZIP) [file pgen.1011059.s014.zip › SIdata/Figure 2 + S1/2E+2F+S1D_WB Stat phase+recovery RpoS/Set 2/lmbchemidoc 2023-12-23 13h58m46s(StarBright B700).tif]

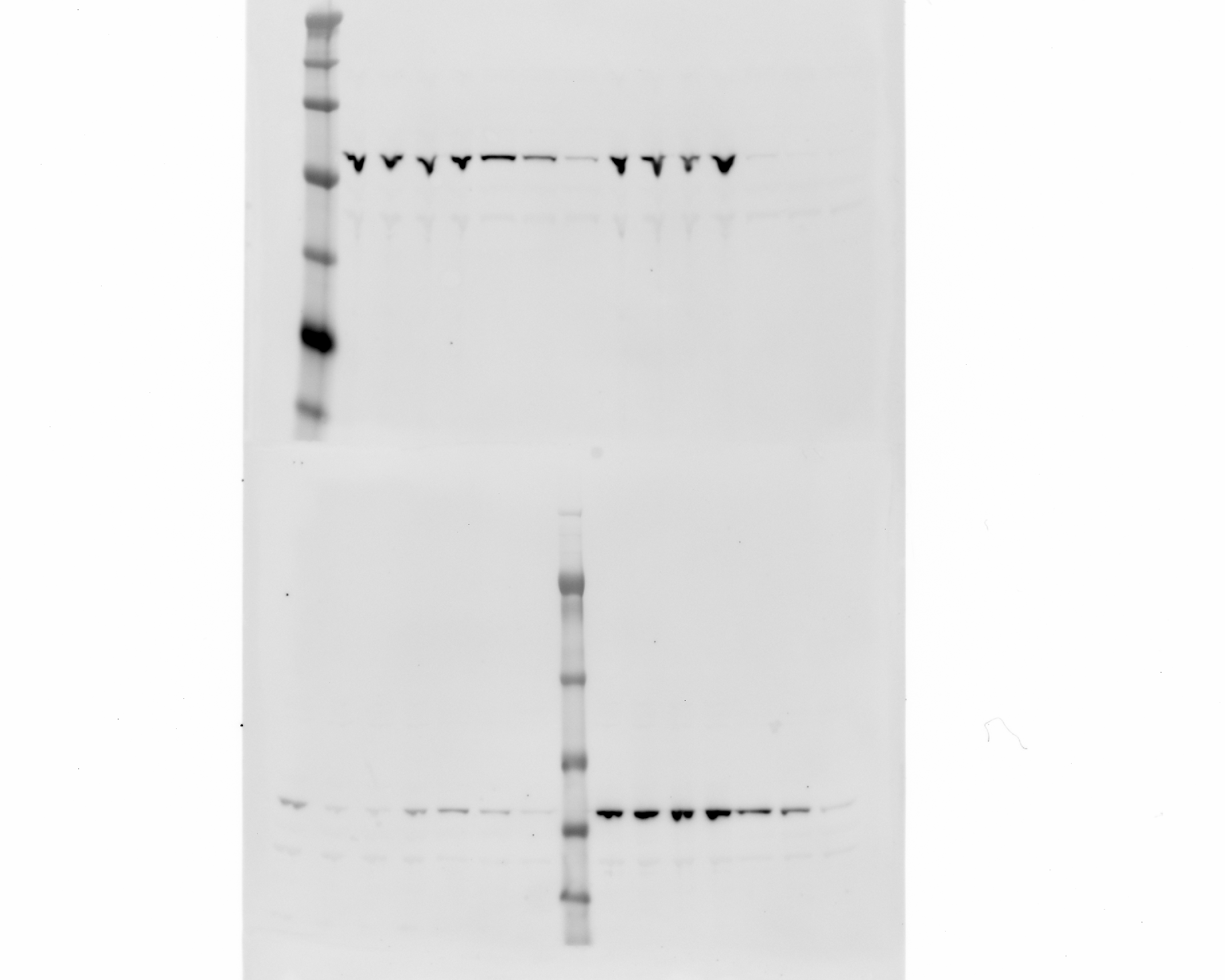

Supplement: S1 Data — (ZIP) [file pgen.1011059.s014.zip › SIdata/Figure 2 + S1/2E+2F+S1D_WB Stat phase+recovery RpoS/Set 2/lmbchemidoc 2023-12-23 13h56m15s(StarBright B700).tif]

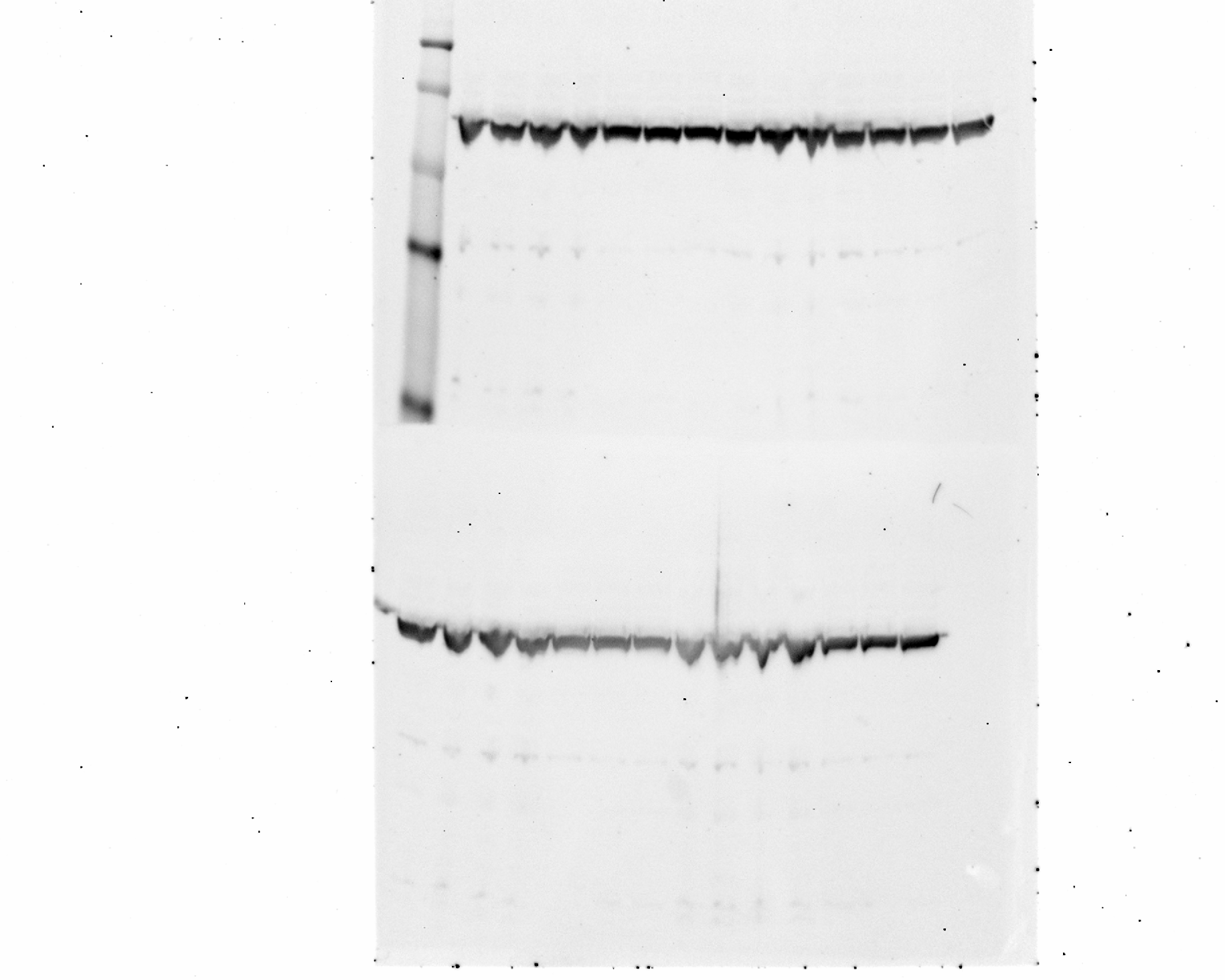

Supplement: S1 Data — (ZIP) [file pgen.1011059.s014.zip › SIdata/Figure 2 + S1/2E+2F+S1D_WB Stat phase+recovery RpoS/Set 2/lmbchemidoc 2023-12-23 13h58m46s(DyLight 800).tif]

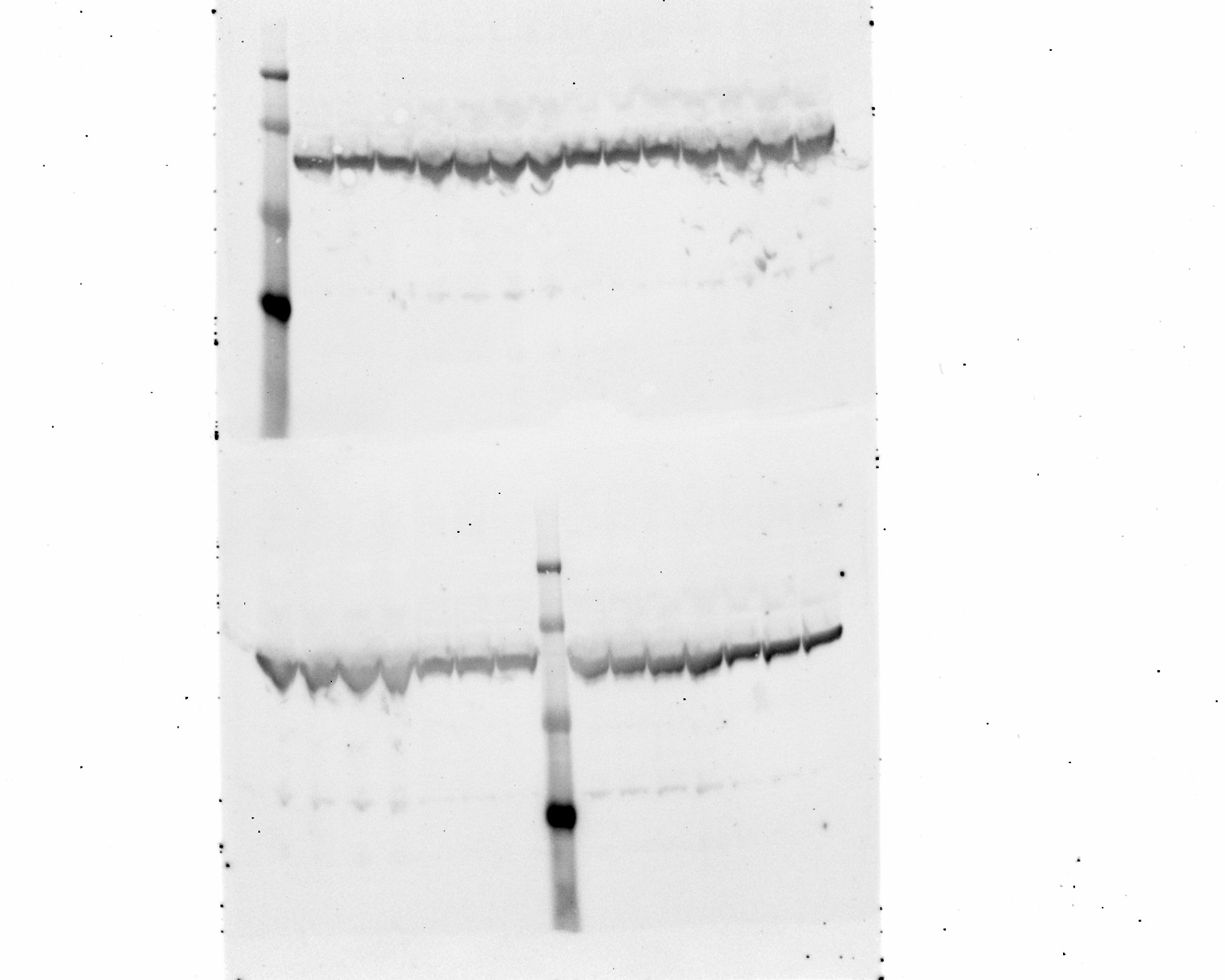

Supplement: S1 Data — (ZIP) [file pgen.1011059.s014.zip › SIdata/Figure 2 + S1/2E+2F+S1D_WB Stat phase+recovery RpoS/Set 1/lmbchemidoc 2023-12-22 17h44m00s(DyLight 800).tif]

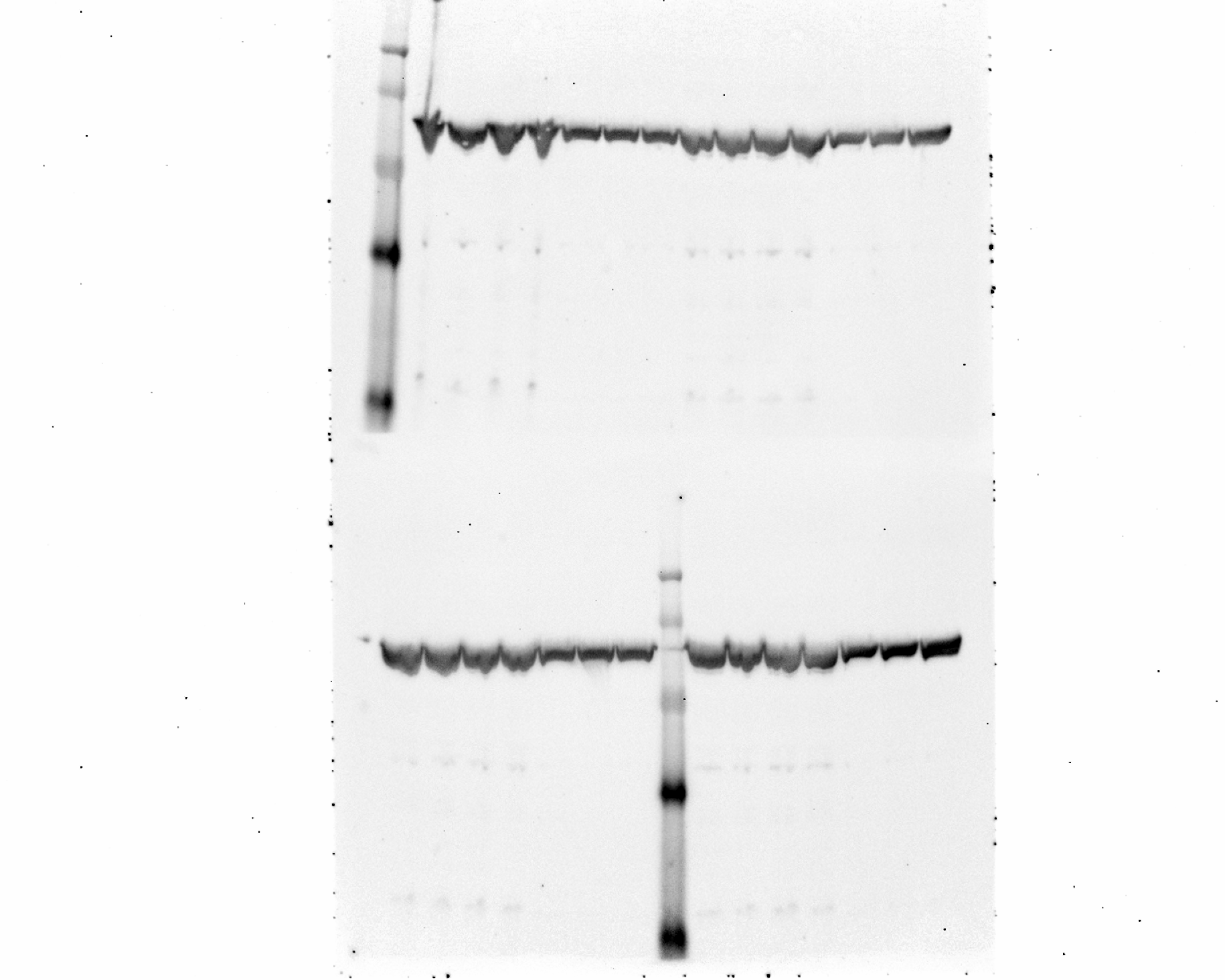

Supplement: S1 Data — (ZIP) [file pgen.1011059.s014.zip › SIdata/Figure 2 + S1/2E+2F+S1D_WB Stat phase+recovery RpoS/Set 1/lmbchemidoc 2023-12-22 17h41m53s(DyLight 800).tif]

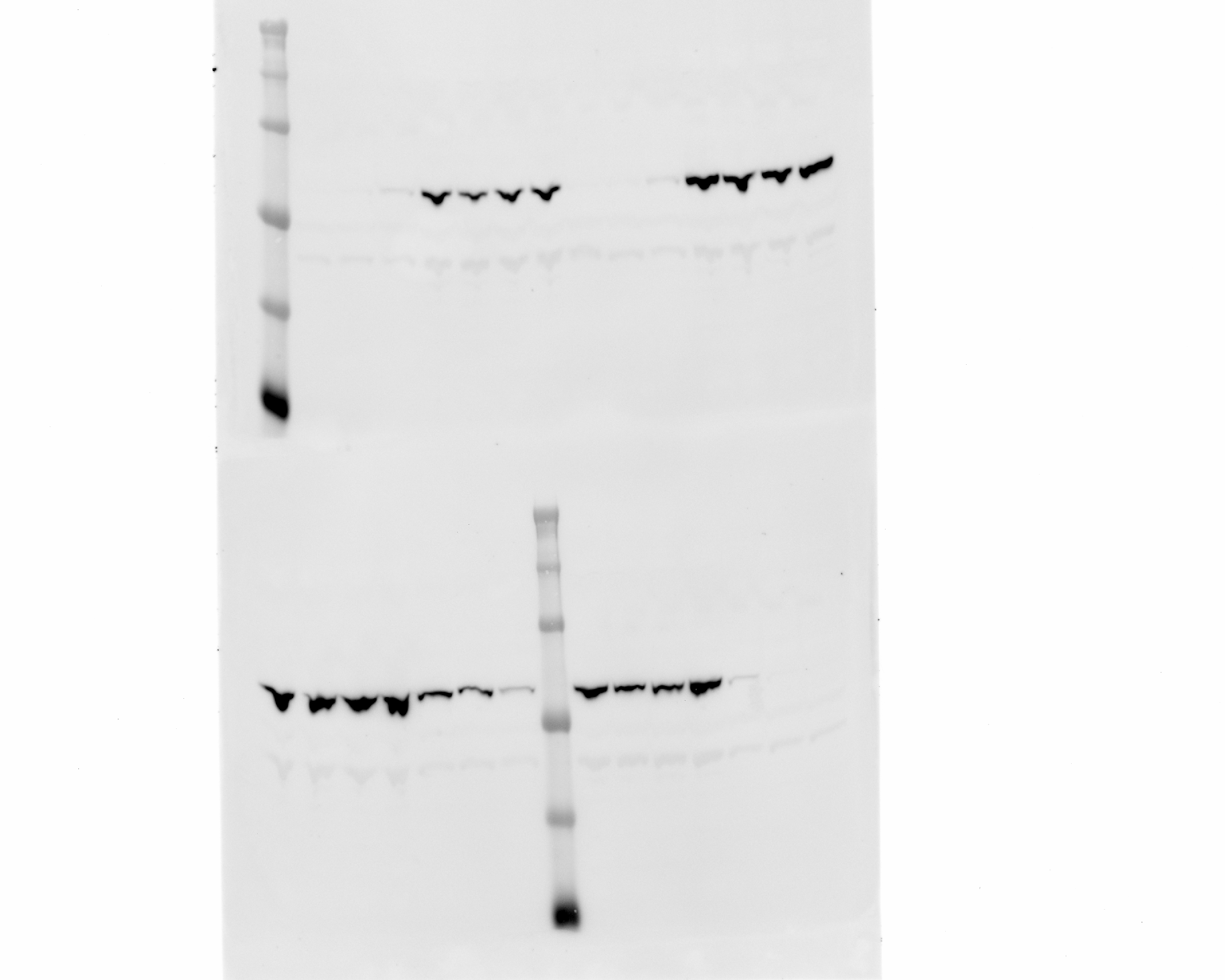

Supplement: S1 Data — (ZIP) [file pgen.1011059.s014.zip › SIdata/Figure 2 + S1/2E+2F+S1D_WB Stat phase+recovery RpoS/Set 1/lmbchemidoc 2023-12-22 17h44m00s(StarBright B700).tif]

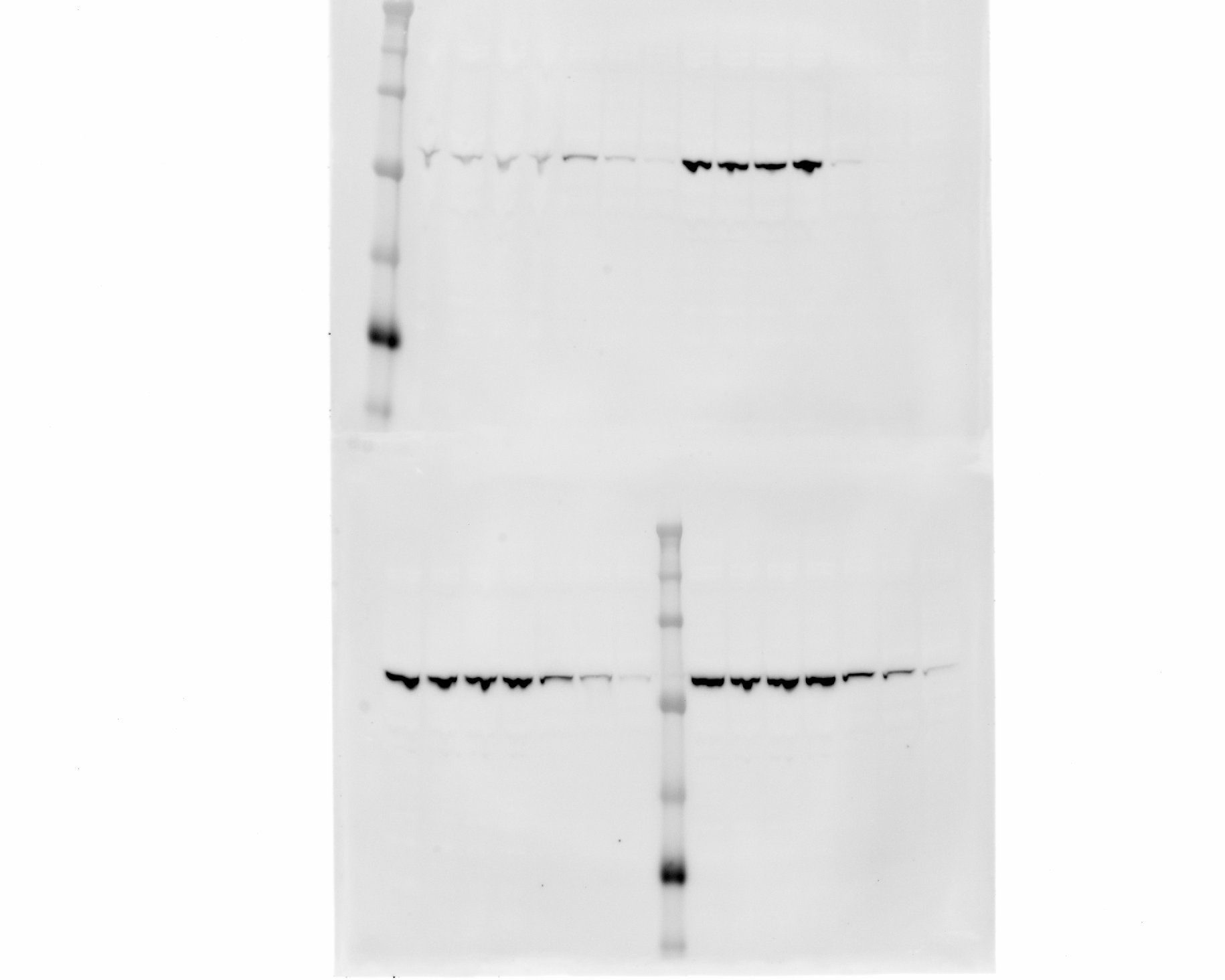

Supplement: S1 Data — (ZIP) [file pgen.1011059.s014.zip › SIdata/Figure 2 + S1/2E+2F+S1D_WB Stat phase+recovery RpoS/Set 1/lmbchemidoc 2023-12-22 17h41m53s(StarBright B700).tif]

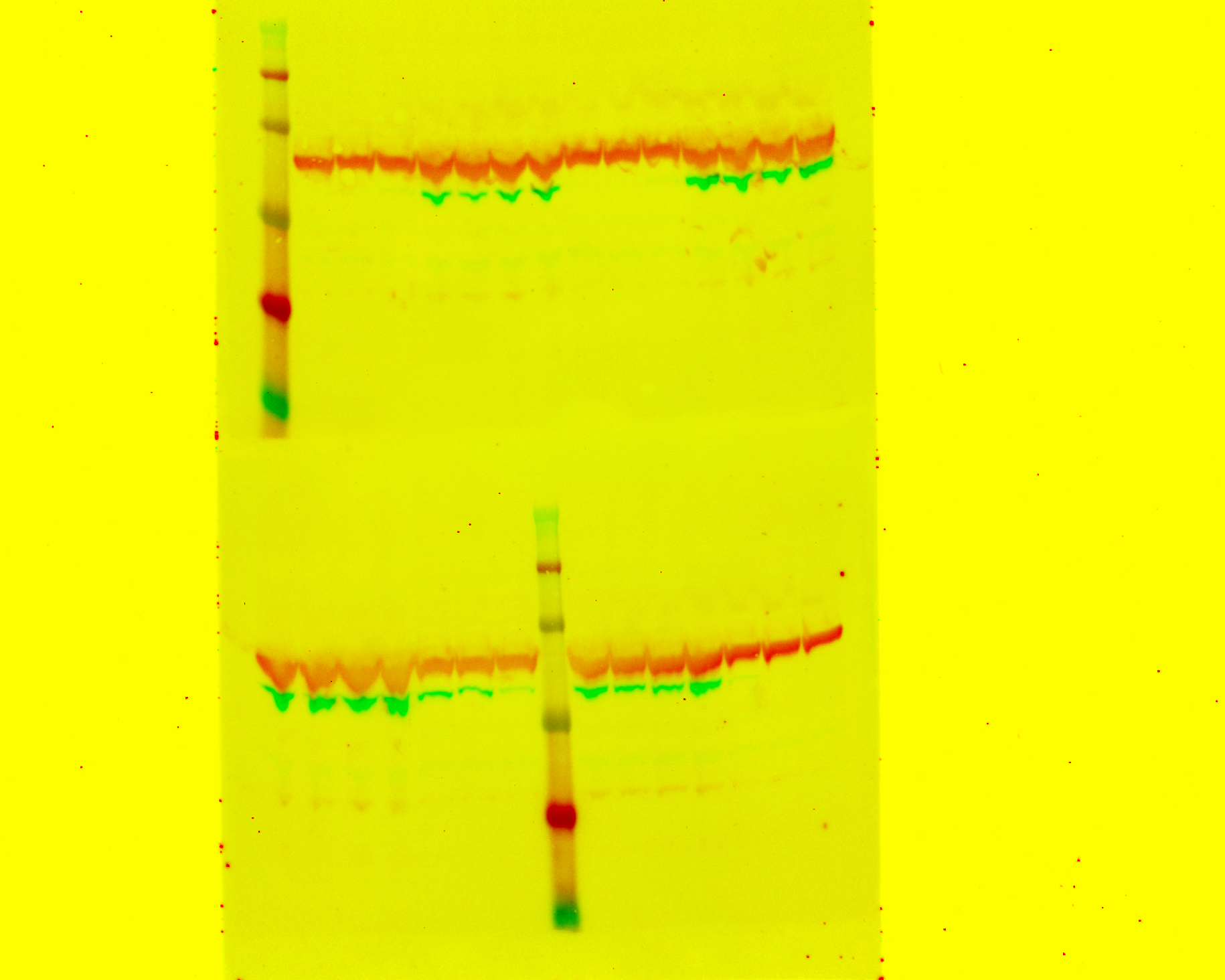

Supplement: S1 Data — (ZIP) [file pgen.1011059.s014.zip › SIdata/Figure 2 + S1/2E+2F+S1D_WB Stat phase+recovery RpoS/Set 1/lmbchemidoc 2023-12-22 17h44m00s(Composite).tif]

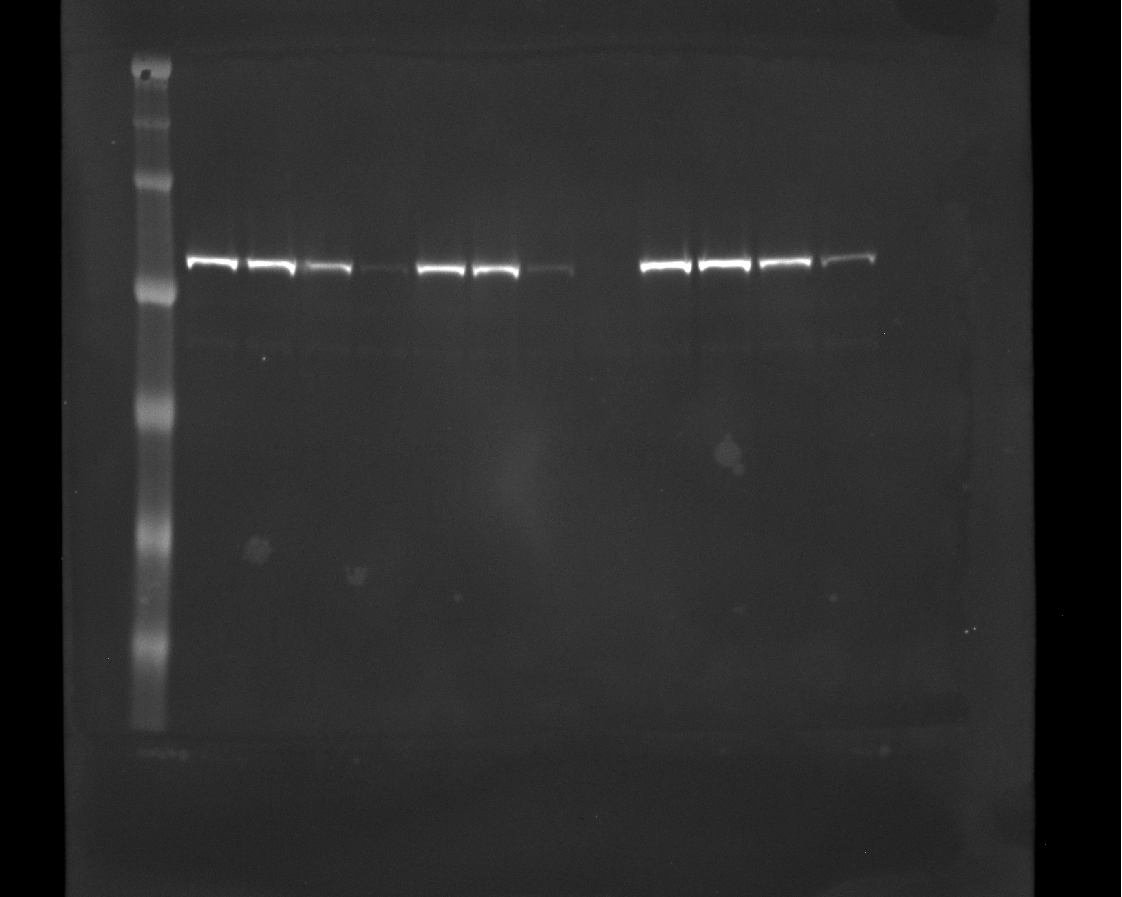

Supplement: S1 Data — (ZIP) [file pgen.1011059.s014.zip › SIdata/Figure 2 + S1/2S+S1C_WB Glucose starvation+recovery RpoS/2021-02-22/4sec(StarBright B700).tif]

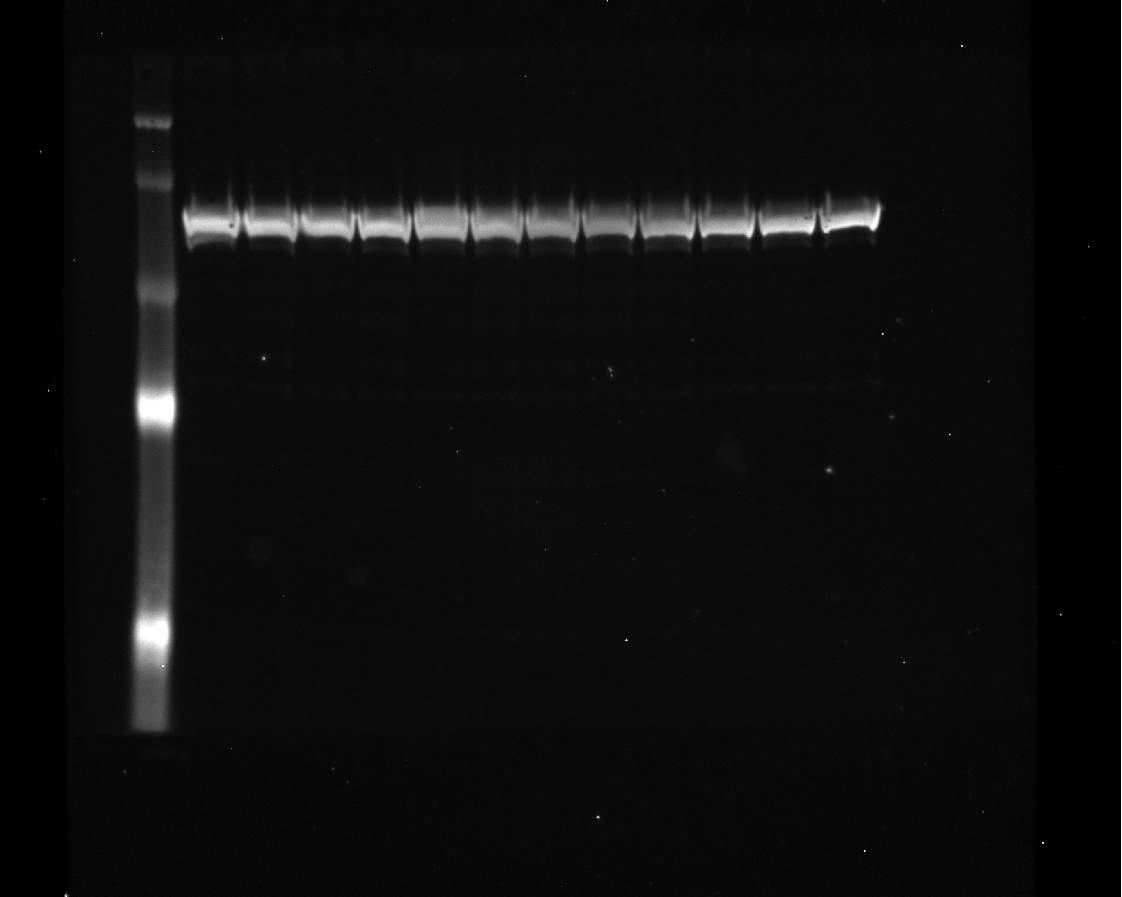

Supplement: S1 Data — (ZIP) [file pgen.1011059.s014.zip › SIdata/Figure 2 + S1/2S+S1C_WB Glucose starvation+recovery RpoS/2021-02-22/lmbchemidoc 2021-02-22 16h45m46s(DyLight 800).tif]

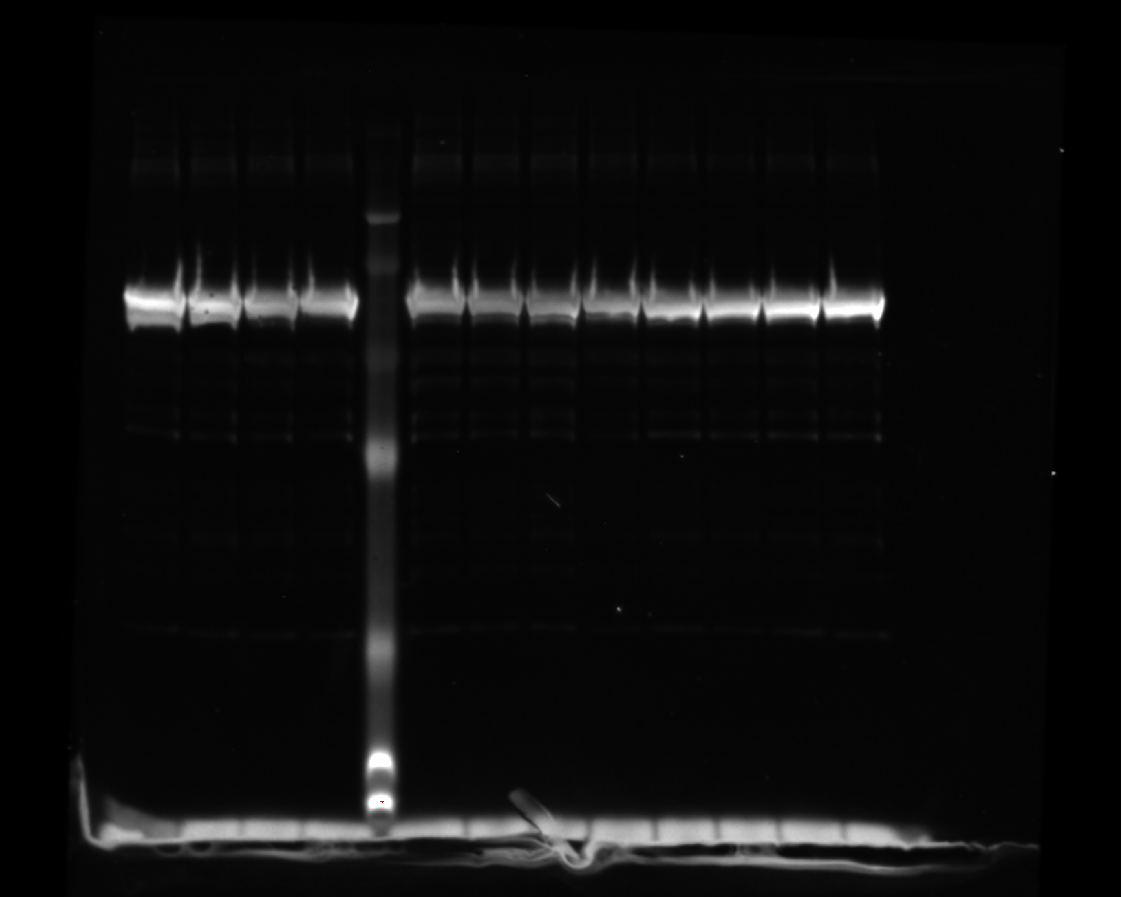

Supplement: S1 Data — (ZIP) [file pgen.1011059.s014.zip › SIdata/Figure 2 + S1/2S+S1C_WB Glucose starvation+recovery RpoS/2021-03-25/lmbchemidoc 2021-03-25 16h06m26s(DyLight 800).tif]

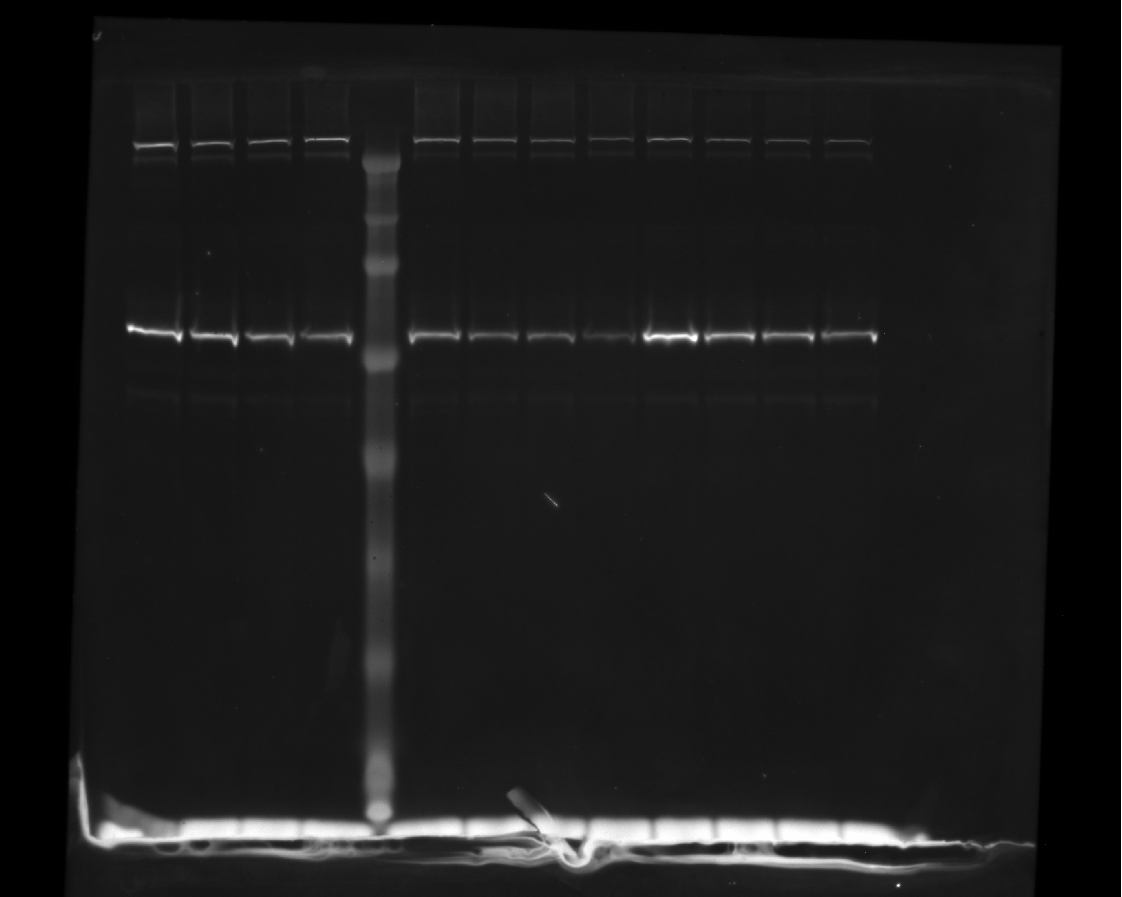

Supplement: S1 Data — (ZIP) [file pgen.1011059.s014.zip › SIdata/Figure 2 + S1/2S+S1C_WB Glucose starvation+recovery RpoS/2021-03-25/lmbchemidoc 2021-03-25 16h08m05s(StarBright B700).tif]

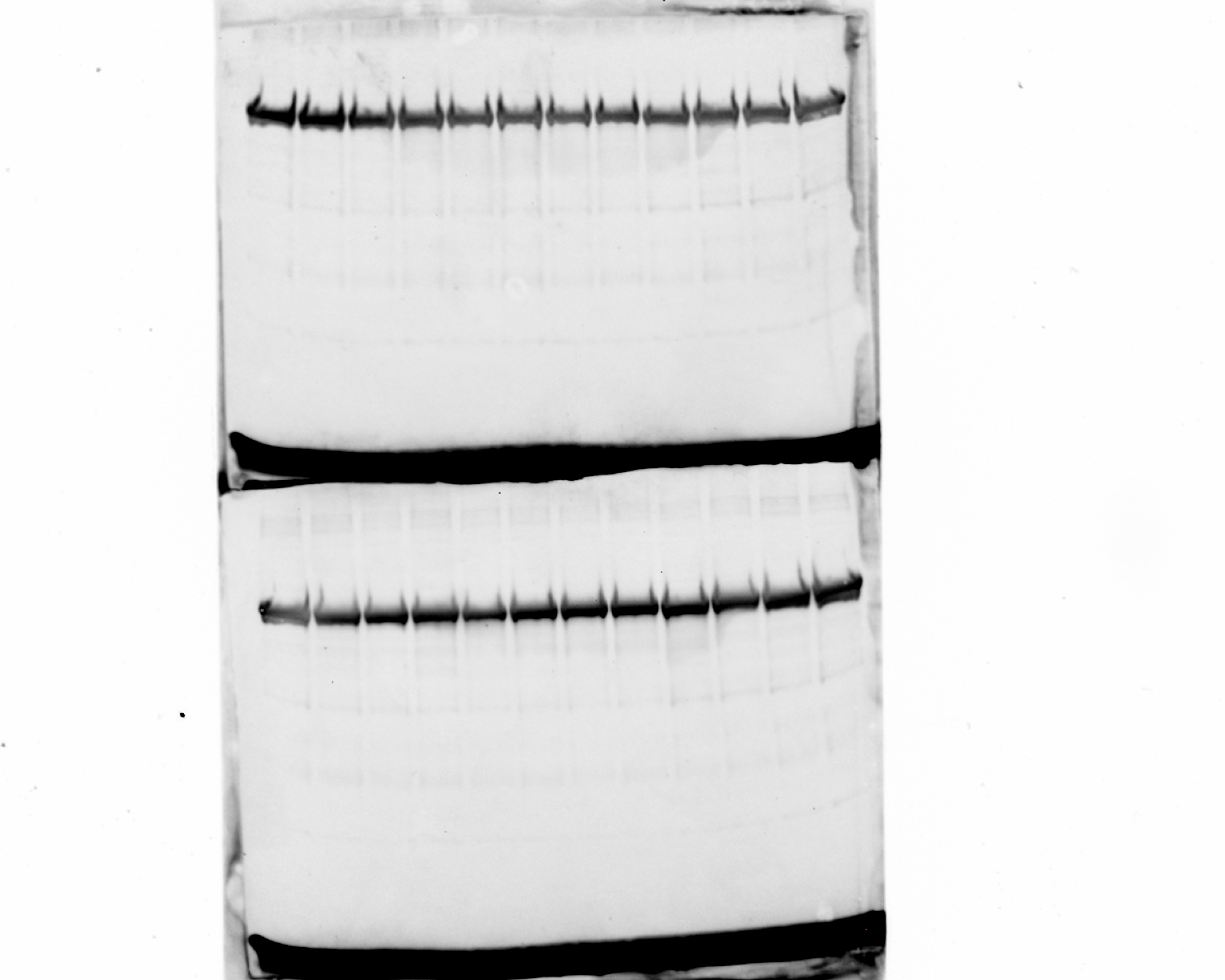

Supplement: S1 Data — (ZIP) [file pgen.1011059.s014.zip › SIdata/Figure 2 + S1/2S+S1C_WB Glucose starvation+recovery RpoS/2022-01-11/lmbchemidoc 2022-01-10 18h10m08s(DyLight 800).jpg]

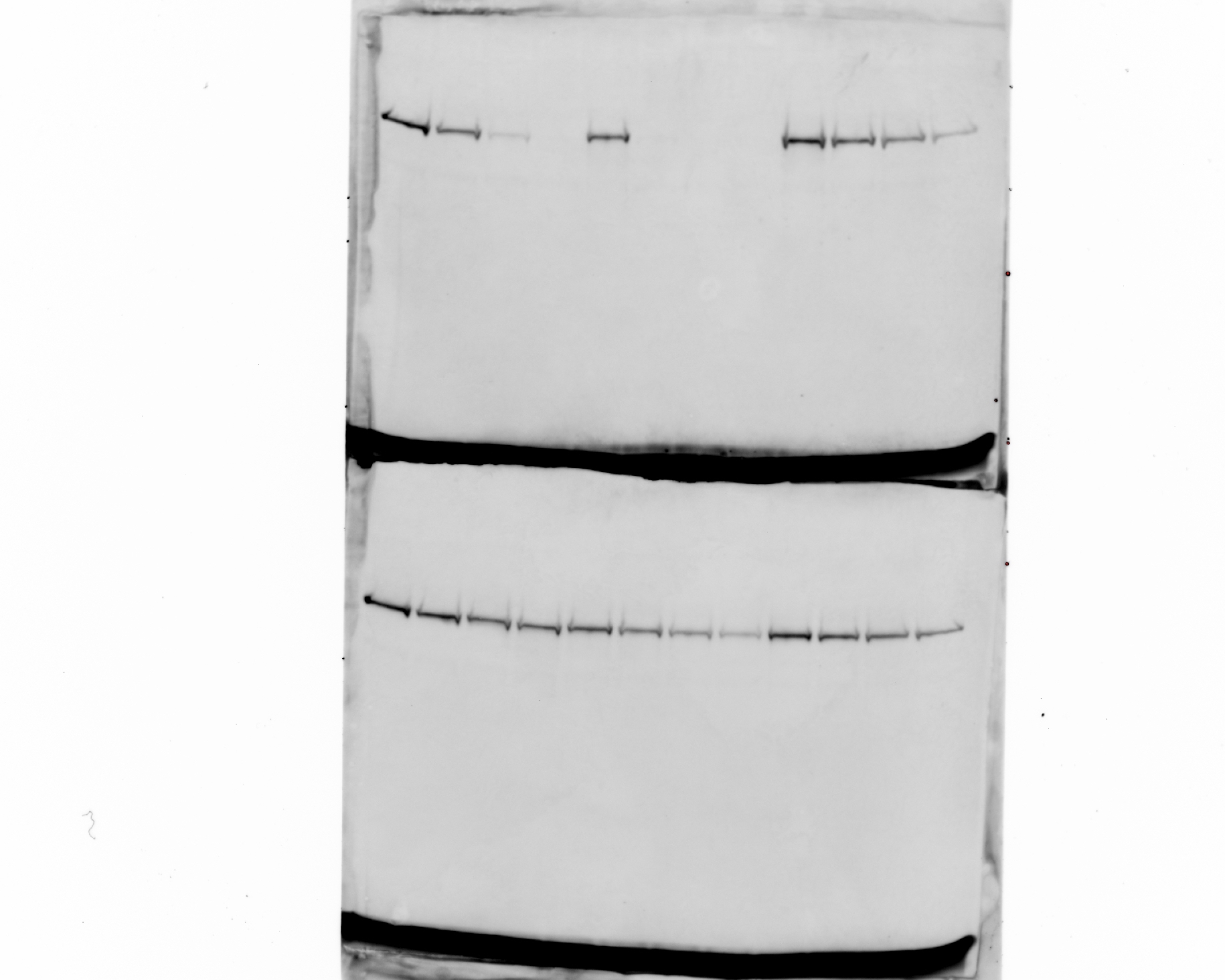

Supplement: S1 Data — (ZIP) [file pgen.1011059.s014.zip › SIdata/Figure 2 + S1/2S+S1C_WB Glucose starvation+recovery RpoS/2022-01-11/lmbchemidoc 2022-01-10 18h10m08s(StarBright B700).jpg]

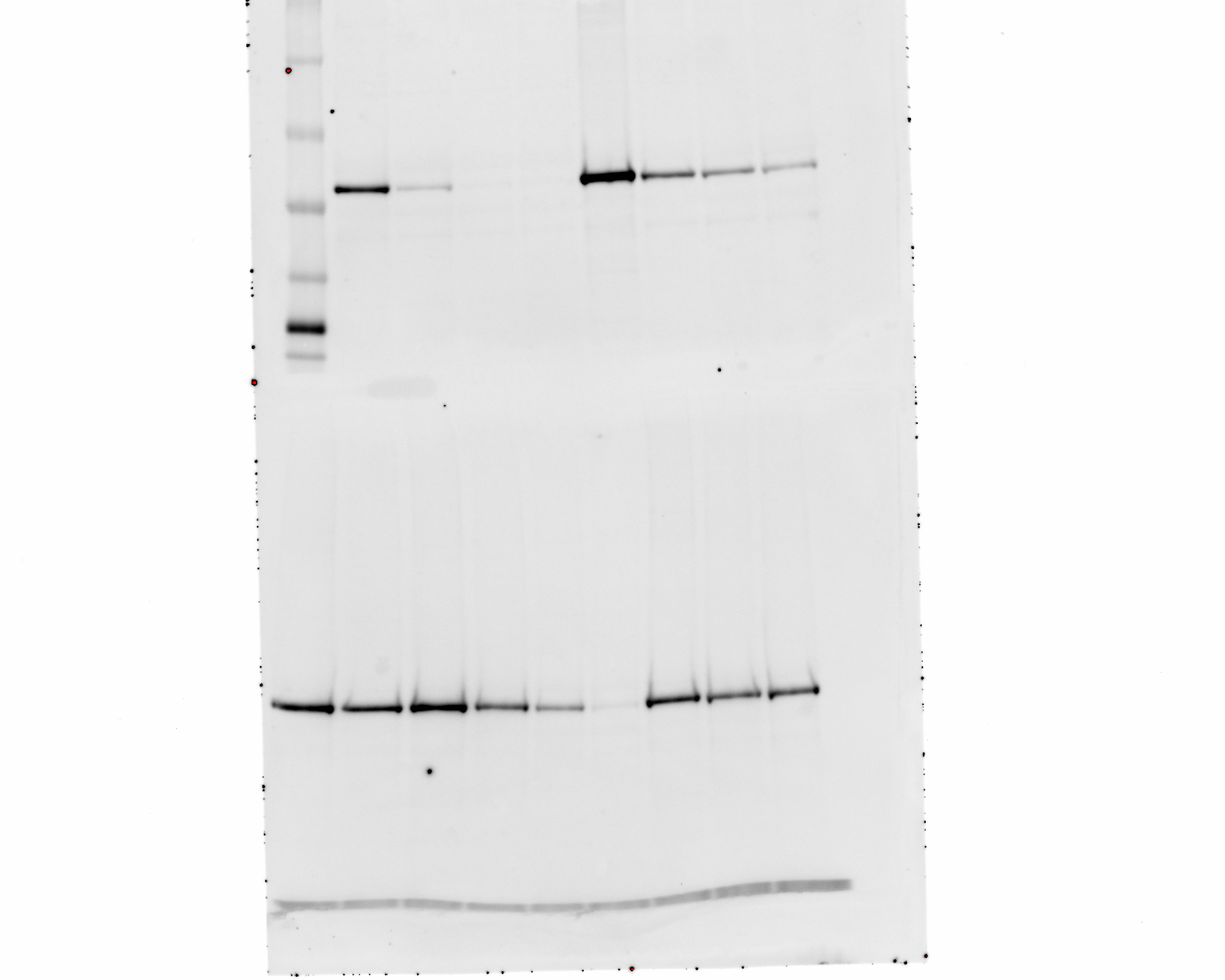

Supplement: S1 Data — (ZIP) [file pgen.1011059.s014.zip › SIdata/Figure 2 + S1/2S+S1C_WB Glucose starvation+recovery RpoS/2021-12-28/lmbchemidoc 2021-12-28 17h23m57s(StarBright B700).tif]

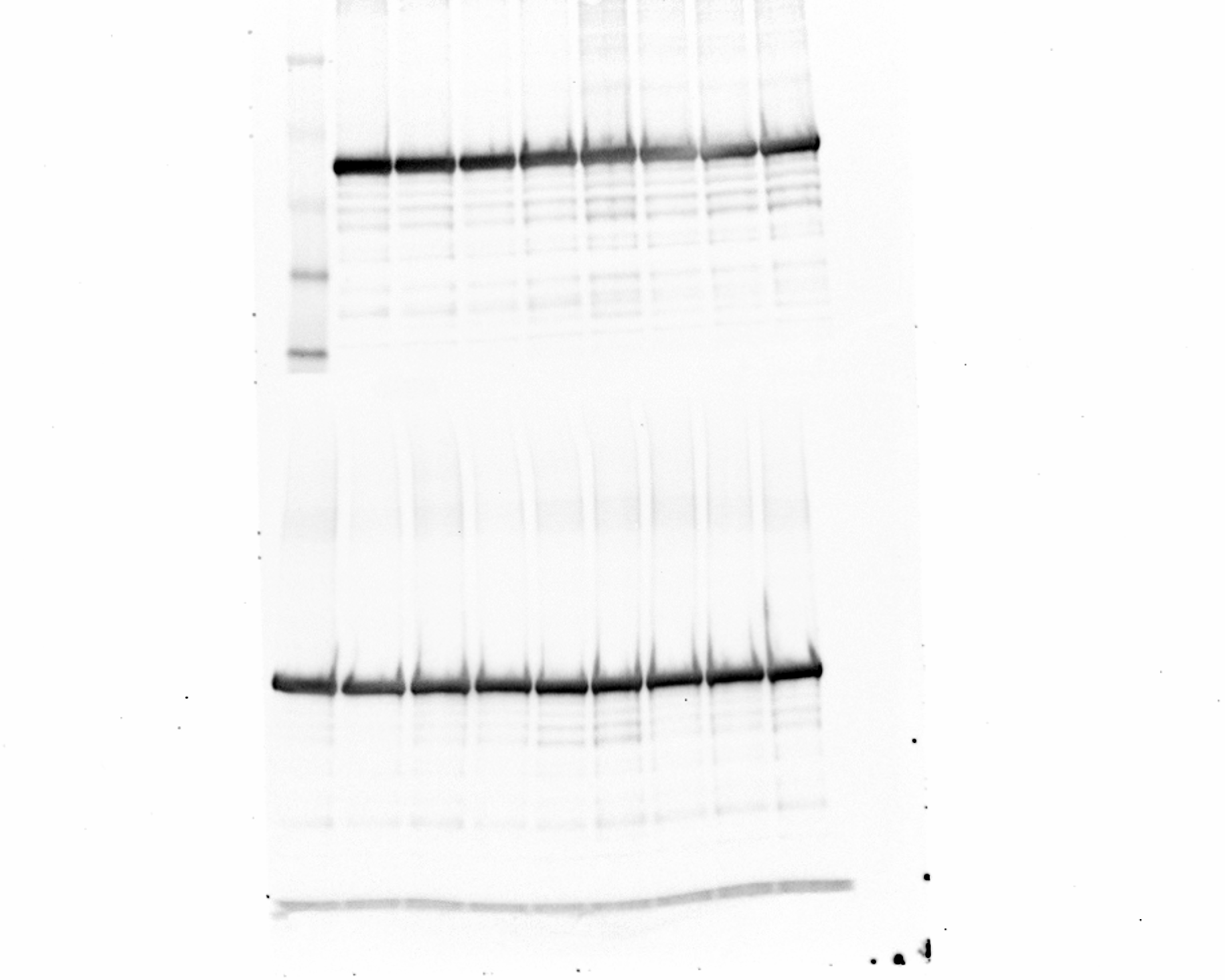

Supplement: S1 Data — (ZIP) [file pgen.1011059.s014.zip › SIdata/Figure 2 + S1/2S+S1C_WB Glucose starvation+recovery RpoS/2021-12-28/lmbchemidoc 2021-12-28 17h23m57s(DyLight 800).tif]

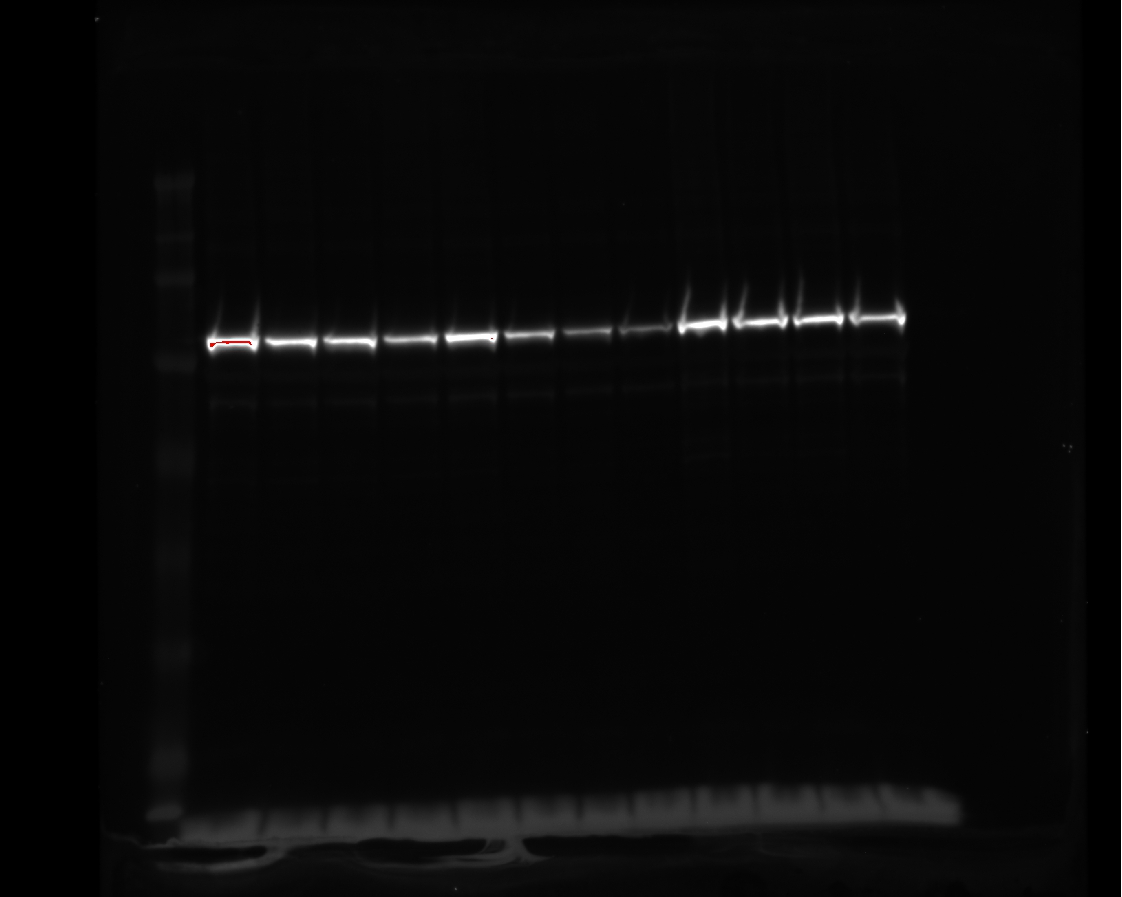

Supplement: S1 Data — (ZIP) [file pgen.1011059.s014.zip › SIdata/Figure 2 + S1/2S+S1C_WB Glucose starvation+recovery RpoS/2021-03-19/lmbchemidoc 2021-03-19 18h40m30s(StarBright B700).jpg]

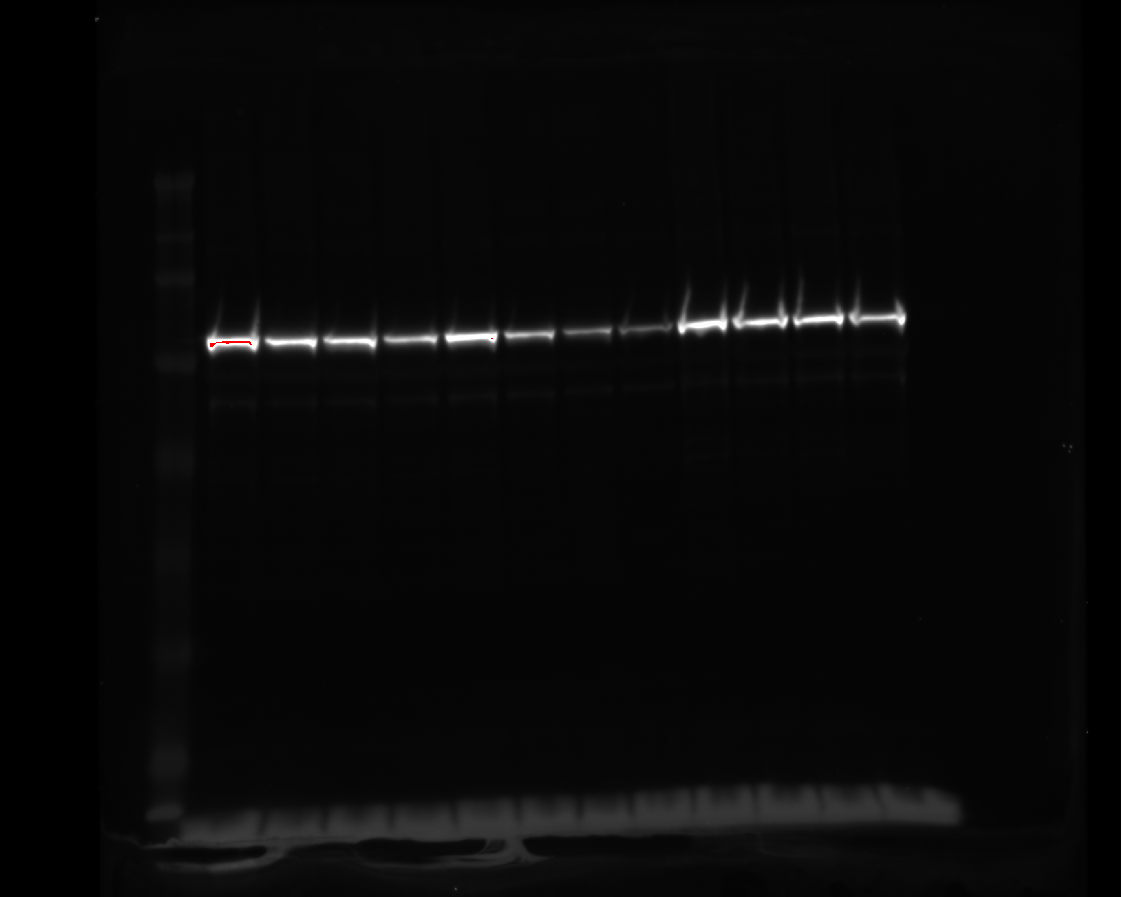

Supplement: S1 Data — (ZIP) [file pgen.1011059.s014.zip › SIdata/Figure 2 + S1/2S+S1C_WB Glucose starvation+recovery RpoS/2021-03-19/lmbchemidoc 2021-03-19 18h40m30s(StarBright B700).tif]

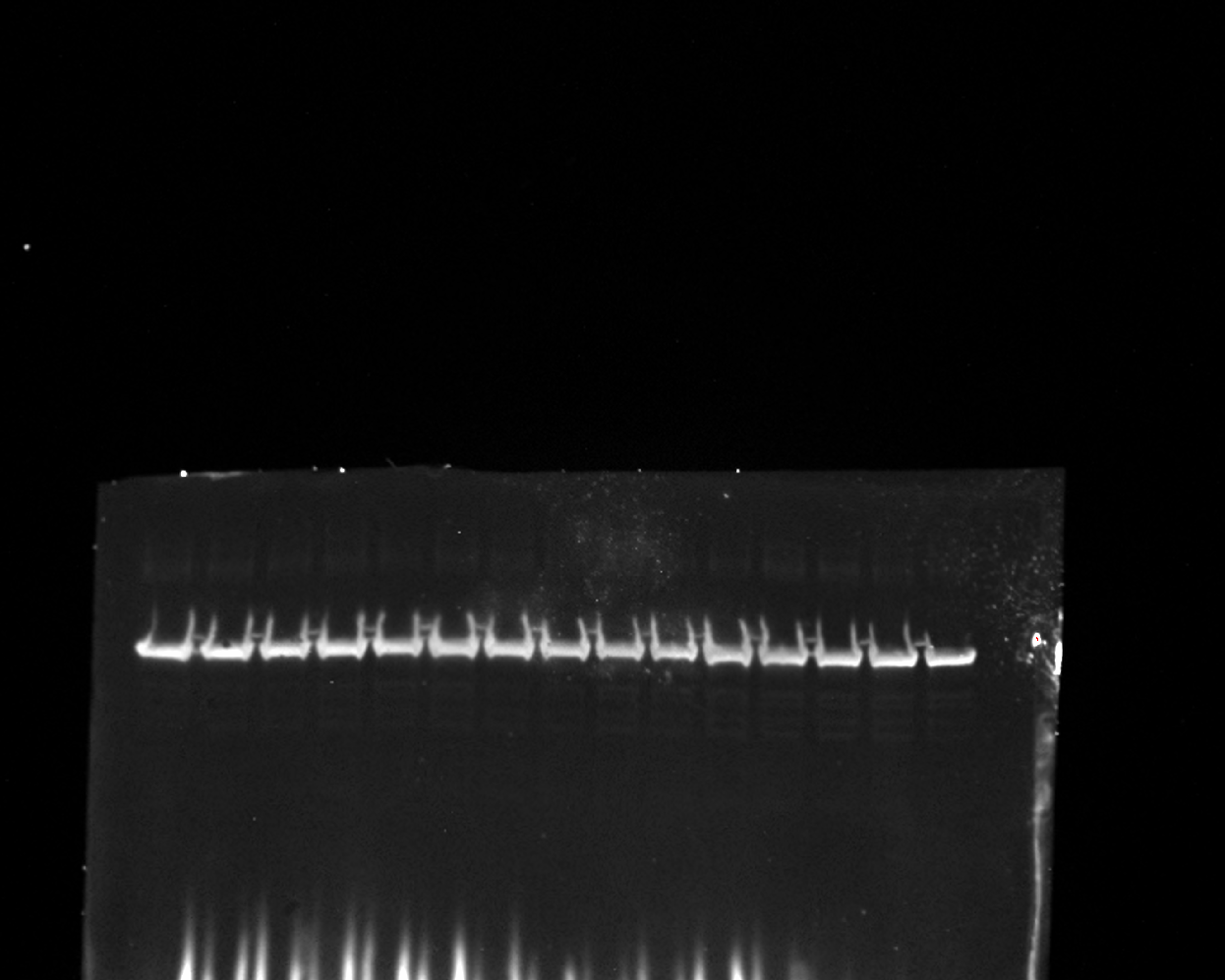

Supplement: S1 Data — (ZIP) [file pgen.1011059.s014.zip › SIdata/Figure 2 + S1/2S+S1C_WB Glucose starvation+recovery RpoS/2021-03-17/lmbchemidoc 2021-03-17 18h01m20s(DyLight 800).tif]

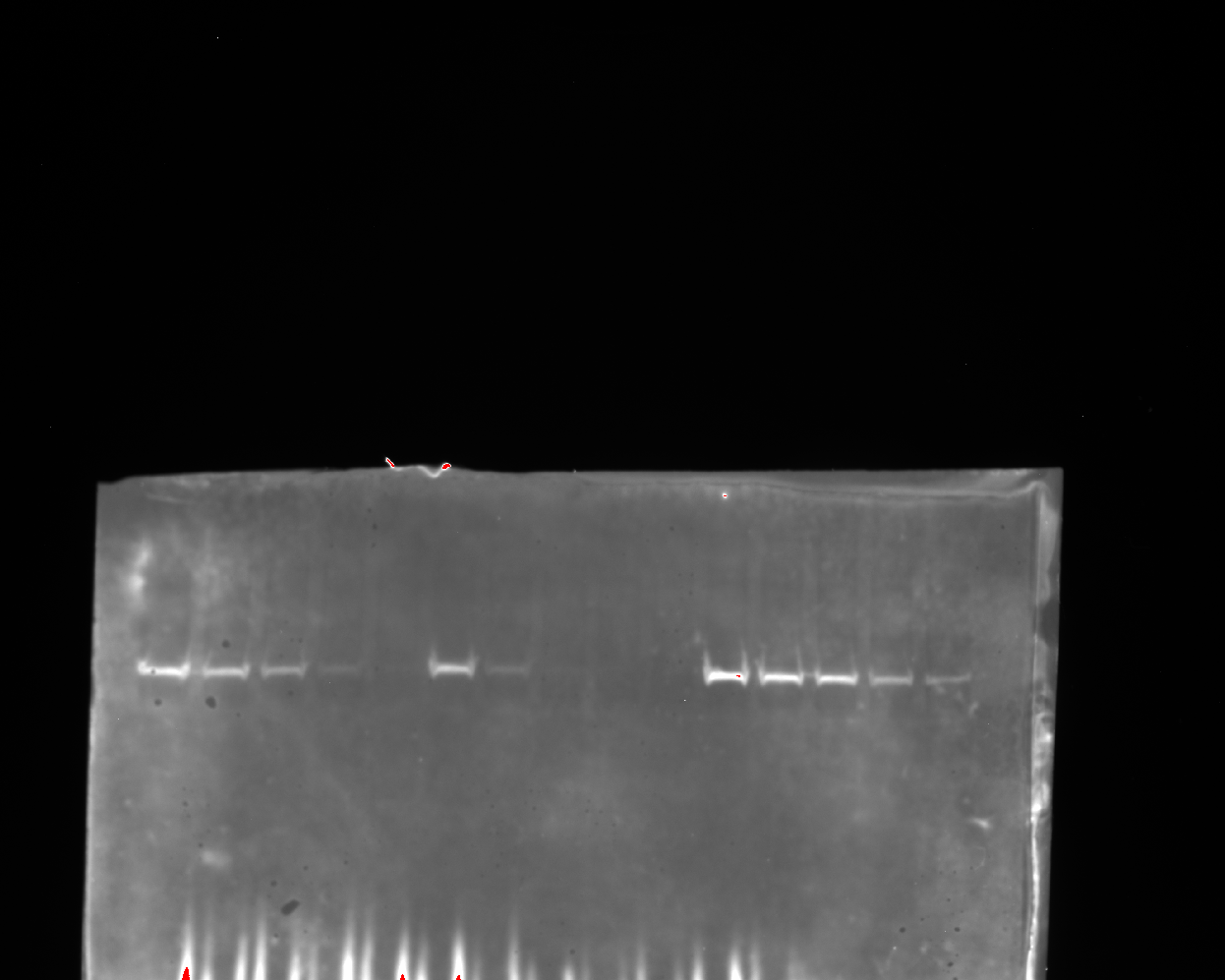

Supplement: S1 Data — (ZIP) [file pgen.1011059.s014.zip › SIdata/Figure 2 + S1/2S+S1C_WB Glucose starvation+recovery RpoS/2021-03-17/lmbchemidoc 2021-03-17 18h01m20s(StarBright B700).tif]

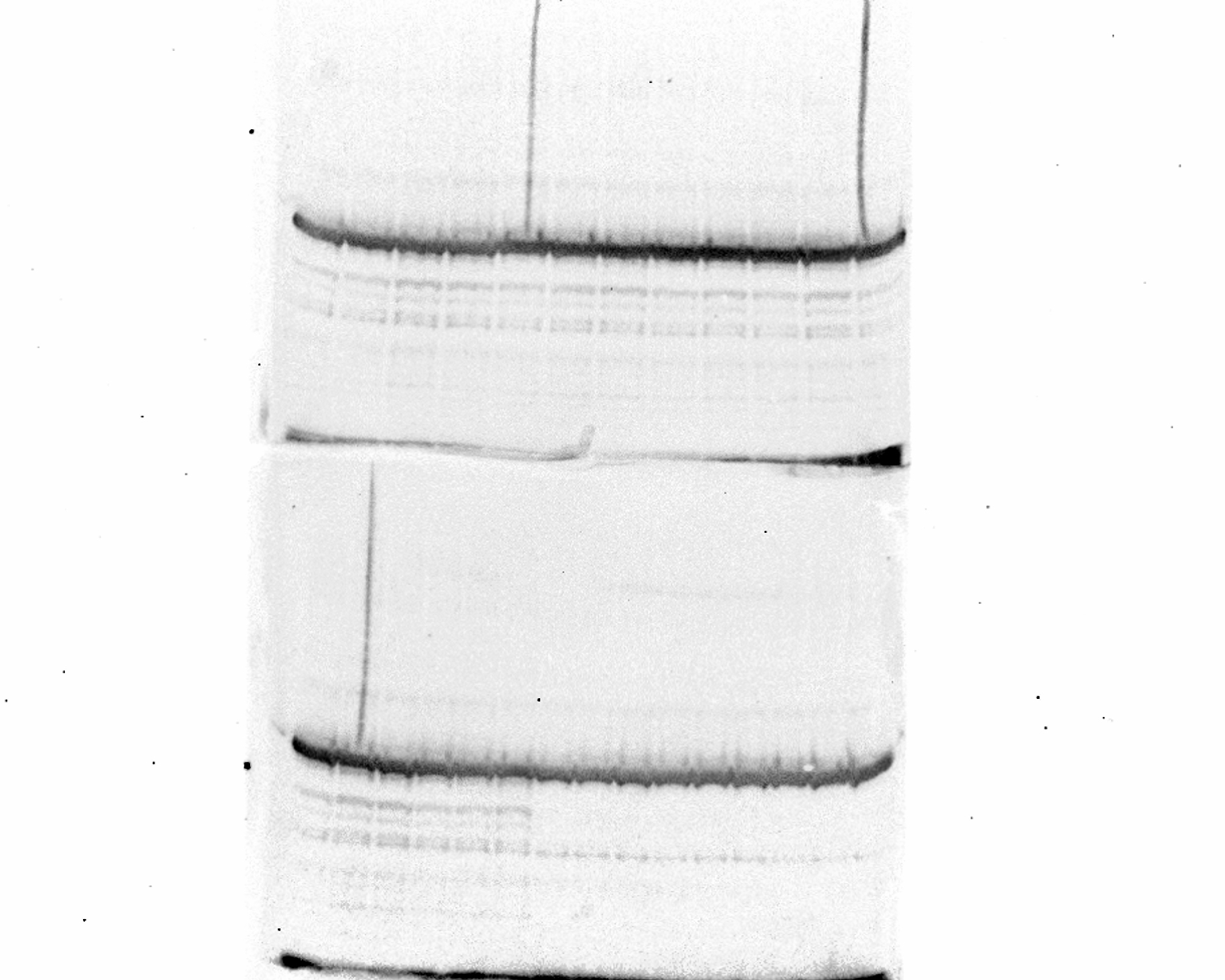

Supplement: S1 Data — (ZIP) [file pgen.1011059.s014.zip › SIdata/Figure 2 + S1/2B+S1B_WB Stat phase+recovery RpoS/2022-03-30/lmbchemidoc 2022-03-30 14h37m55s(DyLight 800).jpg]

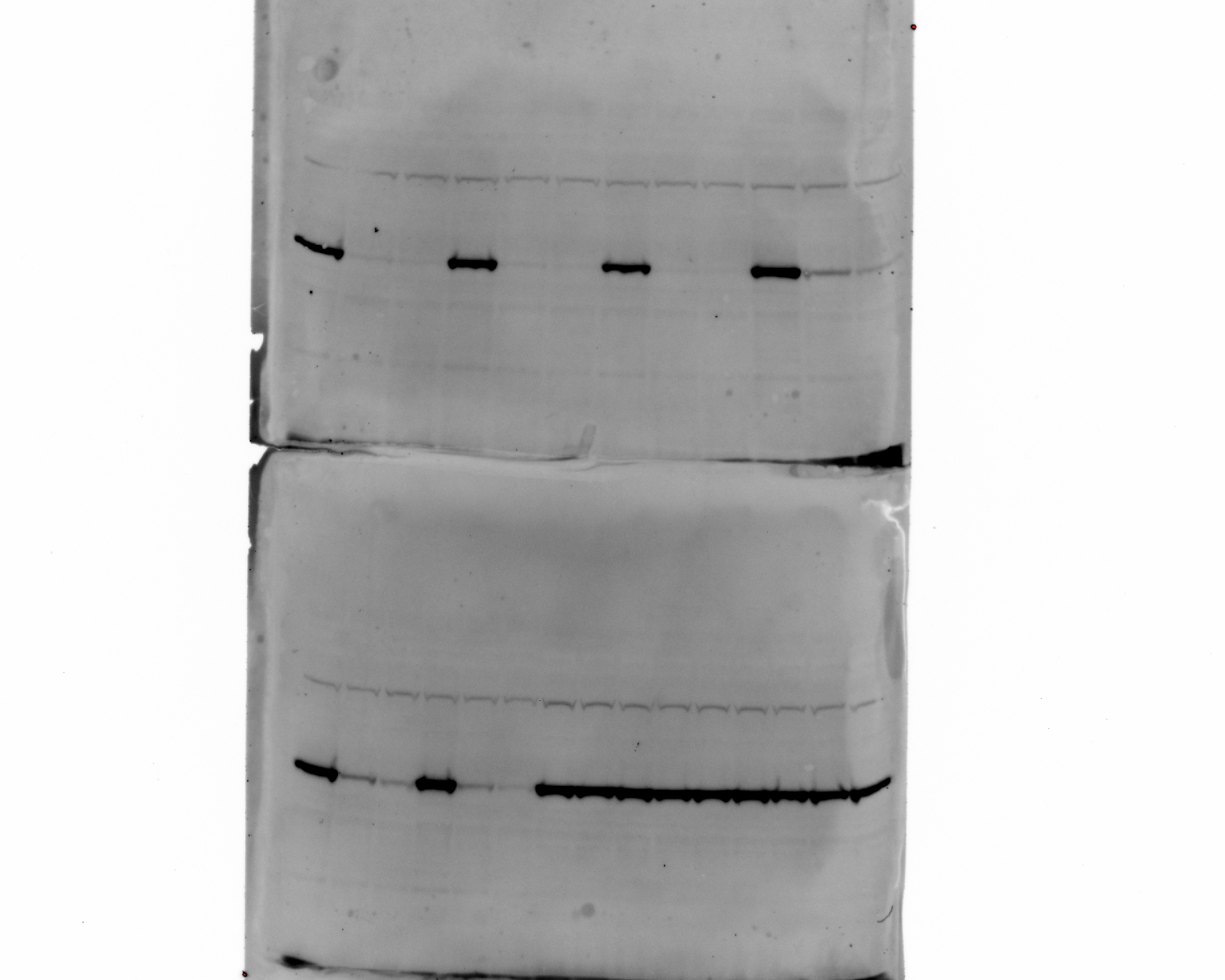

Supplement: S1 Data — (ZIP) [file pgen.1011059.s014.zip › SIdata/Figure 2 + S1/2B+S1B_WB Stat phase+recovery RpoS/2022-03-30/lmbchemidoc 2022-03-30 14h37m55s(StarBright B700).jpg]

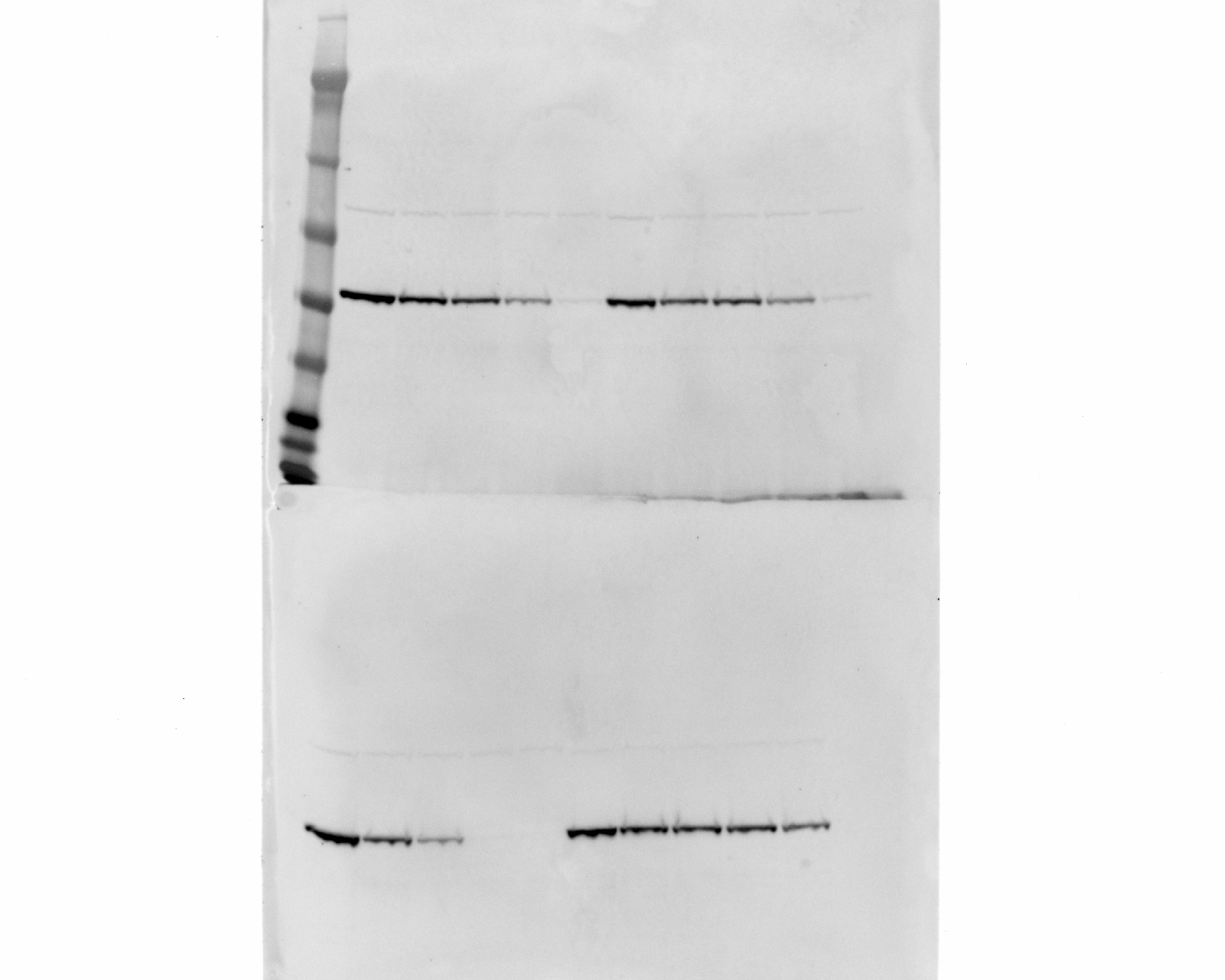

Supplement: S1 Data — (ZIP) [file pgen.1011059.s014.zip › SIdata/Figure 2 + S1/2B+S1B_WB Stat phase+recovery RpoS/2022-03-31/lmbchemidoc 2022-03-31 17h37m05s(StarBright B700).jpg]

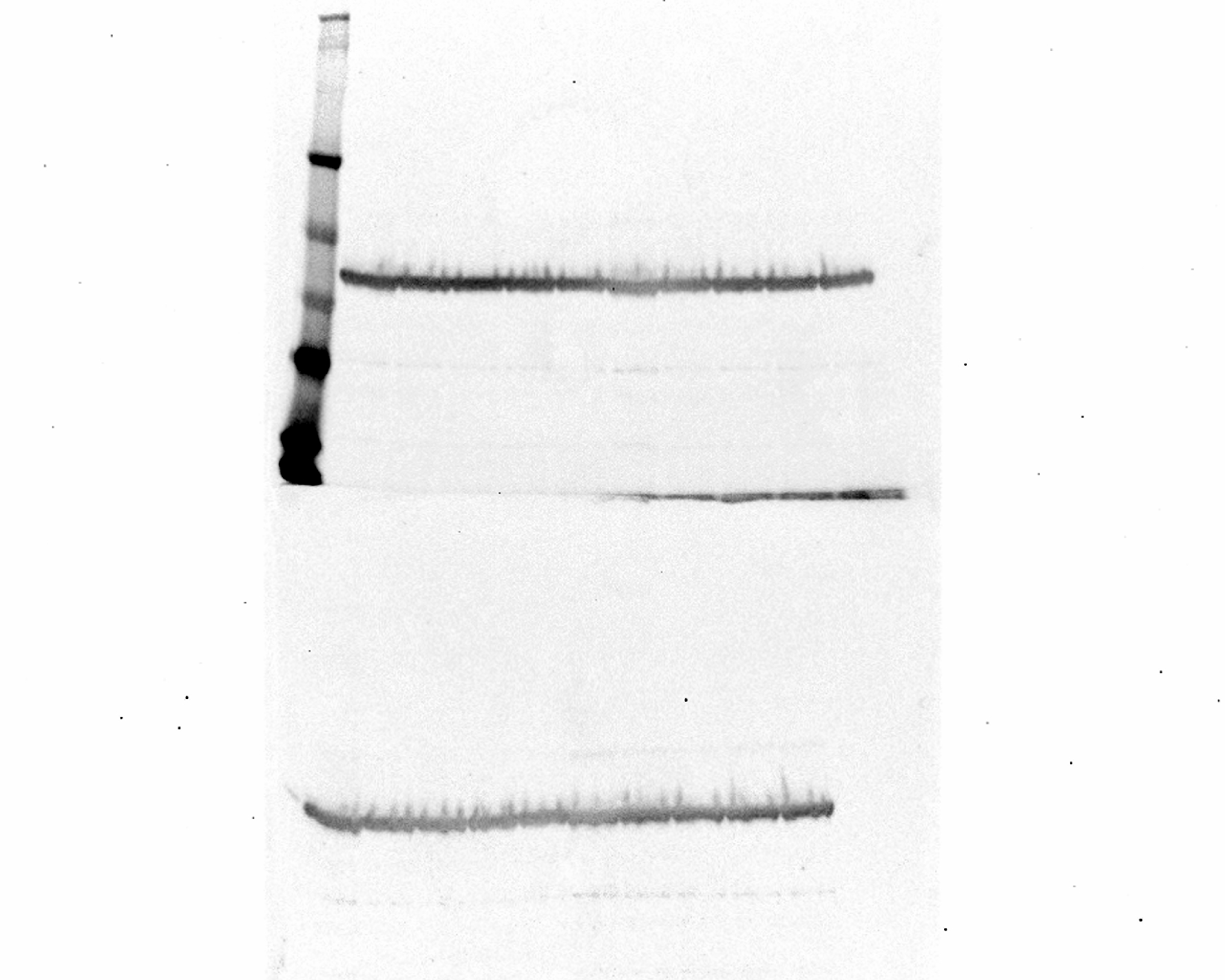

Supplement: S1 Data — (ZIP) [file pgen.1011059.s014.zip › SIdata/Figure 2 + S1/2B+S1B_WB Stat phase+recovery RpoS/2022-03-31/lmbchemidoc 2022-03-31 17h36m00s(DyLight 800).jpg]

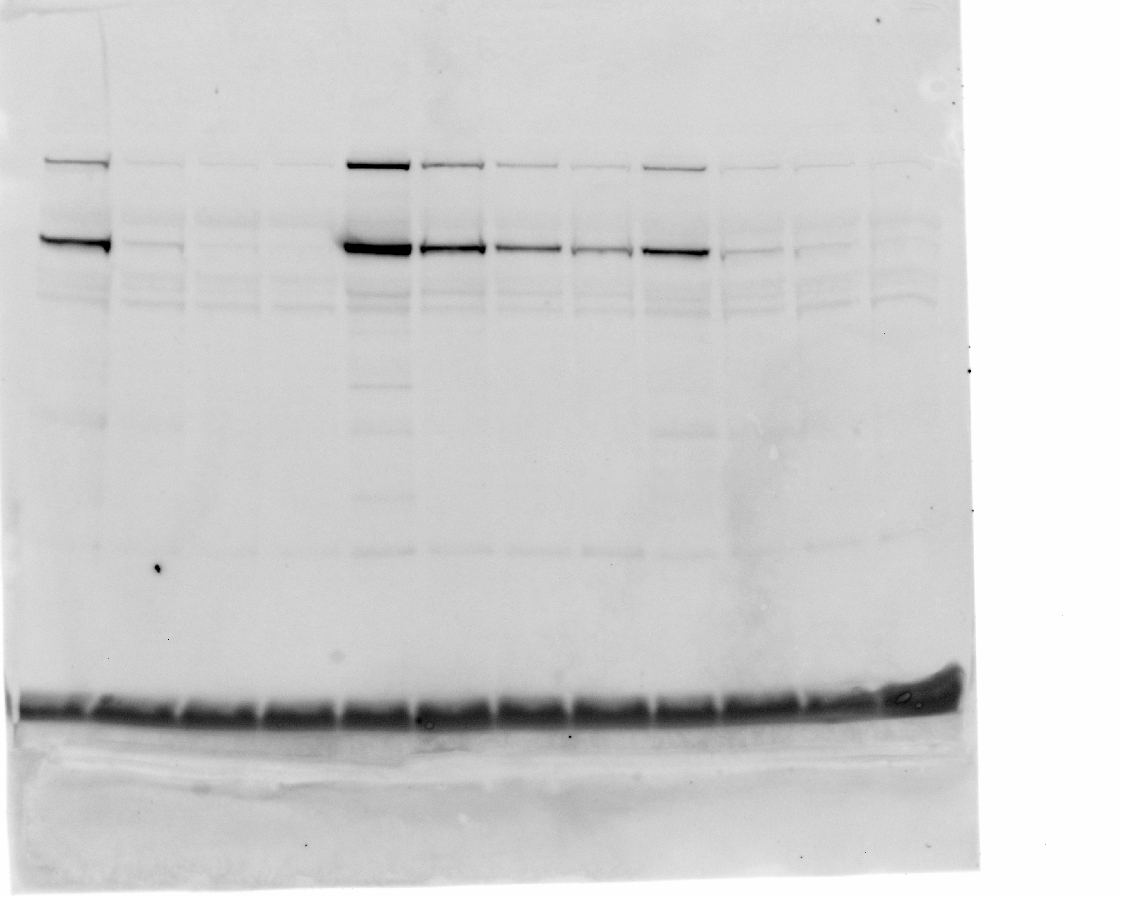

Supplement: S1 Data — (ZIP) [file pgen.1011059.s014.zip › SIdata/Figure 2 + S1/2B+S1B_WB Stat phase+recovery RpoS/2022-03-09/lmbchemidoc 2022-03-09 16h06m09s(StarBright B700).jpg]

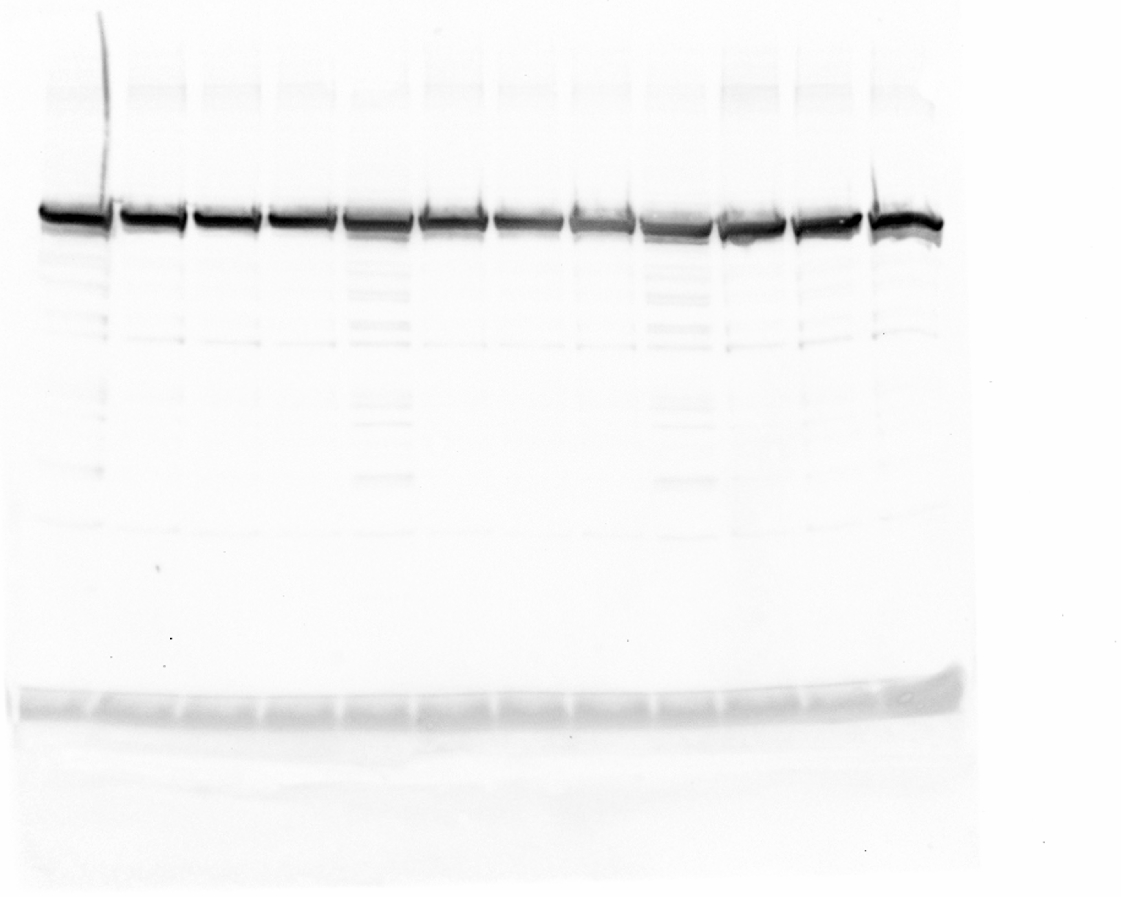

Supplement: S1 Data — (ZIP) [file pgen.1011059.s014.zip › SIdata/Figure 2 + S1/2B+S1B_WB Stat phase+recovery RpoS/2022-03-09/lmbchemidoc 2022-03-09 16h06m09s(DyLight 800).jpg]

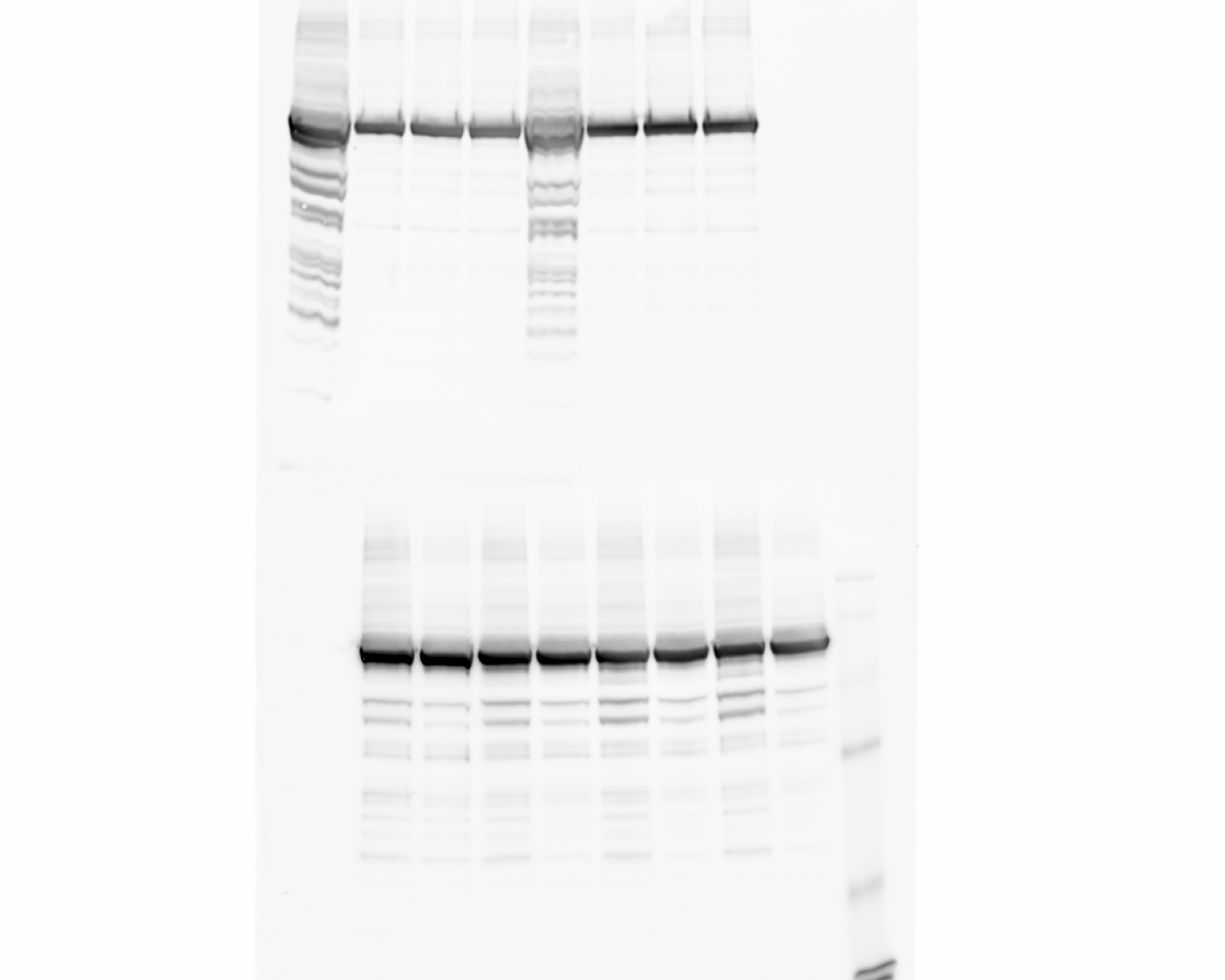

Supplement: S1 Data — (ZIP) [file pgen.1011059.s014.zip › SIdata/Figure 2 + S1/2B+S1B_WB Stat phase+recovery RpoS/2022-03-04/lmbchemidoc 2022-03-03 18h40m35s(DyLight 800).tif]

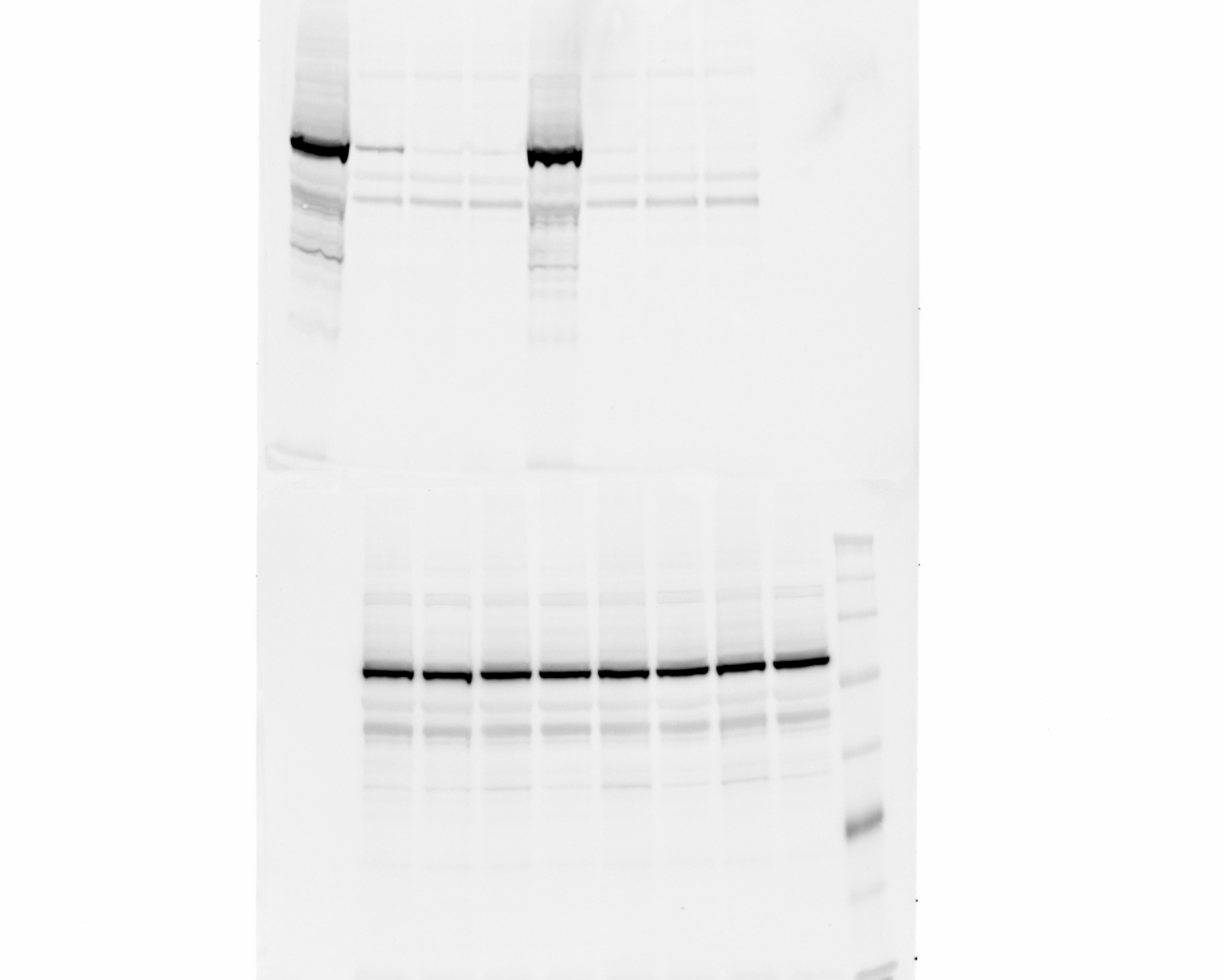

Supplement: S1 Data — (ZIP) [file pgen.1011059.s014.zip › SIdata/Figure 2 + S1/2B+S1B_WB Stat phase+recovery RpoS/2022-03-04/lmbchemidoc 2022-03-03 18h40m35s(StarBright B700).tif]

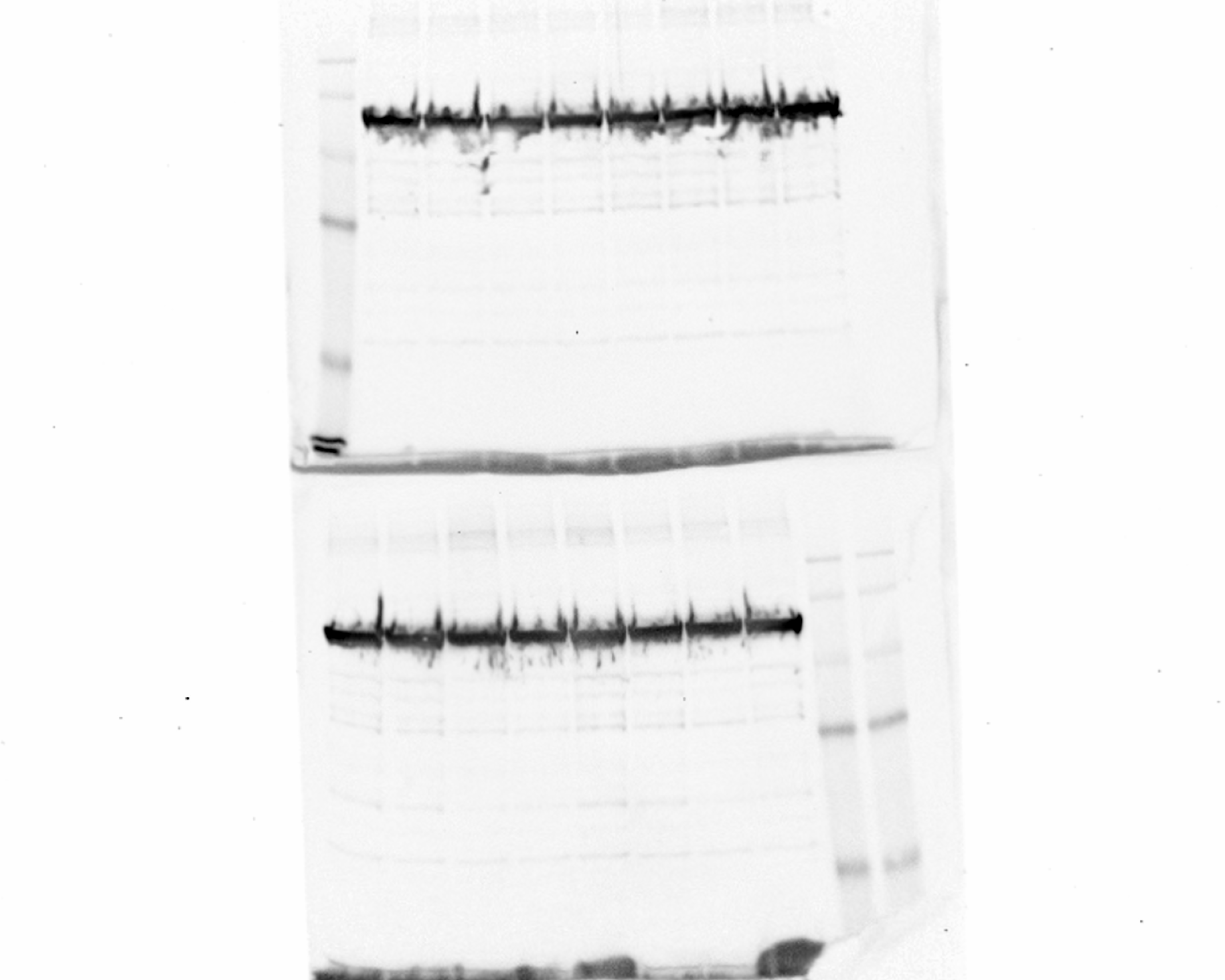

Supplement: S1 Data — (ZIP) [file pgen.1011059.s014.zip › SIdata/Figure 2 + S1/2B+S1B_WB Stat phase+recovery RpoS/2022-01-14/lmbchemidoc 2022-01-14 17h18m22s(DyLight 800).tif]

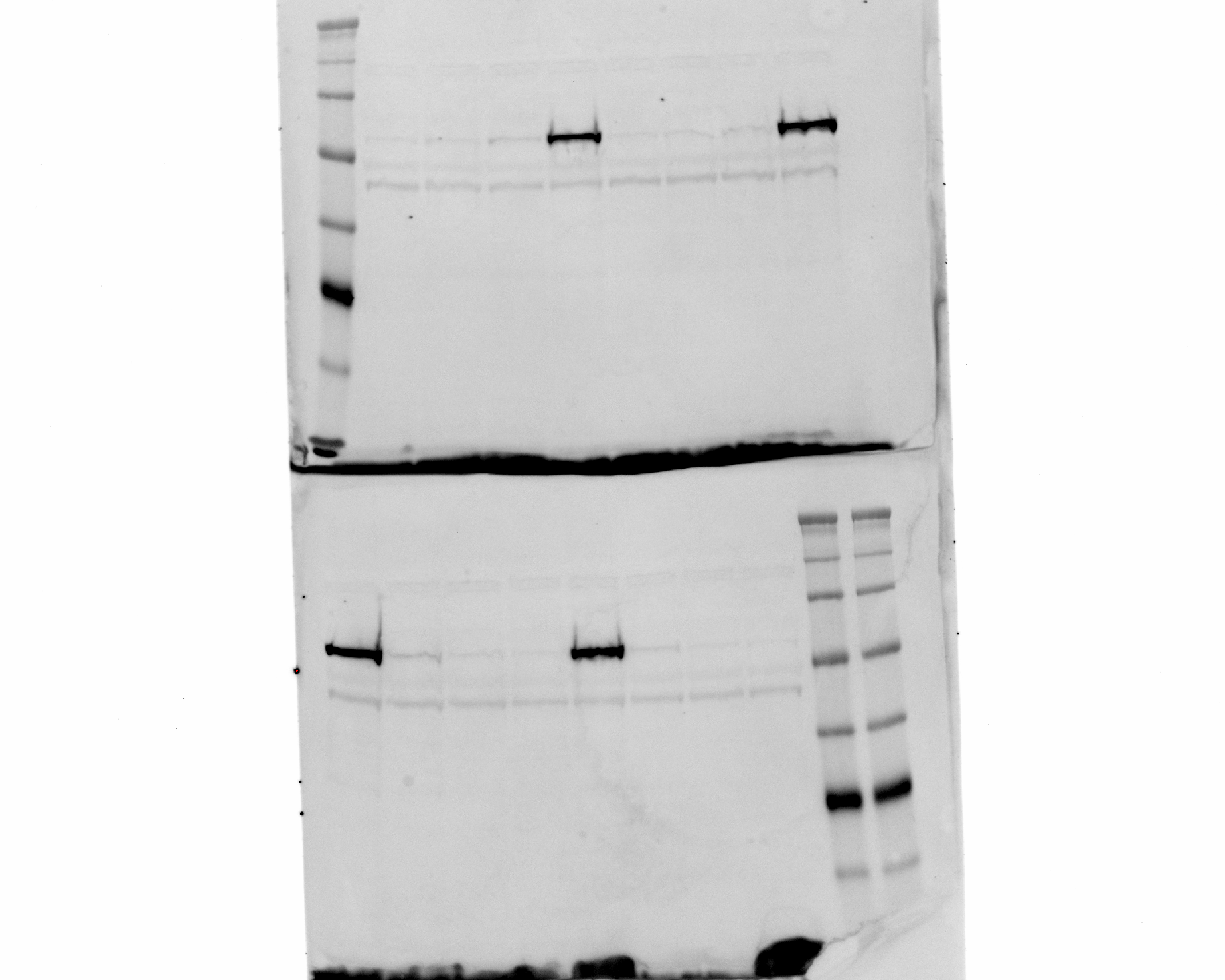

Supplement: S1 Data — (ZIP) [file pgen.1011059.s014.zip › SIdata/Figure 2 + S1/2B+S1B_WB Stat phase+recovery RpoS/2022-01-14/lmbchemidoc 2022-01-14 17h18m22s(StarBright B700).tif]

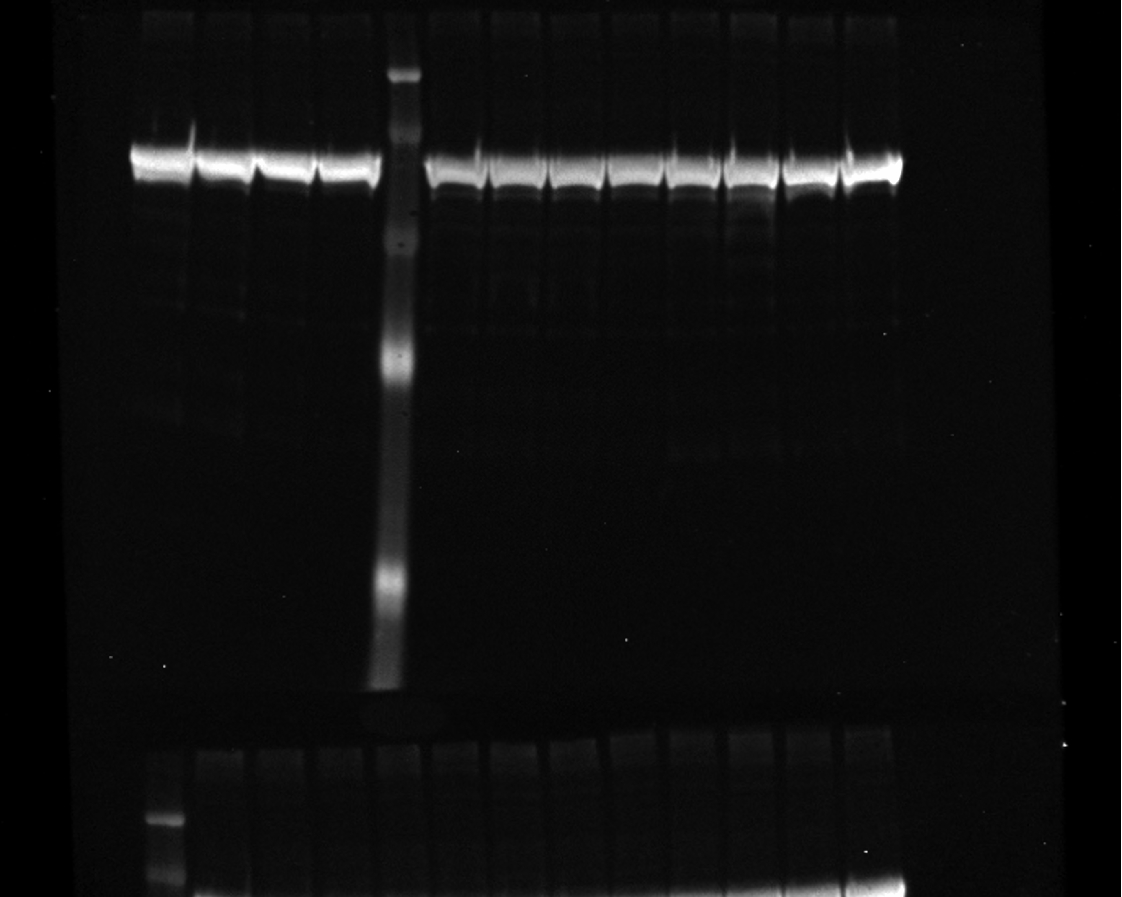

Supplement: S1 Data — (ZIP) [file pgen.1011059.s014.zip › SIdata/Figure 5 + S5 + S6/S5/S5C_WB phosphate starvation RpoS/lmbchemidoc 2021-01-25 17h40m07s(DyLight 800).jpg]

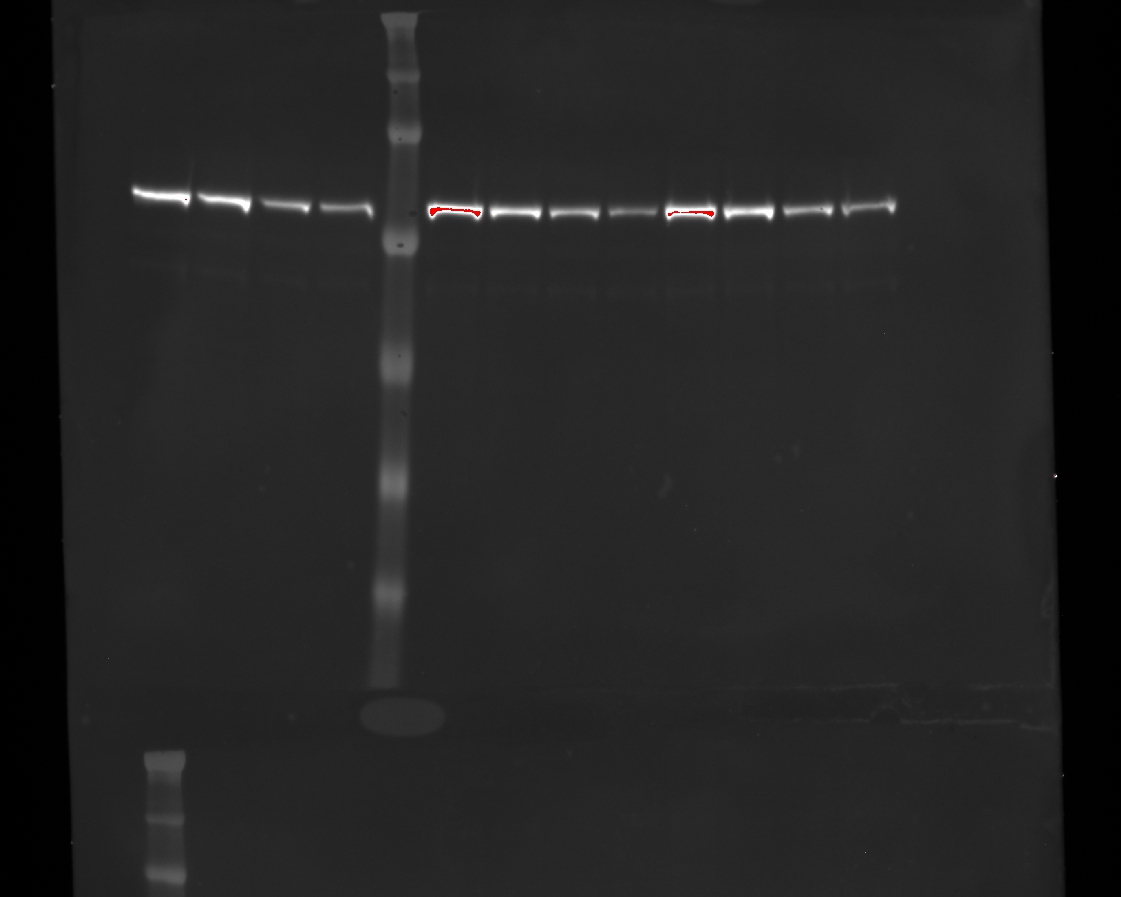

Supplement: S1 Data — (ZIP) [file pgen.1011059.s014.zip › SIdata/Figure 5 + S5 + S6/S5/S5C_WB phosphate starvation RpoS/lmbchemidoc 2021-01-25 17h40m07s(StarBright B700).jpg]

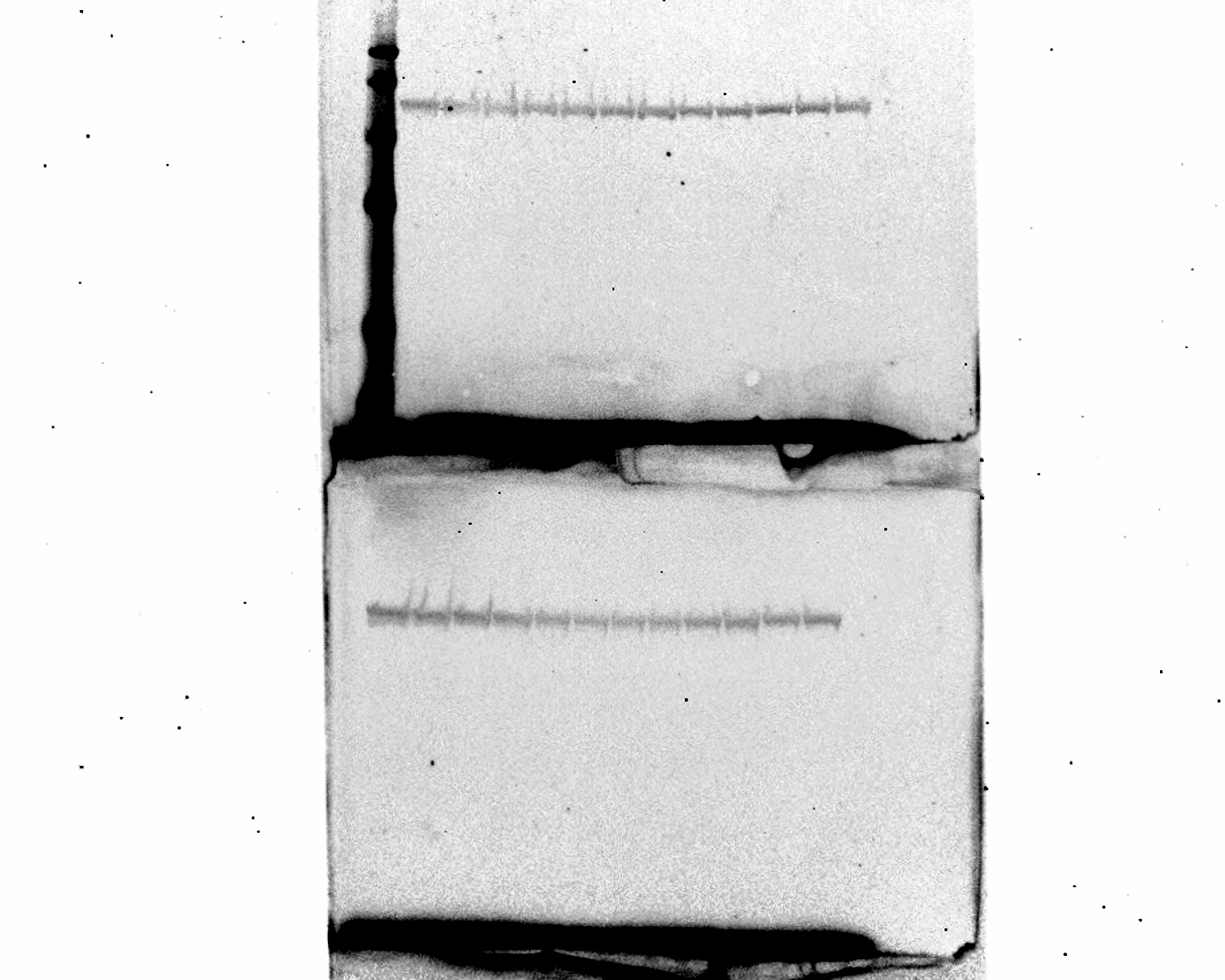

Supplement: S1 Data — (ZIP) [file pgen.1011059.s014.zip › SIdata/Figure 5 + S5 + S6/S5/S5C_WB phosphate starvation RpoS/lmbchemidoc 2023-12-14 17h51m30s(DyLight 800).jpg]

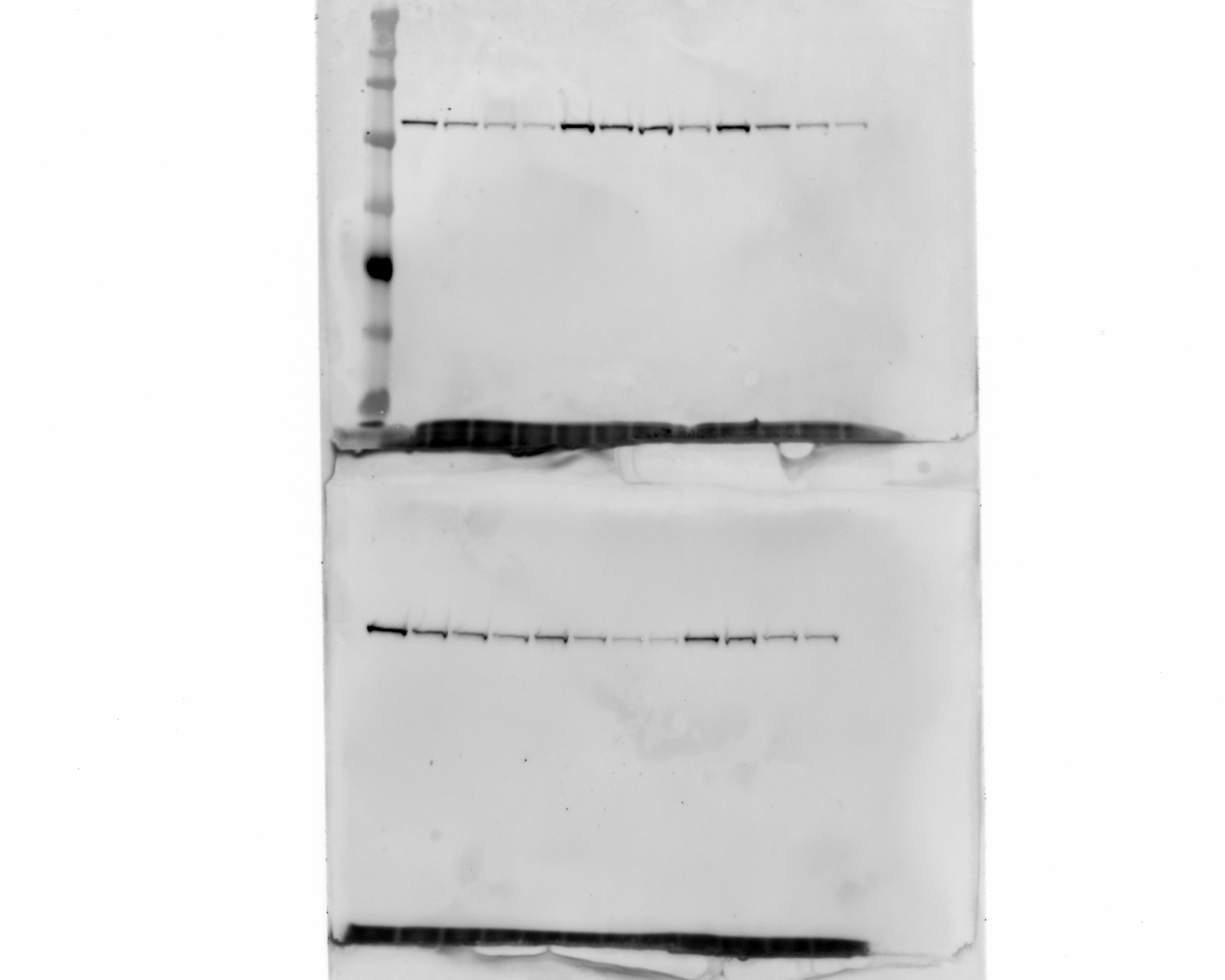

Supplement: S1 Data — (ZIP) [file pgen.1011059.s014.zip › SIdata/Figure 5 + S5 + S6/S5/S5C_WB phosphate starvation RpoS/lmbchemidoc 2023-12-14 17h51m30s(StarBright B700).jpg]

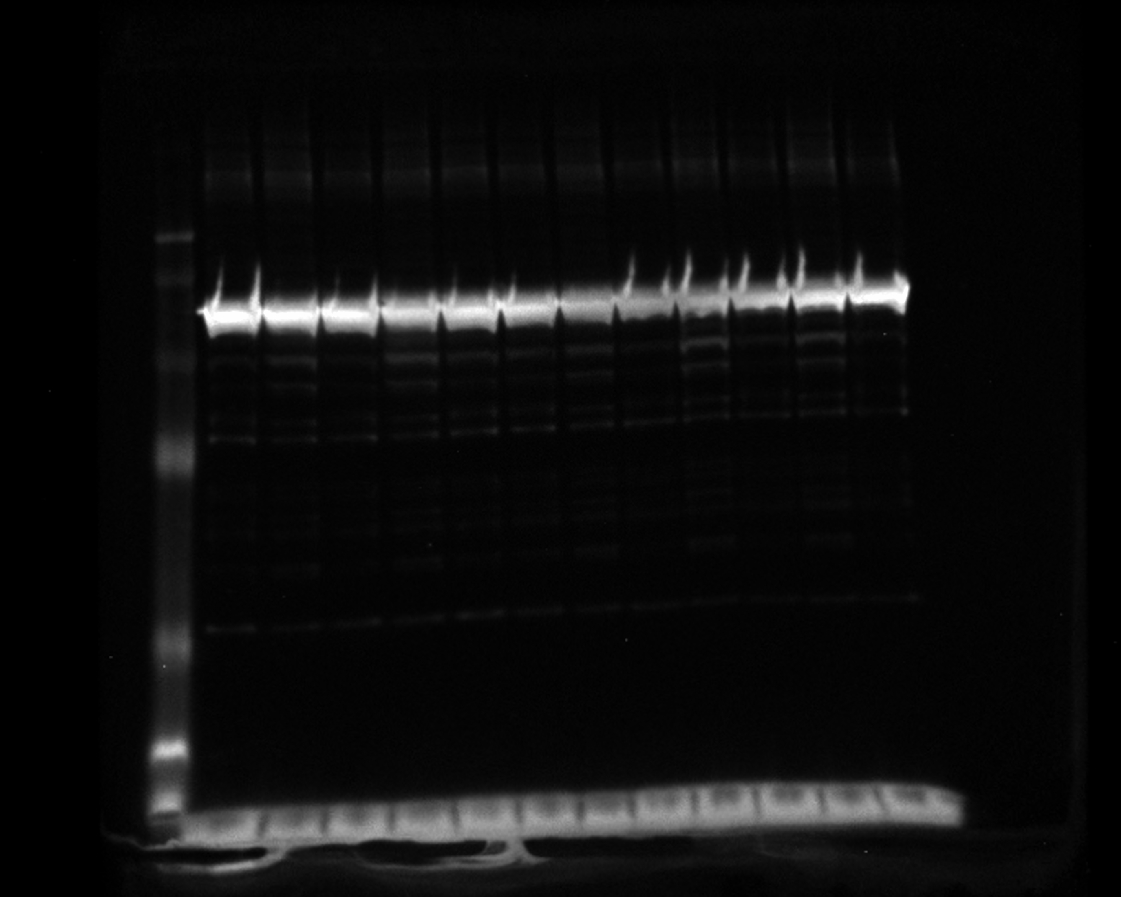

Supplement: S1 Data — (ZIP) [file pgen.1011059.s014.zip › SIdata/Figure 5 + S5 + S6/S5/S5D_WB glucose starvation RpoS/lmbchemidoc 2021-03-19 18h40m30s(DyLight 800).jpg]

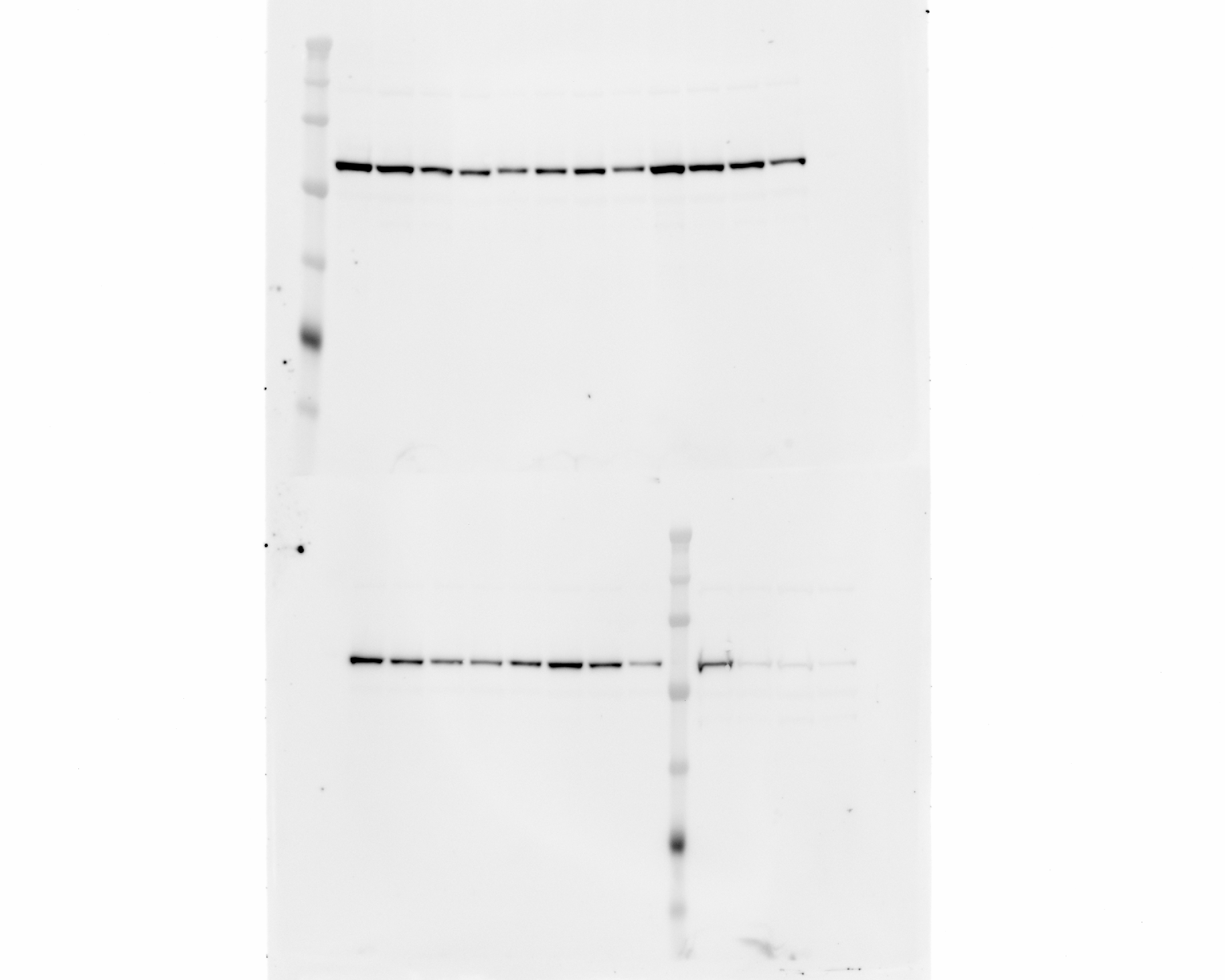

Supplement: S1 Data — (ZIP) [file pgen.1011059.s014.zip › SIdata/Figure 5 + S5 + S6/S5/S5D_WB glucose starvation RpoS/lmbchemidoc 2023-12-15 16h49m48s(StarBright B700).tif]

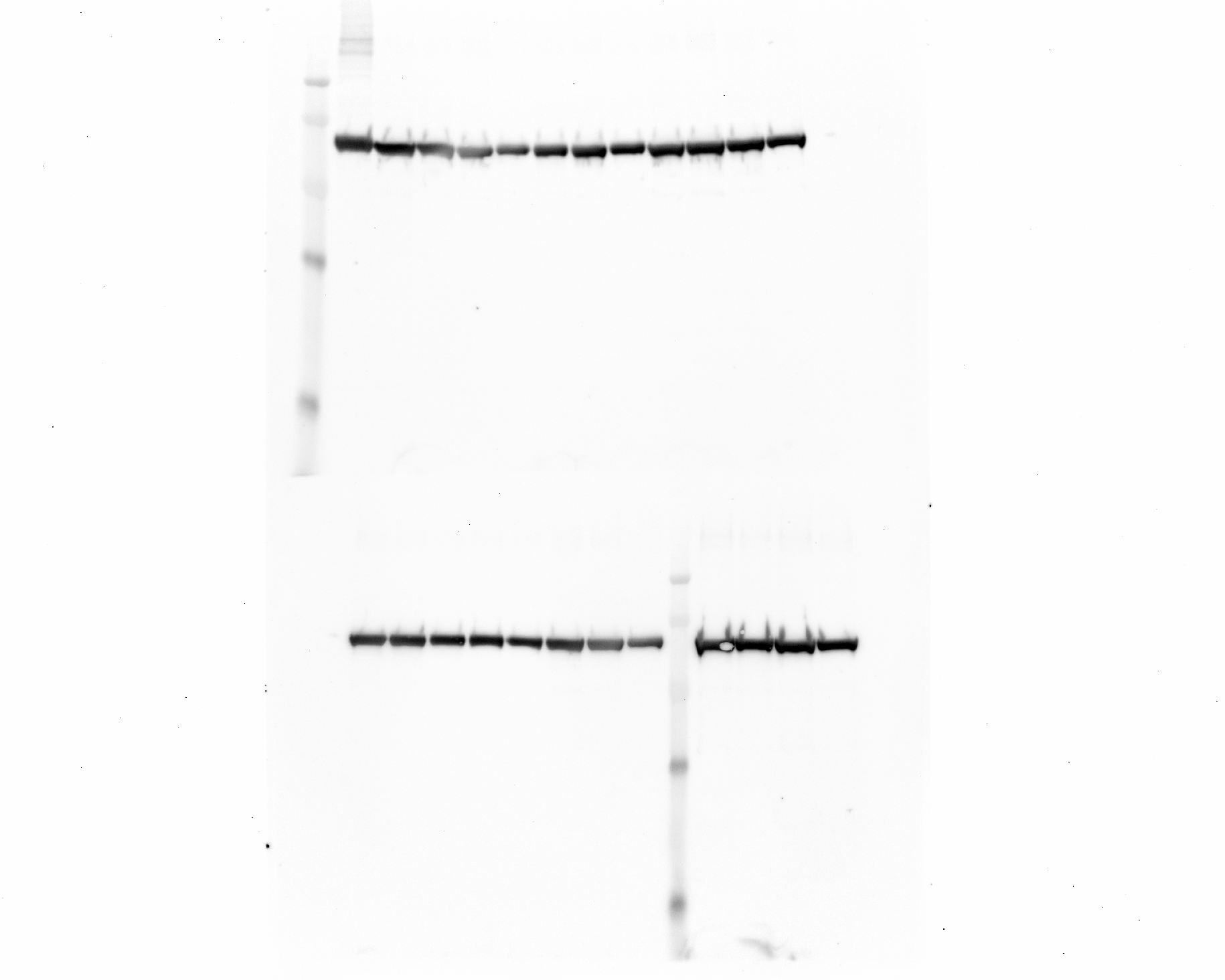

Supplement: S1 Data — (ZIP) [file pgen.1011059.s014.zip › SIdata/Figure 5 + S5 + S6/S5/S5D_WB glucose starvation RpoS/lmbchemidoc 2023-12-15 16h49m48s(DyLight 800).tif]

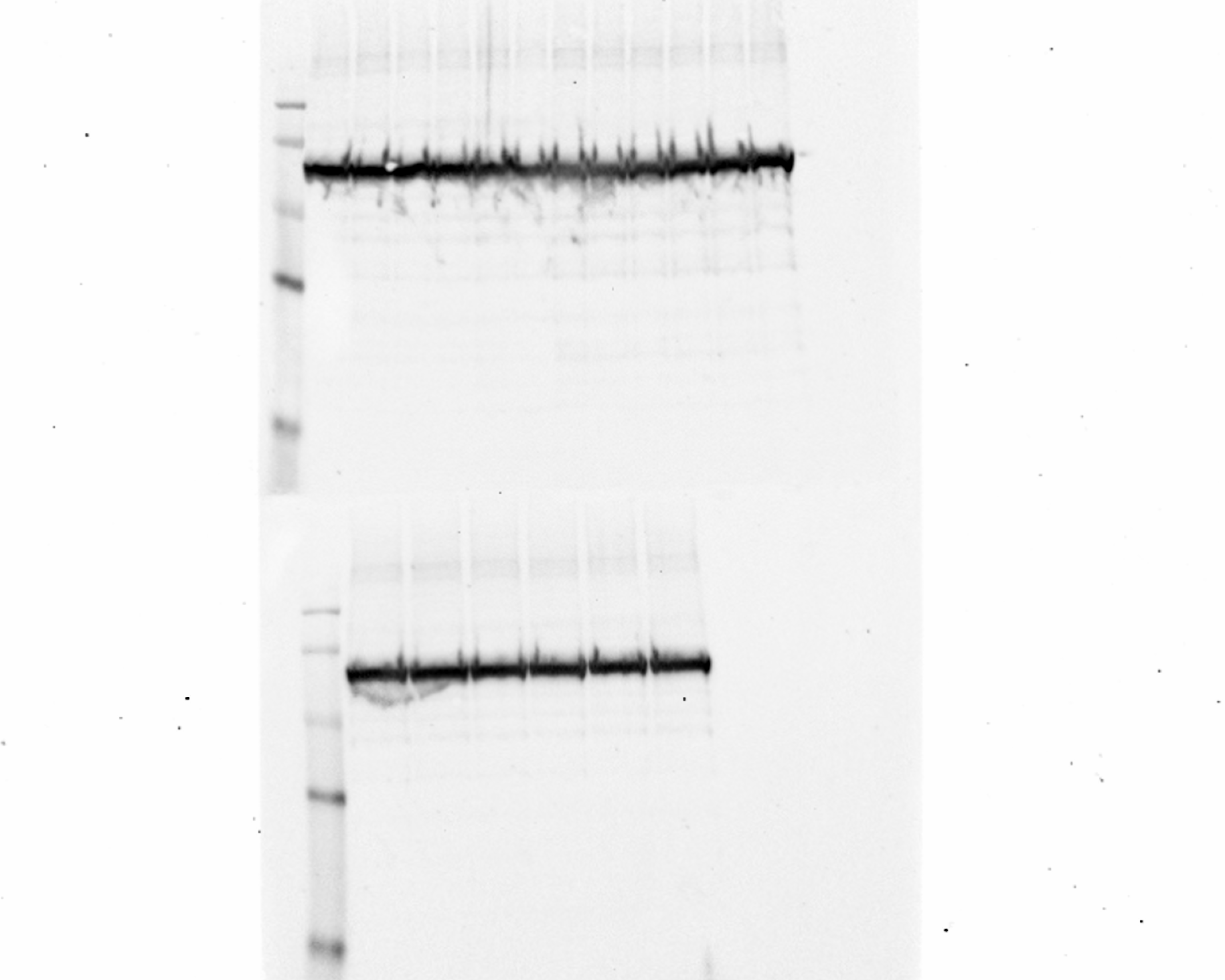

Supplement: S1 Data — (ZIP) [file pgen.1011059.s014.zip › SIdata/Figure 8 + S9/Fig8_WB/Set 2/lmbchemidoc 2023-03-03 16h45m42s(DyLight 800).tif]

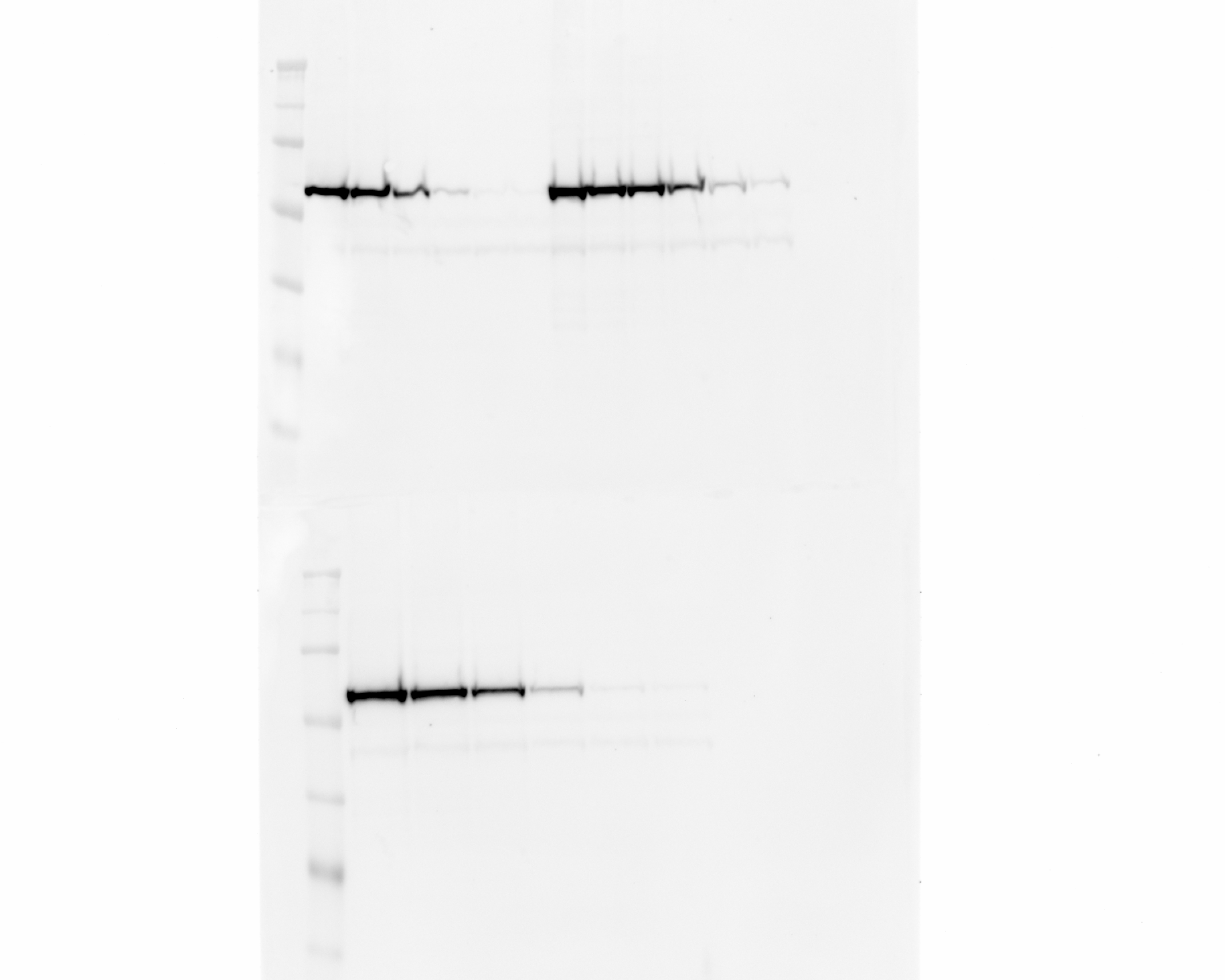

Supplement: S1 Data — (ZIP) [file pgen.1011059.s014.zip › SIdata/Figure 8 + S9/Fig8_WB/Set 2/lmbchemidoc 2023-03-03 16h45m42s(StarBright B700).tif]

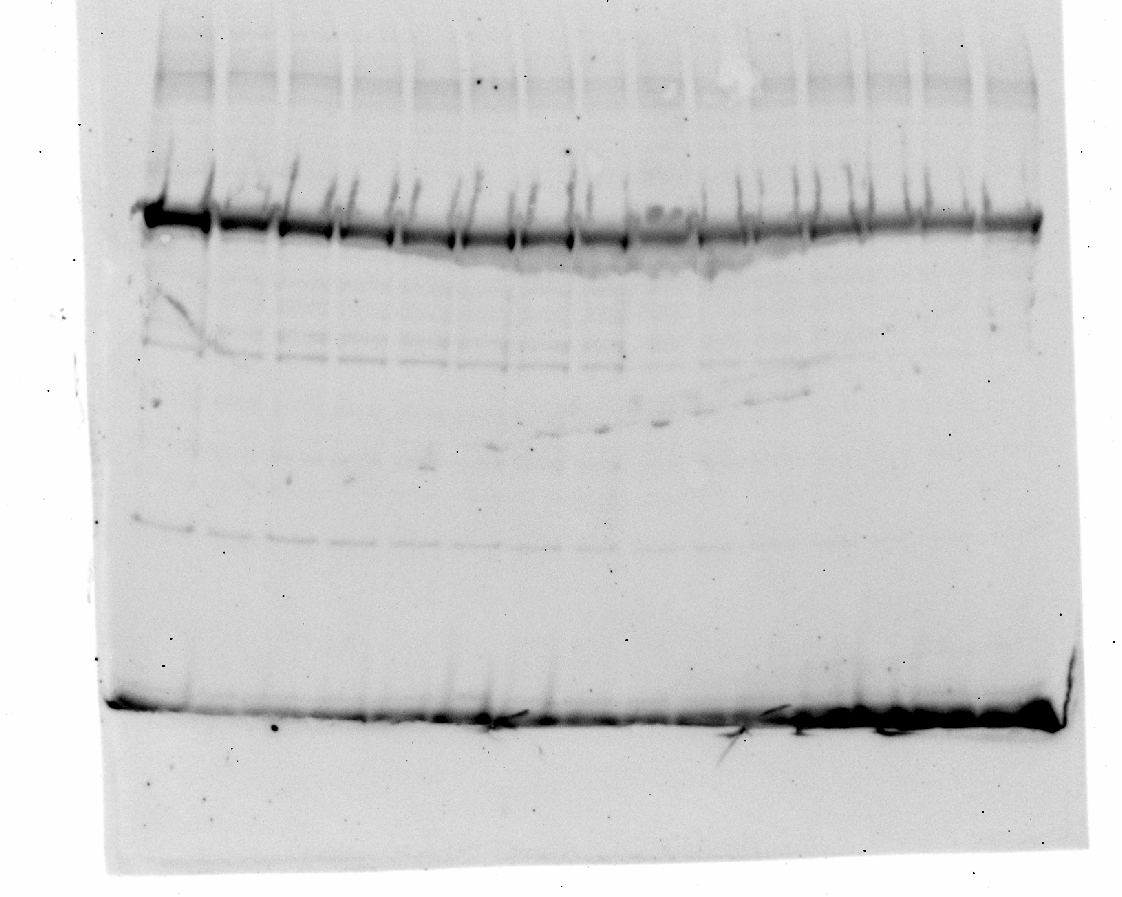

Supplement: S1 Data — (ZIP) [file pgen.1011059.s014.zip › SIdata/Figure 8 + S9/Fig8_WB/Set 3/lmbchemidoc 2023-03-31 14h50m06s(DyLight 800).tif]

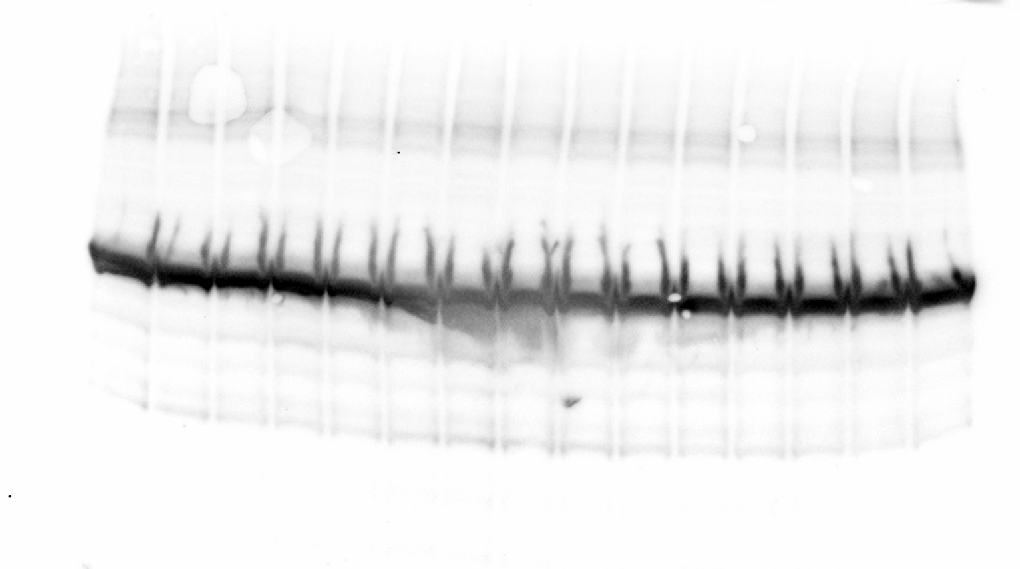

Supplement: S1 Data — (ZIP) [file pgen.1011059.s014.zip › SIdata/Figure 8 + S9/Fig8_WB/Set 3/lmbchemidoc 2023-03-31 14h46m34s(DyLight 800).tif]

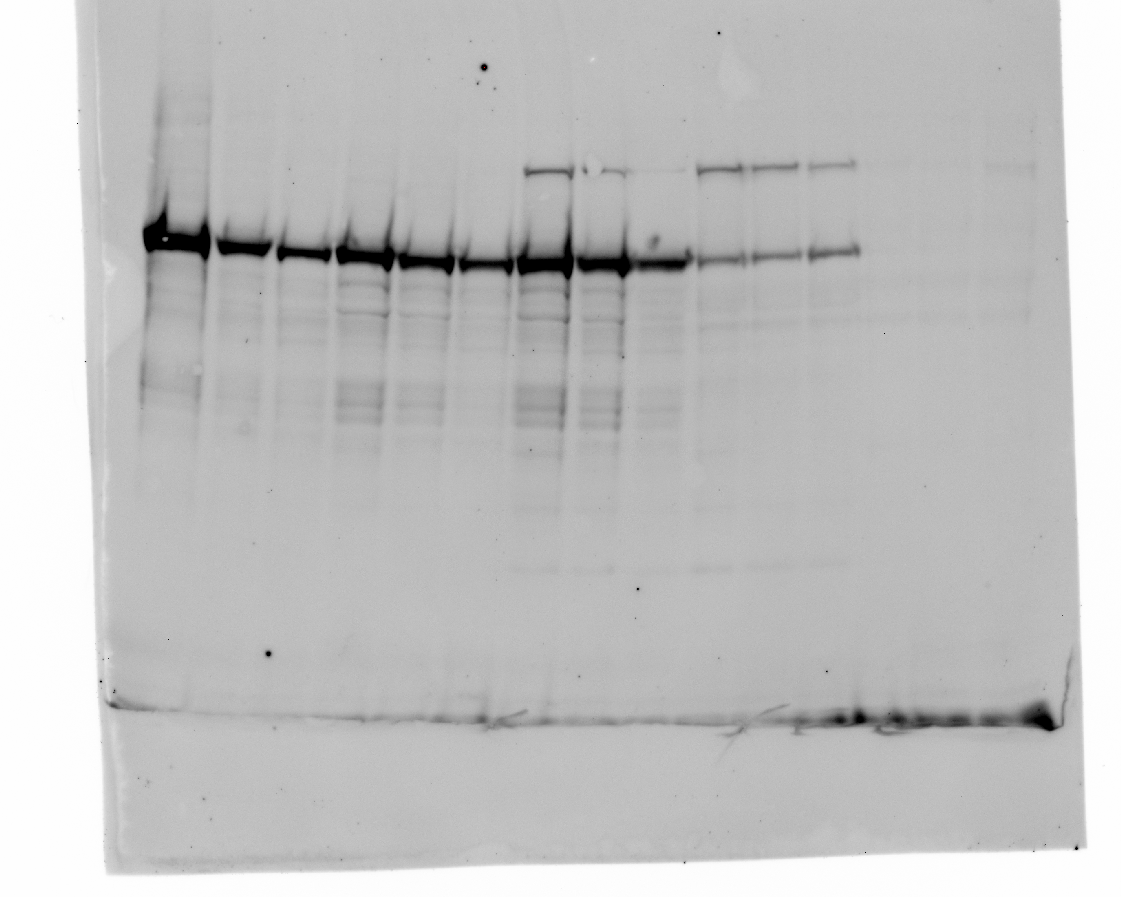

Supplement: S1 Data — (ZIP) [file pgen.1011059.s014.zip › SIdata/Figure 8 + S9/Fig8_WB/Set 3/lmbchemidoc 2023-03-31 14h50m06s(StarBright B700).tif]

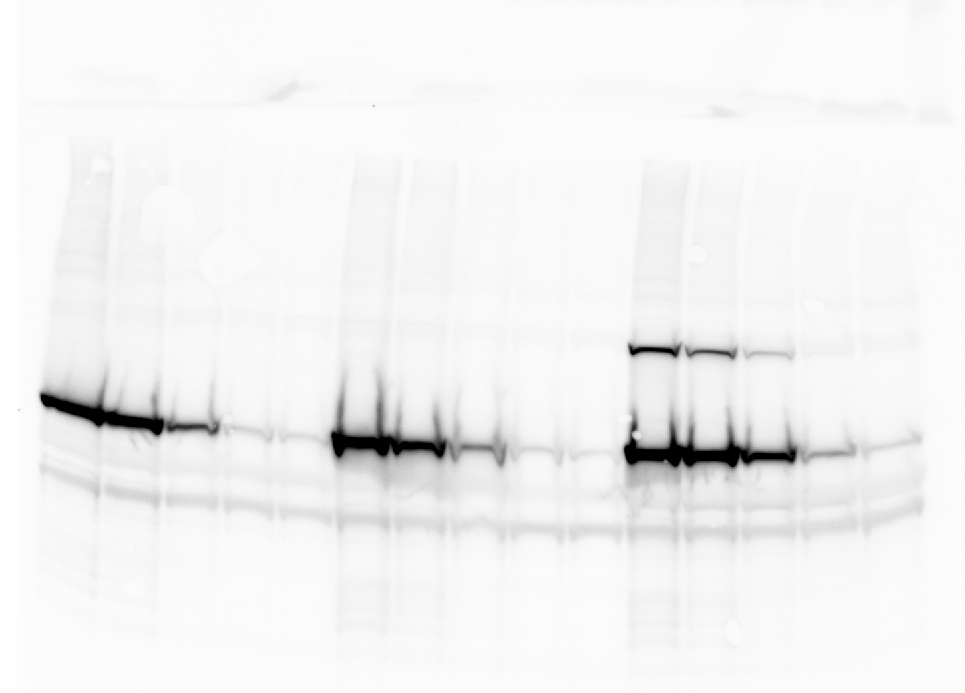

Supplement: S1 Data — (ZIP) [file pgen.1011059.s014.zip › SIdata/Figure 8 + S9/Fig8_WB/Set 3/lmbchemidoc 2023-03-31 14h46m34s(StarBright B700).tif]

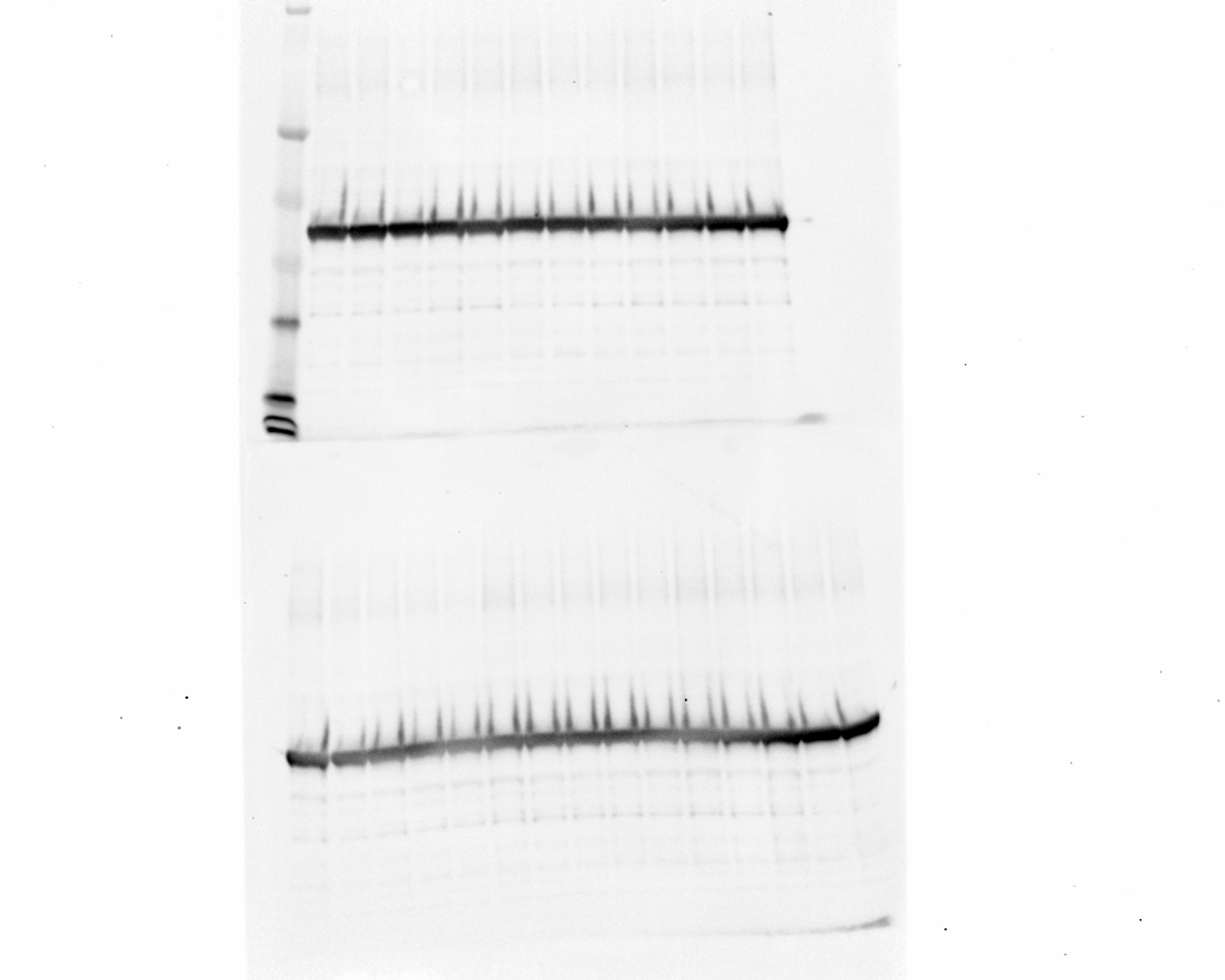

Supplement: S1 Data — (ZIP) [file pgen.1011059.s014.zip › SIdata/Figure 8 + S9/Fig8_WB/Set 4/lmbchemidoc 2023-01-13 15h36m58s(DyLight 800).tif]

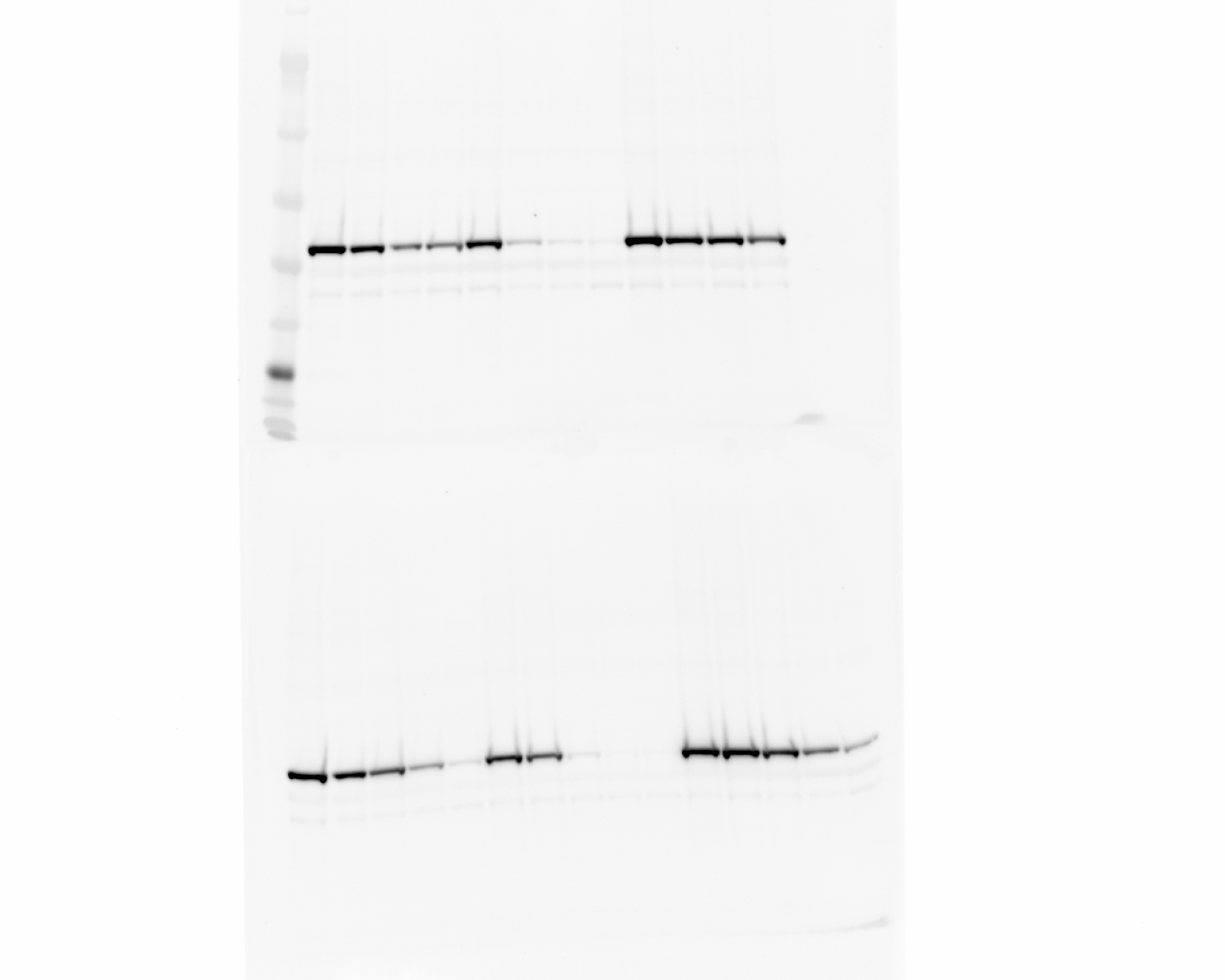

Supplement: S1 Data — (ZIP) [file pgen.1011059.s014.zip › SIdata/Figure 8 + S9/Fig8_WB/Set 4/lmbchemidoc 2023-01-13 15h36m58s(StarBright B700).tif]

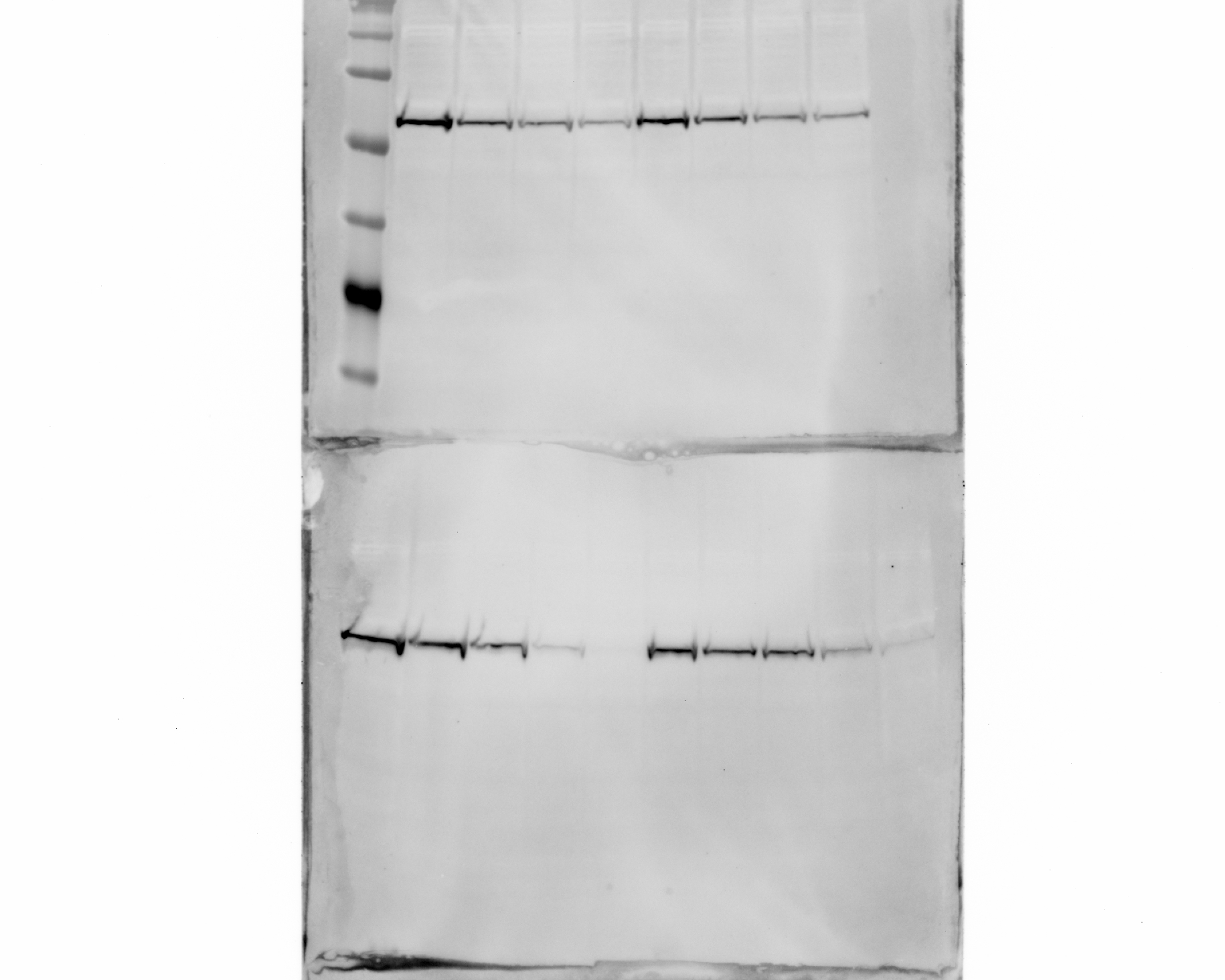

Supplement: S1 Data — (ZIP) [file pgen.1011059.s014.zip › SIdata/Figure 8 + S9/Fig8_WB/Set 1/lmbchemidoc 2023-02-23 15h58m40s(StarBright B700).jpg]

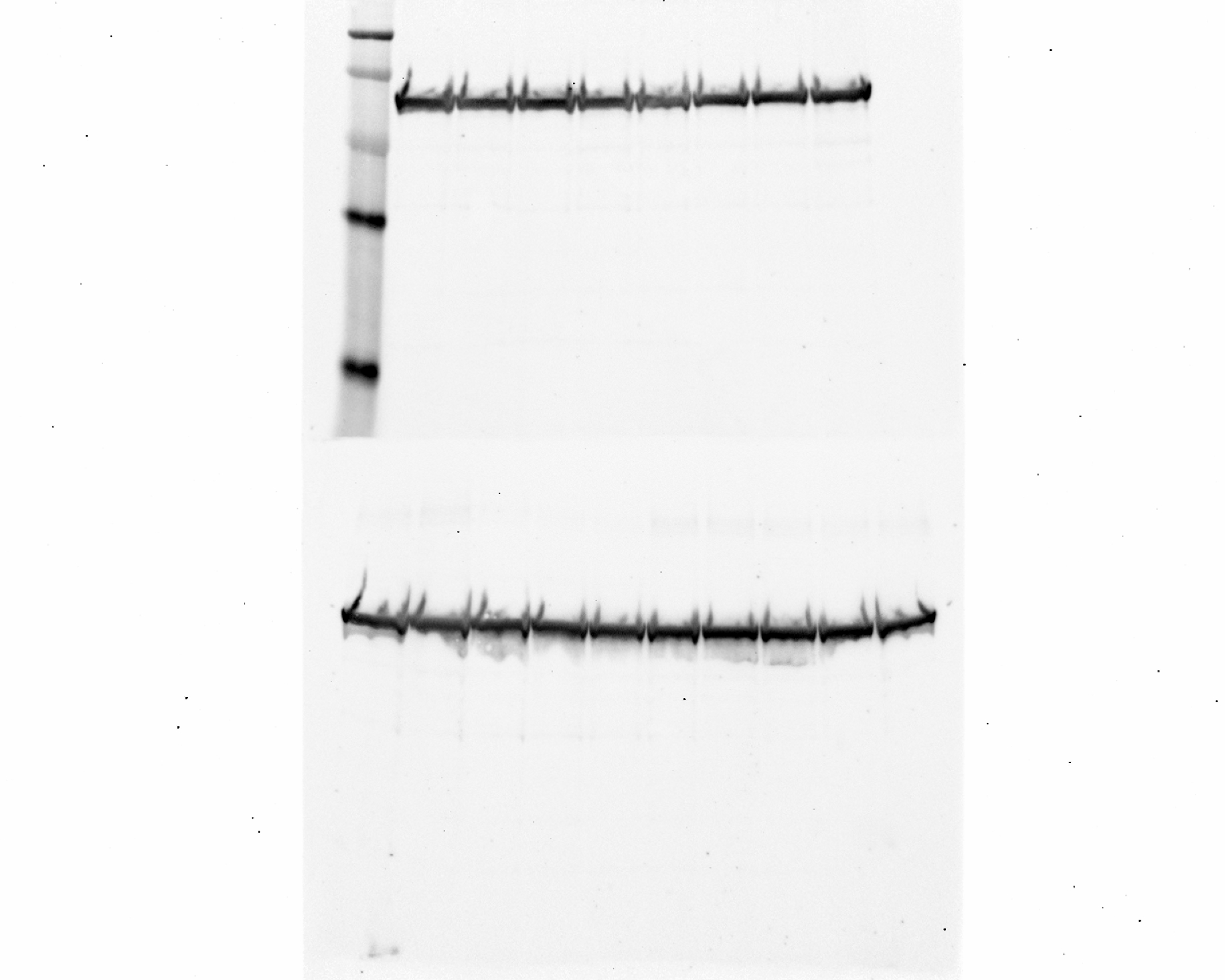

Supplement: S1 Data — (ZIP) [file pgen.1011059.s014.zip › SIdata/Figure 8 + S9/Fig8_WB/Set 1/lmbchemidoc 2023-02-23 15h58m40s(DyLight 800).jpg]

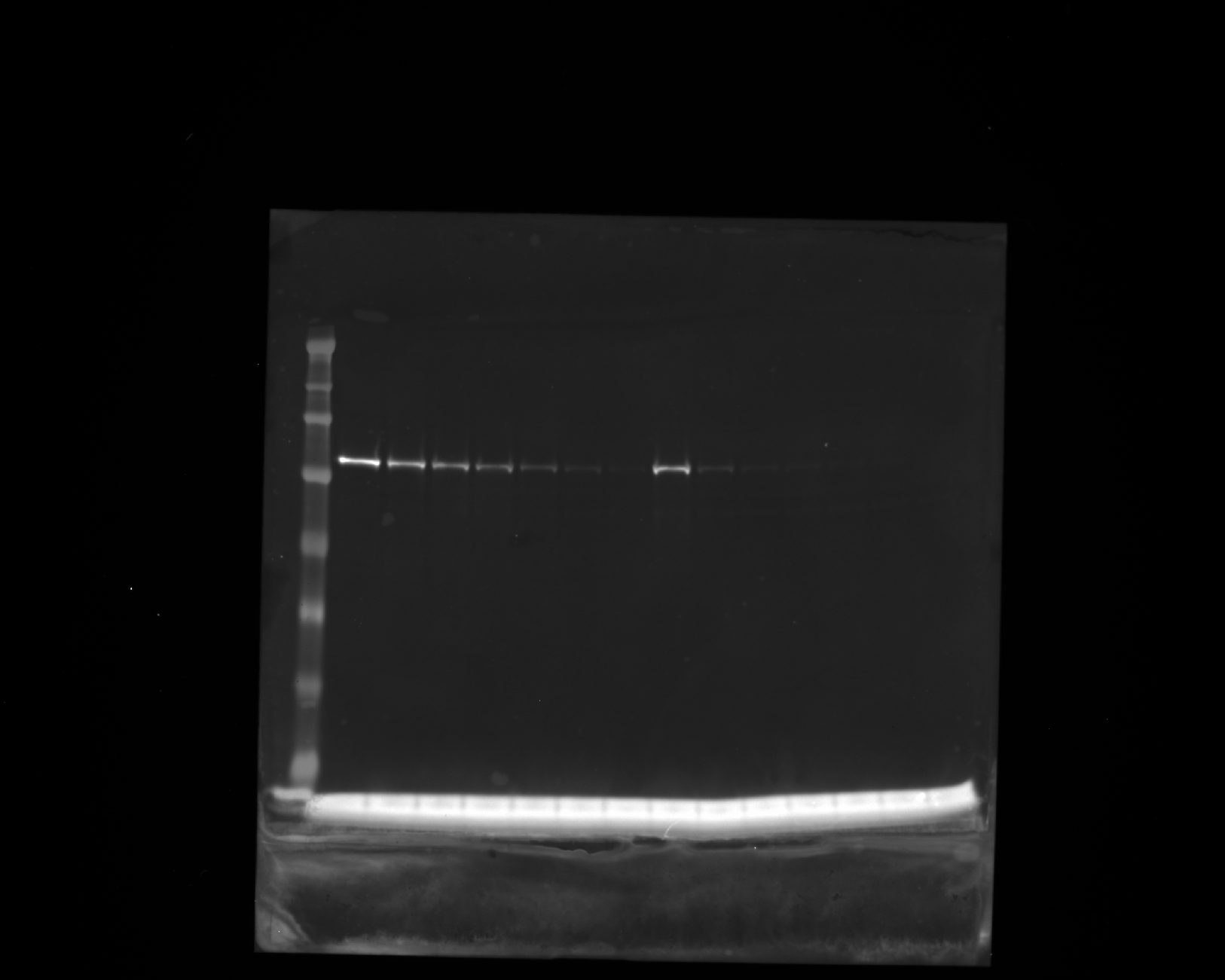

Supplement: S1 Data — (ZIP) [file pgen.1011059.s014.zip › SIdata/Figure 1 + S1A/1B+1C+1D+1E/WB RpoS chase recovery phosphate starvation/2020-07-30/lmbchemidoc 2020-07-30 18h41m38s(StarBright B700).tif]

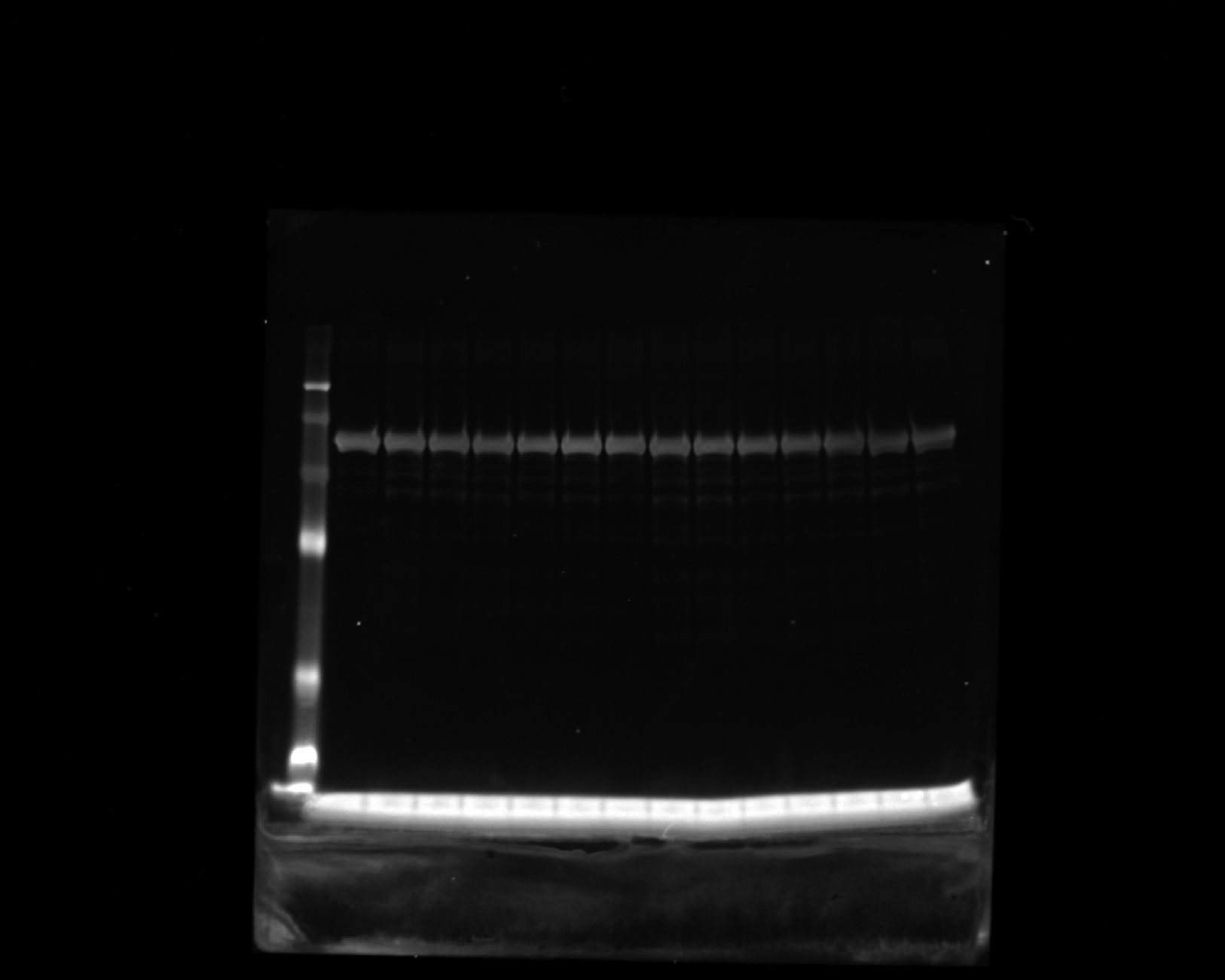

Supplement: S1 Data — (ZIP) [file pgen.1011059.s014.zip › SIdata/Figure 1 + S1A/1B+1C+1D+1E/WB RpoS chase recovery phosphate starvation/2020-07-30/lmbchemidoc 2020-07-30 18h41m38s(DyLight 800).tif]

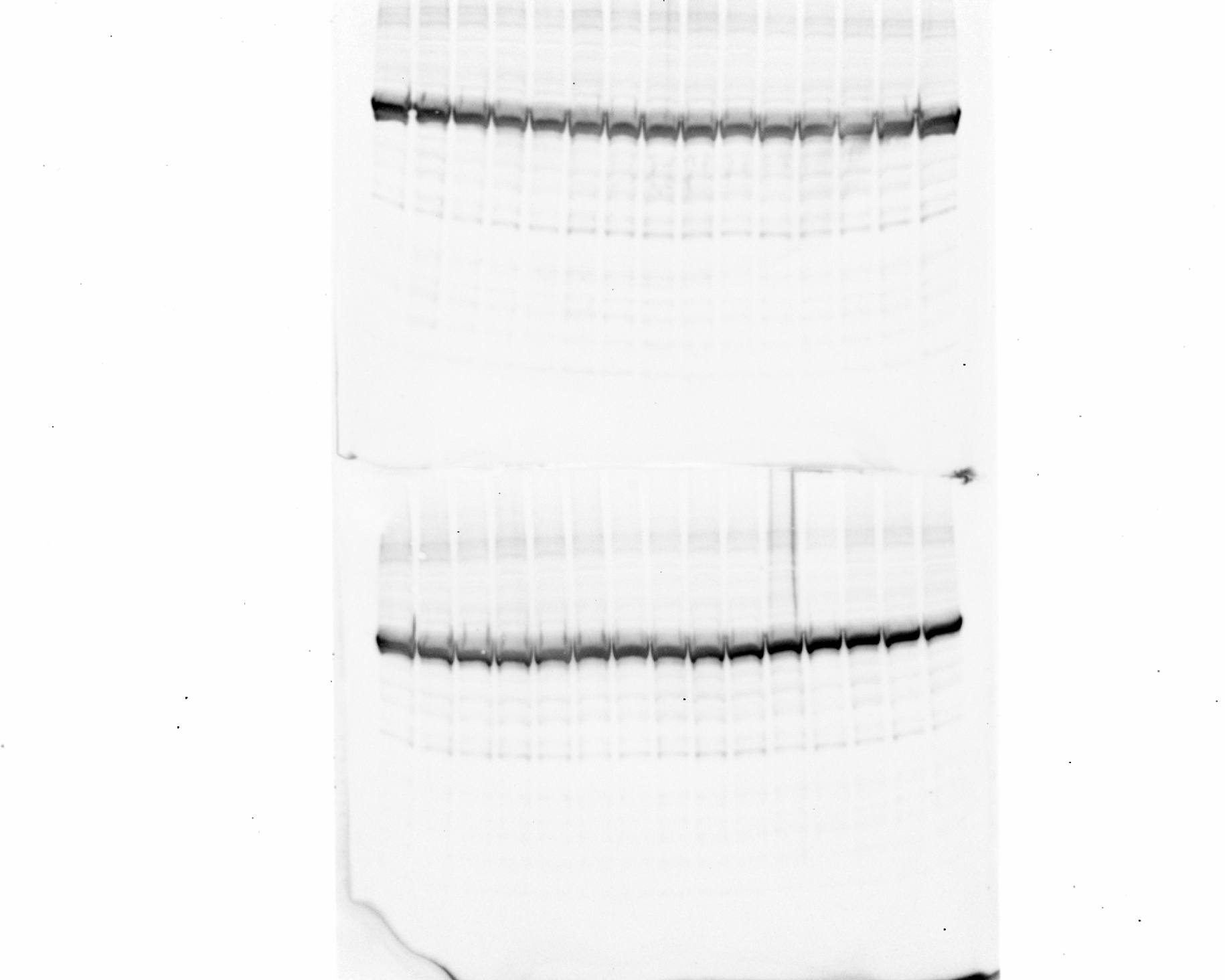

Supplement: S1 Data — (ZIP) [file pgen.1011059.s014.zip › SIdata/Figure 1 + S1A/1B+1C+1D+1E/WB RpoS chase recovery phosphate starvation/2023-02-06/EFTu.jpg]

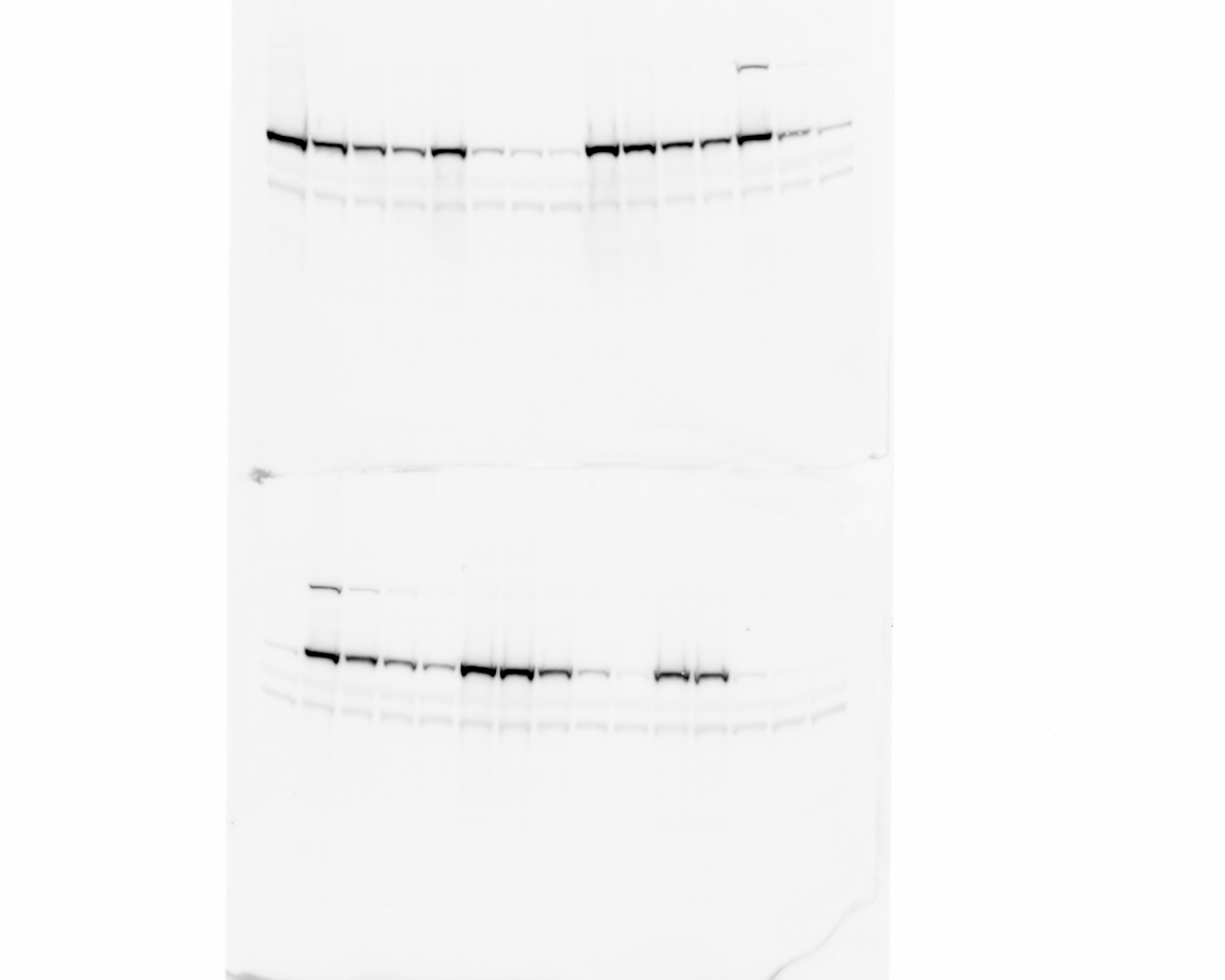

Supplement: S1 Data — (ZIP) [file pgen.1011059.s014.zip › SIdata/Figure 1 + S1A/1B+1C+1D+1E/WB RpoS chase recovery phosphate starvation/2023-02-06/RpoS.jpg]

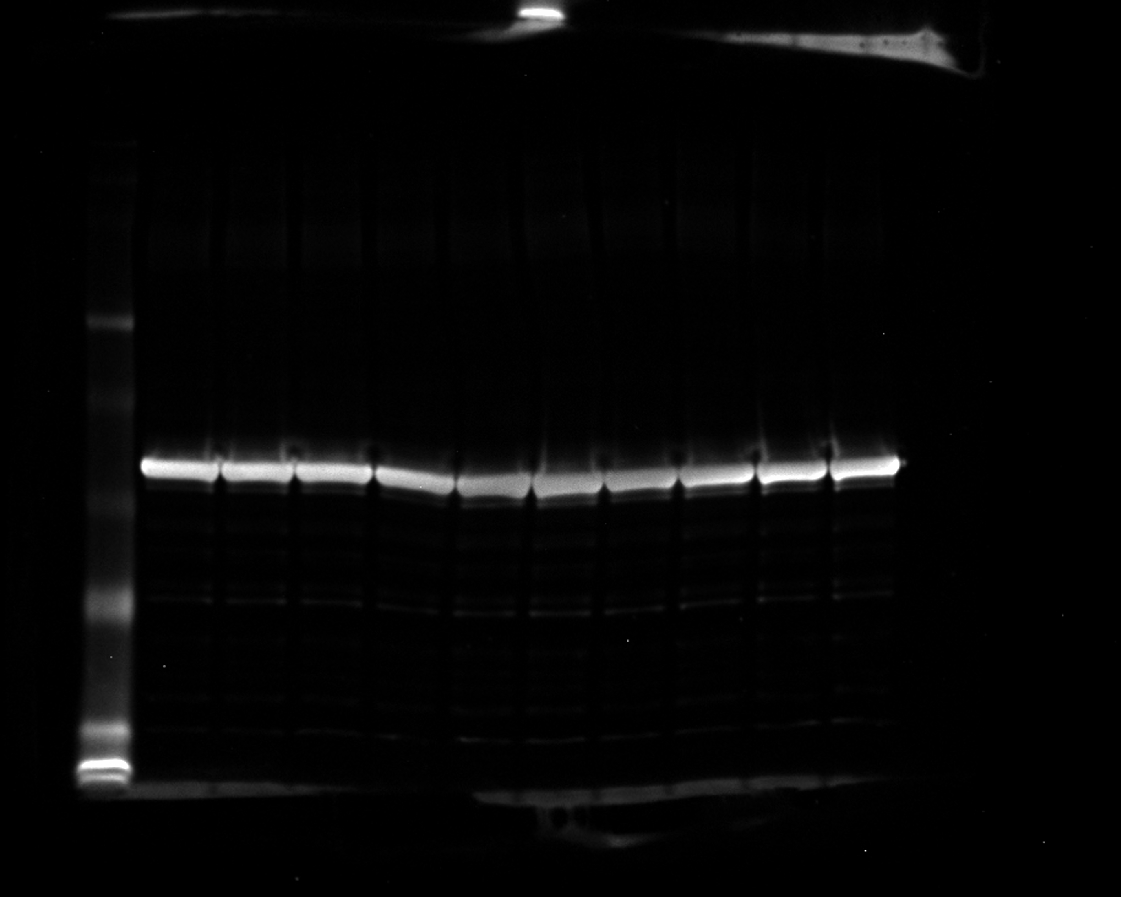

Supplement: S1 Data — (ZIP) [file pgen.1011059.s014.zip › SIdata/Figure 1 + S1A/1B+1C+1D+1E/WB RpoS chase recovery phosphate starvation/2021-04-09/lmbchemidoc 2021-04-09 17h18m30s(DyLight 800).jpg]

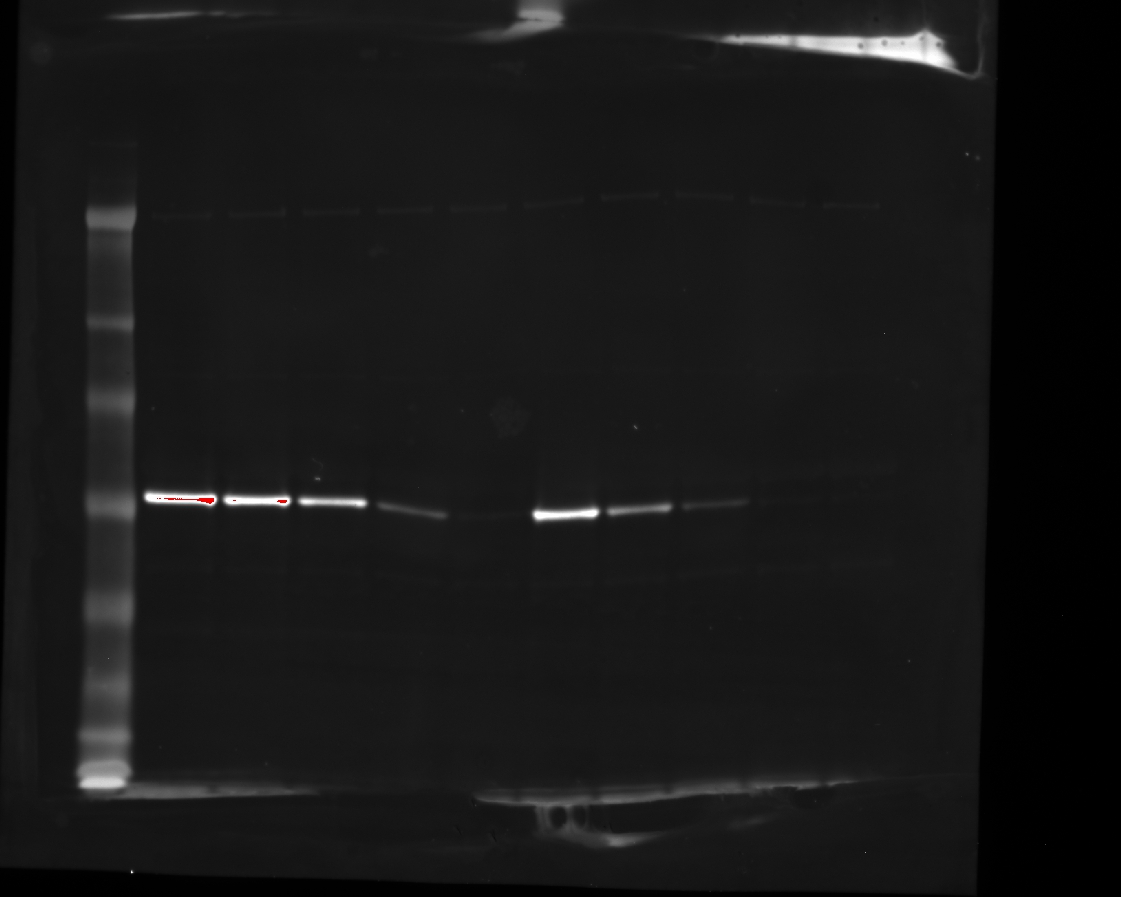

Supplement: S1 Data — (ZIP) [file pgen.1011059.s014.zip › SIdata/Figure 1 + S1A/1B+1C+1D+1E/WB RpoS chase recovery phosphate starvation/2021-04-09/lmbchemidoc 2021-04-09 17h18m30s(StarBright B700).jpg]

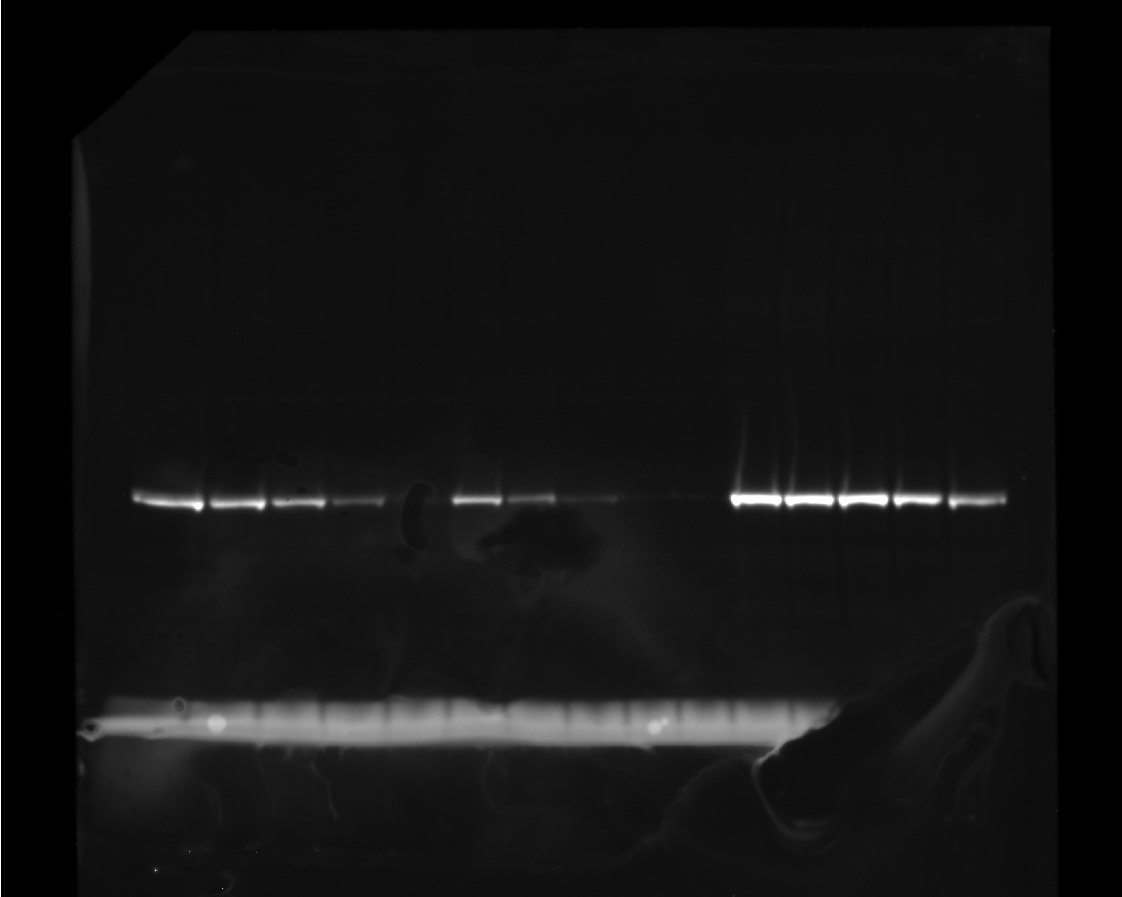

Supplement: S1 Data — (ZIP) [file pgen.1011059.s014.zip › SIdata/Figure 1 + S1A/1B+1C+1D+1E/WB RpoS chase recovery phosphate starvation/2021-04-08/lmbchemidoc 2021-04-08 13h29m35s(StarBright B700).jpg]
