# Supplementary material for: A negative feedback loop is critical for recovery of RpoS after stress in Escherichia coli
Source: PLoS Genet. 2024 Mar 11;20(3):e1011059. doi: 10.1371/journal.pgen.1011059 (PMC10957080; doi:10.1371/journal.pgen.1011059)
Supplement: S5 Fig — A) Western blot against RpoS showing RpoS degradation during recovery from phosphate starvation in the strains WT, crl-R51A (SB148) and Δcrl (SB147), primary data for Fig 5A. Experiment was performed as described in Fig 1A. B) Western blot against RpoS showing RpoS degradation after glucose starvation in the WT and Δcrl (SB147) strains, primary data for Fig 5B. Experiment was performed as described in Fig 1. C) Western blot and quantification of RpoS degradation during phosphate starvation in WT, crl-R51A (SB148) and Δcrl (SB147) strains. Experiment was performed as described in Fig 1A. D) Western blot and quantification of RpoS degradation during glucose starvation in WT and Δcrl (SB147) strains. Experiment was performed as described in Fig 1A. E) RpoS half-lives in MG1655 and Δcrl (SB147) strains during (stress) and after (recovery) phosphate starvation and glucose starvation as determined in experiments shown in Figs 5A, 5B, S5A, S5B, S5C and S5D. RpoS half-lives correspond to the time at which half of the t0 RpoS protein disappears in a chase assay. ND: Not determined. (PDF) [file pgen.1011059.s005.pdf]

Supplemental Fig S5

A Recovery from phosphate starvation (chase)

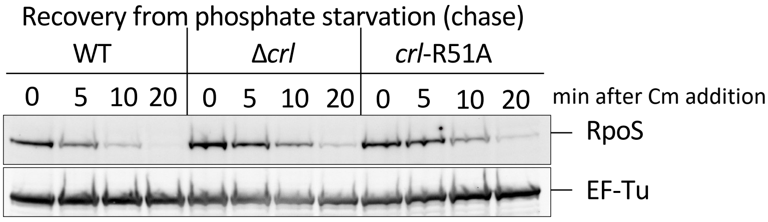

B Recovery from glucose starvation (chase)

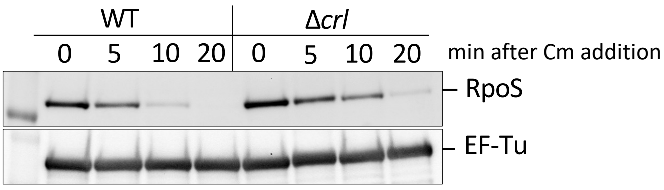

C Phosphate starvation - chase

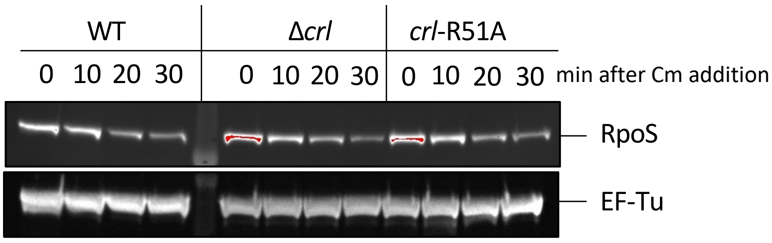

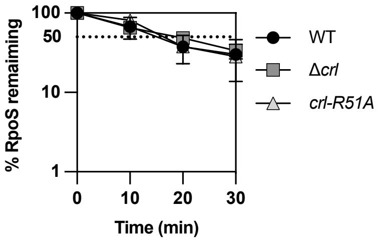

D Glucose starvation - chase

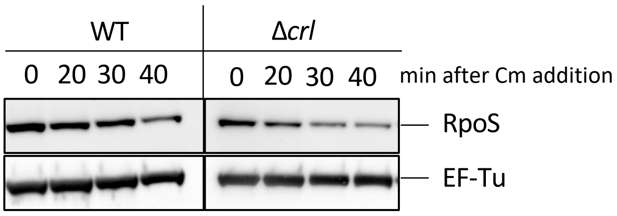

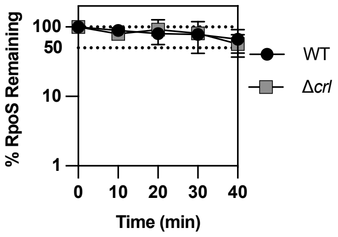

E

**RpoS half-lives (min)**	**Phosphate starvation**		**Glucose starvation**	
	Stress	Recovery	Stress	Recovery
WT	17	5.5	> 40	5.5
Δ*crl*	20	11	> 40	9
crl-R51A	17	11	ND	ND
