# Supplementary material for: A negative feedback loop is critical for recovery of RpoS after stress in Escherichia coli
Source: PLoS Genet. 2024 Mar 11;20(3):e1011059. doi: 10.1371/journal.pgen.1011059 (PMC10957080; doi:10.1371/journal.pgen.1011059)
Supplement: S1 Table — (PDF) [file pgen.1011059.s010.pdf]

Supplemental Table S1: Strains used in this study

Strains ID	Genotype	Reference
MG1655	MG1655 *crl* +	S Gottesman lab collection
BTH101	F- cya- 99 araD139 galE15 galK16 rpsL1 (StrR) hsdR2 mcrA1 mcrB1 relA1	Karimova et al, 1998
NM801	MG1655 zeo-Cp17-*rpoS* $_{750}$-*mCherry* at *lac* site	zeo-Cp17-*rpoS750* PCR fragment + NRD1166
NM802	MG1655 zeo-Cp17-*rpoS* $_{477}$-*mCherry* at *lac* site	zeo-Cp17-*rpoS477* PCR fragment + NRD1166
NM1100	MG1655 mini-λ::tet Δ*crl::IS*	MG1655 electroporated with mini-λ::tet
NRD1116	MG1655 zeo-*kan* -pBAD-*ccdB* -*mCherry* at the *lac* site *miniλ::tet*	Walling et *al* , 2022
JW0230	BW25113 Δ*crl::kan*	Keio collection; Baba et al, 2006
JW0232	BW25113 Δ*proB::kan*	Keio collection; Baba et al, 2006
JW0377	BW25113 Δ*proC::kan*	Keio collection; Baba et al, 2006
JW1252	BW25113 Δ*trpA::kan*	Keio collection; Baba et al, 2006
SG30013	MG1655 zeo-Cp17-*rpoS* $_{750}$-*lacZ* at lambda site	Zhou and Gottesman, 2006
AB006	*MG1655 ΔiraP::Kan*	Bougdour et *al* , 2006
AB011	*CRB316 rssA2::cm*	Bougdour et *al* , 2006
AB023	*MG1655 iraM::kan*	Bougdour et al, 2008
AB046	*MG1655 iraD::tet*	Bougdour et al, 2008
AB165	*MG1655 ΔrpoS::tet*	Bougdour et *al* , 2006
BA124	BTH101 *rssA2::cm*	P1 (AB011) + BTH101
BA754	*MG1655 Zeo-Cp17-rpoS+1(in nlpD)786-lacZ (9th codon)*	Majdalani et *al* , 2023
INH23	*MG1655 Δcrl::kan*	P1(JW0230) + MG1655
INH24	*MG1655 Δcrl::FRT*	pCP20 + INH23
INH26	*MG1655 Δcrl::FRT ΔiraP::Kan*	P1 (AB006) + INH24
INH28	MG1655 zeo-Cp17-*rpoS* $_{750}$-*lacZ* Δ*rpoS::tet*	P1 (AB165) + SB30013
AT59	*MG1655 Δcrl::IS*	S Gottesman lab collection
AT82	*MG1655 Δcrl::IS ΔrssB::tet*	P1 (BTH101 Δ*rssB::tet*)+AT59
AT485	NM1100 Δ*rssB-N1-168AA::kan-pBAD-Kid miniλ::tet*	Δ*rssB-N1-168AA::kan-pBAD-Kid* PCR fragment (primers AT249+AT254) + NM1100
AT596	MG1655 *rssA2::cm*	P1 (BA124) + MG1655
SB55	MG1655 Δ*crl* ::IS *zeo* -*PgadB* -mCherry at *lac* site	*PgadB* fragment PCR (primers SB14+SB15) + NRD1116
SB66	MG1655 *zeo* -*PgadB* -mCherry at *lac* site	P1 (SB55) + MG1655
SB67	MG1655 Δ*rpoS::tet* *zeo* -*PgadB* -mCherry at *lac* site	P1 (AB165) + SB66
SB71	*BTH101 ΔrpoS::kan ΔrssB::tet*	P1 (AT82) + BTH101 Δ*rpoS::kan*
SB94	*MG1655 ΔrssB::tet*	P1 (AT82) + MG1655
SB98	NM1100 Δ*crl::kan-pBAD-ccdB*	Δ*crl::kan-pBAD-ccdB* PCR fragment (primers SB24+SB39) + NM1100
SB124	MG1655 Δ*proB::kan*	P1 (JW0232)+MG1655
SB125	MG1655 Δ*proC::kan*	P1 (JW0377)+MG1655
SB137	*NM1100 crl+*	gBlock SB-03 + SB98
SB138	*NM1100 Δcrl*	gBlock SB-02 + SB98
SB139	*NM1100 crl-* R51A	gBlock SB-01 + SB98
SB147	*MG1655 Δcrl*	P1 (SB138) + SB124
SB148	*MG1655 crl-* R51A	P1 (SB139) + SB124
SB150	MG1655 zeo-Cp17-*rpoS* $_{750}$-*lacZ* Δ*rpoS::tet* *rssA2::cm*	P1 (AT596)+ INH28
SB151	*MG1655 ΔiraP*	P1 (AT596)+ INH28
SB164	MG1655 Δ*trpA::kan*	P1 (JW1252)+MG1655
SB173	MG1655 zeo-Cp17-*rpoS* $_{750}$-*lacZ* Δ*rpoS::tet* *rssA2::cm* Δ*iraP::kan*	P1 (AB006) + SB150
SB174	MG1655 zeo-Cp17-*rpoS* $_{750}$-*lacZ* Δ*rpoS::tet* *rssA2::cm* Δ*crl::kan*	P1 (INH23) + SB150
SB175	MG1655 zeo-Cp17-*rpoS* $_{750}$-*lacZ* Δ*rpoS::tet* Δ*iraP::kan*	P1 (AB006) + INH28
SB176	MG1655 zeo-Cp17-*rpoS* $_{750}$-*lacZ* Δ*rpoS::tet* Δ*crl::kan*	P1 (INH23) + INH28
SB179	MG1655 zeo-Cp17-*rpoS* $_{750}$-*lacZ* Δ*iraP::kan*	P1 (AB006) + SG30013
SB180	MG1655 zeo-Cp17-*rpoS* $_{750}$-*lacZ* Δ*crl::kan*	P1 (INH23) + SG30013
SB190	*MG1655 rssB-* D58A	gBlock SB-10 + AT485
SB192	*MG1655 rssB-* D58E	gBlock SB-9 + AT485
SB198	*MG1655 rssB* -D58P	gBlock SB-8 + AT485

Supplemental Table S1: Strains used in this study (continued)

Strains ID	Genotype	Reference
SB212	*MG1655 iraP-SPA*	Battesti et *al* , 2015
SB225	MG1655 zeo-Cp17-*rpoS* $_{750}$*-mCherry* Δ*rssB::tet*	P1 (AT82) + NM801
SB226	MG1655 zeo-Cp17-*rpoS* $_{750}$*-mCherry rssA2::cm*	P1(AT596) + NM801
SB228	MG1655 zeo-Cp17-*rpoS* $_{750}$*-mCherry* Δ*crl::kan*	P1 (INH23) + NM801
SB230	*MG1655 zeo-Cp17-rpoS* $_{750}$*-mCherry* Δ*proB::kan*	P1(JW0232) + NM801
SB238	MG1655 zeo-Cp17-*rpoS* $_{750}$*-mCherry* Δ*rpoS::tet*	P1 (AB165) + NM801
SB239	MG1655 zeo-Cp17-*rpoS* $_{750}$*-mCherry* Δ*rpoS::tet* Δ*crl::kan*	P1 (INH23) + SB238
SB242	MG1655 Δ*rssB::tet zeo* -P*gadB* -mCherry at *lac* site	P1 (AT82) + SB66
SB243	MG1655 Δ*crl::kan zeo* -P*gadB* -mCherry at *lac* site	P1 (INH23) + SB66
SB280	MG1655 zeo-Cp17-*rpoS* $_{750}$*-mCherry* Δ*rpoS::tet* Δ*crl::kan rssA2::cm*	P1(AT596) + SB239
SB281	MG1655 zeo-Cp17-*rpoS* $_{750}$*-mCherry* Δ*rpoS::tet rssA2::cm*	P1(AT596) + SB238
SB282	MG1655 zeo-Cp17-*rpoS* $_{750}$*-mCherry* Δ*crl::kan rssA2::cm*	P1(AT596) + SB228
SB283	MG1655 zeo-Cp17-*rpoS* $_{750}$*-mCherry* Δ*rssB::tet* Δ*crl::kan*	P1 (INH23) + SB225
SB296	MG1655 Δ*rpoS::tet* Δ*crl::kan zeo* -P*gadB* -mCherry at *lac* site	P1 (INH23) + SB67
SB297	MG1655 Δ*rssB::tet* Δ*crl::kan zeo* -P*gadB* -mCherry at *lac* site	P1 (INH23) + SB242
SB298	MG1655 Δ*rssB::tet* Δ*rpoS::kan zeo* -P*gadB* -mCherry at *lac* site	P1 (Δ*rpoS::kan*) + SB242
SB364	*MG1655 iraD::tet*	P1 (AB046) + MG1655
SB365	*MG1655 iraD::tet* Δ*iraP*	P1 (AB046) + SB151
SB341	*MG1655 zeo-Cp17-rpoS* $_{750}$*-mCherry crl* -R51A	P1(SB148) + SB230
SB437	MG1655 zeo-Cp17-*rpoS* $_{477}$*-mCherry* Δ*crl::kan*	P1(INH23) + NM802
SB439	MG1655 zeo-Cp17-*rpoS* $_{477}$*-mCherry* Δ*rpoS::tet*	P1(AB165) + NM802
SB470	MG1655 Δ*ssrS::kan*	Storz lab collection
SB497	NM1100 Δ*rsd::kan-pBAD-ccdB* Δ*crl:* :IS	Δ*rsd::kan-pBAD-ccdB* fragment PCR (primers SB196+SB197) + NM1100
SB498	NM1100 Δ*rsd* Δ*crl* ::IS	gBlock SB-47+ SB497
SB503	NM1100 Δ*rsd* Δ*crl* ::IS Δ*proB::kan*	P1 (JW0232) + SB498
SB505	MG1655 Δ*rsd* crl+	P1 (SB137) + SB503
SB539	*MG1655* Δ*iraM::kan*	P1 (AB023) + MG1655
SB540	*MG1655* Δ*iraP* Δ*iraM::kan*	P1 (AB023) + SB151
SB541	*MG1655* Δ*iraD::tet* Δ*iraM::kan*	P1 (AB023) + SB364
SB542	*MG1655* Δ*iraP* Δ*iraD::tet* Δ*iraM::kan*	P1 (AB023) + SB365
