# Supplementary material for: A negative feedback loop is critical for recovery of RpoS after stress in Escherichia coli
Source: PLoS Genet. 2024 Mar 11;20(3):e1011059. doi: 10.1371/journal.pgen.1011059 (PMC10957080; doi:10.1371/journal.pgen.1011059)
Supplement: S2 Table — (PDF) [file pgen.1011059.s011.pdf]

Supplemental Table S2: Plasmids used in this study

Vectors ID	Description	Reference
pSIM6	mini**λ**::*amp*	Hashimoto-Gotoh et al, 1981
pUT18linker	empty pUT18	Battesti et al, 2012
pKT25linker	empty pKT25	Battesti et al, 2012
pQE80L	empty pQE80L	Qiagen
pSB8	pUT18-*crl*	This work
pSB19	pQE80L-mCherry	This work
pSB21	pQE80L-*yodD* p-mCherry	This work
pSB22	pQE80L-*osmY* p-mCherry	This work
pSB23	pQE80L-*gadB* p-mCherry	This work
pSB26	pUT18-*crl* -R51A	This work
pSB34	pKT25-*rpoS*	Battesti et al, 2013
pSB37	pQE80L-*rssAB* p-mCherry	This work
pSB38	pQE80L-*osmE* p-mCherry	This work
pSB48	pQE80L-*rssB* p-mCherry	This work
