# Supplementary material for: A negative feedback loop is critical for recovery of RpoS after stress in Escherichia coli
Source: PLoS Genet. 2024 Mar 11;20(3):e1011059. doi: 10.1371/journal.pgen.1011059 (PMC10957080; doi:10.1371/journal.pgen.1011059)
Supplement: S3 Table — (PDF) [file pgen.1011059.s012.pdf]

Supplemental Table S3

Primers ID	Description (method)	Sequences 5' - 3'
SB-1	Forward *crl* -R51A (quickchange)	GCACCGGAAGTGGCCGAATTCTGGGGC
SB-2	Reverse *crl* -R51A (quickchange)	GCCCCAGAATTCGGCCACTTCCGGTGC
SB-14	Forward P*gadB* (Transformation into NRD1116 strain)	ATACTATGCCGATATACTATGCCGATGATTAATTGTCAACCATATTGTATTGTTTTTCAA
SB-15	Reverse P*gadB* (Transformation into NRD1116 strain / Gibson into pQE80L-mCherry in combination with SB-33 primer)	tgatgatggccatgttatcctcctcgcccttgctcaccatagctgACCTCCTAAAATGATTGGATCGCAT
SB-24	Forward *crl::kan-pBAD-ccdB* (transformation into NM1100 strain)	CCAATTTGGTAAAACAGTTGCATCACAACAGGAGATAGCATTAGAAGAACTCGTCAAGAA
SB-39	Reverse *crl::kan-pBAD-ccdB* (transformation into NM1100 strain)	AATGAATATTGCCGGATGTGATGCATCCGGCACATTTCACttatattccccagaacatca
SB-28	Reverse pQE80L (Gibson with mCherry)	TTTCTCGAGGTGAAGACGAAAGGGCCTCGTGATAC
SB-29	Forward pQE80L (Gibson with mCherry)	CTTGGACTCCTGTTGATAGATCCAGTAATGACCTC
SB-30	Forward mCherry (Gibson with pQE80L)	CTTCACCTCGAGAAAAGGAGGTcagctATGGTGAGCAAGGGCGAGGA
SB-31	Reverse mCherry (Gibson with pQE80L)	CAACAGGAGTCCAAGCTACTTGTACAGCTCGTCCA
SB-33	Forward P*gadB* (Gibson)	CTTCACCTCGAGAAACATATTGTATTGTTTTTCAA
SB-34	Forward P*osmY* (Gibson)	CTTCACCTCGAGAAATATGTTTTCGCTGATATCCC
SB-17	Reverse P*osmY* (Gibson)	tgatgatggccatgttatcctcctcgcccttgctcaccatagctgACCTCCTGTATGTTTGCTCGTAATT
SB-37	Forward P*yodD* (Gibson)	CTTCACCTCGAGAAACGTTTTCCTCCTGTGGCTTT
SB-196	Reverse P*yodD* (Gibson)	tgatgatggccatgttatcctcctcgcccttgctcaccatagctgACCTCCTCAACCGTTACTCTGTTGT
SB-197	Forward P*osmE* (Gibson)	CTTCACCTCGAGAAATGAACCCCTCCAGACAGAAA
SB-47	Reverse P*osmE* (Gibson)	CATagctgACCTCCTATTCCGTCCTCTTGTTTATC
SB-38	Forward promoters (Gibson with pQE80L-mCherry)	AGGAGGTcagctATGGTGAGCAAGGGCGAGGAGGA
SB-43	Forward P*rssB* (Gibson with pQE80L-mCherry)	AGGAGGTcagctATGCGTTGGAGTGAATGCATTGC
SB-44	Reverse P*rssB* (Gibson with pQE80L-mCherry)	CTCGCCCTTGCTCACTTTTCCGACCAATGGCTGCG
SB-45	Reverse pQE80L-mCherry (Gibson with P*rssB*)	CATagctgACCTCCTTTTCTCGAGGTGAAGACGAA
SB-66	Forward pQE80L-mCherry (Gibson with P*rssB*)	GTGAGCAAGGGCGAGGAGGATAACATGGCCATCAT
SB-72	Forward pQE80L inserts (sequencing)	CTTTCGTCTTCACCTCGAG
SB-74	Reverse pQE80L inserts (sequencing)	CCTCCTCGCCCTTGCTC
SB-92	Reverse pSB37 (Gibson with GB-SB005 = ΔP2 P*rssB*)	CCGGATTTGCGCATACTGAATGGTTGGGATTGACC
SB-93	Forward pSB37 (Gibson with GB-SB005 = ΔP2 P*rssB*)	GAGAAAGATAAAAATAGGGCTGGCGCTGGGATCTG
SB-94	Reverse pSB37 (Gibson with GB-SB006 = ΔP1 P*rssB*)	GATATTTGCGGGCAAACAGGTTGAATCAGAATATC
SB-95	Forward pSB37 (Gibson with GB-SB006 = ΔP1 P*rssB*)	CGAGATCGAGGGCGAGGGCGAGGGCCGCCCCTACG
SB-196	Forward *rsd::kan-pBad-ccdB* (transformation into NM1100)	TCACTGAGCAGTTTTTGAATACAAACTTGCGGAGTCAATCttagaagaactcgtcaagaa
SB-197	Reverse *rsd::kan-pBad-ccdB* (transformation into NM1100)	TGGGGCATTGAATGTAAATTACGCGTTAACAGCGCAGAACttatattccccagaacatca
AT249	Forward Δ*rssB-N1-168AA::kan-pBAD-Kid* (Transformation into NM1100)	AGCATGCCACTATTGAGTAAAGCCAGTCAGGGGAGAGAACTTAGAAGAACTCGTCAAGAA
AT254	Reverse Δ*rssB-N1-168AA::kan-pBAD-Kid* (Transformation into NM1100)	CCGCGGCAACCAATTGACGATAATTAACCCGGCAATGGGATCAAGTCAGAATAGTGGACA
RpoS750-mCherNRD1166F	Forward *zeo-Cp17-rpoS750* (Transformation into NRD1166)	ATA CTA TGC CGA TAT ACT ATG CCG ATG ATT AAT TGT CAA CCA TTC TGG AGT TTA TTC TT
RpoS750-mCherNRD1166R	Reverse *zeo-Cp17-rpoS750* (Transformation into NRD1166)	CCT TGA TGA TGG CCA TGT TAT CCT CCT CGC CCT TGC TCA CCG TGG TAT CTT CCG GAC CGT
zeo-F	Forward *zeo* (Sequencing)	CTCCACGAAGTCCCGGGAGA
Int-mCh-sequencing primer	Reverse *mCherry* (Sequencing)	CTTGGTCACCTTCAGCTTGG
