# Supplementary material for: A negative feedback loop is critical for recovery of RpoS after stress in Escherichia coli
Source: PLoS Genet. 2024 Mar 11;20(3):e1011059. doi: 10.1371/journal.pgen.1011059 (PMC10957080; doi:10.1371/journal.pgen.1011059)
Supplement: S4 Table — (PDF) [file pgen.1011059.s013.pdf]

Supplemental Table S4

gBlocks ID	Description	Sequences 5' - 3'
SB-GB001	integrate crl with R51A mutation in the chromosome, in place of the kan-pBad-kid cassette at the crl locus	TCTTCAGGAAATCACCGACTGGATCGAAAAACGCTTGTGTTAAAAATTTGCTAAAT<br>TTTGCCAATTTGGTAAAACAGTTGCATCACAACAGGAGATAGCAatgACGTTACCG<br>AGTGGACACCCGAAGAGCAGATTGATCAAAAAATTTACCGCACTAGGCCCGTATA<br>TTCGTGAAGGTAAGTGCAAAGATAATCGATTCTTTTTCGATTGTCTGGCTGTATGC<br>GTCAACGTGAAACCGGCACCGGAAGTGgccGAATTCTGGGGCTGGTGGATGGAG<br>CTGGAAGCGCAGGAATCCCGTTTTACCTACAGTTACCAGTTTGGTCTGTTCGATAA<br>AGCAGGCGACTGGAAGAGTGTTCCGGTAAAAGACACTGAAGTGGTTGAACGACT<br>GGAGCACACCCTGCGTGAGTTTCACGAGAAGCTGCGTGAACTGCTGACGACGCT<br>GAATCTGAAGCTGGAACCGGCGGATGATTTTCGTGACGAGCCGGTGAAGTTAAC<br>GGCGtgaGTGAAATGTGCCGGATGCATCACATCCGGCAATATTCATTAAAACTGAT<br>ACGTCATGCCAACCGCGACAATATCATCATTATTAATATTCAATTTGTTAT
SB-GB002	integrate Δcrl in the chromosome, in place of the kan-pBad-kid cassette at the crl locus	TCTTCAGGAAATCACCGACTGGATCGAAAAACGCTTGTGTTAAAAATTTGCTAAAT<br>TTTGCCAATTTGGTAAAACAGTTGCATCACAACAGGAGATAGCAGTGAAATGTGC<br>CGGATGCATCACATCCGGCAATATTCATTAAAACTGATACGTCATGCCAACCGCGA<br>CAATATCATCATTATTAATATTCAATTTGTTAT
SB-GB003	integrate crl in the chromosome, in place of the kan-pBad-kid cassette at the crl locus	TCTTCAGGAAATCACCGACTGGATCGAAAAACGCTTGTGTTAAAAATTTGCTAAAT<br>TTTGCCAATTTGGTAAAACAGTTGCATCACAACAGGAGATAGCAatgACGTTACCG<br>AGTGGACACCCGAAGAGCAGATTGATCAAAAAATTTACCGCACTAGGCCCGTATA<br>TTCGTGAAGGTAAGTGCAAAGATAATCGATTCTTTTTCGATTGTCTGGCTGTATGC<br>GTCAACGTGAAACCGGCACCGGAAGTGCGTGAATTCTGGGGCTGGTGGATGGAG<br>CTGGAAGCGCAGGAATCCCGTTTTACCTACAGTTACCAGTTTGGTCTGTTCGATAA<br>AGCAGGCGACTGGAAGAGTGTTCCGGTAAAAGACACTGAAGTGGTTGAACGACT<br>GGAGCACACCCTGCGTGAGTTTCACGAGAAGCTGCGTGAACTGCTGACGACGCT<br>GAATCTGAAGCTGGAACCGGCGGATGATTTTCGTGACGAGCCGGTGAAGTTAAC<br>GGCGtgaGTGAAATGTGCCGGATGCATCACATCCGGCAATATTCATTAAAACTGAT<br>ACGTCATGCCAACCGCGACAATATCATCATTATTAATATTCAATTTGTTAT
SB-GB005	ΔP2 PrssB. To be used in Gibson assembly with primers SB-92+93 on pSB37	TTCAGTATGCGCAAATCCGGCCATCAATTCGGCACGTAACGCCGCTGCTCCACAA<br>GAGGGATGCCAGGTCTTTATTAAATAATCTGCGTCTTGCATCACAAAAGCGCAGT<br>AACGCGAACGCATGAGATGTTCTGGATCAGGTGCAACCTTTTCACCAGacTACCAC<br>AGGGACAAAGCTGAGACACAAATAATCTCCCTGGAAACAATAACGGCGTATTAAC<br>CGCCTGAGTAGCACTATGTTAACCGAGCAGTAGCGATGTGGCTACGATTGCATTC<br>CAGGGGAATCTTGCGGGAATAATGAGAAAGATAAAAATAGGGC
SB-GB006	ΔP1 PrssB. To be used in Gibson assembly with primers SB-94+95 on pSB37	CCTGTTTGCCCGCAAATATCTACGCTTGATTTCCATCGCGCGCACGCTGCCATTGC<br>GGCCGGACAGCTGGCAGTGGAAAGGAAAATGGACGAACTTTTGCCGTTGGTACG<br>CACCAACATTTGACCAGAATTTTTATCTACACTTAAGTTAATTCTGaaAGCCAGTCA<br>GGGGAGAGAACatgACGCAGCCATTGGTCGGAAAAGTGAGCAAGGGCGAGGAG<br>GATAACATGGCCATCATCAAGGAGTTCATGCGCTTCAAGGTGCACATGGAGGGCT<br>CCGTGAACGGCCACGAGTTCGAGATCGAGGGCGAGGGCG
SB-GB008	integrate RssB D58P into NM1000 rssB N1-168 AA::kanKid (strain #AT485)	CTACACTTAAGTTAATTCTGACAGGCGCAGGTGGCAATAGCATGCCACTATTGAG<br>TAAAGCCAGTCAGGGGAGAGAACatgACGCAGCCATTGGTCGGAAAACAGATTCT<br>CATTGTTGAAGATGAGCAGGTATTTCGCTCGCTTCTGGATTCATGGTTTTCCTCATT<br>GGGAGCGACAACGGTACTGGCGGCTGATGGGGTGGATGCCCTTGAGTTGCTGGG<br>AGGTTTCACTCCAGACCTGATGATATGTccgATCGCGATGCCACGAATGAACGGGC<br>TTAAACTGCTGGAGCATATACGTAACAGAGGCGACCAGACCCCAGTTCTGGTGAT<br>ATCTGCCACTGAAAATATGGCAGATATTGCCAAAGCGTTACGTCTGGGCGTTGAA<br>GATGTTTTGCTGAAACCAGTTAAAGATCTGAATCGCTTGCGCGAGATGGTTTTTGC<br>CTGTCTCTATCCCAGCATGTTTAATTCGCGCGTTGAGGAAGAGGAAAGGCTTTTTC<br>GCGACTGGGATGCAATGGTTGATAACCCTGCCGCAGCGGCGAAATTATTACAGG<br>AACTACAACCGCCGGTTCAGCAGGTGATTTCCCATTGCCGGGTTAATTATCGTCAA<br>TTGGTTGCCGCGGACAAACCCGGCCTGGTGCTTGATATTGCCGCACTTTCGG
SB-GB009	integrate RssB D58E into NM1000 rssB N1-168 AA::kanKid (strain #AT485)	CTACACTTAAGTTAATTCTGACAGGCGCAGGTGGCAATAGCATGCCACTATTGAG<br>TAAAGCCAGTCAGGGGAGAGAACatgACGCAGCCATTGGTCGGAAAACAGATTCT<br>CATTGTTGAAGATGAGCAGGTATTTCGCTCGCTTCTGGATTCATGGTTTTCCTCATT<br>GGGAGCGACAACGGTACTGGCGGCTGATGGGGTGGATGCCCTTGAGTTGCTGGG<br>AGGTTTCACTCCAGACCTGATGATATGTgaaATCGCGATGCCACGAATGAACGGG<br>CTTAAACTGCTGGAGCATATACGTAACAGAGGCGACCAGACCCCAGTTCTGGTGA<br>TATCTGCCACTGAAAATATGGCAGATATTGCCAAAGCGTTACGTCTGGGCGTTGA<br>AGATGTTTTGCTGAAACCAGTTAAAGATCTGAATCGCTTGCGCGAGATGGTTTTTG<br>CCTGTCTCTATCCCAGCATGTTTAATTCGCGCGTTGAGGAAGAGGAAAGGCTTTTT<br>CGCGACTGGGATGCAATGGTTGATAACCCTGCCGCAGCGGCGAAATTATTACAGG<br>AACTACAACCGCCGGTTCAGCAGGTGATTTCCCATTGCCGGGTTAATTATCGTCAA<br>TTGGTTGCCGCGGACAAACCCGGCCTGGTGCTTGATATTGCCGCACTTTCGG
SB-GB010	integrate RssB D58A into NM1000 rssB N1-168 AA::kanKid (strain #AT485)	CTACACTTAAGTTAATTCTGACAGGCGCAGGTGGCAATAGCATGCCACTATTGAG<br>TAAAGCCAGTCAGGGGAGAGAACatgACGCAGCCATTGGTCGGAAAACAGATTCT<br>CATTGTTGAAGATGAGCAGGTATTTCGCTCGCTTCTGGATTCATGGTTTTCCTCATT<br>GGGAGCGACAACGGTACTGGCGGCTGATGGGGTGGATGCCCTTGAGTTGCTGGG<br>AGGTTTCACTCCAGACCTGATGATATGTgccATCGCGATGCCACGAATGAACGGGC<br>TTAAACTGCTGGAGCATATACGTAACAGAGGCGACCAGACCCCAGTTCTGGTGAT<br>ATCTGCCACTGAAAATATGGCAGATATTGCCAAAGCGTTACGTCTGGGCGTTGAA<br>GATGTTTTGCTGAAACCAGTTAAAGATCTGAATCGCTTGCGCGAGATGGTTTTTGC<br>CTGTCTCTATCCCAGCATGTTTAATTCGCGCGTTGAGGAAGAGGAAAGGCTTTTTC<br>GCGACTGGGATGCAATGGTTGATAACCCTGCCGCAGCGGCGAAATTATTACAGG<br>AACTACAACCGCCGGTTCAGCAGGTGATTTCCCATTGCCGGGTTAATTATCGTCAA<br>TTGGTTGCCGCGGACAAACCCGGCCTGGTGCTTGATATTGCCGCACTTTCGG
SB-GB047	integrate Δrsd in the chromosome	ATCCATAGCTCTTGCACTACCTTTGCATCACTGGCATGTTTAACATGGTTTTTACAT<br>TTCTCACTGAGCAGTTTTTGAATACAAACTTGCGGAGTCAATCGTTCTGCGCTGTT<br>AACGCGTAATTTACATTCAATGCCCCATTTGCGGGGCTAATTTCTTGTCGGAGTGC<br>CTTAACTGGCTGAGACCGTTTATTCGGGATC
