# Supplementary figures and images for: A negative feedback loop is critical for recovery of RpoS after stress in Escherichia coli (part 2 of 2)
Source: PLoS Genet. 2024 Mar 11;20(3):e1011059. doi: 10.1371/journal.pgen.1011059 (PMC10957080; doi:10.1371/journal.pgen.1011059)

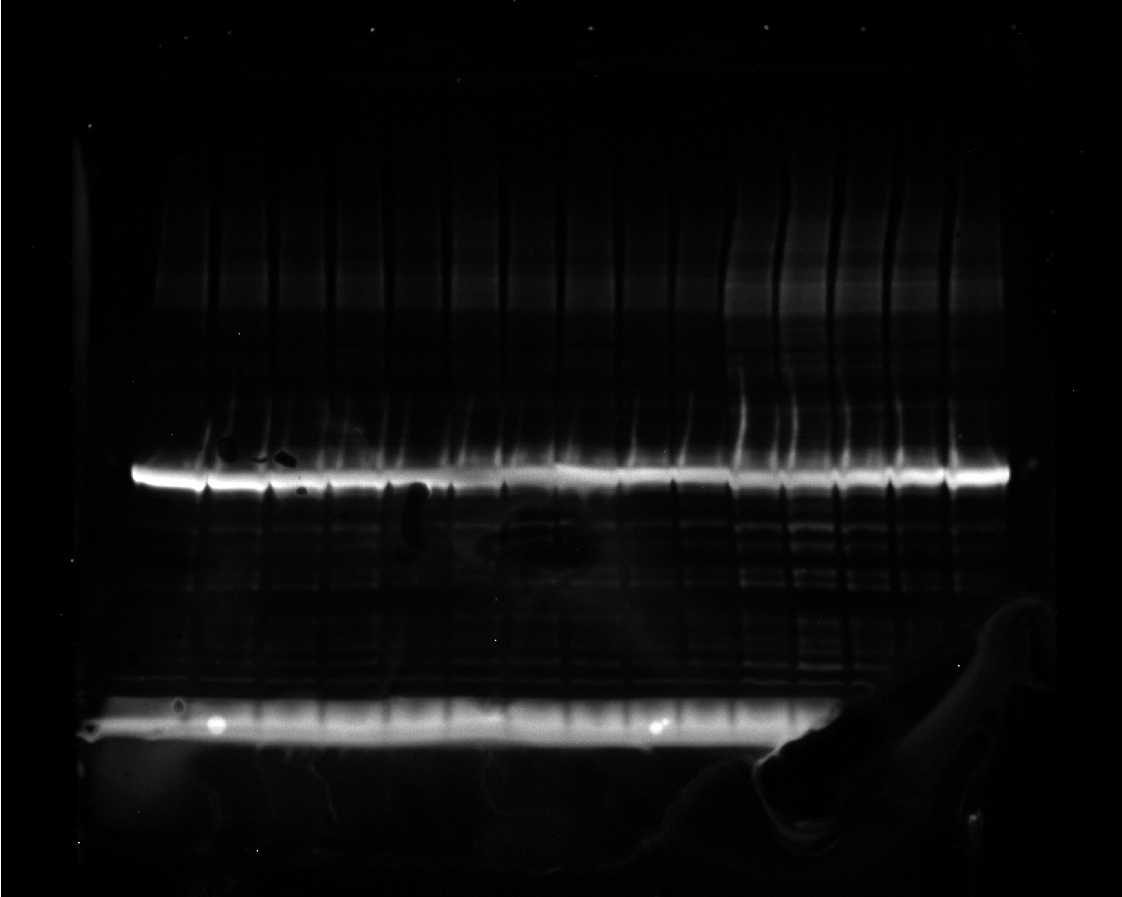

Supplement: S1 Data — (ZIP) [file pgen.1011059.s014.zip › SIdata/Figure 1 + S1A/1B+1C+1D+1E/WB RpoS chase recovery phosphate starvation/2021-04-08/lmbchemidoc 2021-04-08 13h30m29s(DyLight 800).jpg]

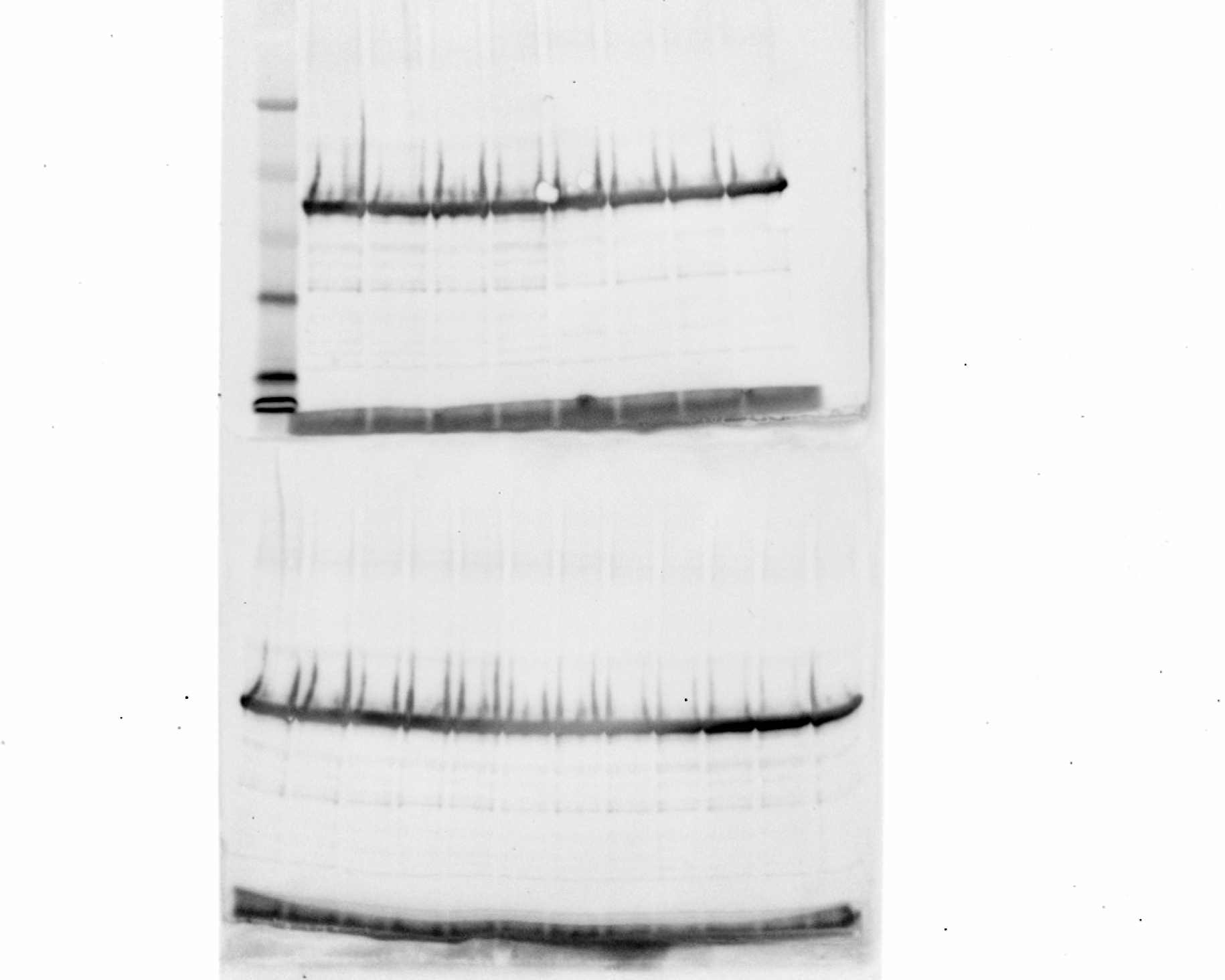

Supplement: S1 Data — (ZIP) [file pgen.1011059.s014.zip › SIdata/Figure 1 + S1A/1B+1C+1D+1E/WB RpoS chase phosphate starvation/2023-02-02/EFTu.jpg]

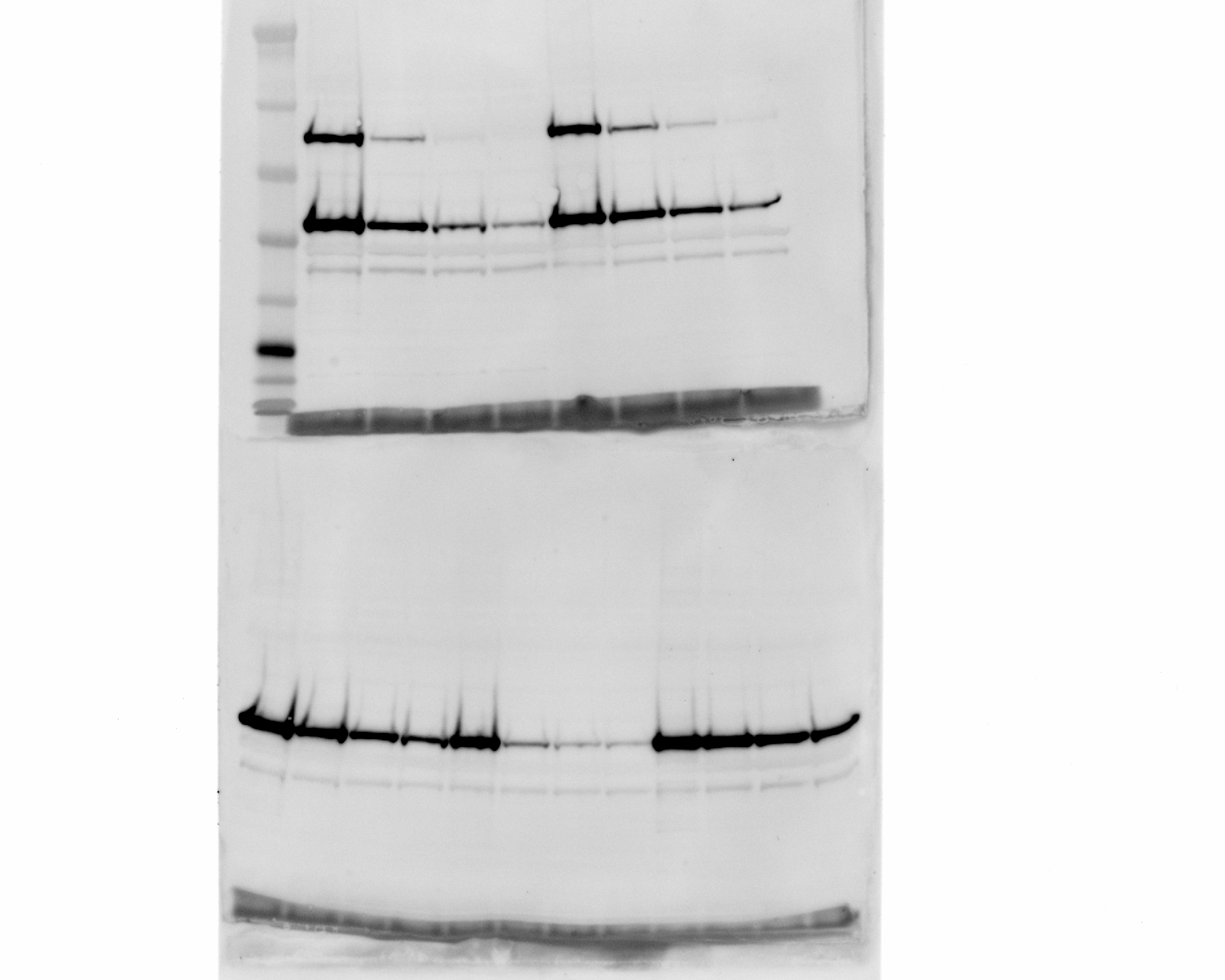

Supplement: S1 Data — (ZIP) [file pgen.1011059.s014.zip › SIdata/Figure 1 + S1A/1B+1C+1D+1E/WB RpoS chase phosphate starvation/2023-02-02/RpoS.jpg]

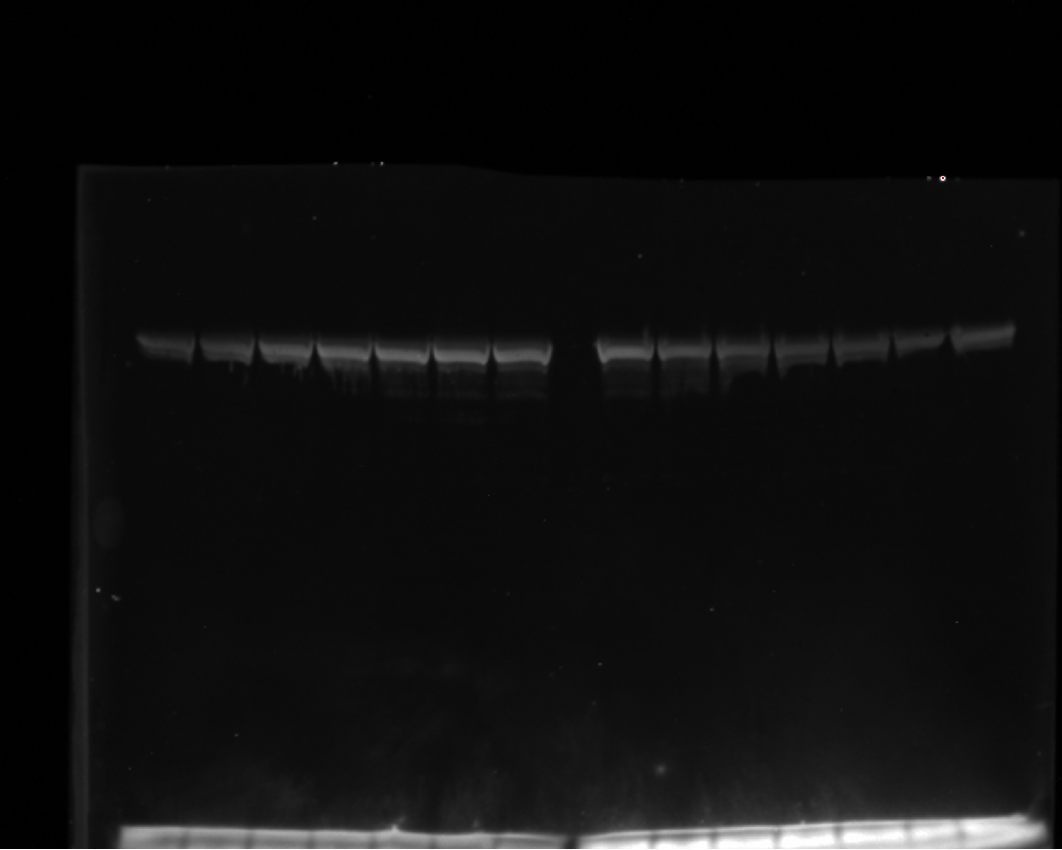

Supplement: S1 Data — (ZIP) [file pgen.1011059.s014.zip › SIdata/Figure 1 + S1A/S1A + part of 1D - RpoS no chase phosphate starvation/WB RpoS no chase phosphate starvation/Set 2/GelB EFTu.tif]

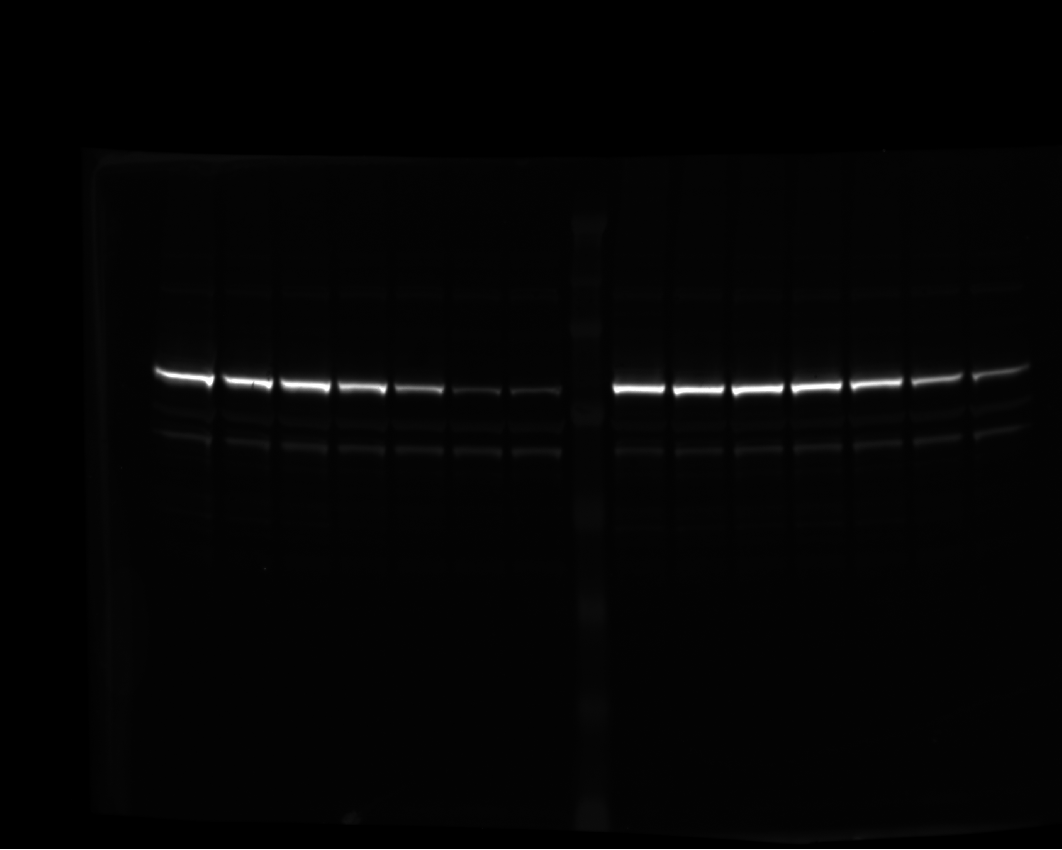

Supplement: S1 Data — (ZIP) [file pgen.1011059.s014.zip › SIdata/Figure 1 + S1A/S1A + part of 1D - RpoS no chase phosphate starvation/WB RpoS no chase phosphate starvation/Set 2/GelA RpoS.tif]

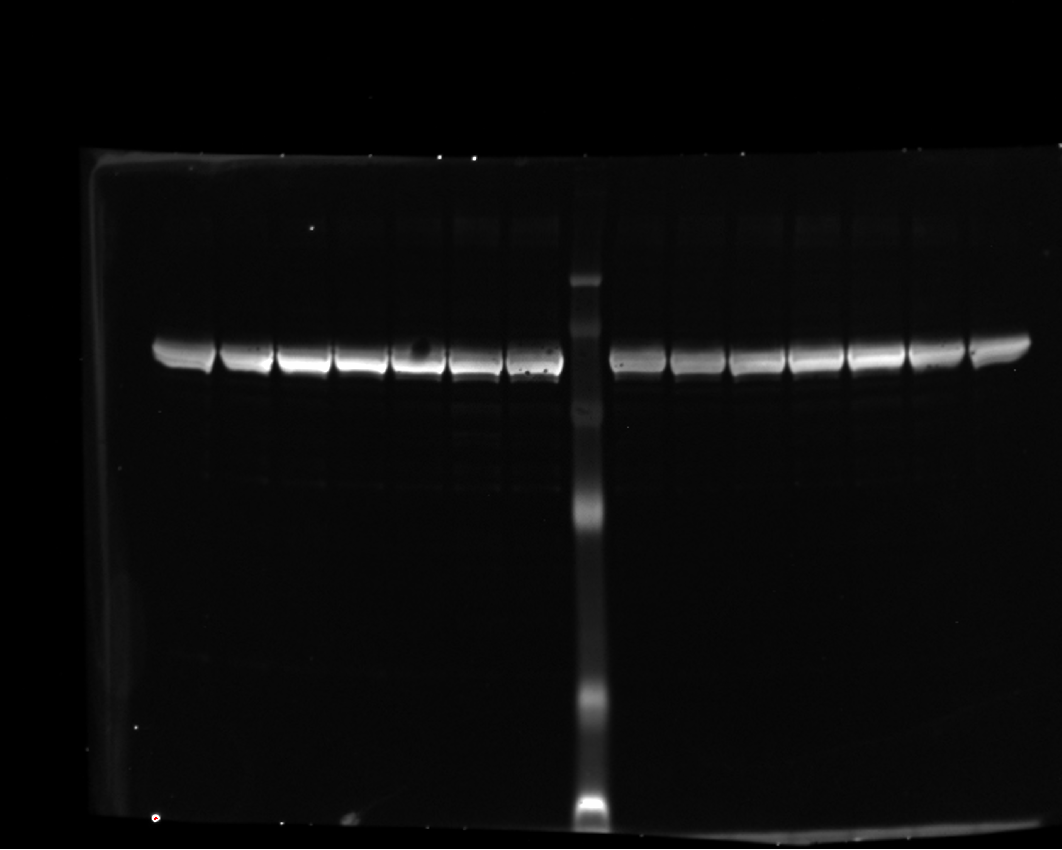

Supplement: S1 Data — (ZIP) [file pgen.1011059.s014.zip › SIdata/Figure 1 + S1A/S1A + part of 1D - RpoS no chase phosphate starvation/WB RpoS no chase phosphate starvation/Set 2/GelA EFTu.tif]

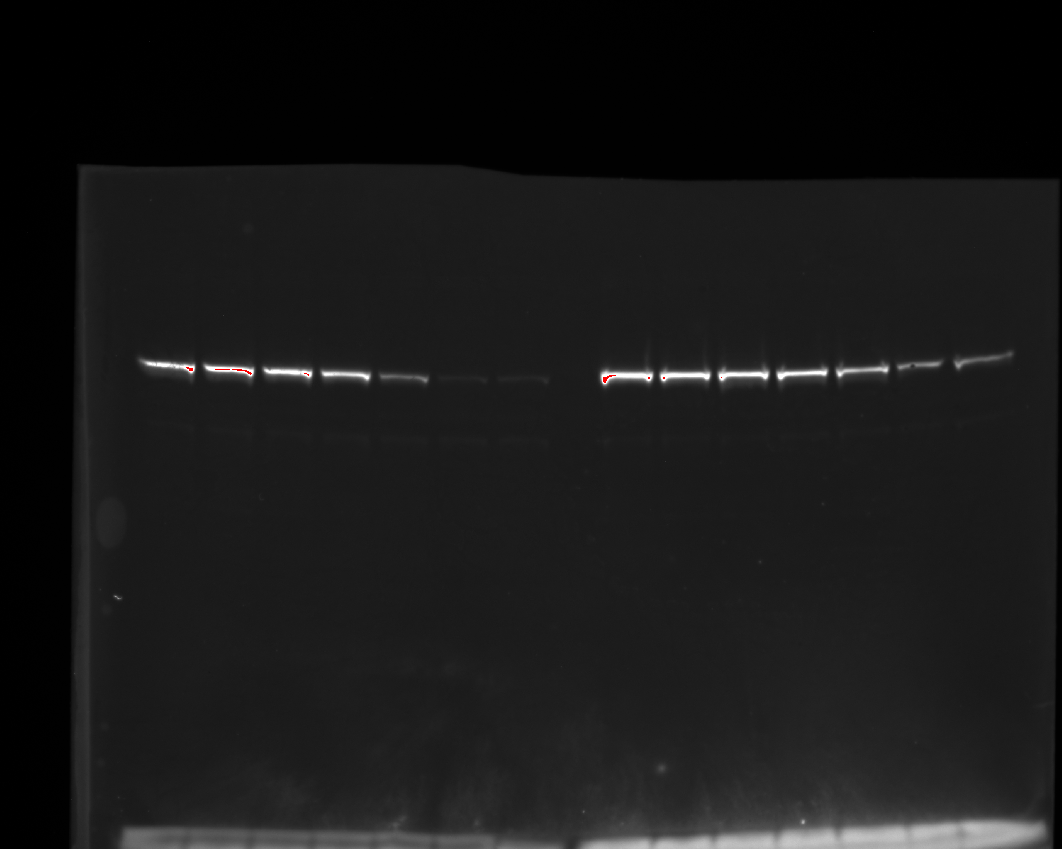

Supplement: S1 Data — (ZIP) [file pgen.1011059.s014.zip › SIdata/Figure 1 + S1A/S1A + part of 1D - RpoS no chase phosphate starvation/WB RpoS no chase phosphate starvation/Set 2/GelB RpoS.tif]

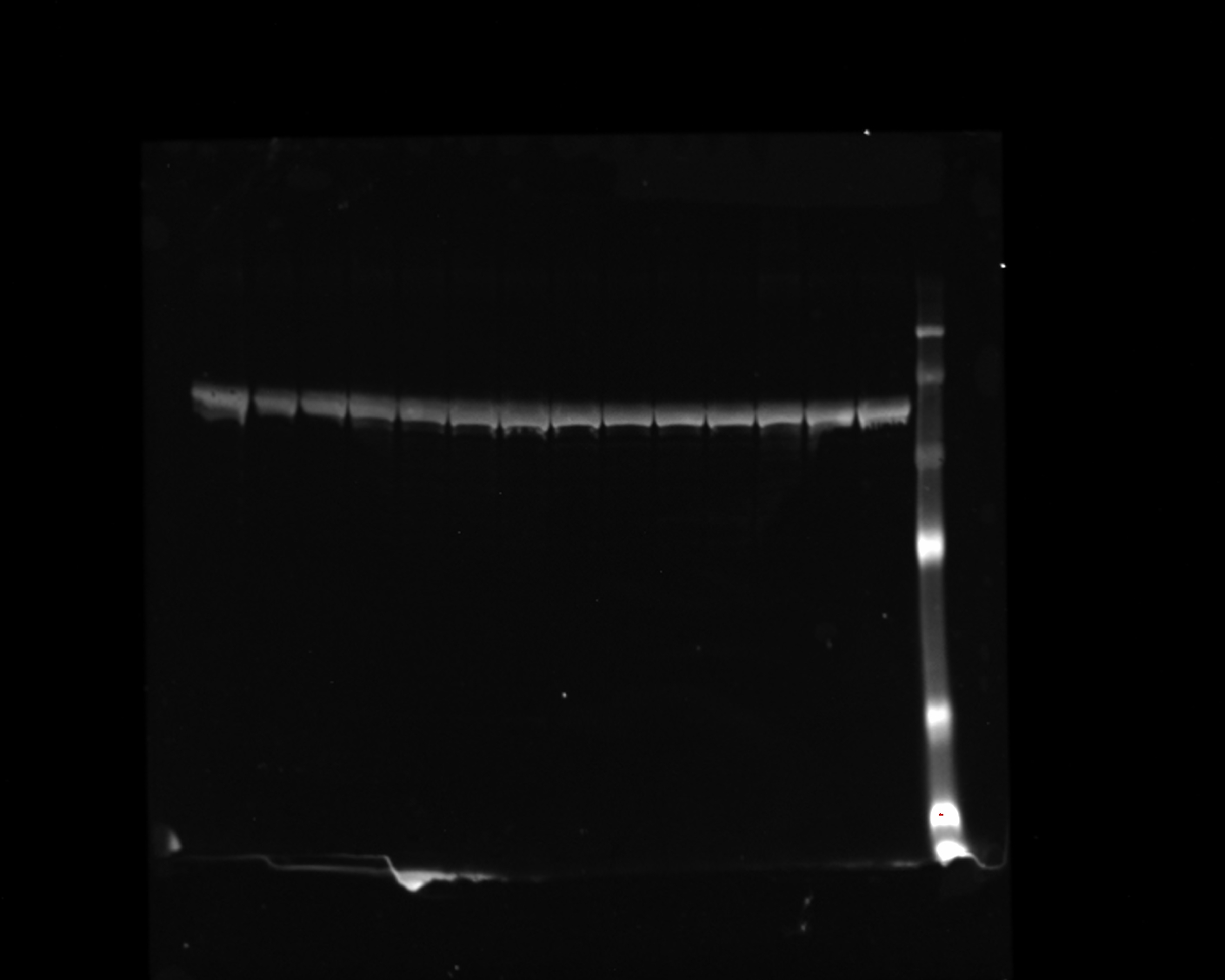

Supplement: S1 Data — (ZIP) [file pgen.1011059.s014.zip › SIdata/Figure 1 + S1A/S1A + part of 1D - RpoS no chase phosphate starvation/WB RpoS no chase phosphate starvation/Set 1/lmbchemidoc 2020-07-02 17h14m23s(DyLight 800).jpg]

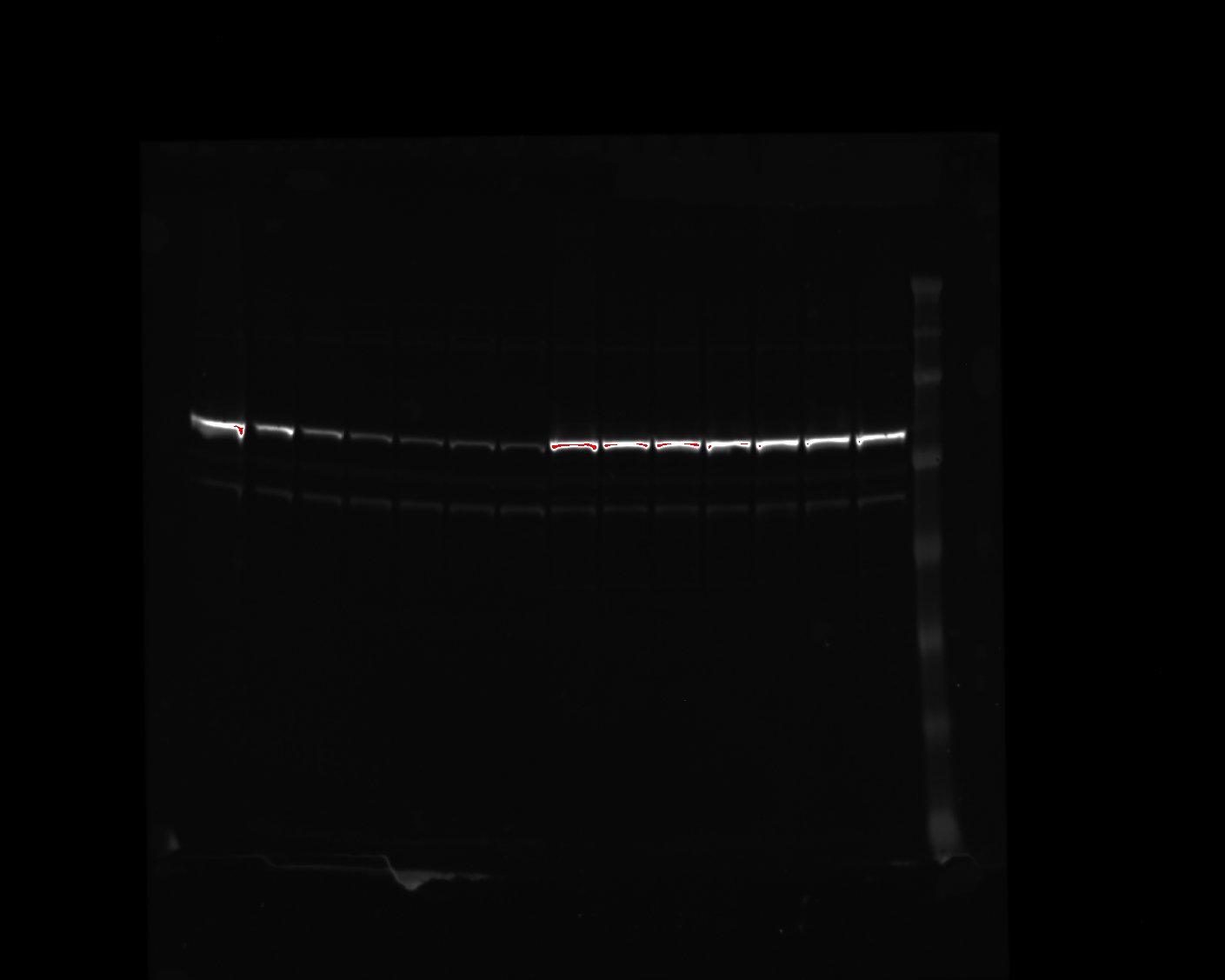

Supplement: S1 Data — (ZIP) [file pgen.1011059.s014.zip › SIdata/Figure 1 + S1A/S1A + part of 1D - RpoS no chase phosphate starvation/WB RpoS no chase phosphate starvation/Set 1/lmbchemidoc 2020-07-02 17h14m23s(StarBright B700).jpg]

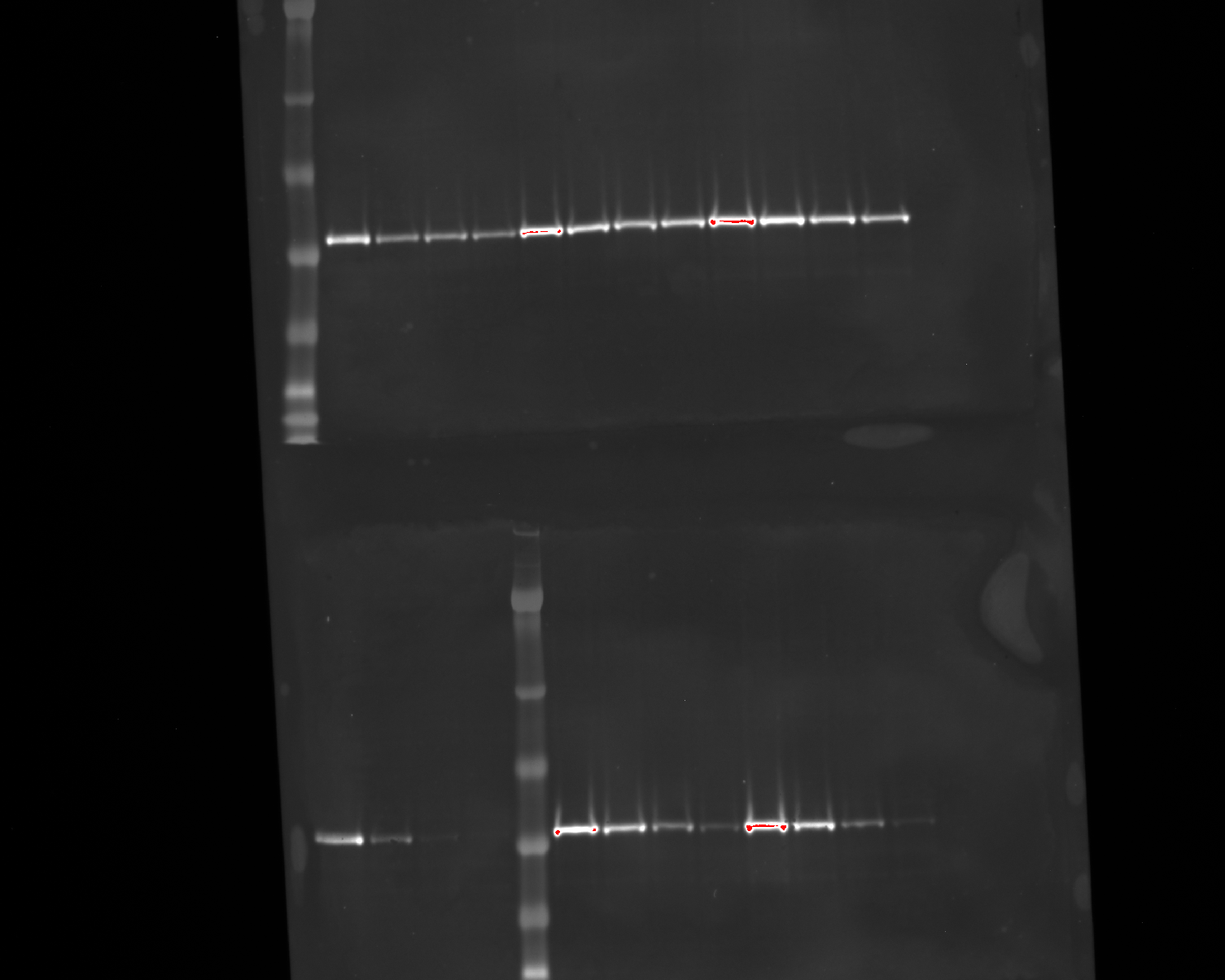

Supplement: S1 Data — (ZIP) [file pgen.1011059.s014.zip › SIdata/Figure 1 + S1A/S1A + part of 1D - RpoS no chase phosphate starvation/WB RpoS no chase recovery phosphate starvation /2020-12-12/lmbchemidoc 2020-12-11 20h23m24s(StarBright B700).tif]

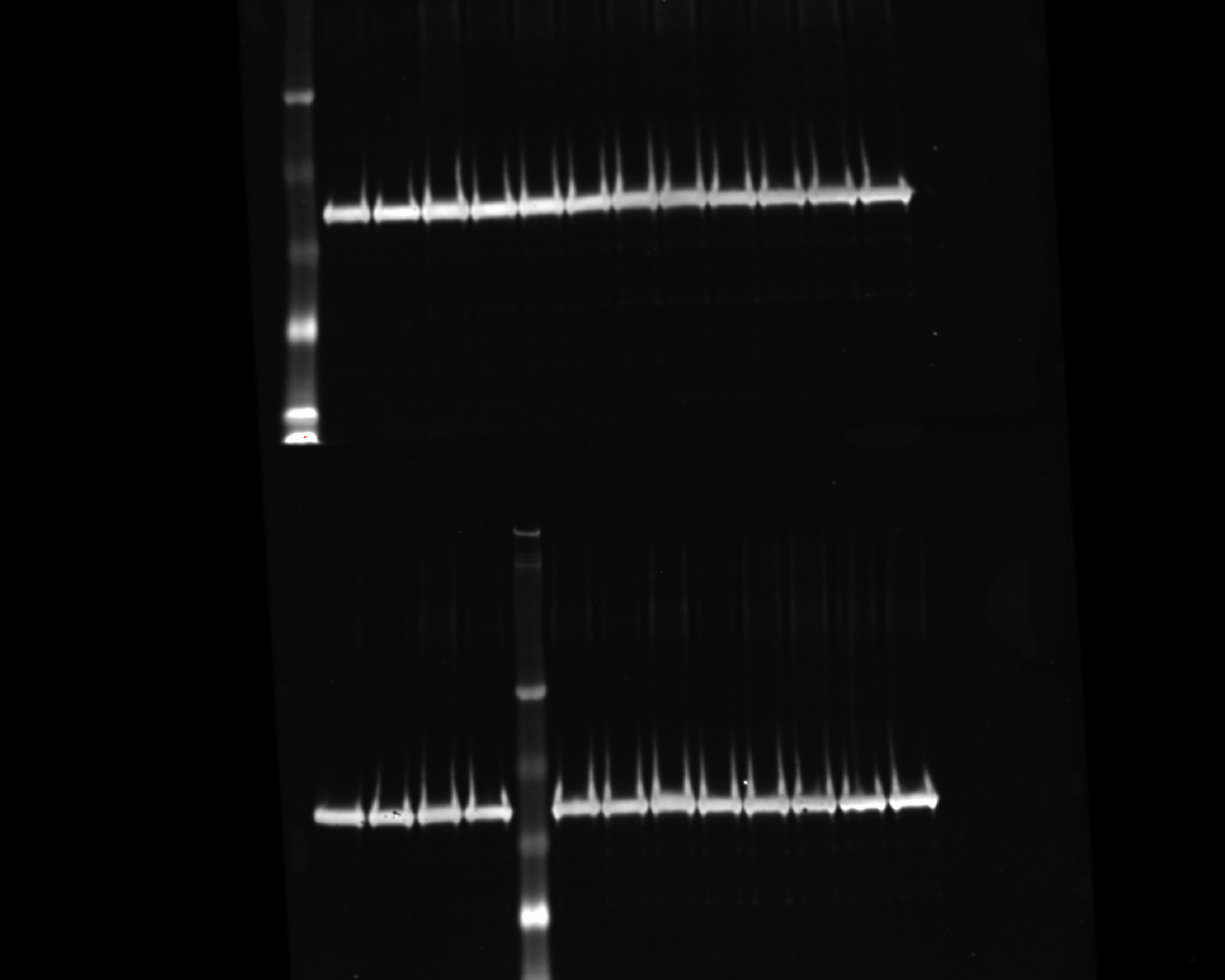

Supplement: S1 Data — (ZIP) [file pgen.1011059.s014.zip › SIdata/Figure 1 + S1A/S1A + part of 1D - RpoS no chase phosphate starvation/WB RpoS no chase recovery phosphate starvation /2020-12-12/lmbchemidoc 2020-12-11 20h23m24s(DyLight 800).tif]

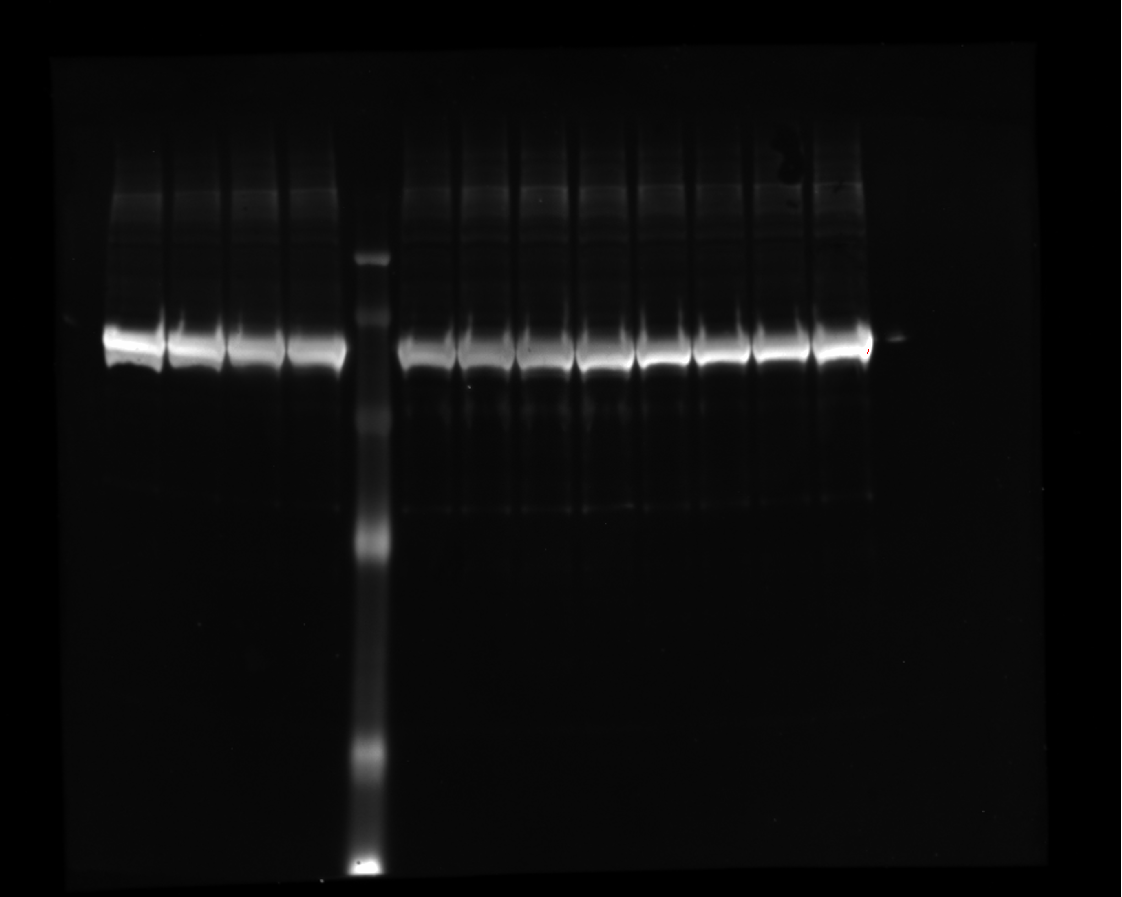

Supplement: S1 Data — (ZIP) [file pgen.1011059.s014.zip › SIdata/Figure 1 + S1A/S1A + part of 1D - RpoS no chase phosphate starvation/WB RpoS no chase recovery phosphate starvation /2020-11-24/lmbchemidoc 2020-11-24 19h48m00s(DyLight 800).tif]

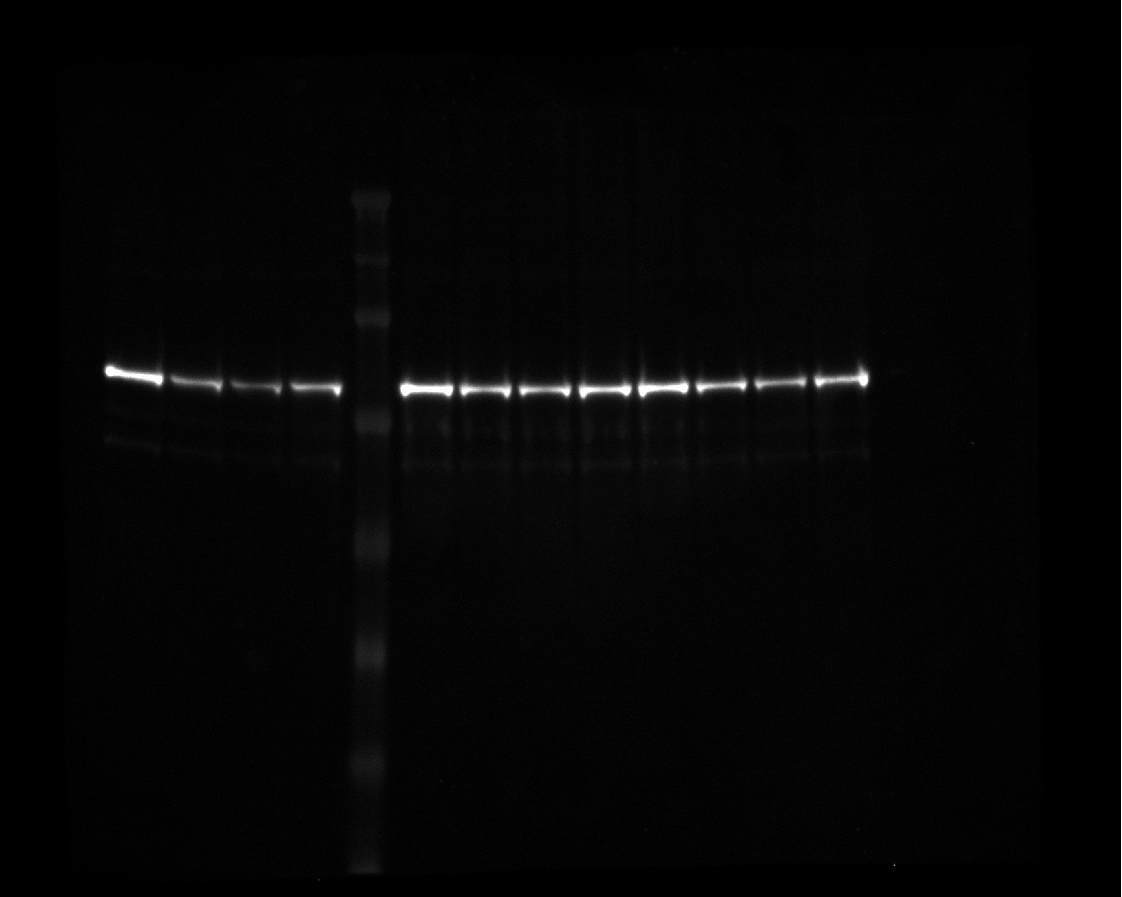

Supplement: S1 Data — (ZIP) [file pgen.1011059.s014.zip › SIdata/Figure 1 + S1A/S1A + part of 1D - RpoS no chase phosphate starvation/WB RpoS no chase recovery phosphate starvation /2020-11-24/lmbchemidoc 2020-11-24 19h48m00s(StarBright B700).tif]

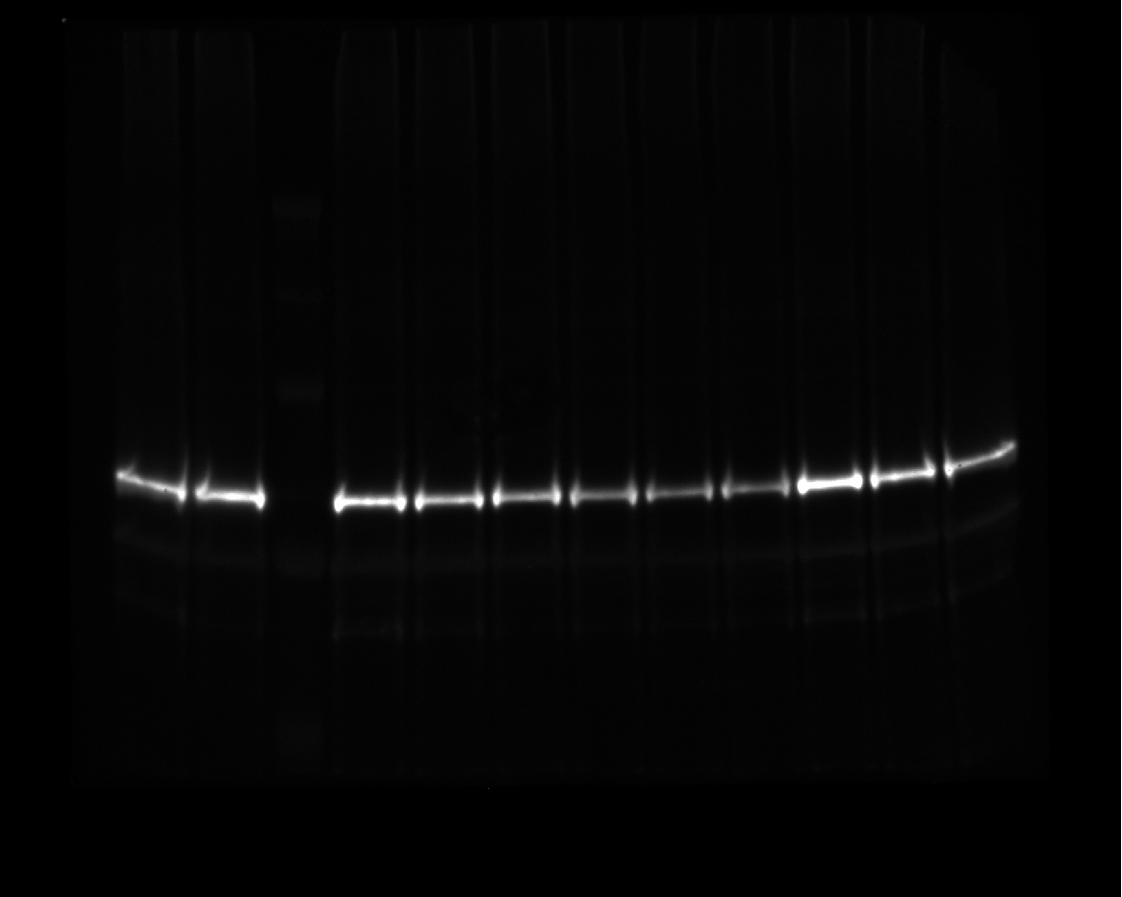

Supplement: S1 Data — (ZIP) [file pgen.1011059.s014.zip › SIdata/Figure 1 + S1A/S1A + part of 1D - RpoS no chase phosphate starvation/WB RpoS no chase recovery phosphate starvation /2020-11-23/lmbchemidoc 2020-11-23 19h56m47s(StarBright B700).tif]

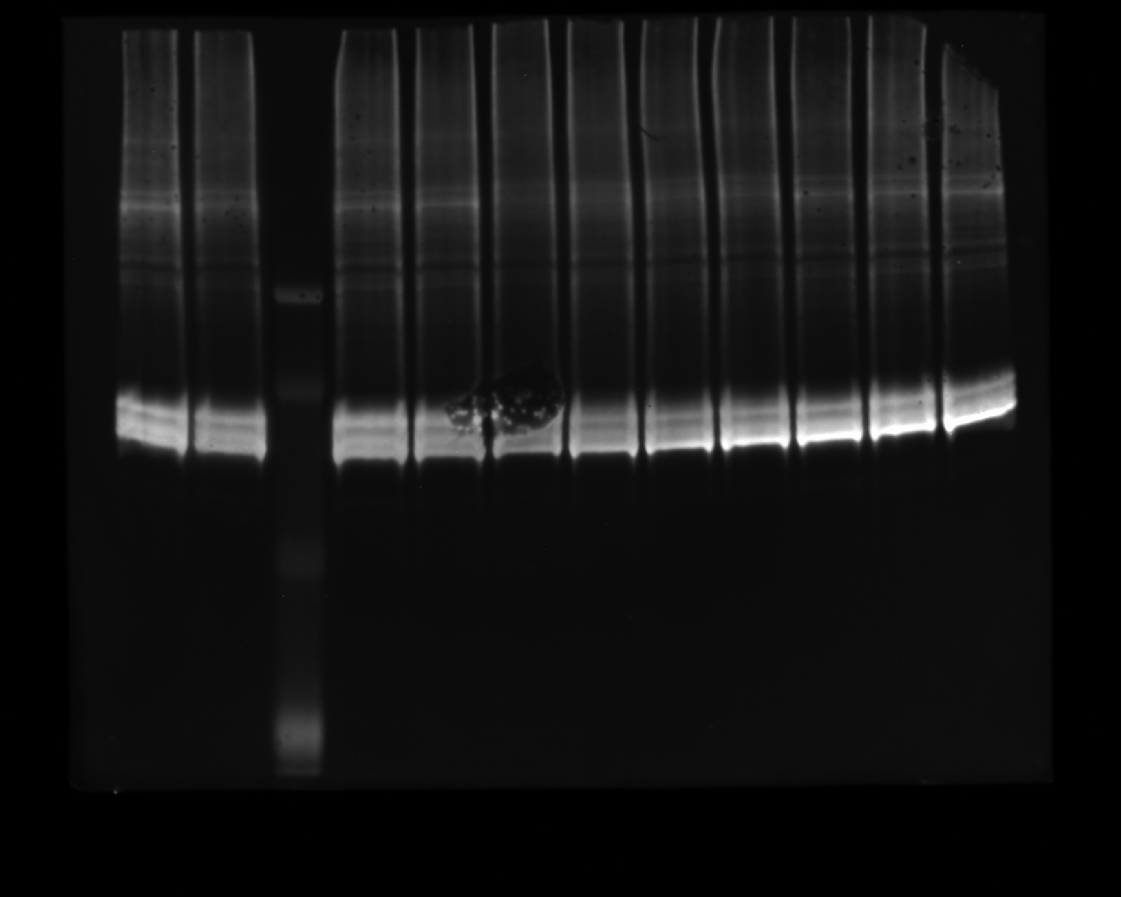

Supplement: S1 Data — (ZIP) [file pgen.1011059.s014.zip › SIdata/Figure 1 + S1A/S1A + part of 1D - RpoS no chase phosphate starvation/WB RpoS no chase recovery phosphate starvation /2020-11-23/lmbchemidoc 2020-11-23 19h56m47s(DyLight 800).tif]

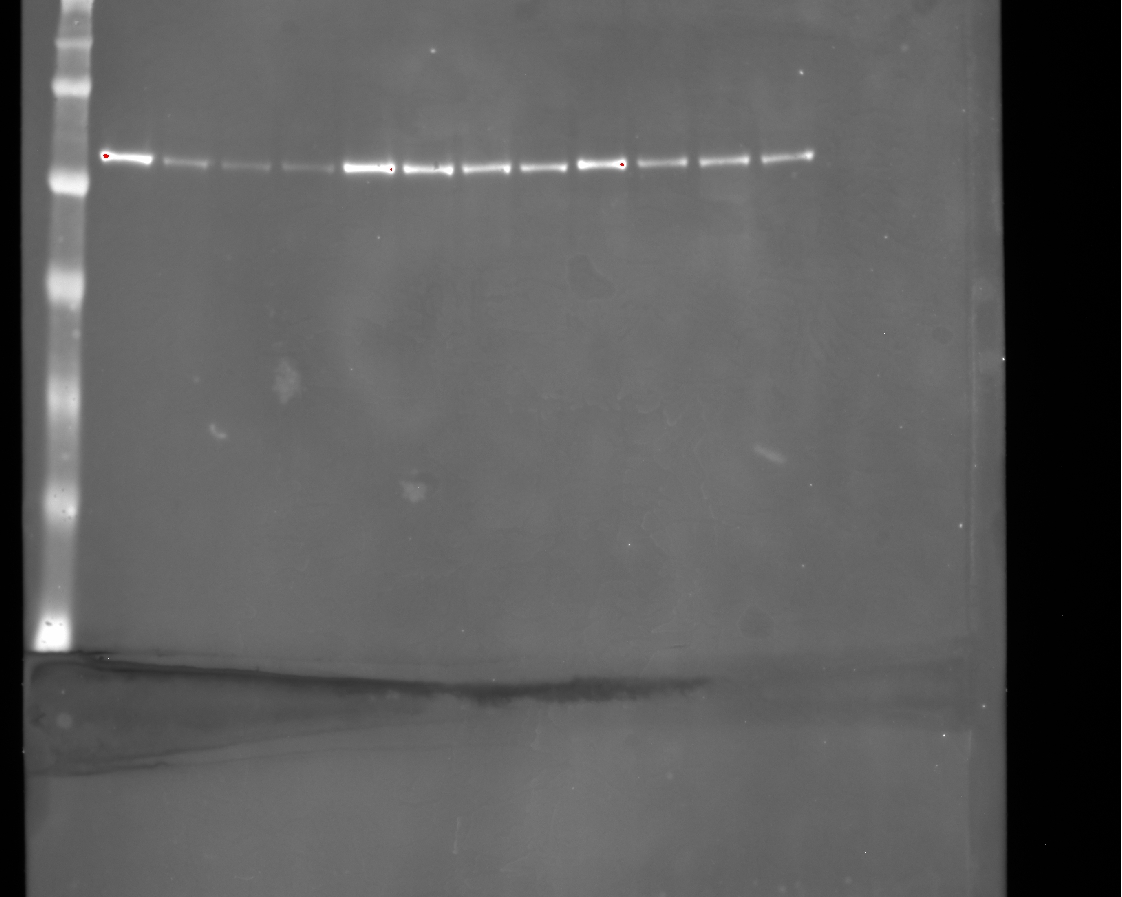

Supplement: S1 Data — (ZIP) [file pgen.1011059.s014.zip › SIdata/Figure 1 + S1A/S1A + part of 1D - RpoS no chase phosphate starvation/WB RpoS no chase recovery phosphate starvation /2020-12-28/lmbchemidoc 2020-12-28 17h47m40s(StarBright B700).jpg]

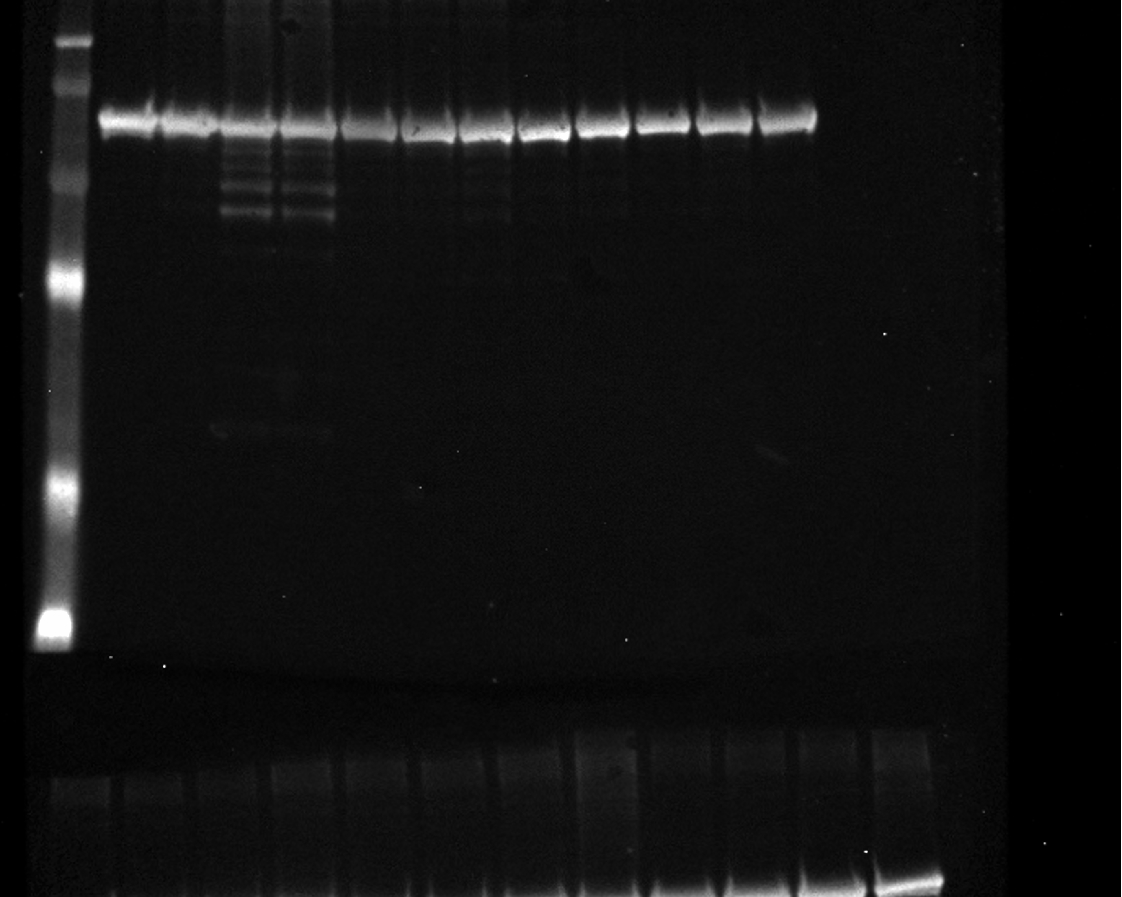

Supplement: S1 Data — (ZIP) [file pgen.1011059.s014.zip › SIdata/Figure 1 + S1A/S1A + part of 1D - RpoS no chase phosphate starvation/WB RpoS no chase recovery phosphate starvation /2020-12-28/lmbchemidoc 2020-12-28 17h47m40s(DyLight 800).jpg]

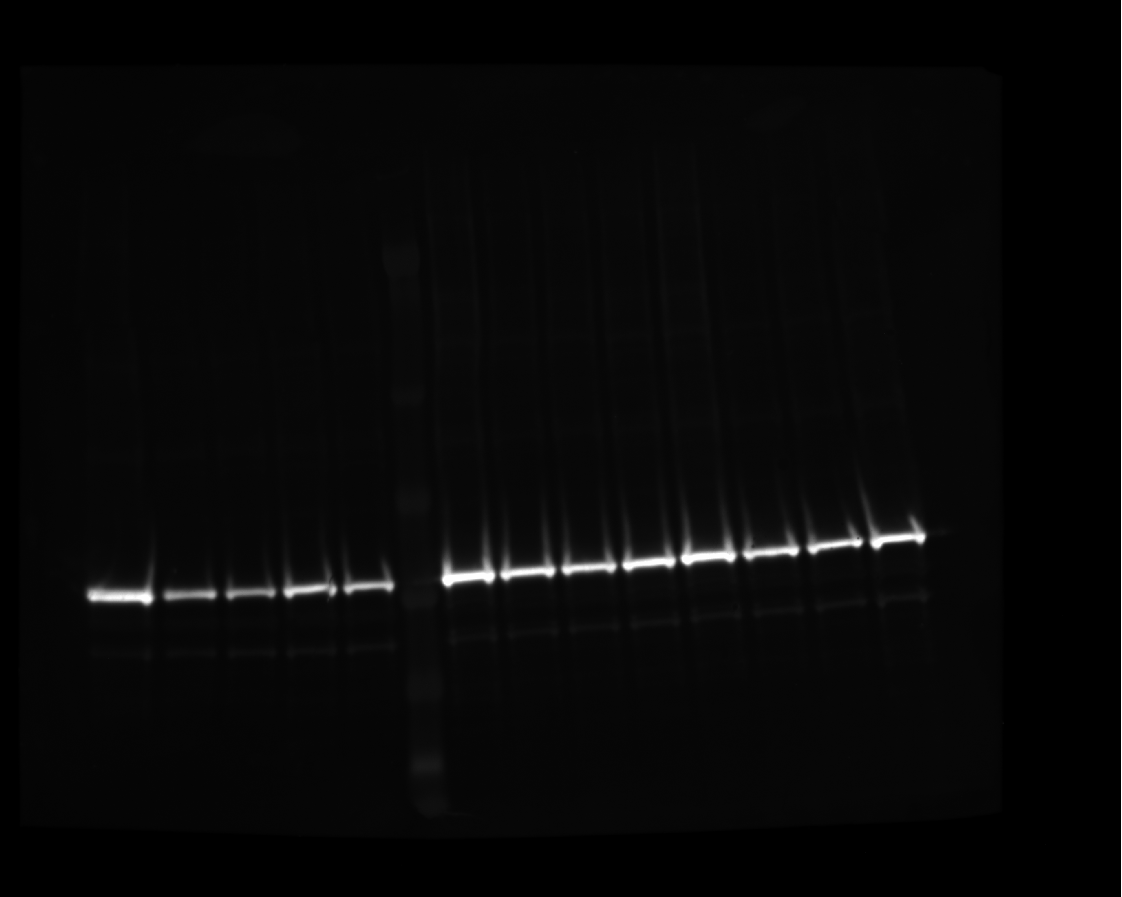

Supplement: S1 Data — (ZIP) [file pgen.1011059.s014.zip › SIdata/Figure 1 + S1A/S1A + part of 1D - RpoS no chase phosphate starvation/WB RpoS no chase recovery phosphate starvation /2020-12-10/lmbchemidoc 2020-12-09 19h50m01s(StarBright B700).tif]

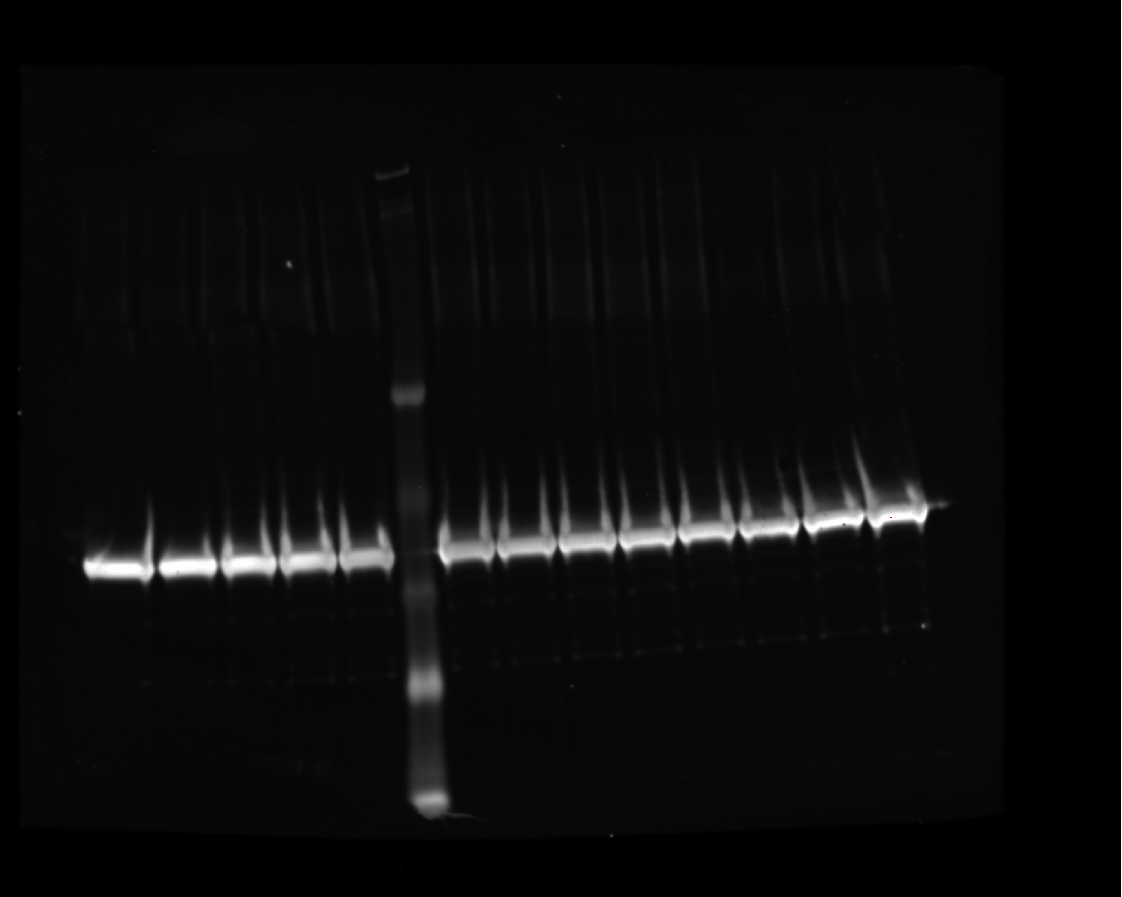

Supplement: S1 Data — (ZIP) [file pgen.1011059.s014.zip › SIdata/Figure 1 + S1A/S1A + part of 1D - RpoS no chase phosphate starvation/WB RpoS no chase recovery phosphate starvation /2020-12-10/lmbchemidoc 2020-12-09 19h50m01s(DyLight 800).tif]

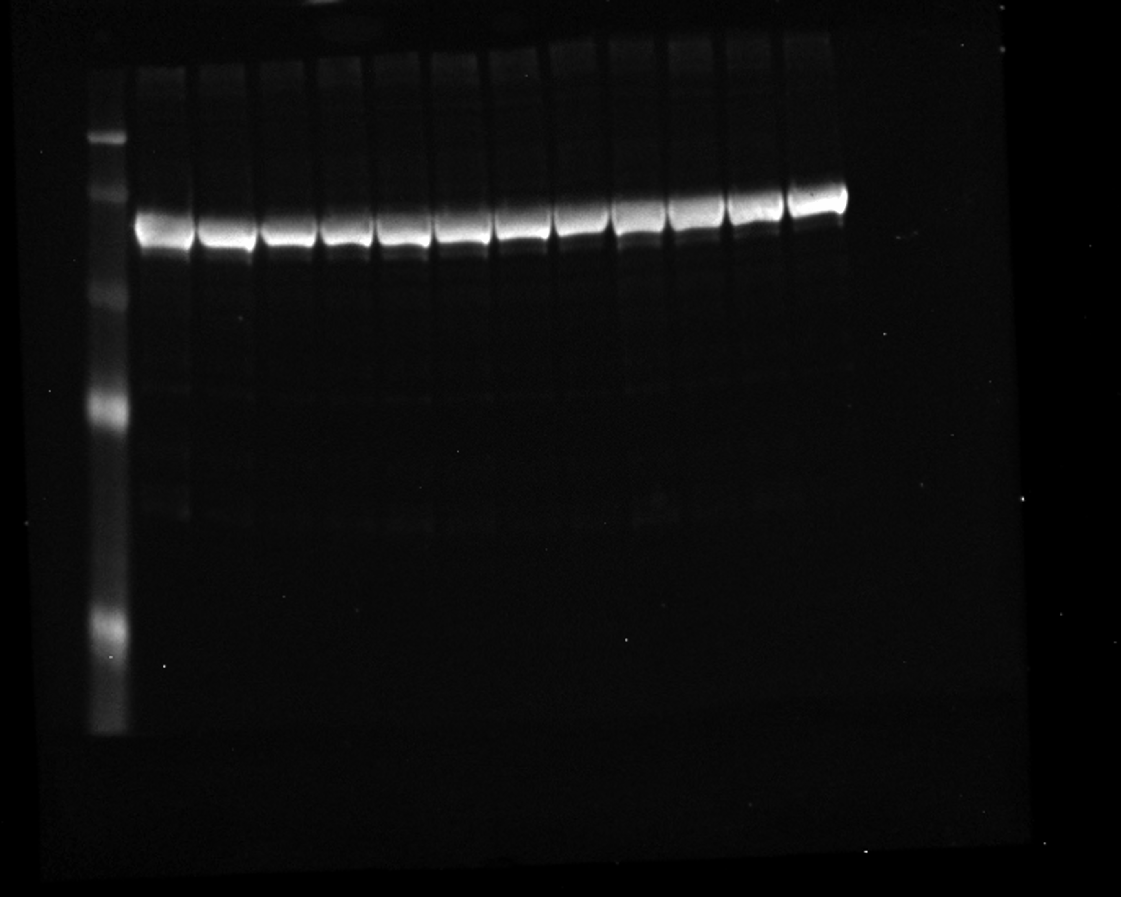

Supplement: S1 Data — (ZIP) [file pgen.1011059.s014.zip › SIdata/Figure 5 + S5 + S6/S5/S5A_phosphate starvation recovery RpoS/Set 2/lmbchemidoc 2021-01-25 17h41m56s(DyLight 800).tif]

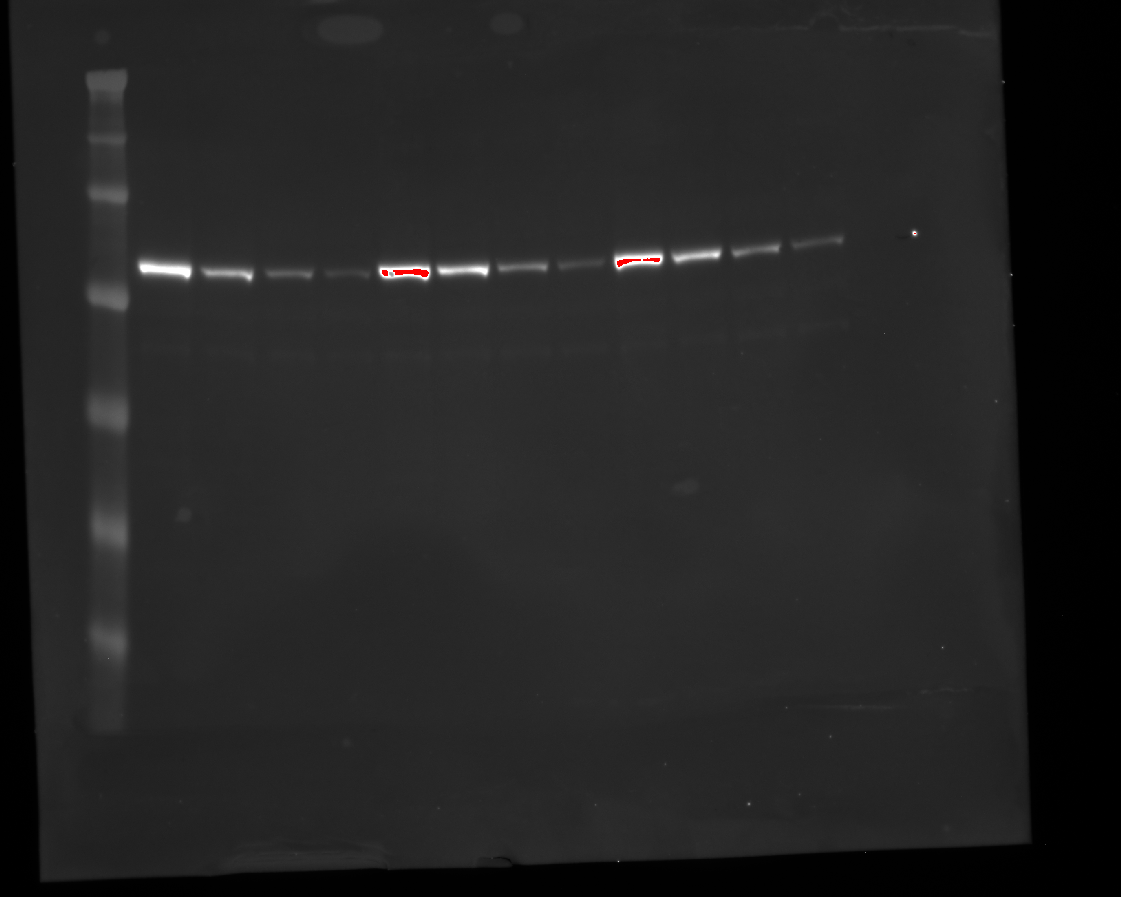

Supplement: S1 Data — (ZIP) [file pgen.1011059.s014.zip › SIdata/Figure 5 + S5 + S6/S5/S5A_phosphate starvation recovery RpoS/Set 2/lmbchemidoc 2021-01-25 17h41m56s(StarBright B700).tif]

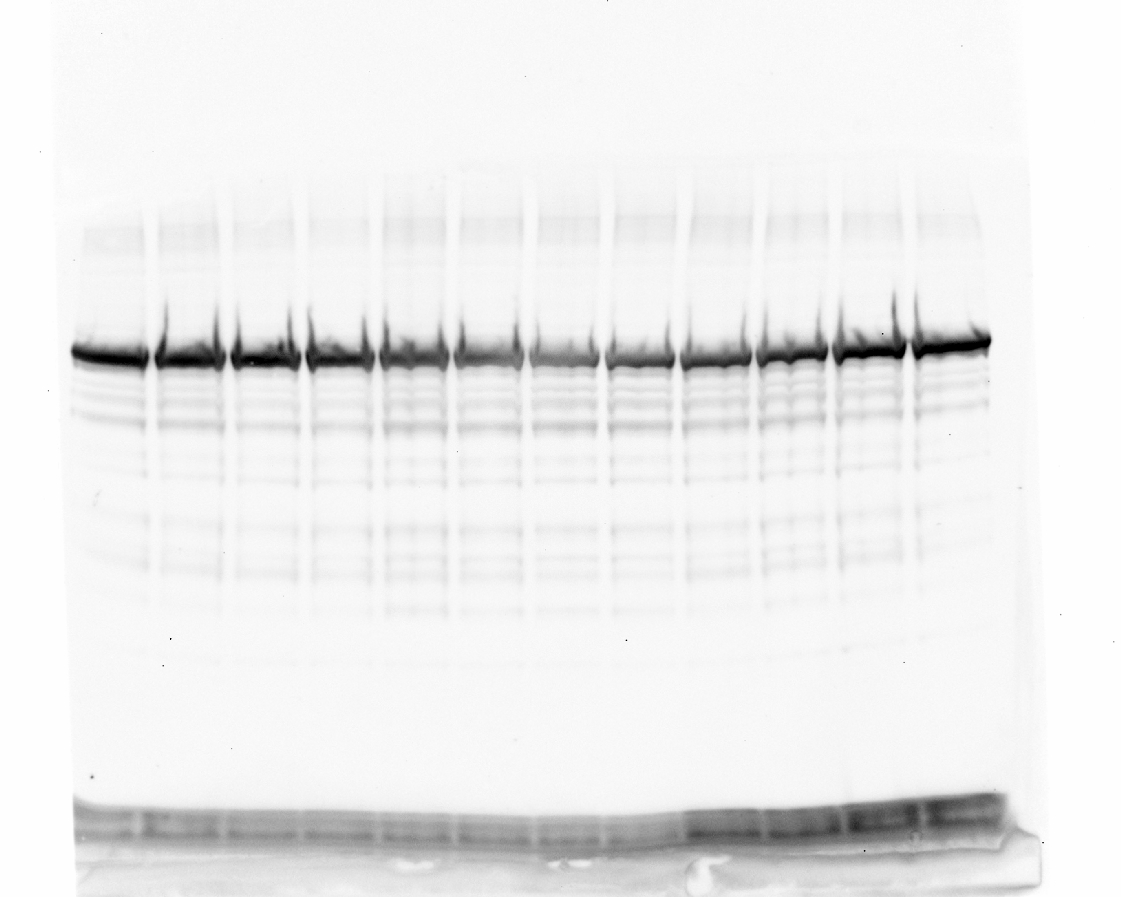

Supplement: S1 Data — (ZIP) [file pgen.1011059.s014.zip › SIdata/Figure 5 + S5 + S6/S5/S5A_phosphate starvation recovery RpoS/Set 3/lmbchemidoc 2022-01-04 17h01m09s(DyLight 800).jpg]

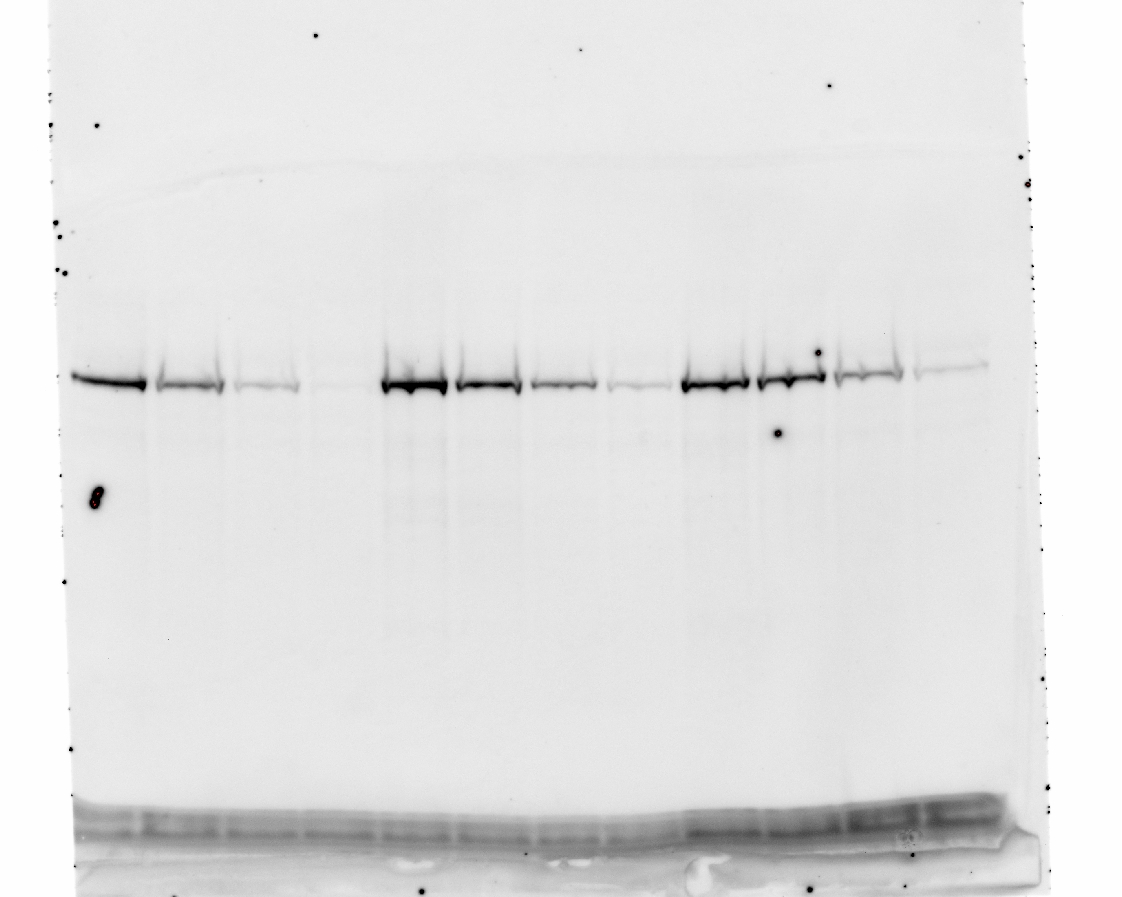

Supplement: S1 Data — (ZIP) [file pgen.1011059.s014.zip › SIdata/Figure 5 + S5 + S6/S5/S5A_phosphate starvation recovery RpoS/Set 3/lmbchemidoc 2022-01-04 17h01m09s(StarBright B700).jpg]

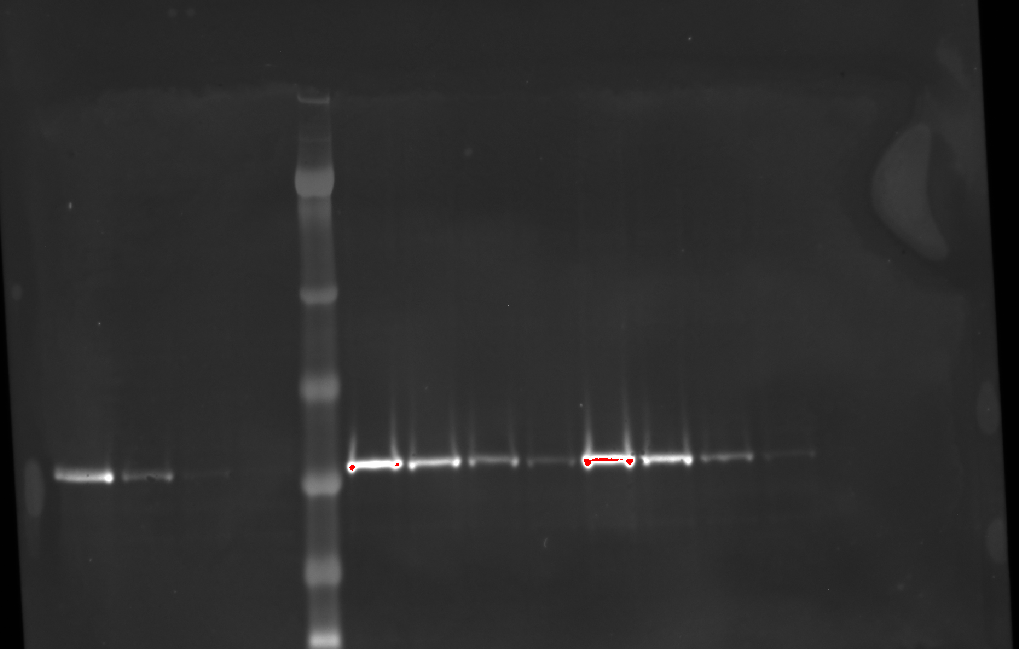

Supplement: S1 Data — (ZIP) [file pgen.1011059.s014.zip › SIdata/Figure 5 + S5 + S6/S5/S5A_phosphate starvation recovery RpoS/Set 1/lmbchemidoc 2020-12-11 20h23m24s(StarBright B700).tif]

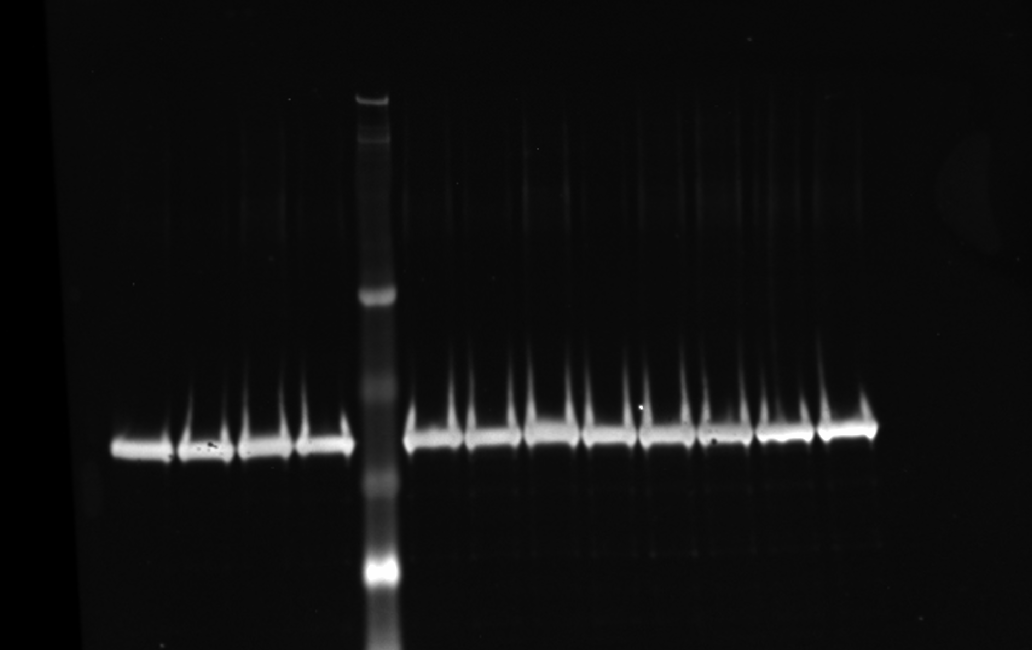

Supplement: S1 Data — (ZIP) [file pgen.1011059.s014.zip › SIdata/Figure 5 + S5 + S6/S5/S5A_phosphate starvation recovery RpoS/Set 1/lmbchemidoc 2020-12-11 20h23m24s(DyLight 800).tif]

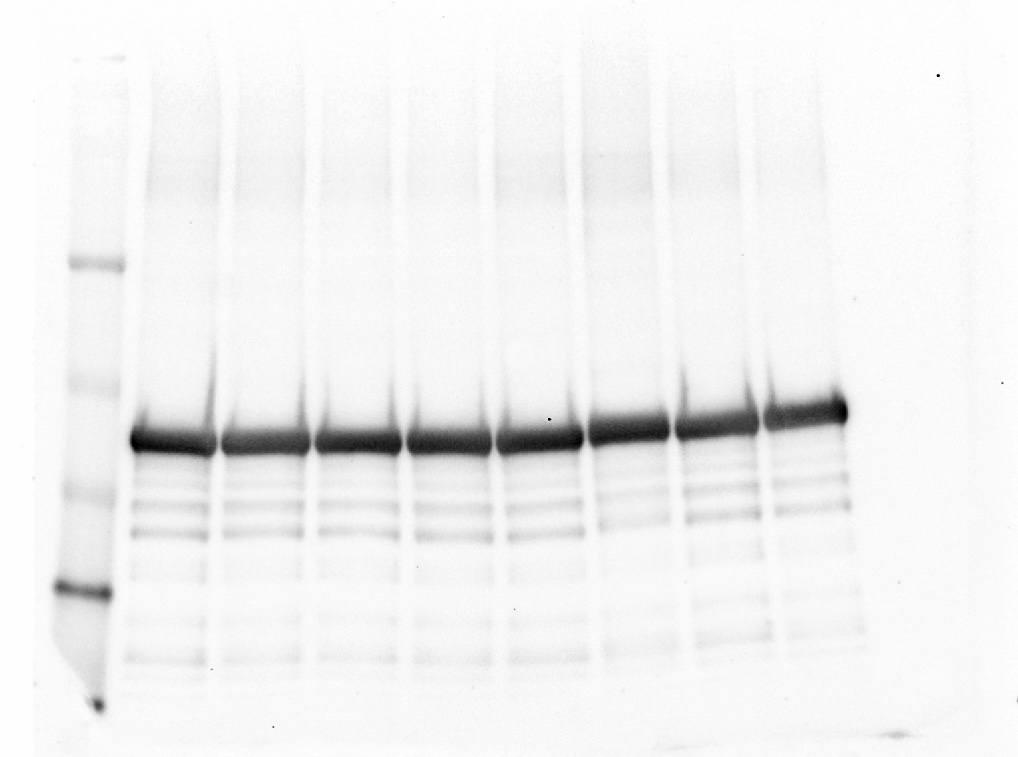

Supplement: S1 Data — (ZIP) [file pgen.1011059.s014.zip › SIdata/Figure 5 + S5 + S6/S5/S5B_glucose starvation recovery RpoS/Set 3/lmbchemidoc 2022-01-04 16h59m33s(DyLight 800).jpg]

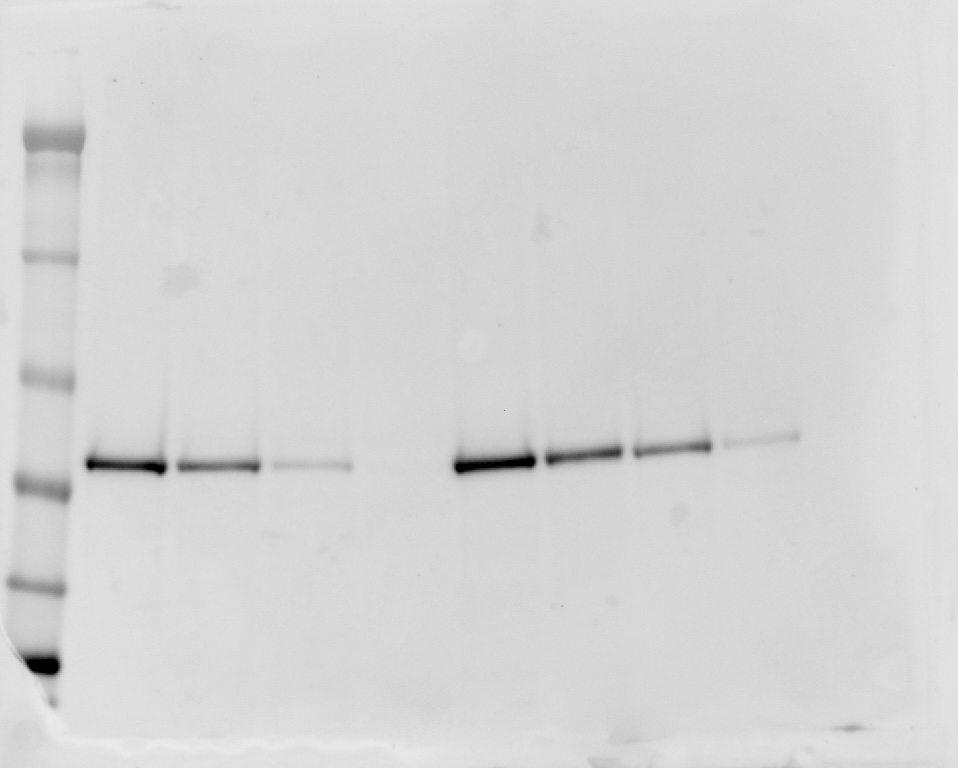

Supplement: S1 Data — (ZIP) [file pgen.1011059.s014.zip › SIdata/Figure 5 + S5 + S6/S5/S5B_glucose starvation recovery RpoS/Set 3/lmbchemidoc 2022-01-04 16h59m33s(StarBright B700).jpg]

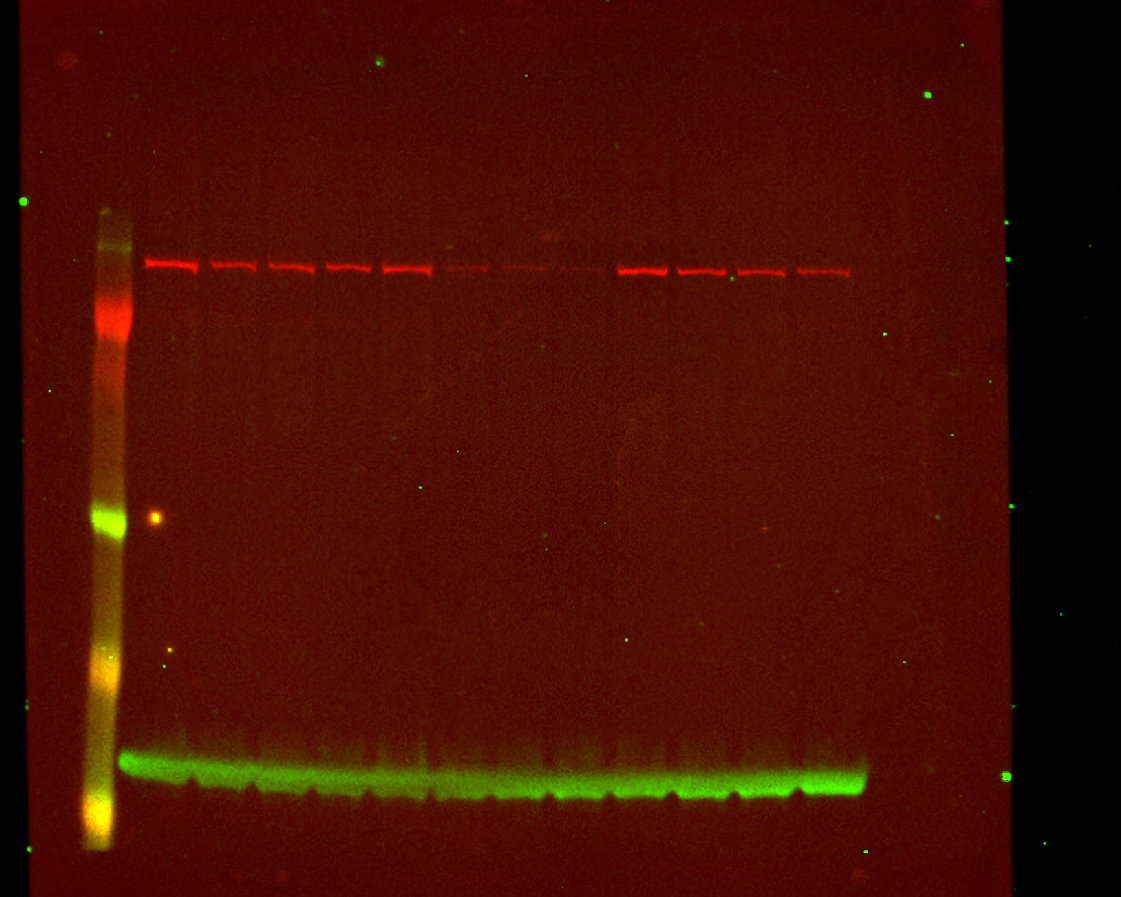

Supplement: S1 Data — (ZIP) [file pgen.1011059.s014.zip › SIdata/Figure 5 + S5 + S6/5 + S6 - RpoSLac/5+S6C_WB/20210311_RpoSLac/lmbchemidoc 2021-03-10 17h04m36s(Composite).jpg]

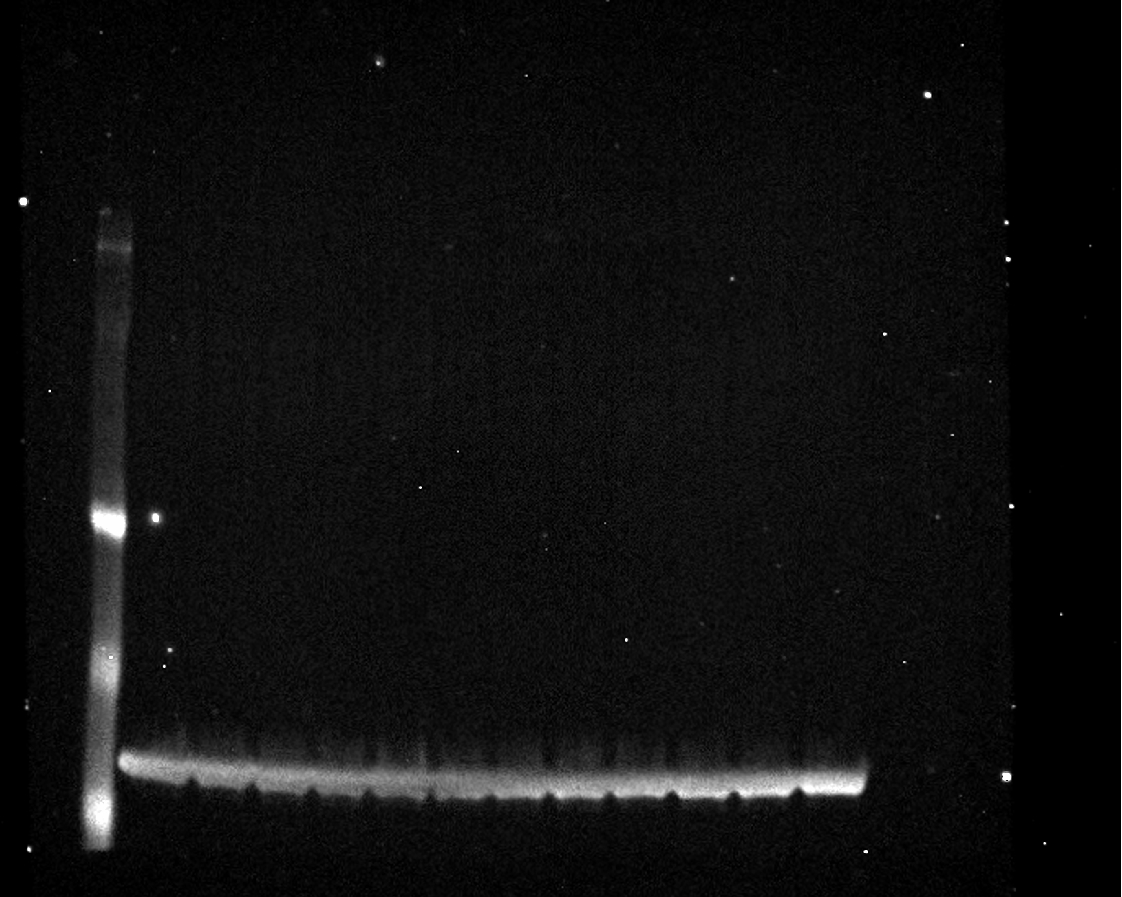

Supplement: S1 Data — (ZIP) [file pgen.1011059.s014.zip › SIdata/Figure 5 + S5 + S6/5 + S6 - RpoSLac/5+S6C_WB/20210311_RpoSLac/lmbchemidoc 2021-03-10 17h04m36s(DyLight 800).jpg]

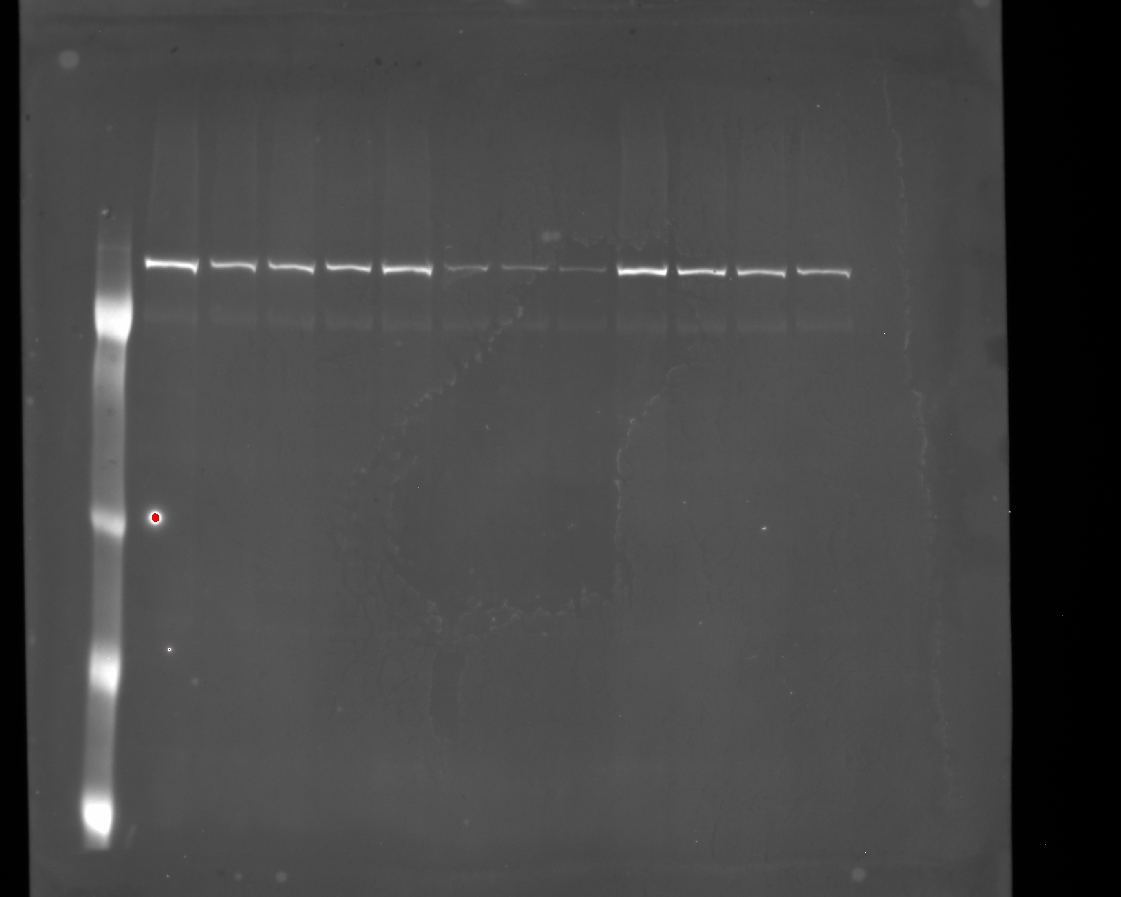

Supplement: S1 Data — (ZIP) [file pgen.1011059.s014.zip › SIdata/Figure 5 + S5 + S6/5 + S6 - RpoSLac/5+S6C_WB/20210311_RpoSLac/lmbchemidoc 2021-03-10 17h04m36s(StarBright B700).jpg]

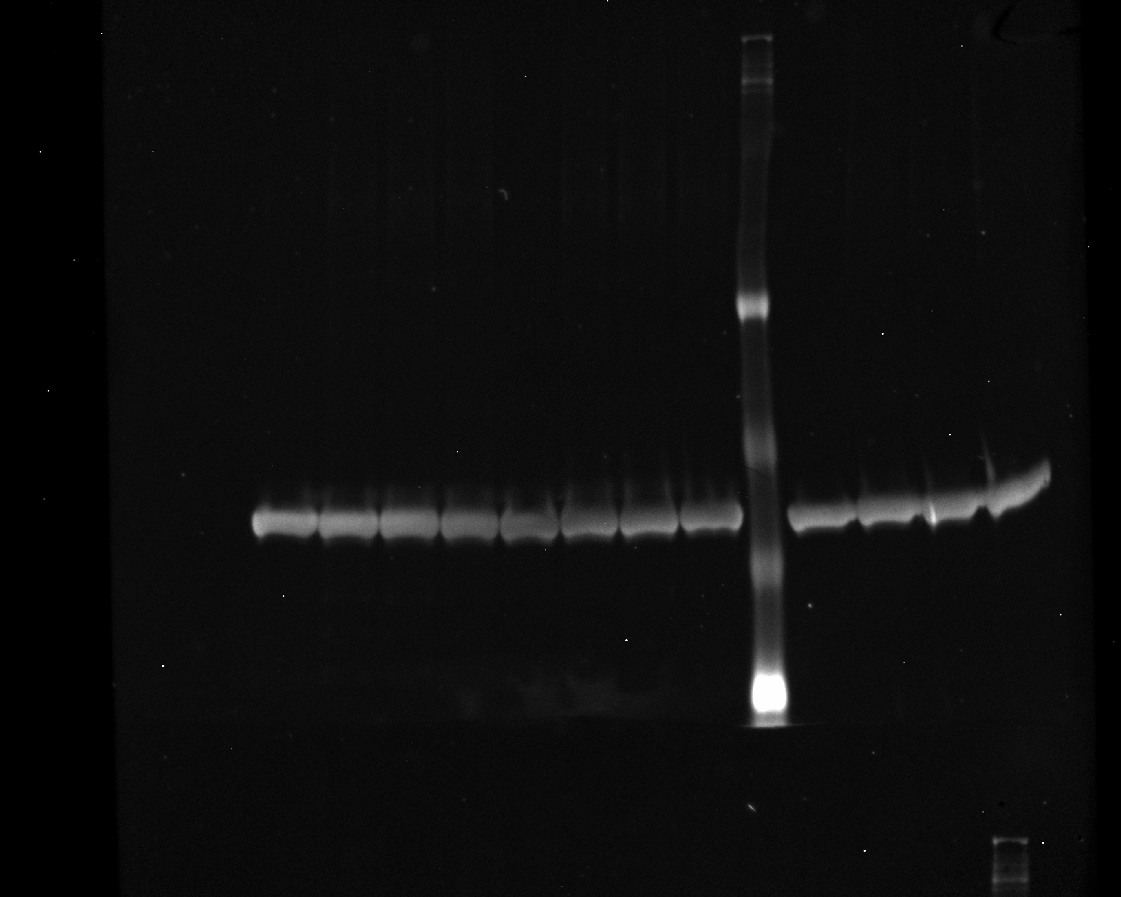

Supplement: S1 Data — (ZIP) [file pgen.1011059.s014.zip › SIdata/Figure 5 + S5 + S6/5 + S6 - RpoSLac/5+S6C_WB/20210224_RpoSLac/lmbchemidoc 2021-02-24 17h11m05s(DyLight 800).tif]

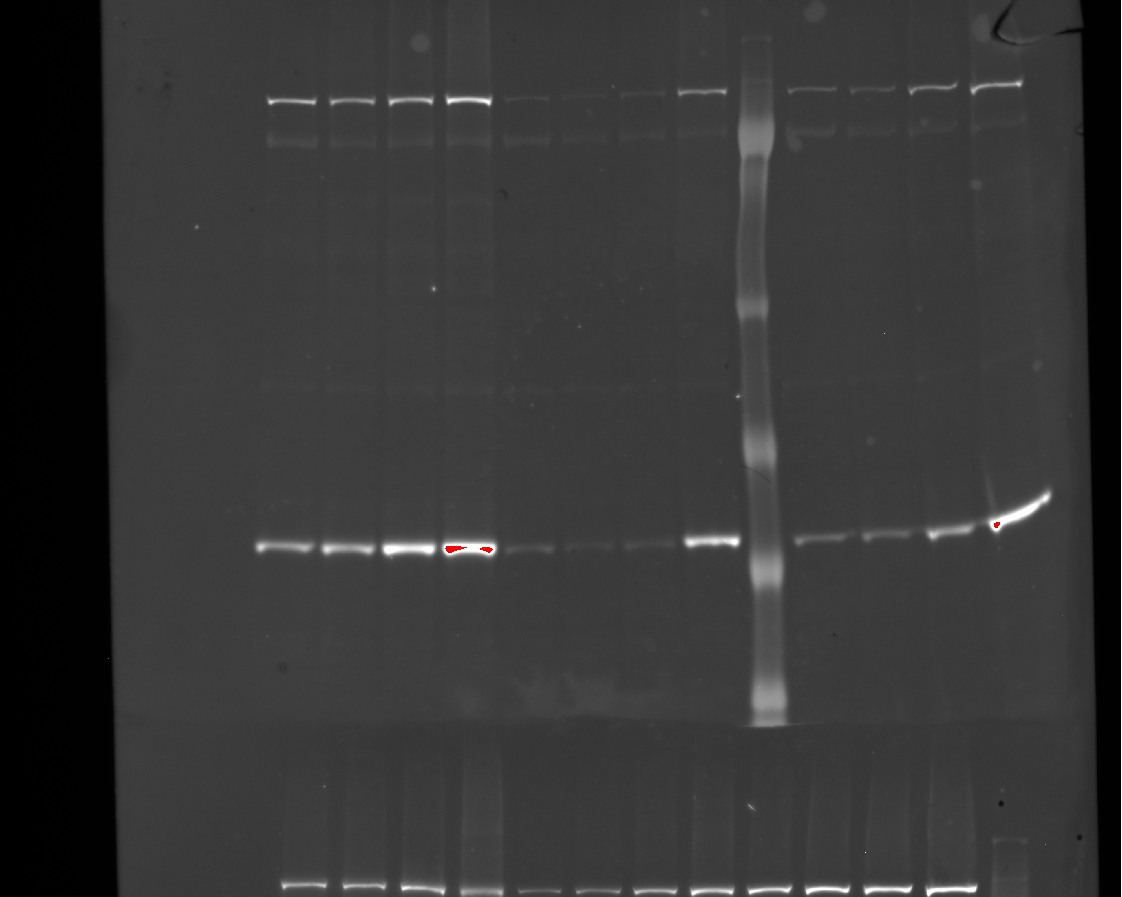

Supplement: S1 Data — (ZIP) [file pgen.1011059.s014.zip › SIdata/Figure 5 + S5 + S6/5 + S6 - RpoSLac/5+S6C_WB/20210224_RpoSLac/lmbchemidoc 2021-02-24 17h11m05s(StarBright B700).jpg]

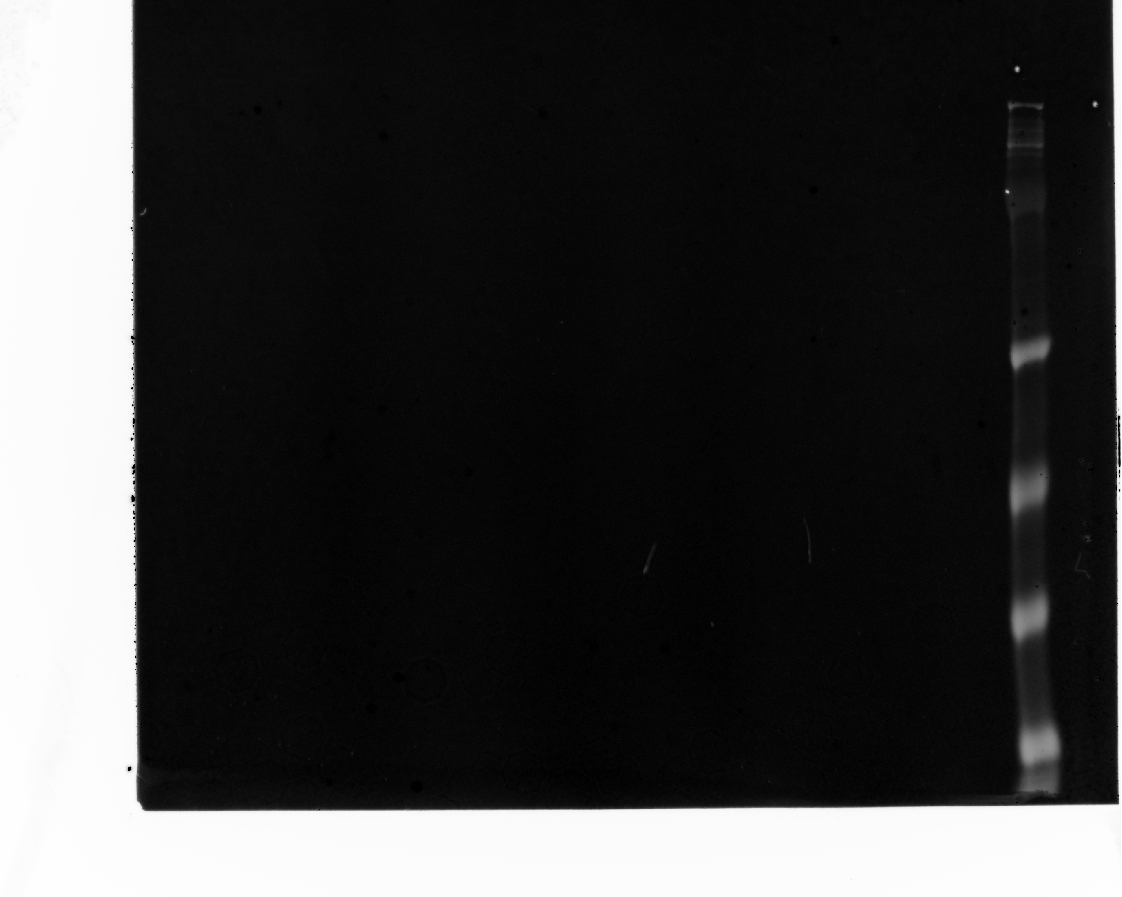

Supplement: S1 Data — (ZIP) [file pgen.1011059.s014.zip › SIdata/Figure 5 + S5 + S6/5 + S6 - RpoSLac/5+S6C_WB/20210224_RpoSLac/lmbchemidoc 2021-02-24 17h14m38s(Colorimetric).jpg]

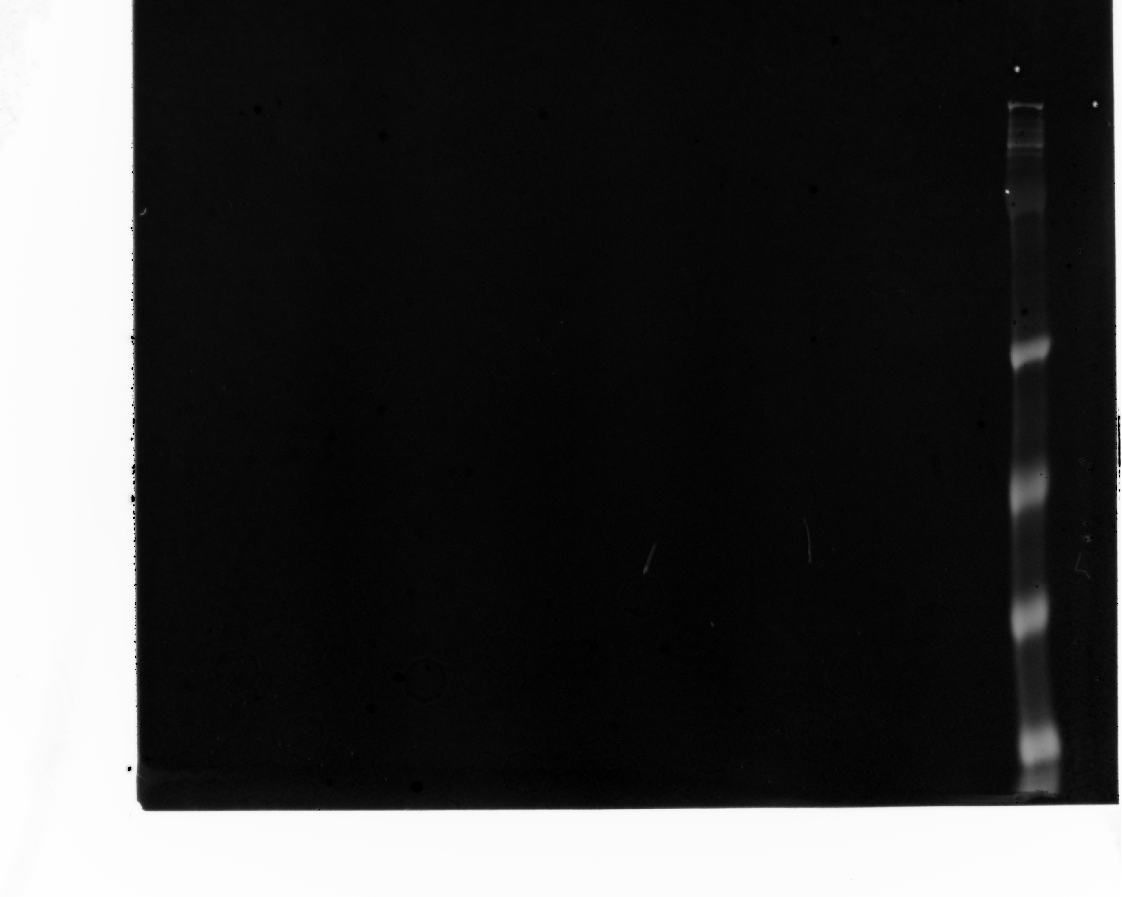

Supplement: S1 Data — (ZIP) [file pgen.1011059.s014.zip › SIdata/Figure 5 + S5 + S6/5 + S6 - RpoSLac/5+S6C_WB/20210224_RpoSLac/lmbchemidoc 2021-02-24 17h14m38s(Colorimetric).tif]

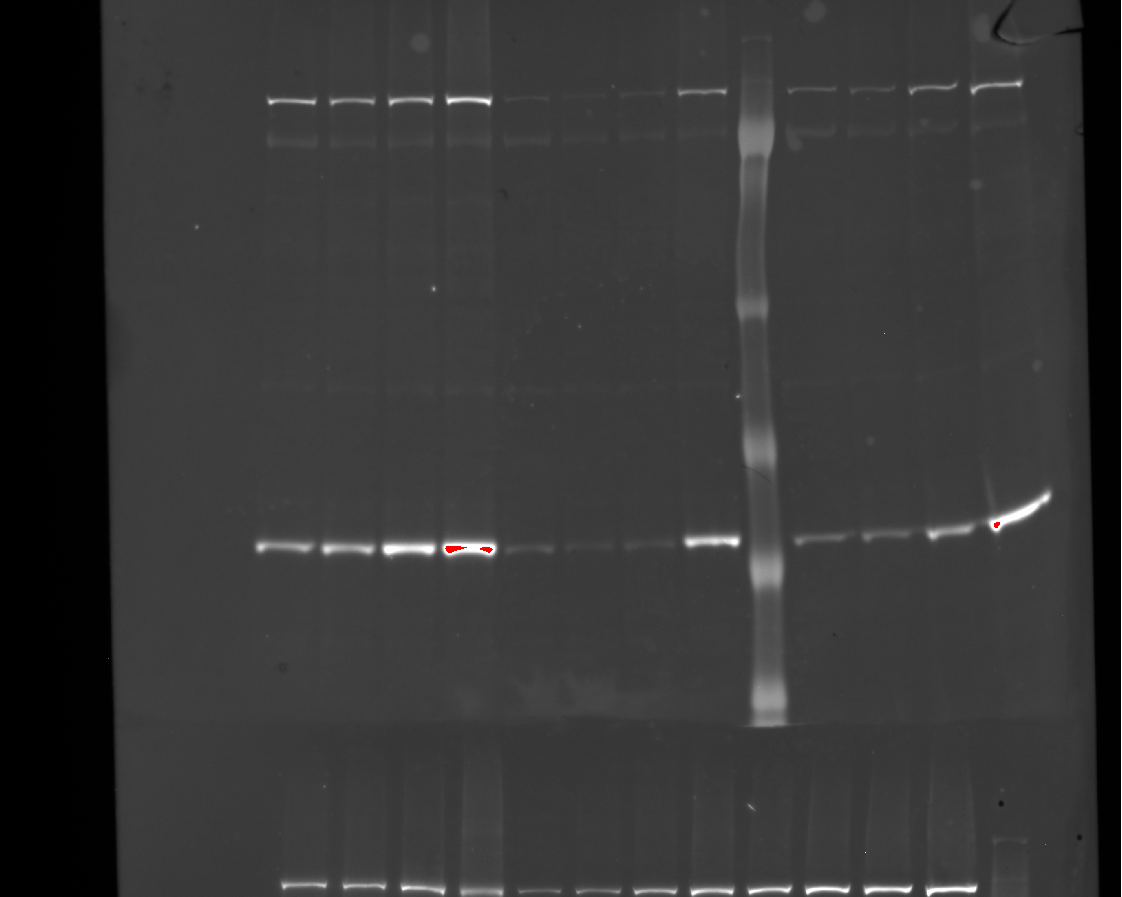

Supplement: S1 Data — (ZIP) [file pgen.1011059.s014.zip › SIdata/Figure 5 + S5 + S6/5 + S6 - RpoSLac/5+S6C_WB/20210224_RpoSLac/lmbchemidoc 2021-02-24 17h11m05s(StarBright B700).tif]

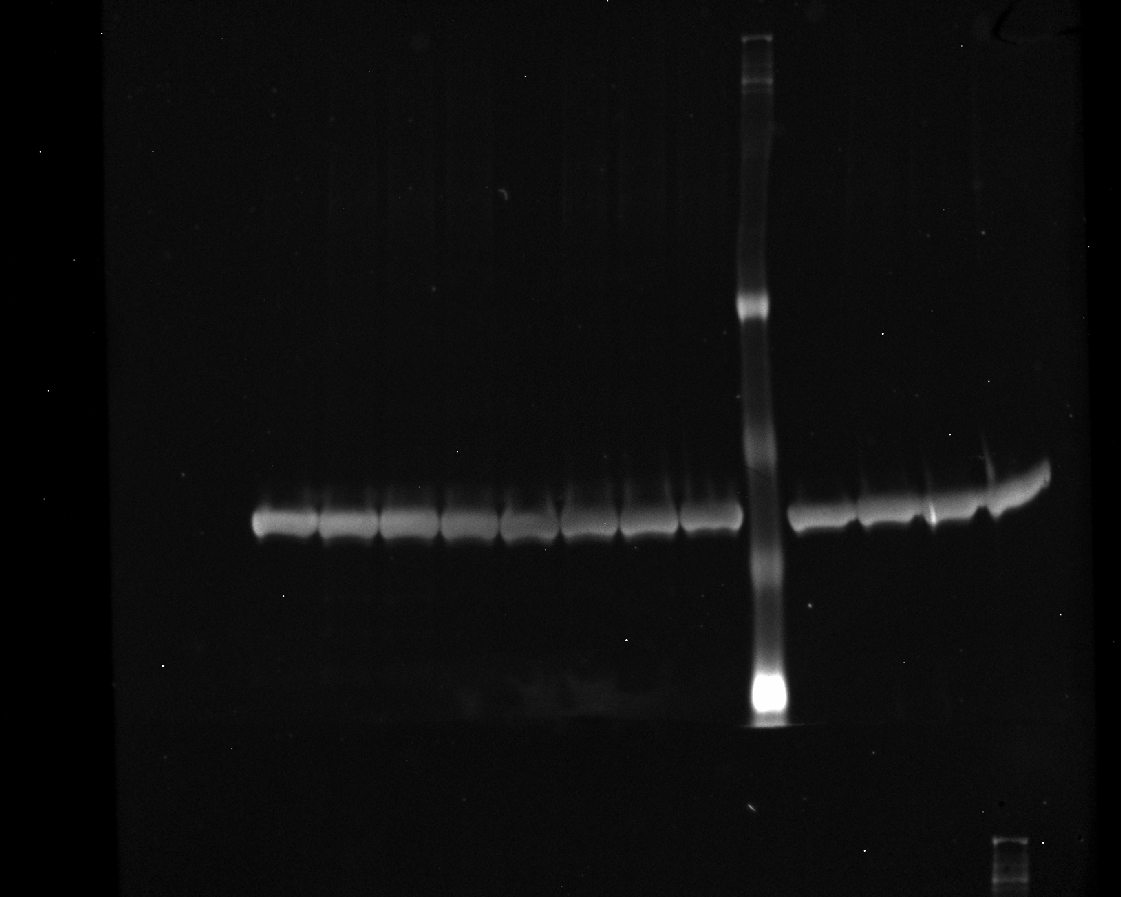

Supplement: S1 Data — (ZIP) [file pgen.1011059.s014.zip › SIdata/Figure 5 + S5 + S6/5 + S6 - RpoSLac/5+S6C_WB/20210224_RpoSLac/lmbchemidoc 2021-02-24 17h11m05s(DyLight 800).jpg]

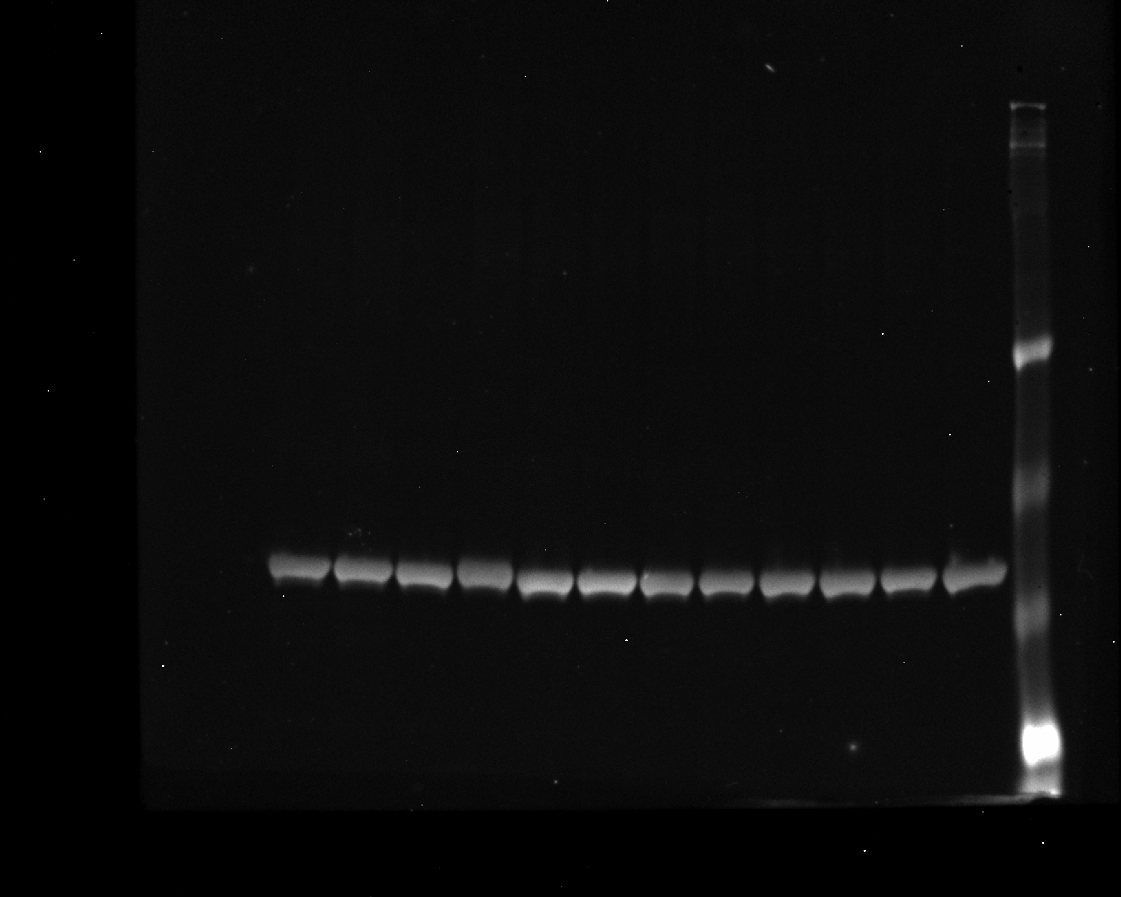

Supplement: S1 Data — (ZIP) [file pgen.1011059.s014.zip › SIdata/Figure 5 + S5 + S6/5 + S6 - RpoSLac/5+S6C_WB/20210224_RpoSLac/lmbchemidoc 2021-02-24 17h14m38s(DyLight 800).jpg]

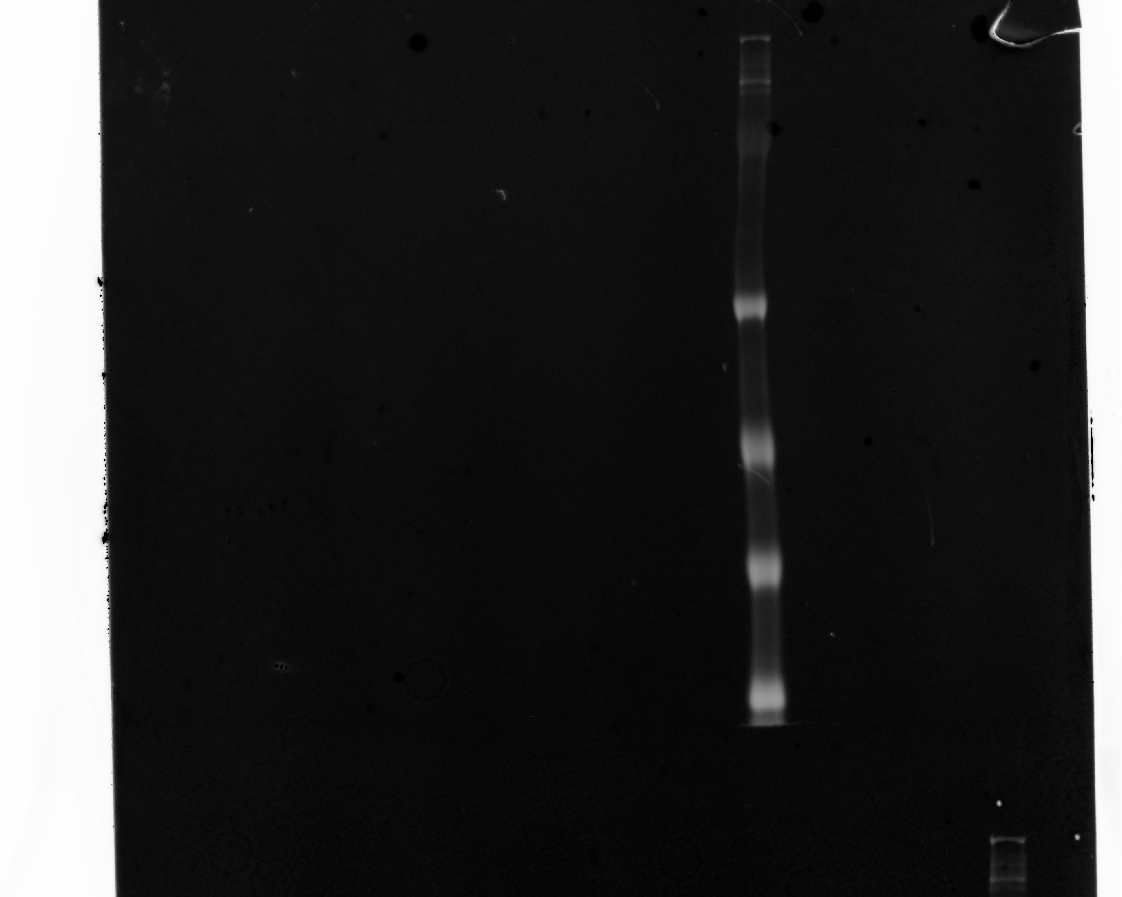

Supplement: S1 Data — (ZIP) [file pgen.1011059.s014.zip › SIdata/Figure 5 + S5 + S6/5 + S6 - RpoSLac/5+S6C_WB/20210224_RpoSLac/lmbchemidoc 2021-02-24 17h11m05s(Colorimetric).tif]

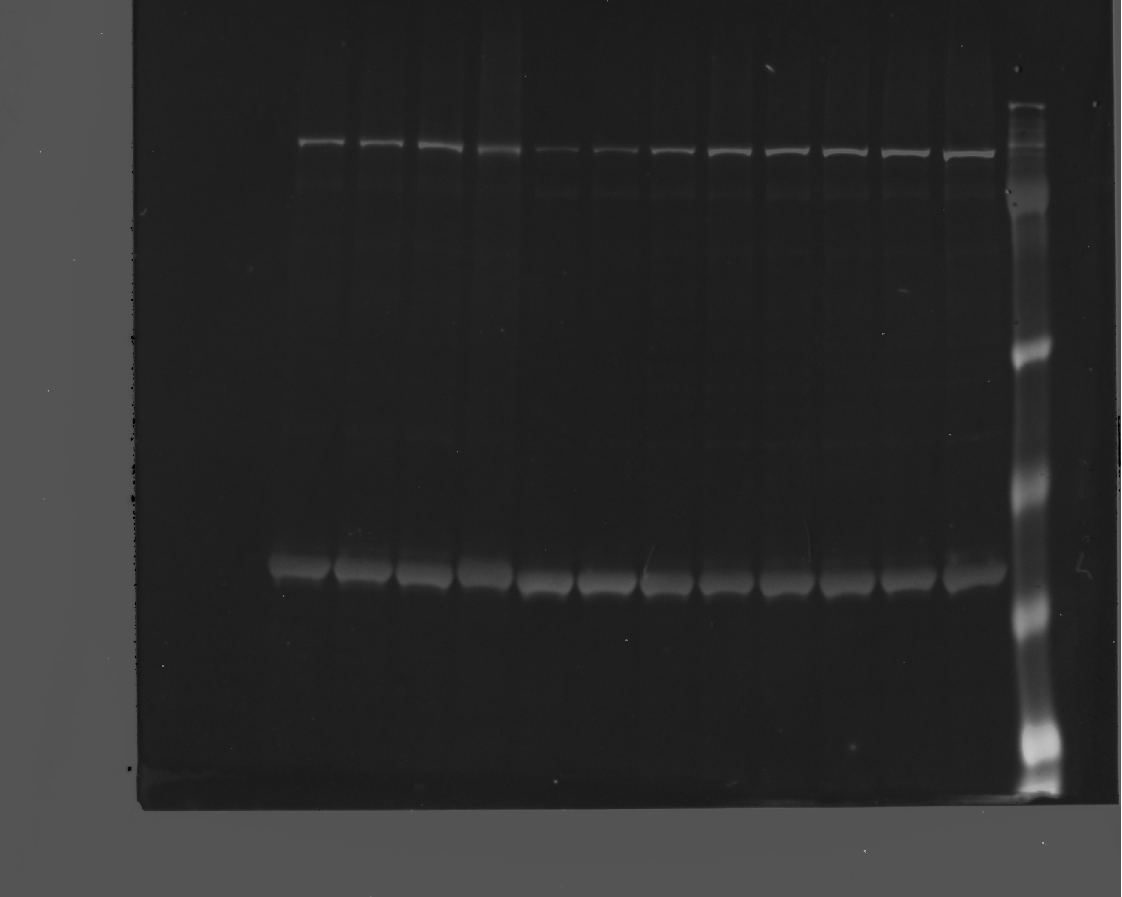

Supplement: S1 Data — (ZIP) [file pgen.1011059.s014.zip › SIdata/Figure 5 + S5 + S6/5 + S6 - RpoSLac/5+S6C_WB/20210224_RpoSLac/lmbchemidoc 2021-02-24 17h14m38s(Composite).tif]

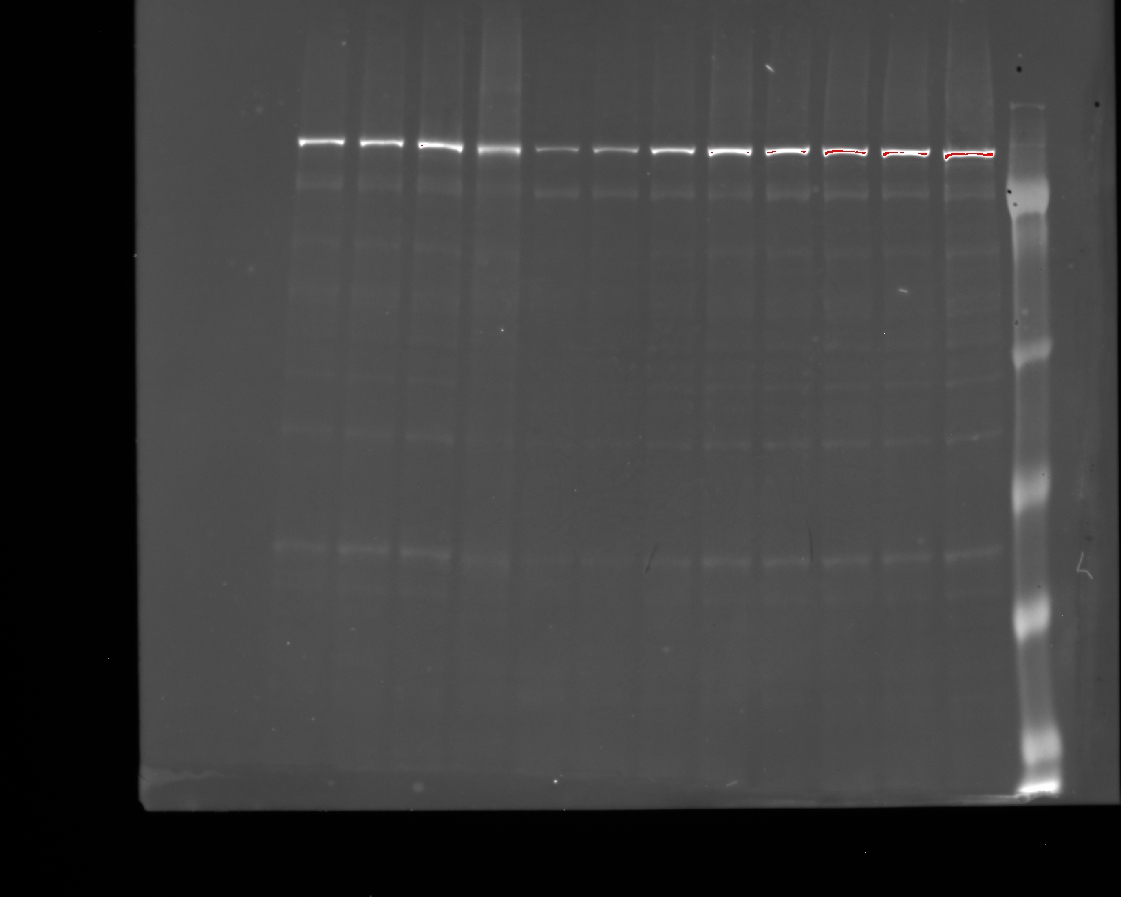

Supplement: S1 Data — (ZIP) [file pgen.1011059.s014.zip › SIdata/Figure 5 + S5 + S6/5 + S6 - RpoSLac/5+S6C_WB/20210224_RpoSLac/lmbchemidoc 2021-02-24 17h14m38s(StarBright B700).jpg]

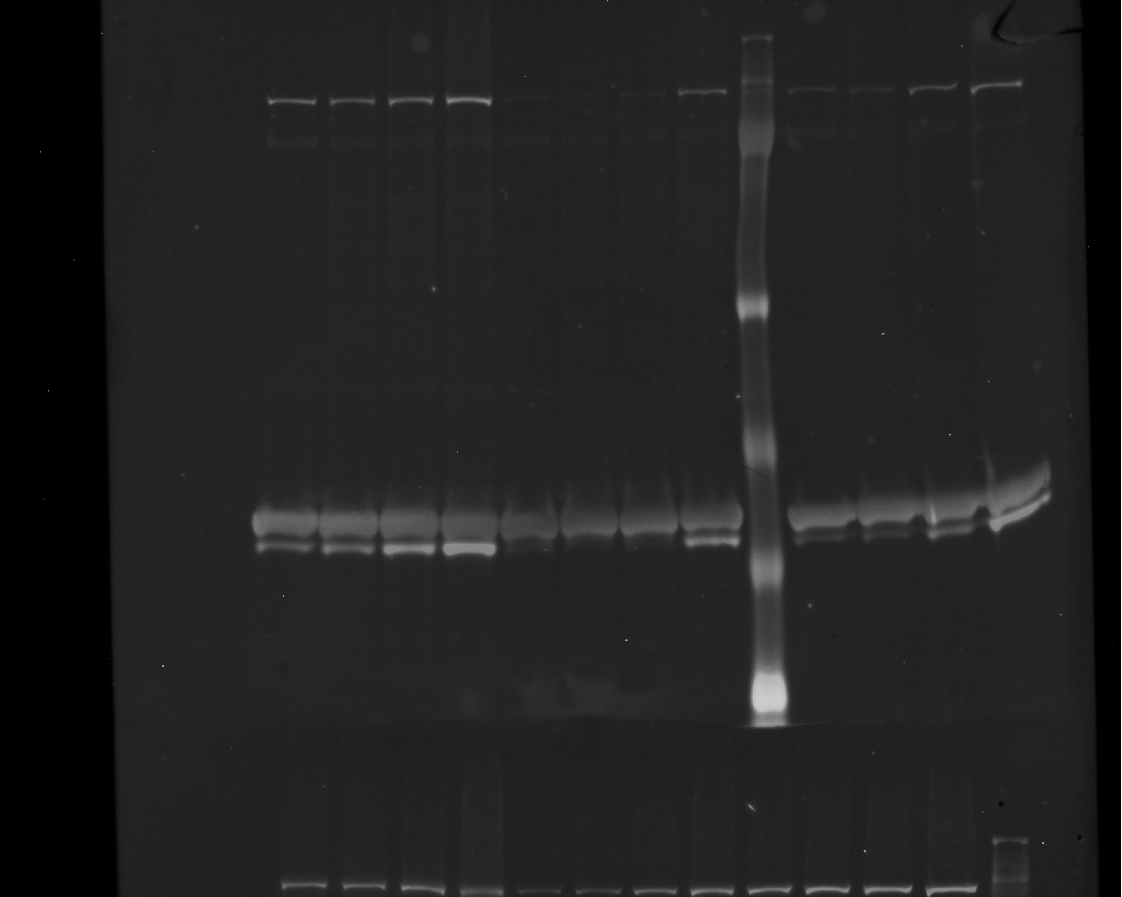

Supplement: S1 Data — (ZIP) [file pgen.1011059.s014.zip › SIdata/Figure 5 + S5 + S6/5 + S6 - RpoSLac/5+S6C_WB/20210224_RpoSLac/lmbchemidoc 2021-02-24 17h11m05s(Composite).jpg]

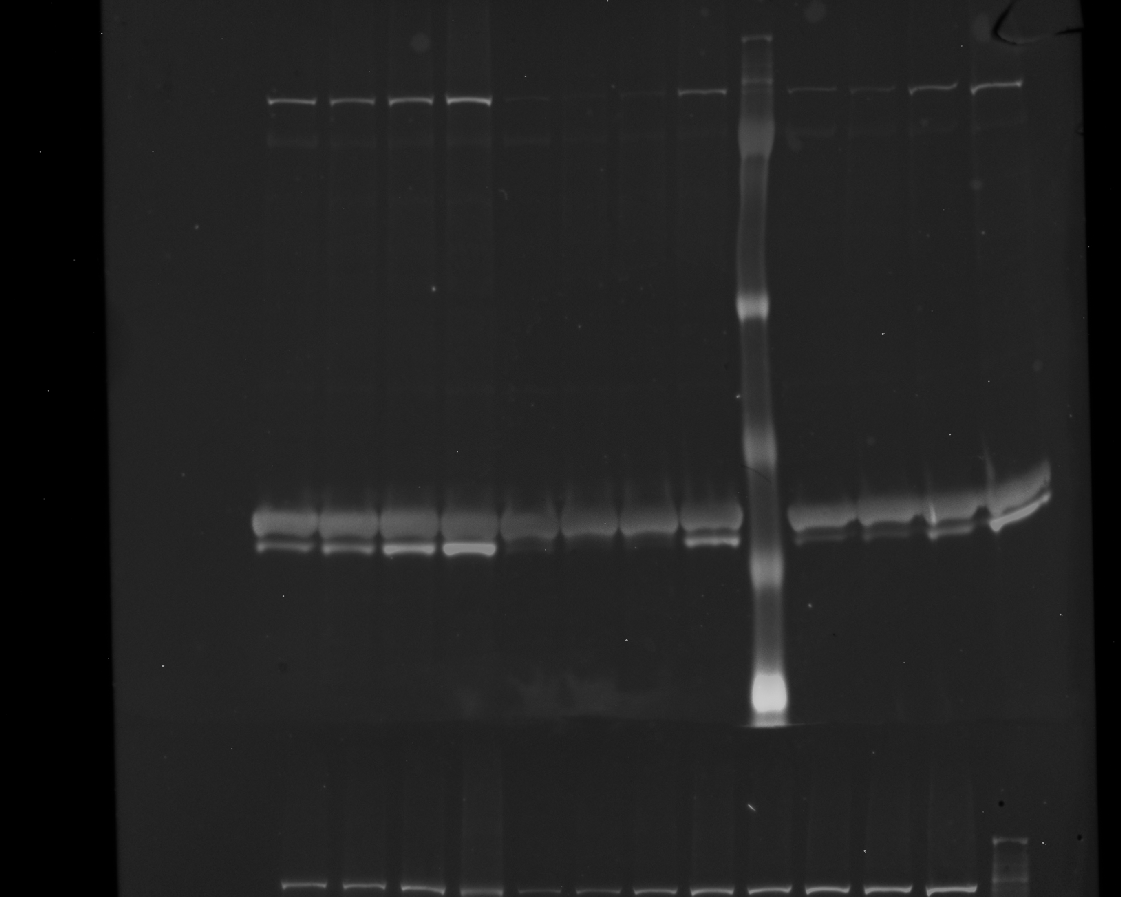

Supplement: S1 Data — (ZIP) [file pgen.1011059.s014.zip › SIdata/Figure 5 + S5 + S6/5 + S6 - RpoSLac/5+S6C_WB/20210224_RpoSLac/lmbchemidoc 2021-02-24 17h11m05s(Composite).tif]

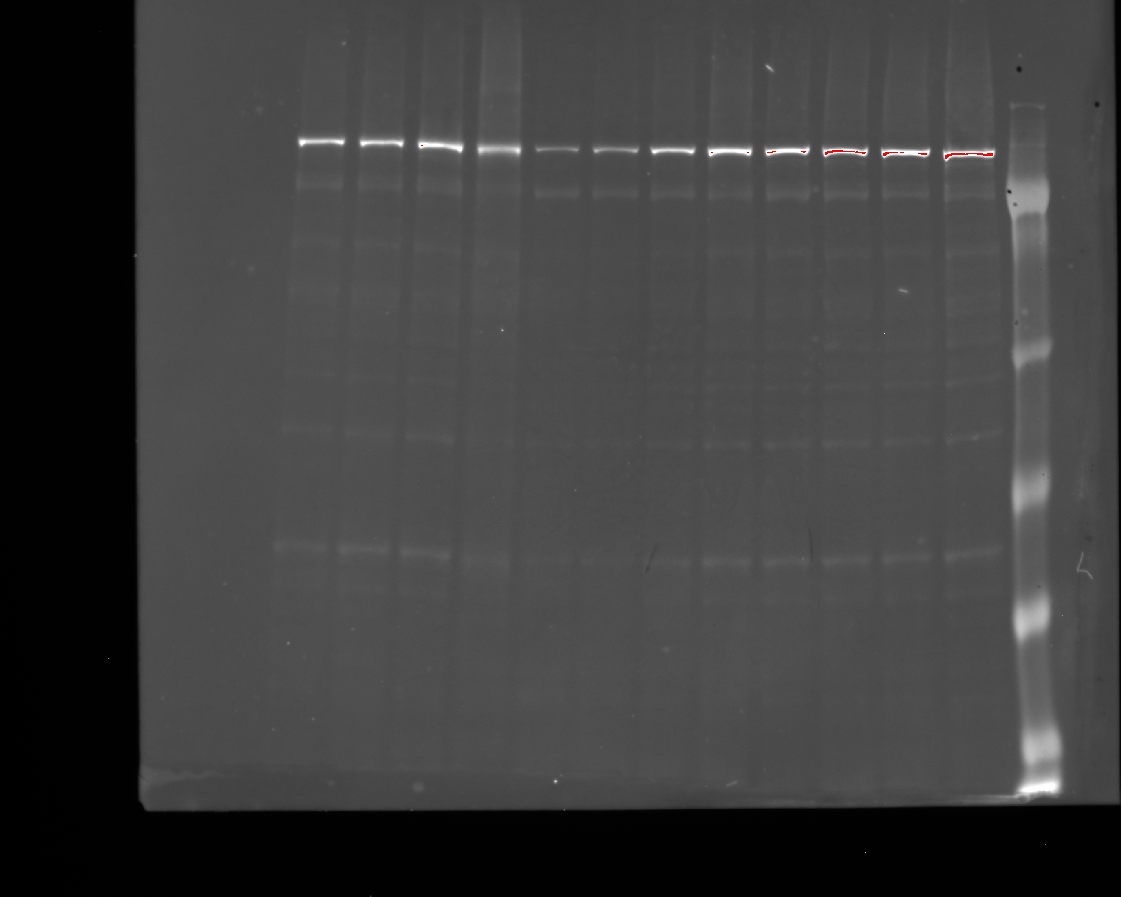

Supplement: S1 Data — (ZIP) [file pgen.1011059.s014.zip › SIdata/Figure 5 + S5 + S6/5 + S6 - RpoSLac/5+S6C_WB/20210224_RpoSLac/lmbchemidoc 2021-02-24 17h14m38s(StarBright B700).tif]

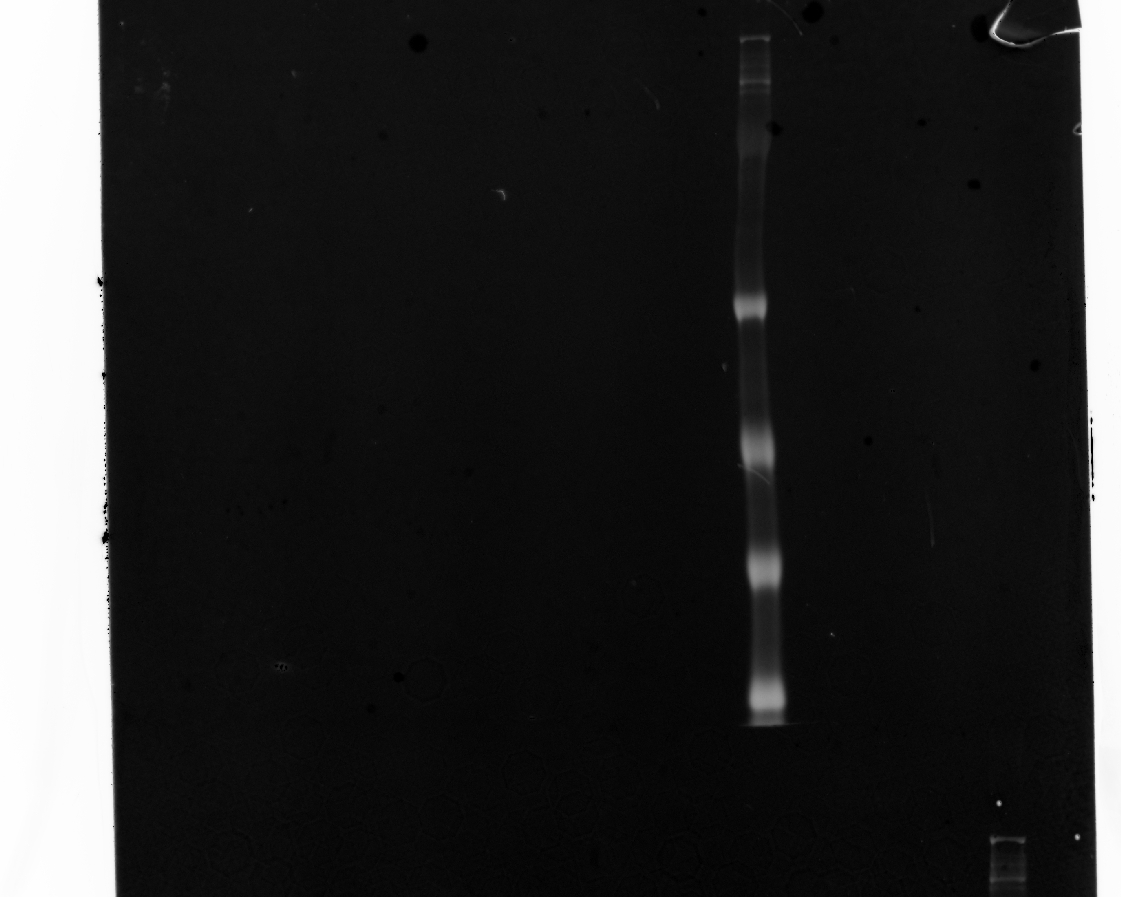

Supplement: S1 Data — (ZIP) [file pgen.1011059.s014.zip › SIdata/Figure 5 + S5 + S6/5 + S6 - RpoSLac/5+S6C_WB/20210224_RpoSLac/lmbchemidoc 2021-02-24 17h11m05s(Colorimetric).jpg]

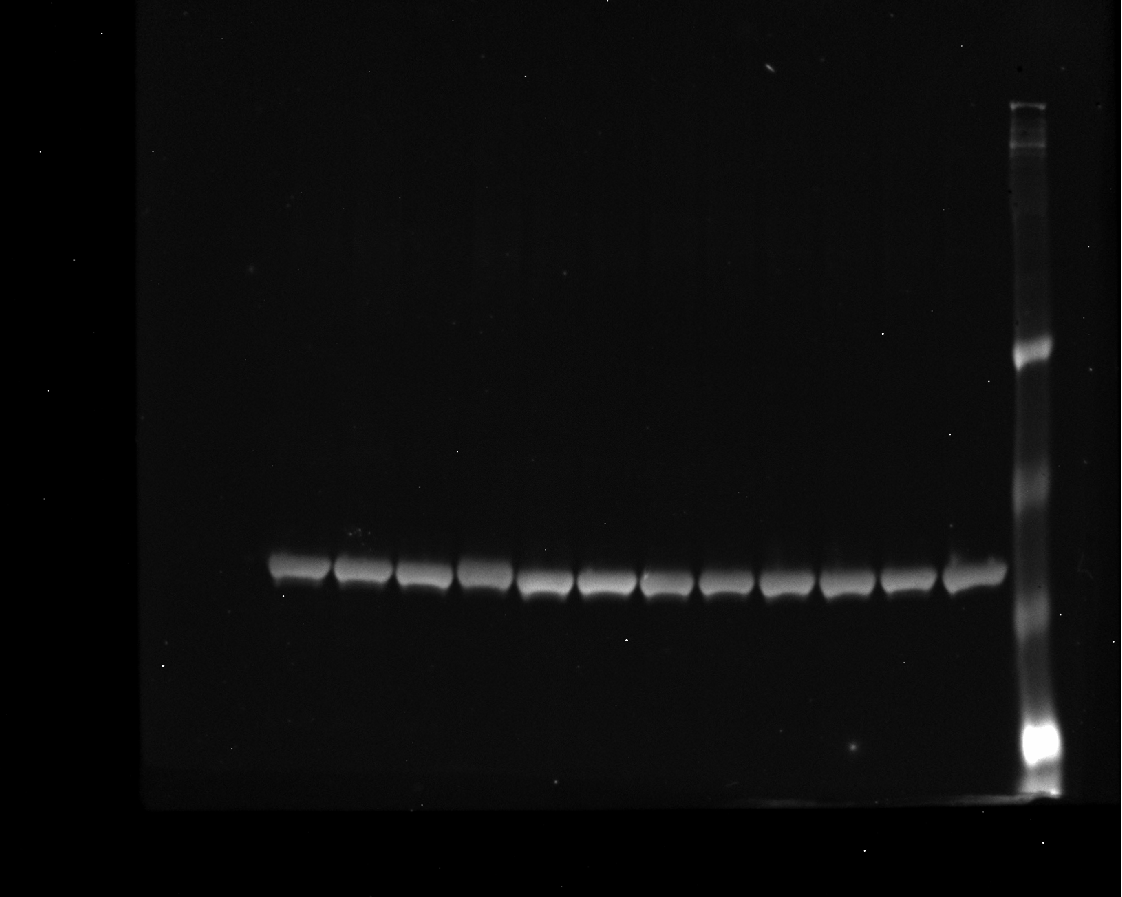

Supplement: S1 Data — (ZIP) [file pgen.1011059.s014.zip › SIdata/Figure 5 + S5 + S6/5 + S6 - RpoSLac/5+S6C_WB/20210224_RpoSLac/lmbchemidoc 2021-02-24 17h14m38s(DyLight 800).tif]

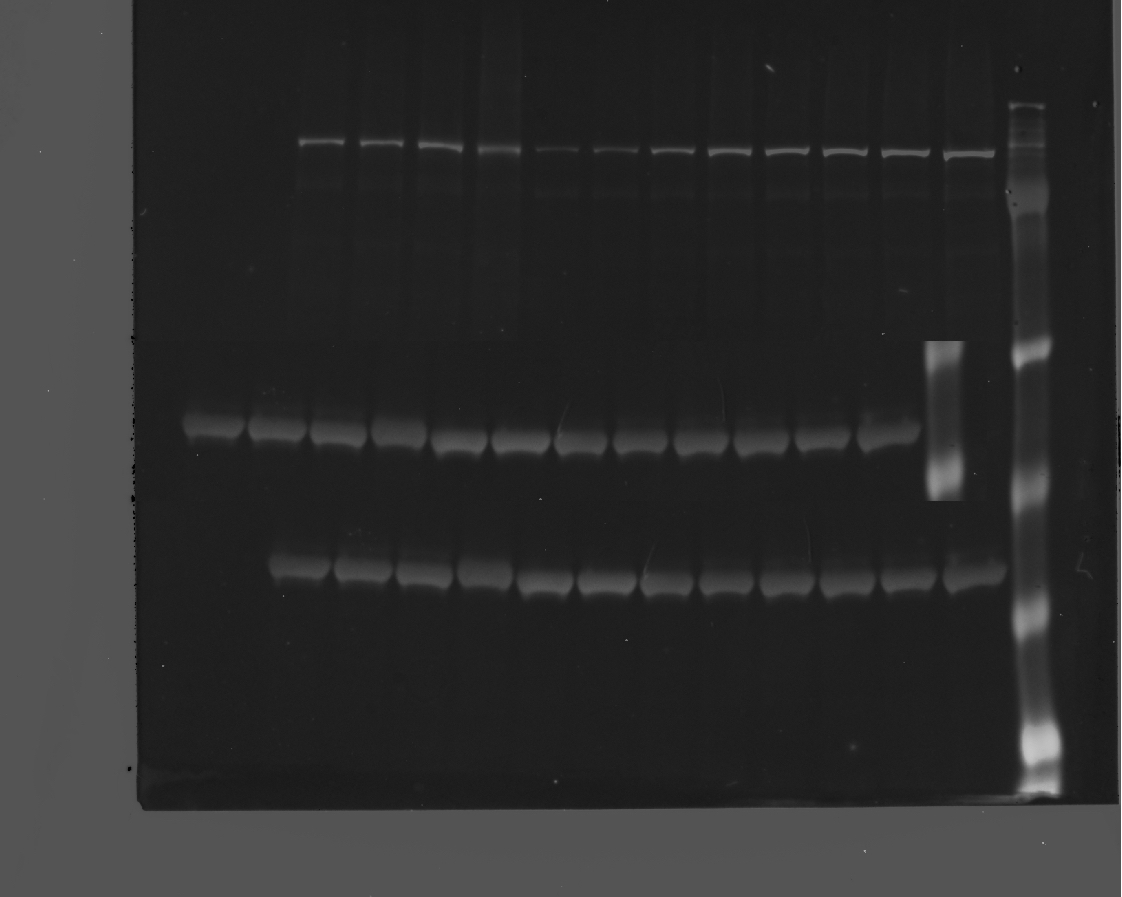

Supplement: S1 Data — (ZIP) [file pgen.1011059.s014.zip › SIdata/Figure 5 + S5 + S6/5 + S6 - RpoSLac/5+S6C_WB/20210224_RpoSLac/lmbchemidoc 2021-02-24 17h14m38s(Composite).jpg]

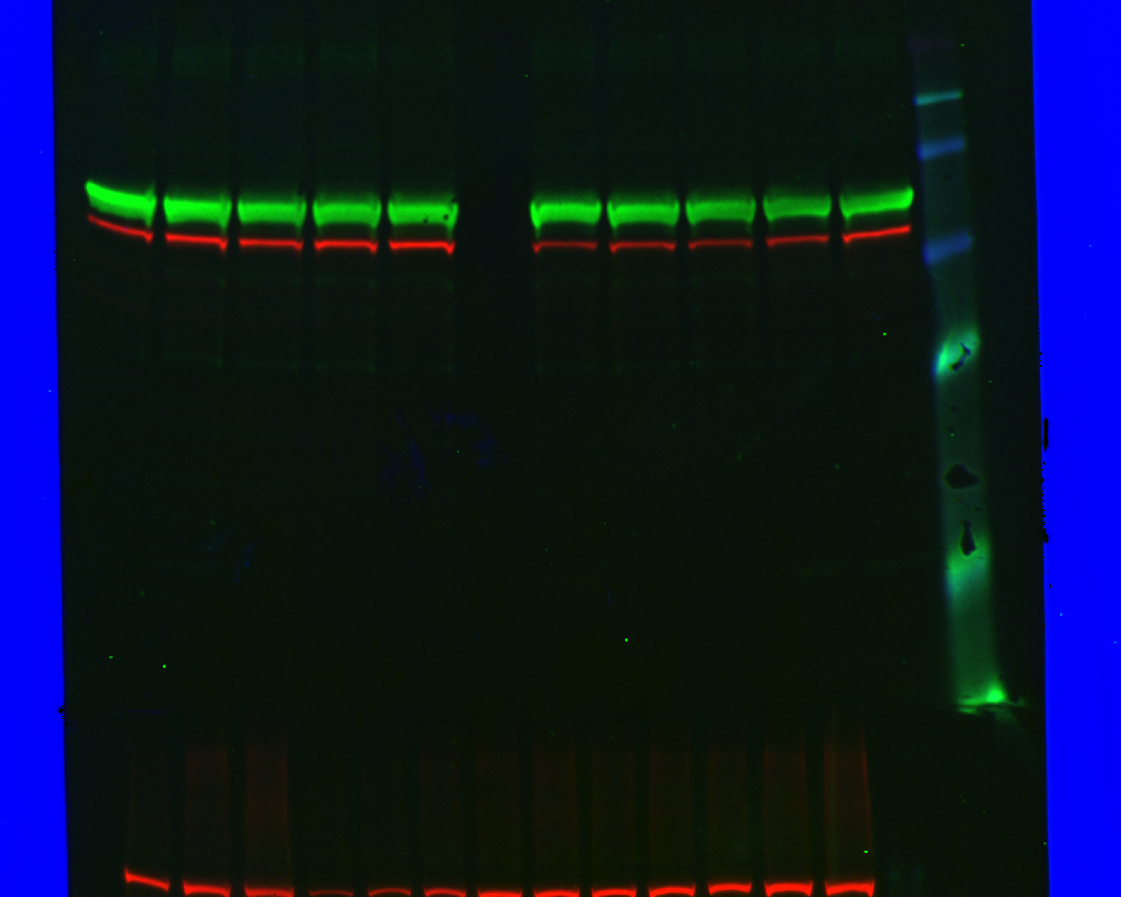

Supplement: S1 Data — (ZIP) [file pgen.1011059.s014.zip › SIdata/Figure 5 + S5 + S6/5 + S6 - RpoSLac/5+S6C_WB/20210218_RpoSLac/lmbchemidoc 2021-02-18 16h23m29s(Composite).tif]

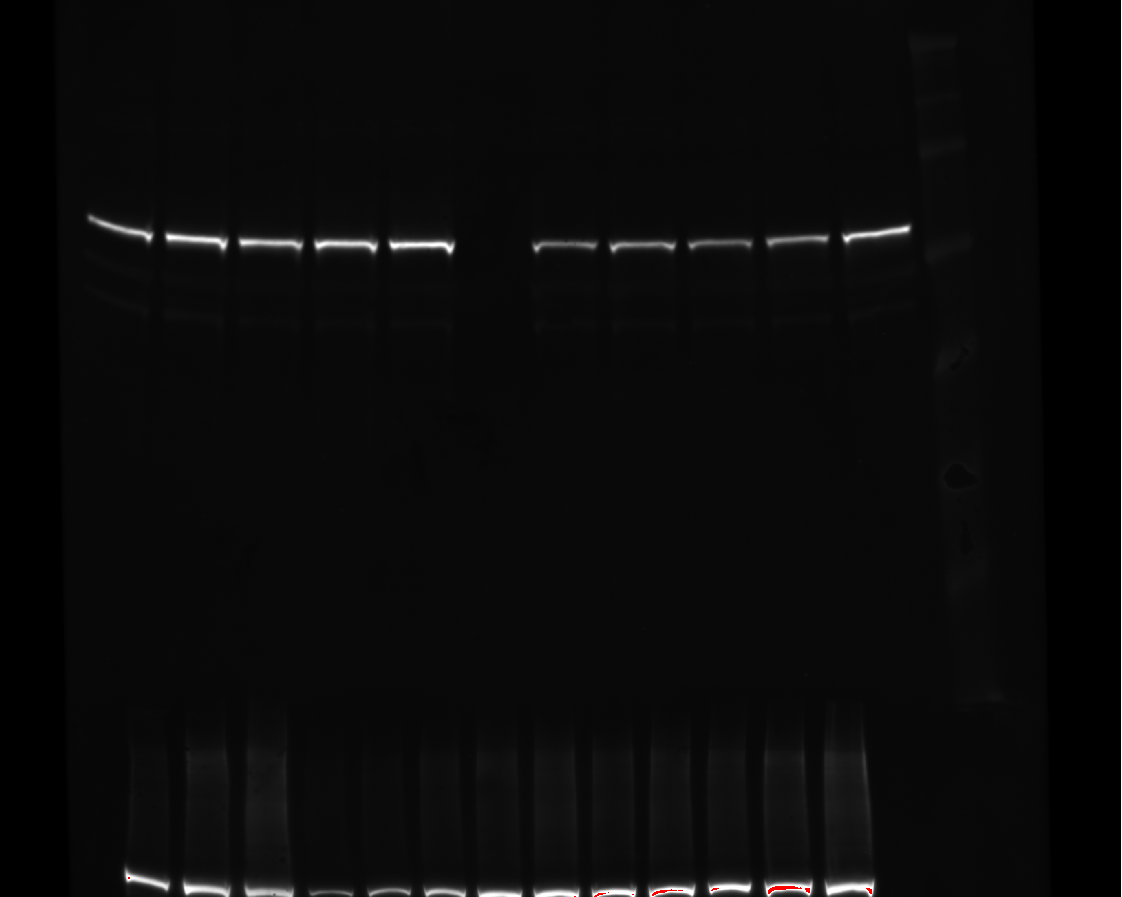

Supplement: S1 Data — (ZIP) [file pgen.1011059.s014.zip › SIdata/Figure 5 + S5 + S6/5 + S6 - RpoSLac/5+S6C_WB/20210218_RpoSLac/lmbchemidoc 2021-02-18 16h23m29s(StarBright B700).tif]

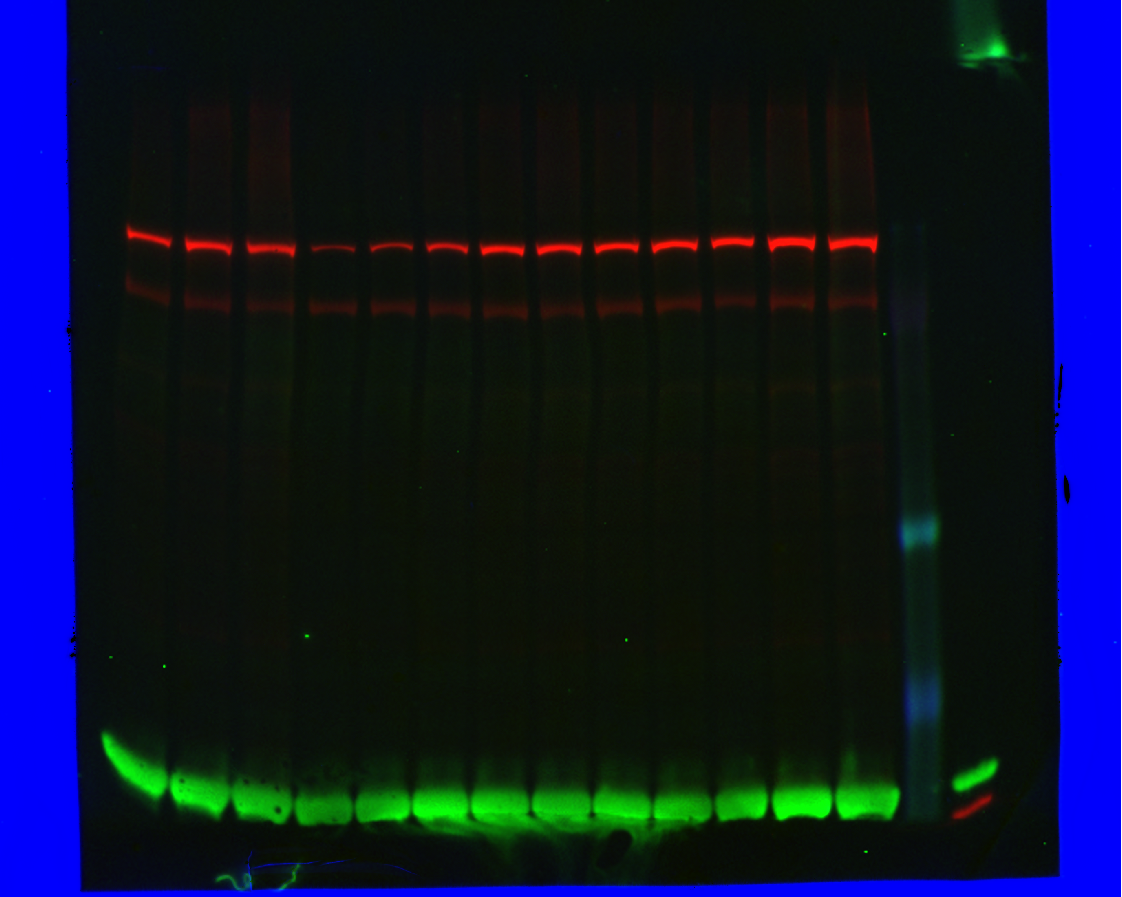

Supplement: S1 Data — (ZIP) [file pgen.1011059.s014.zip › SIdata/Figure 5 + S5 + S6/5 + S6 - RpoSLac/5+S6C_WB/20210218_RpoSLac/lmbchemidoc 2021-02-18 16h20m38s(Composite).tif]

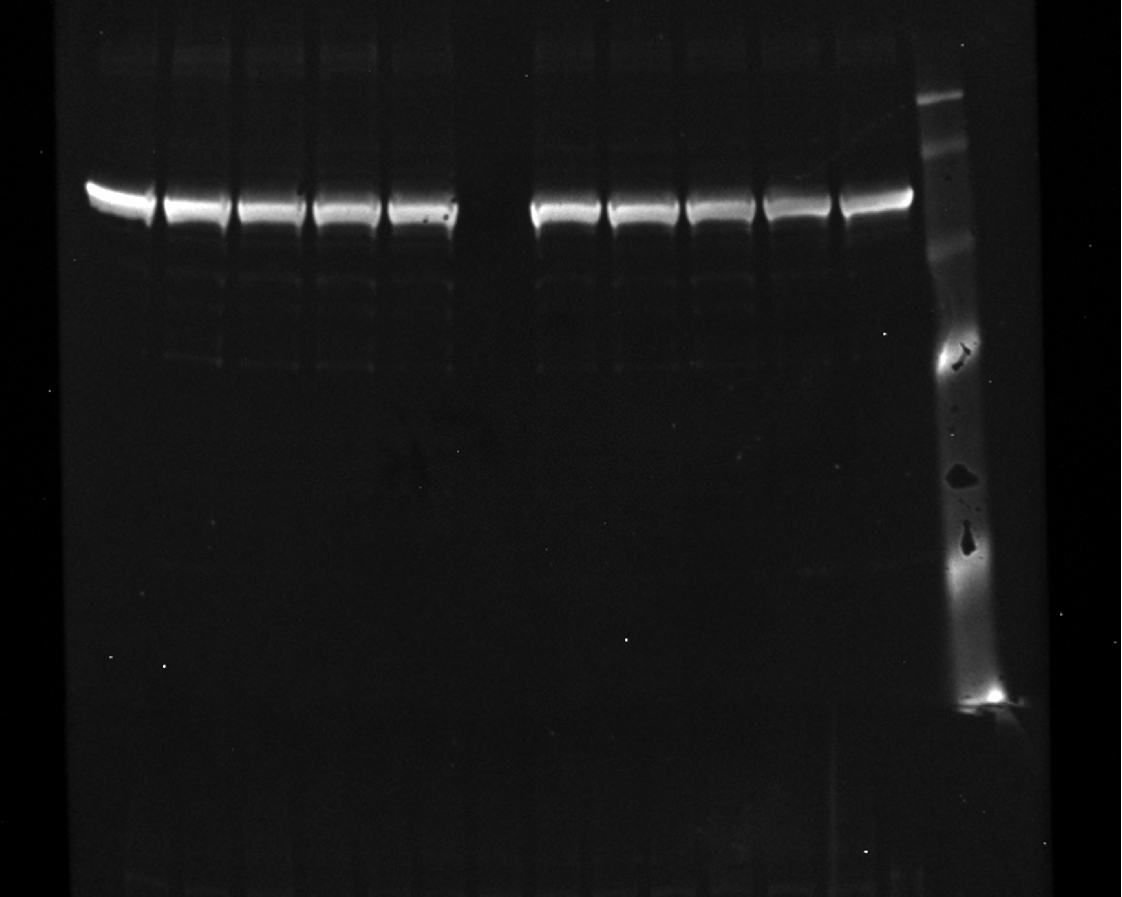

Supplement: S1 Data — (ZIP) [file pgen.1011059.s014.zip › SIdata/Figure 5 + S5 + S6/5 + S6 - RpoSLac/5+S6C_WB/20210218_RpoSLac/lmbchemidoc 2021-02-18 16h23m29s(DyLight 800).jpg]

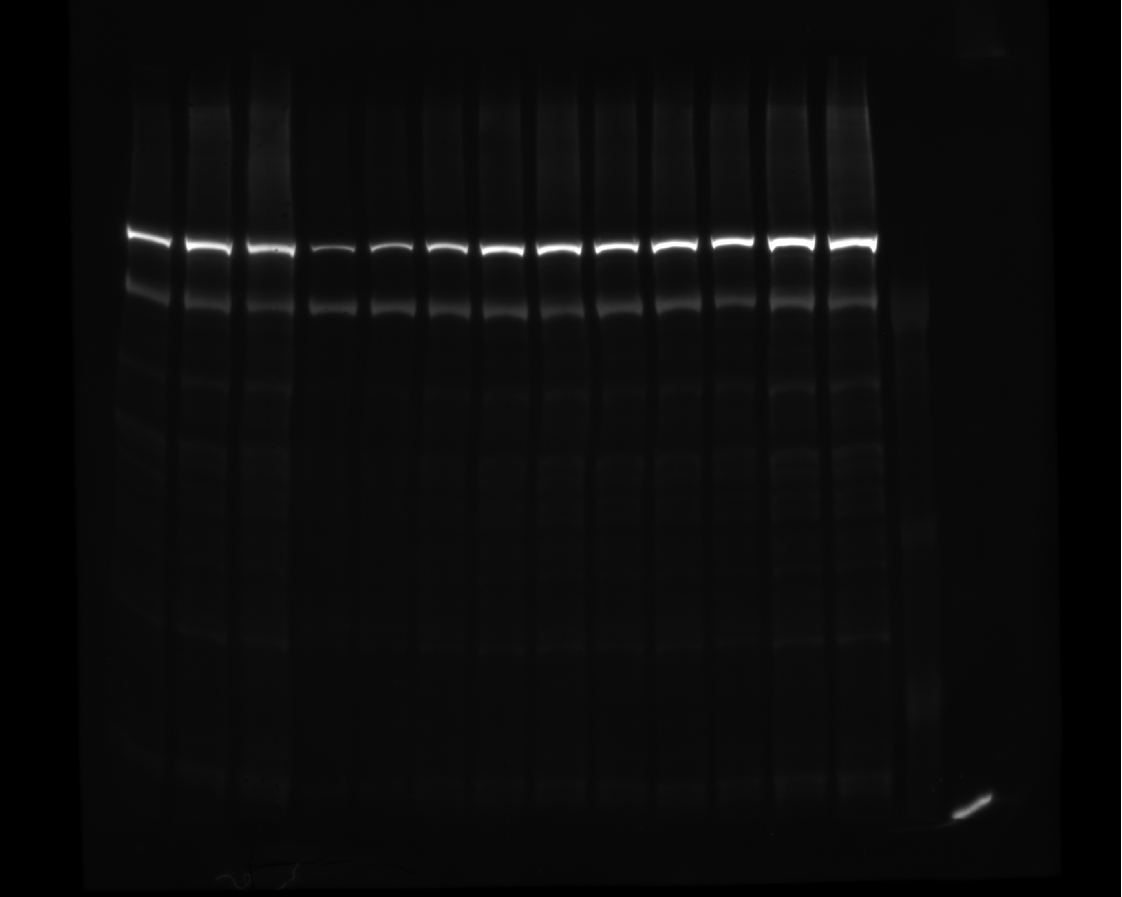

Supplement: S1 Data — (ZIP) [file pgen.1011059.s014.zip › SIdata/Figure 5 + S5 + S6/5 + S6 - RpoSLac/5+S6C_WB/20210218_RpoSLac/lmbchemidoc 2021-02-18 16h20m38s(StarBright B700).jpg]

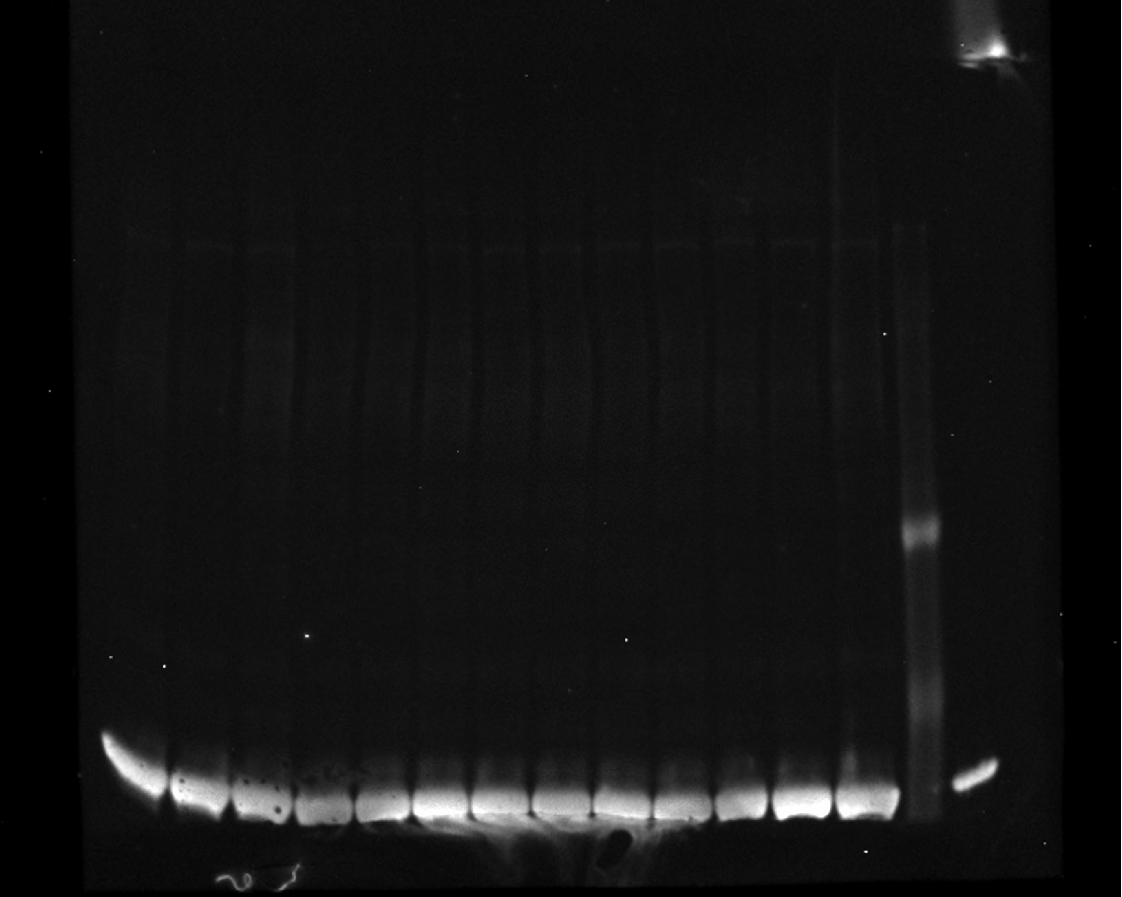

Supplement: S1 Data — (ZIP) [file pgen.1011059.s014.zip › SIdata/Figure 5 + S5 + S6/5 + S6 - RpoSLac/5+S6C_WB/20210218_RpoSLac/lmbchemidoc 2021-02-18 16h20m38s(DyLight 800).jpg]

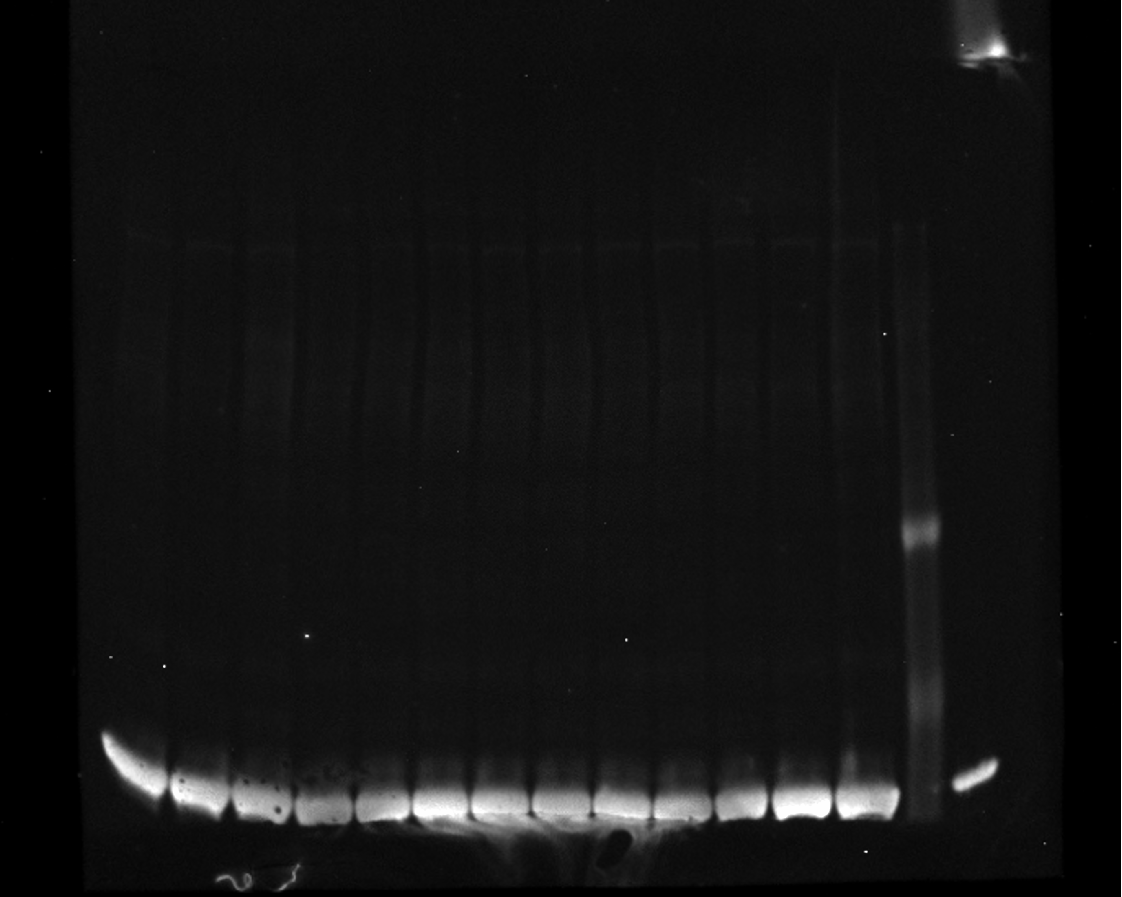

Supplement: S1 Data — (ZIP) [file pgen.1011059.s014.zip › SIdata/Figure 5 + S5 + S6/5 + S6 - RpoSLac/5+S6C_WB/20210218_RpoSLac/lmbchemidoc 2021-02-18 16h20m38s(DyLight 800).tif]

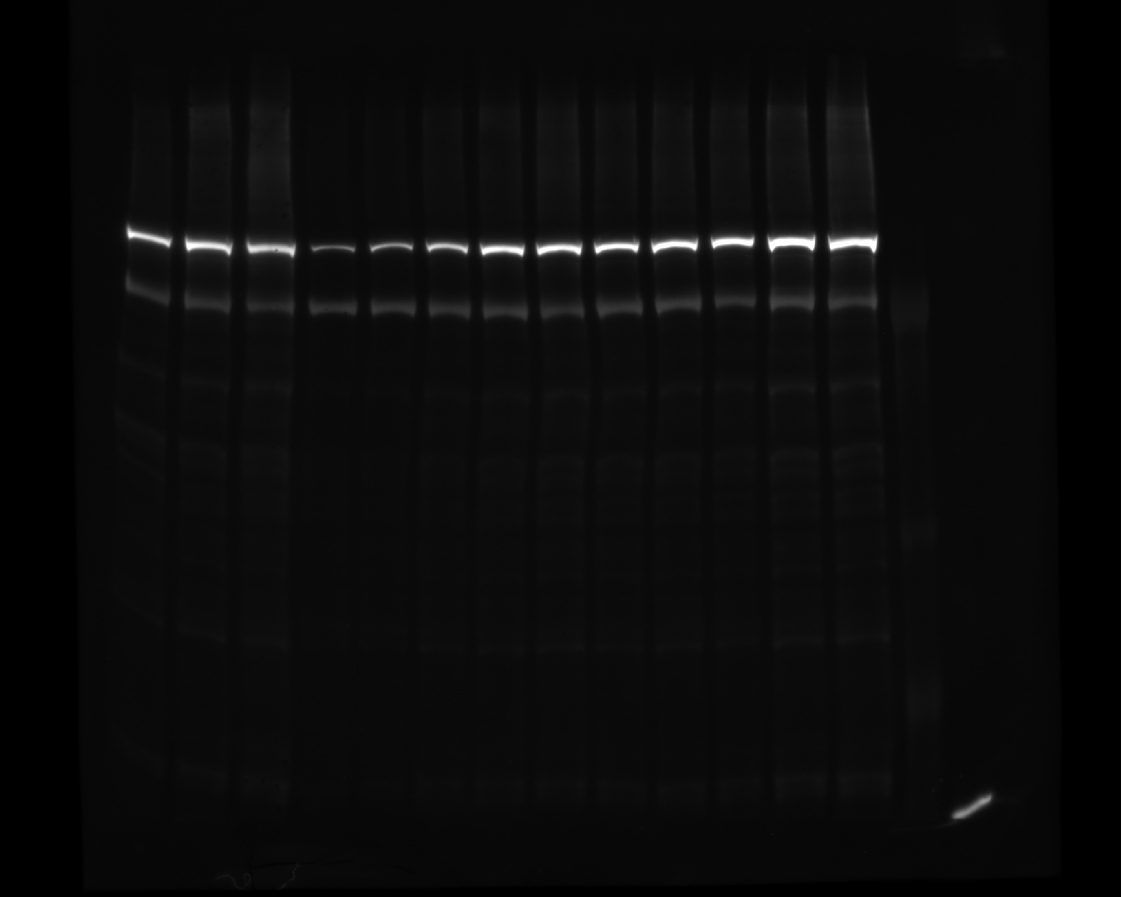

Supplement: S1 Data — (ZIP) [file pgen.1011059.s014.zip › SIdata/Figure 5 + S5 + S6/5 + S6 - RpoSLac/5+S6C_WB/20210218_RpoSLac/lmbchemidoc 2021-02-18 16h20m38s(StarBright B700).tif]

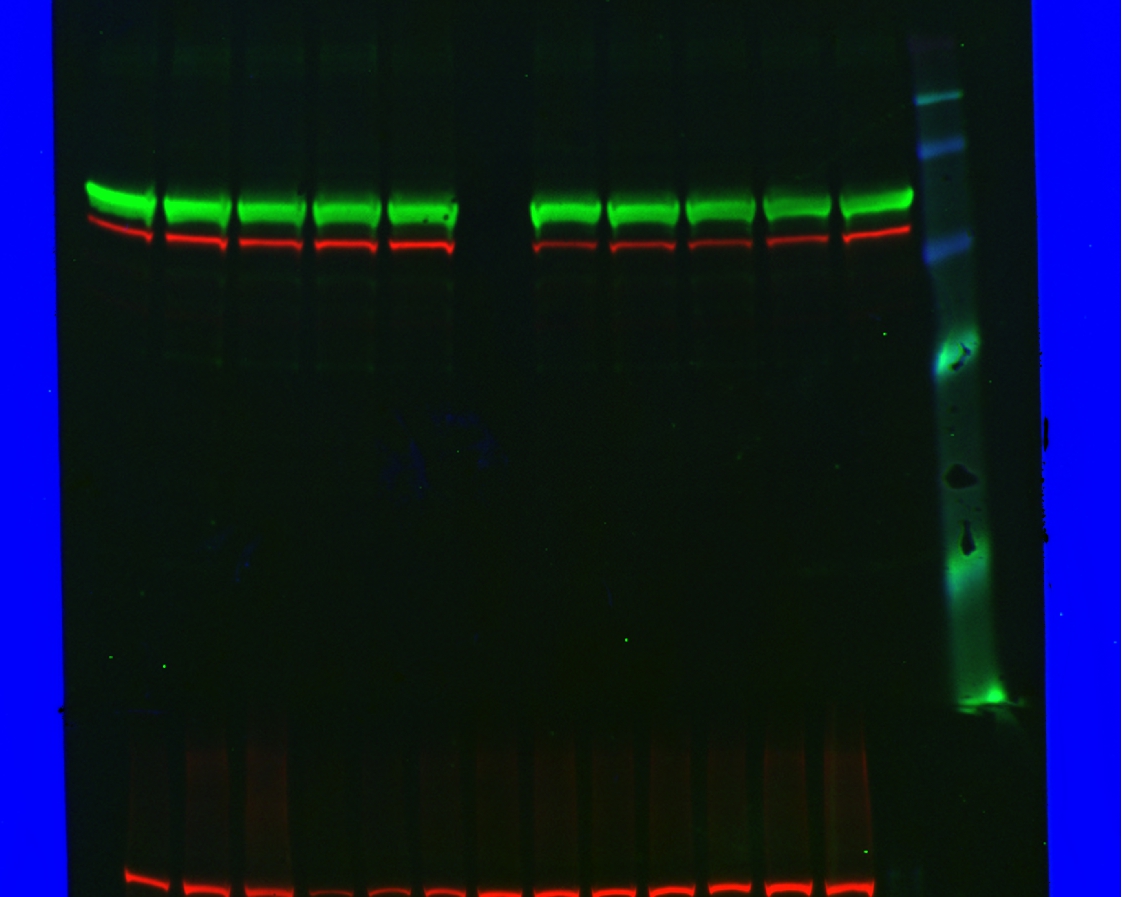

Supplement: S1 Data — (ZIP) [file pgen.1011059.s014.zip › SIdata/Figure 5 + S5 + S6/5 + S6 - RpoSLac/5+S6C_WB/20210218_RpoSLac/lmbchemidoc 2021-02-18 16h23m29s(Composite).jpg]

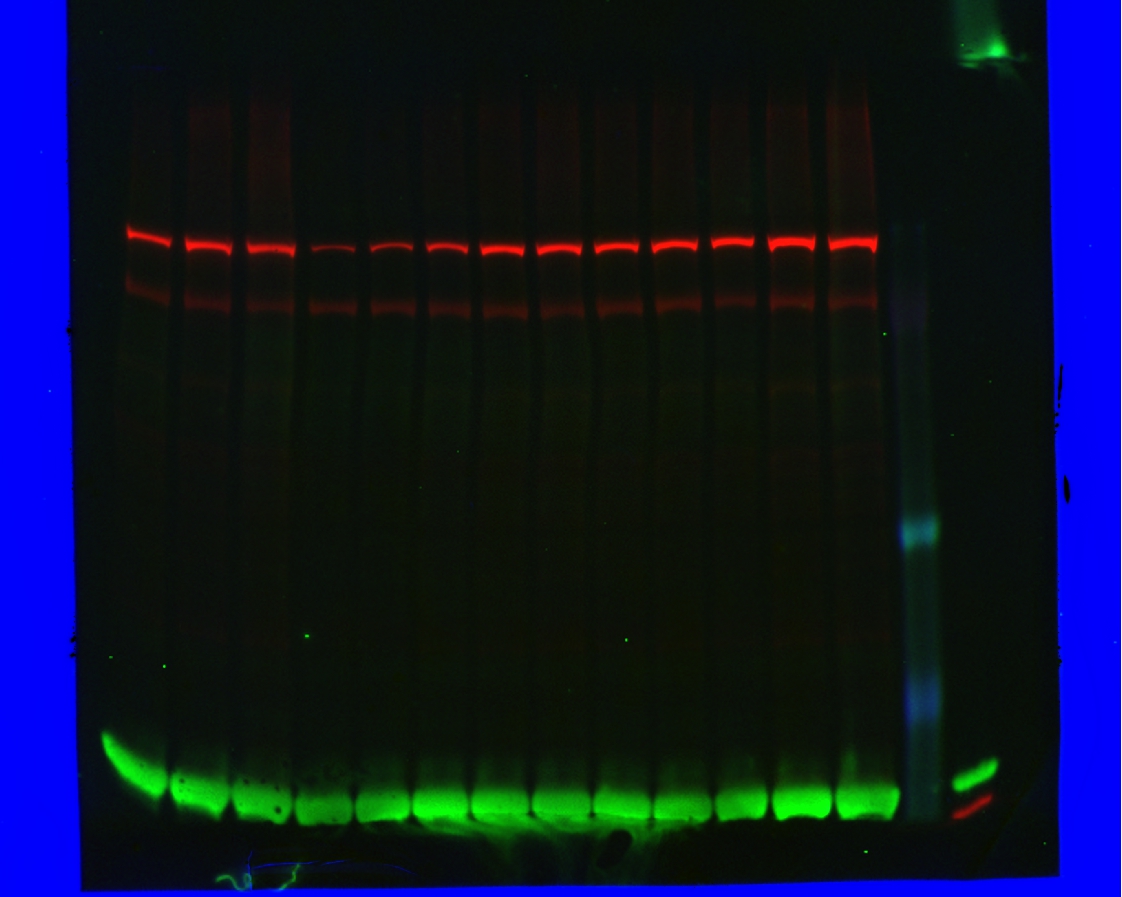

Supplement: S1 Data — (ZIP) [file pgen.1011059.s014.zip › SIdata/Figure 5 + S5 + S6/5 + S6 - RpoSLac/5+S6C_WB/20210218_RpoSLac/lmbchemidoc 2021-02-18 16h20m38s(Composite).jpg]

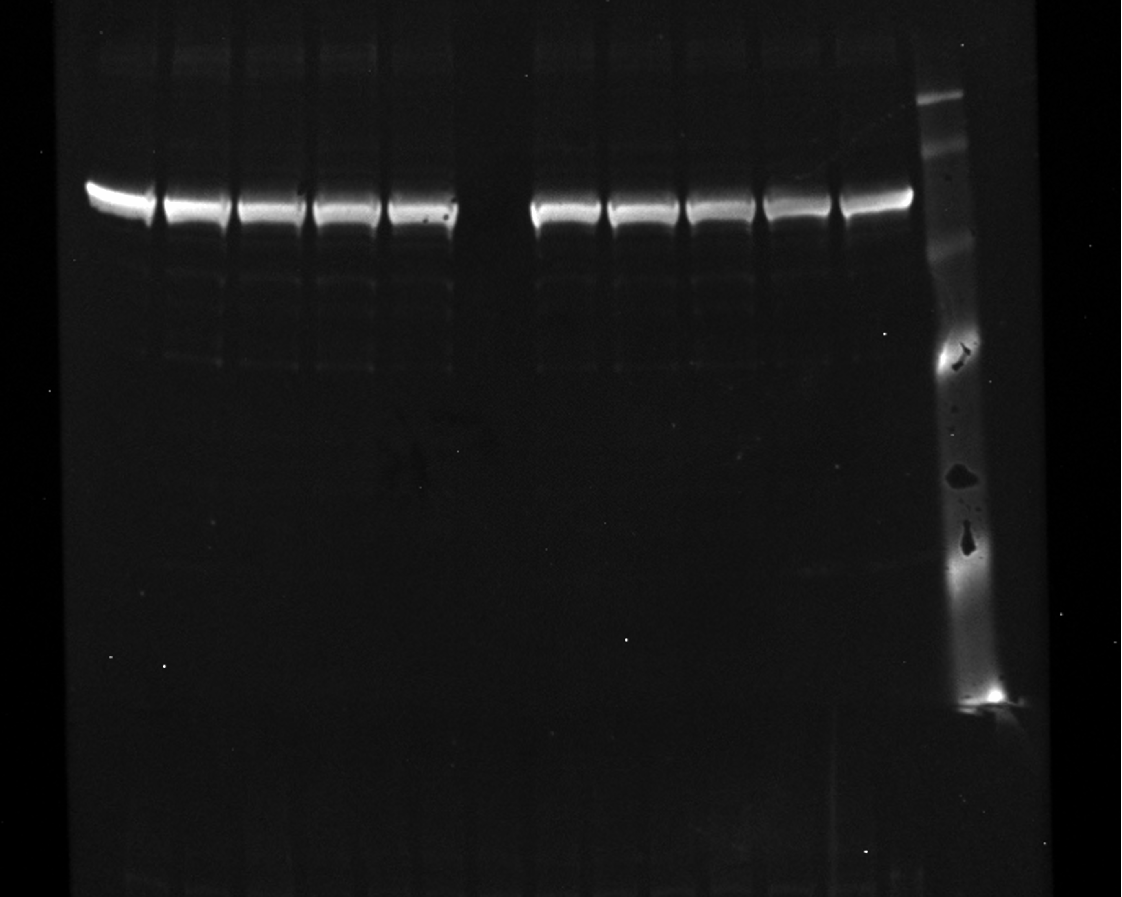

Supplement: S1 Data — (ZIP) [file pgen.1011059.s014.zip › SIdata/Figure 5 + S5 + S6/5 + S6 - RpoSLac/5+S6C_WB/20210218_RpoSLac/lmbchemidoc 2021-02-18 16h23m29s(DyLight 800).tif]

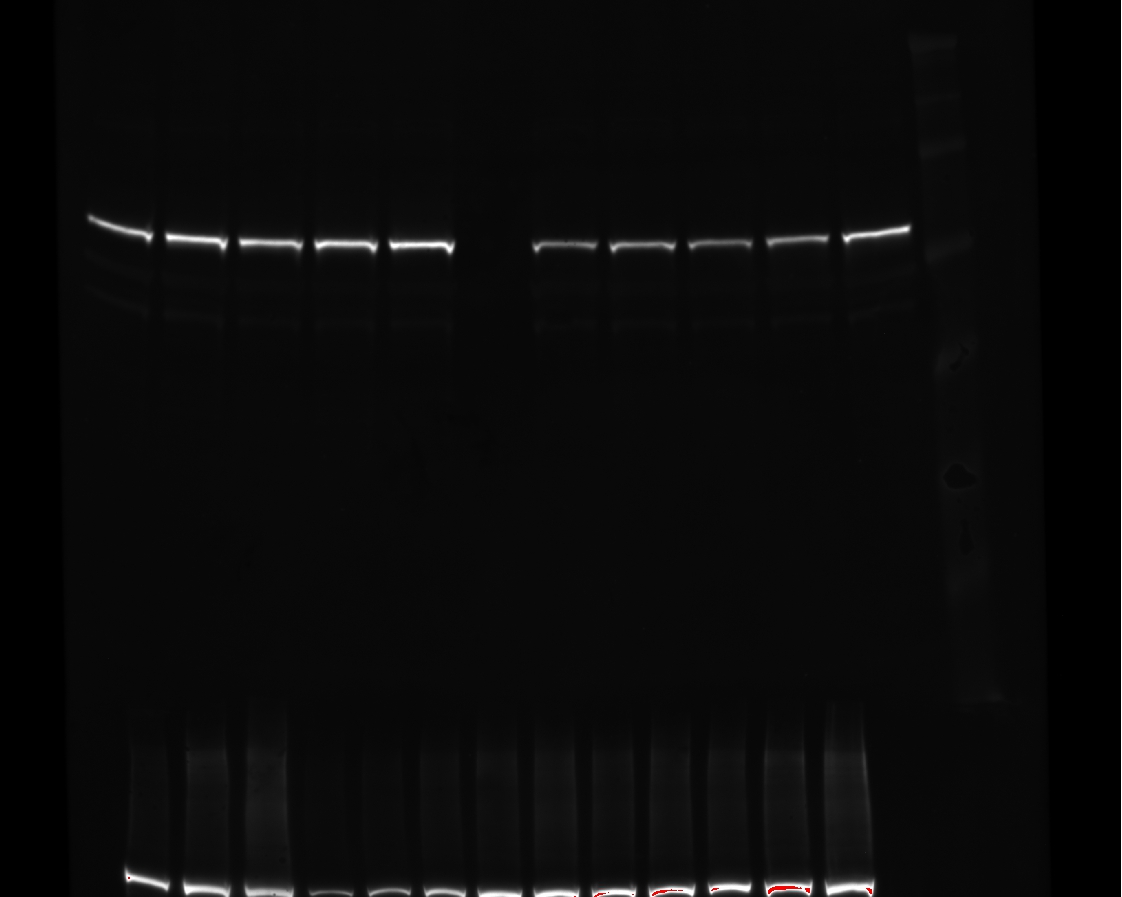

Supplement: S1 Data — (ZIP) [file pgen.1011059.s014.zip › SIdata/Figure 5 + S5 + S6/5 + S6 - RpoSLac/5+S6C_WB/20210218_RpoSLac/lmbchemidoc 2021-02-18 16h23m29s(StarBright B700).jpg]

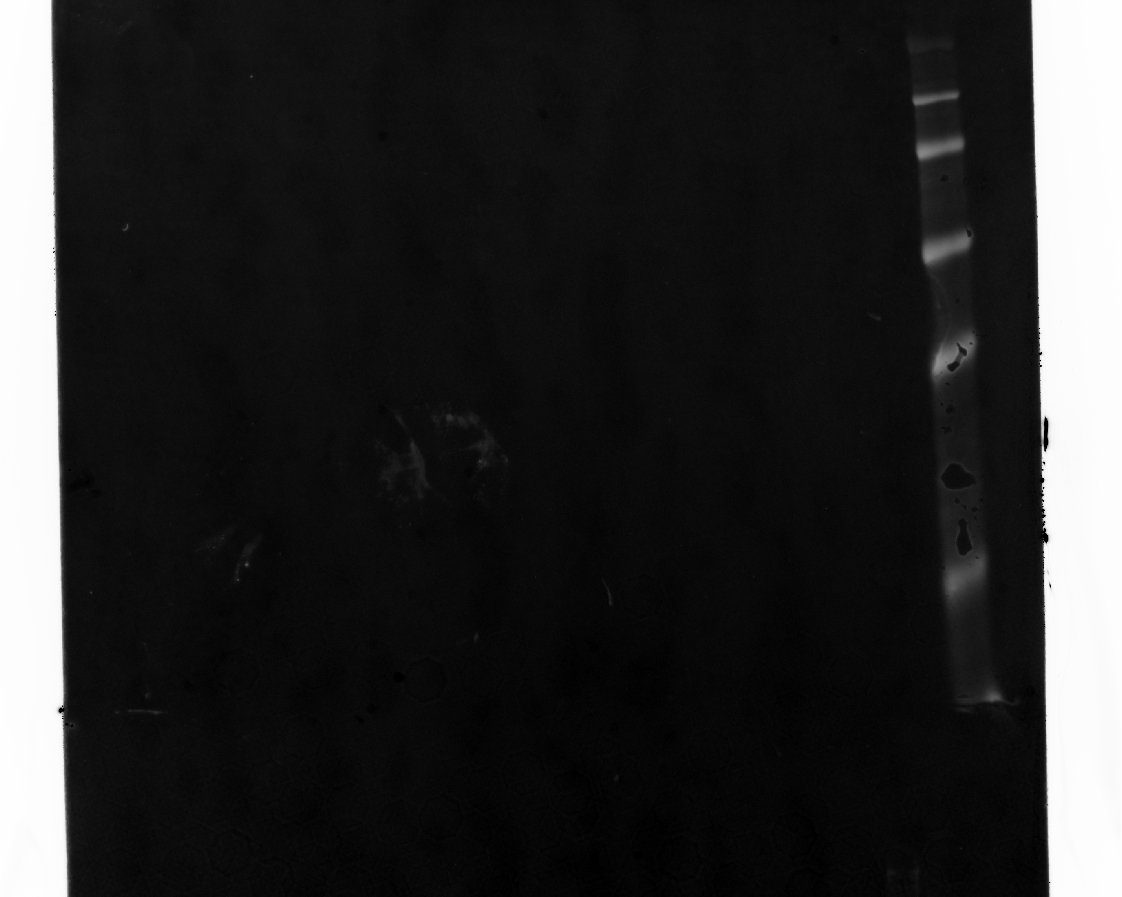

Supplement: S1 Data — (ZIP) [file pgen.1011059.s014.zip › SIdata/Figure 5 + S5 + S6/5 + S6 - RpoSLac/5+S6C_WB/20210218_RpoSLac/lmbchemidoc 2021-02-18 16h23m29s(Colorimetric).jpg]

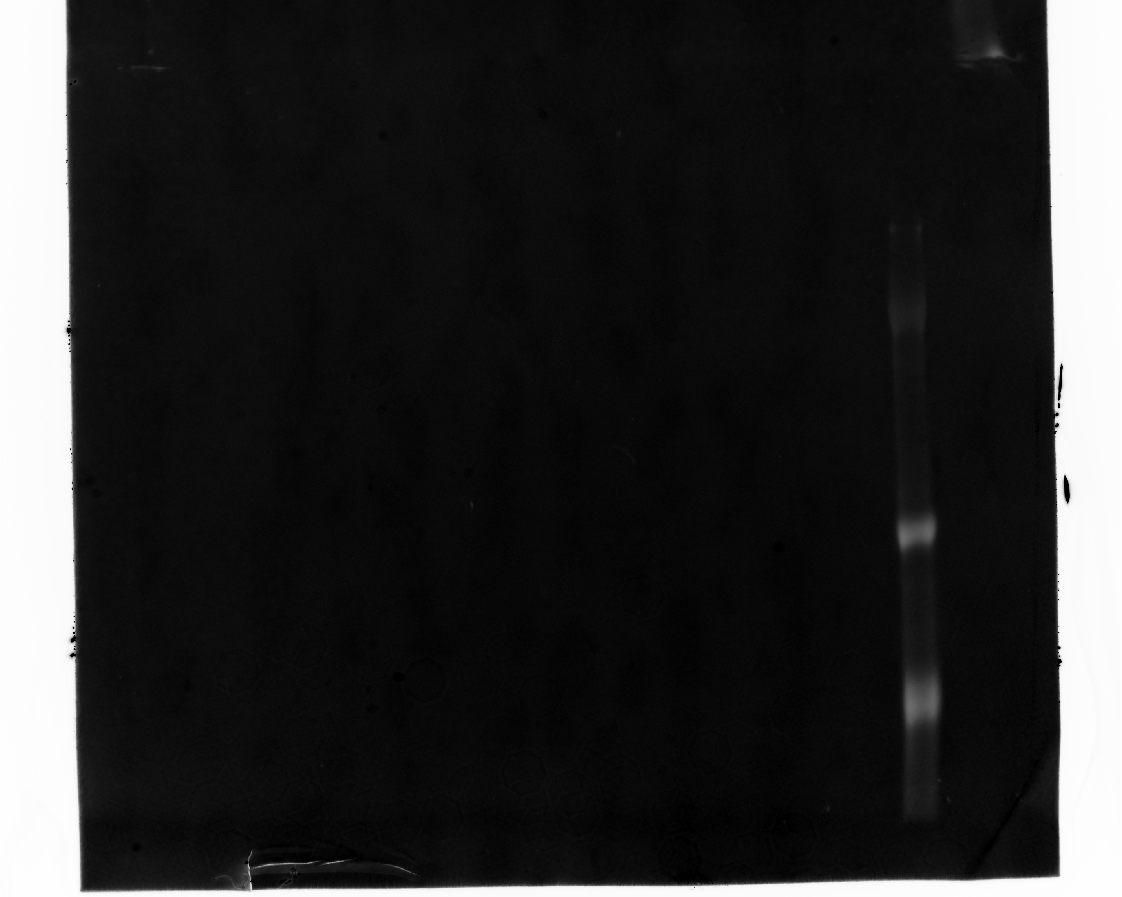

Supplement: S1 Data — (ZIP) [file pgen.1011059.s014.zip › SIdata/Figure 5 + S5 + S6/5 + S6 - RpoSLac/5+S6C_WB/20210218_RpoSLac/lmbchemidoc 2021-02-18 16h20m38s(Colorimetric).tif]

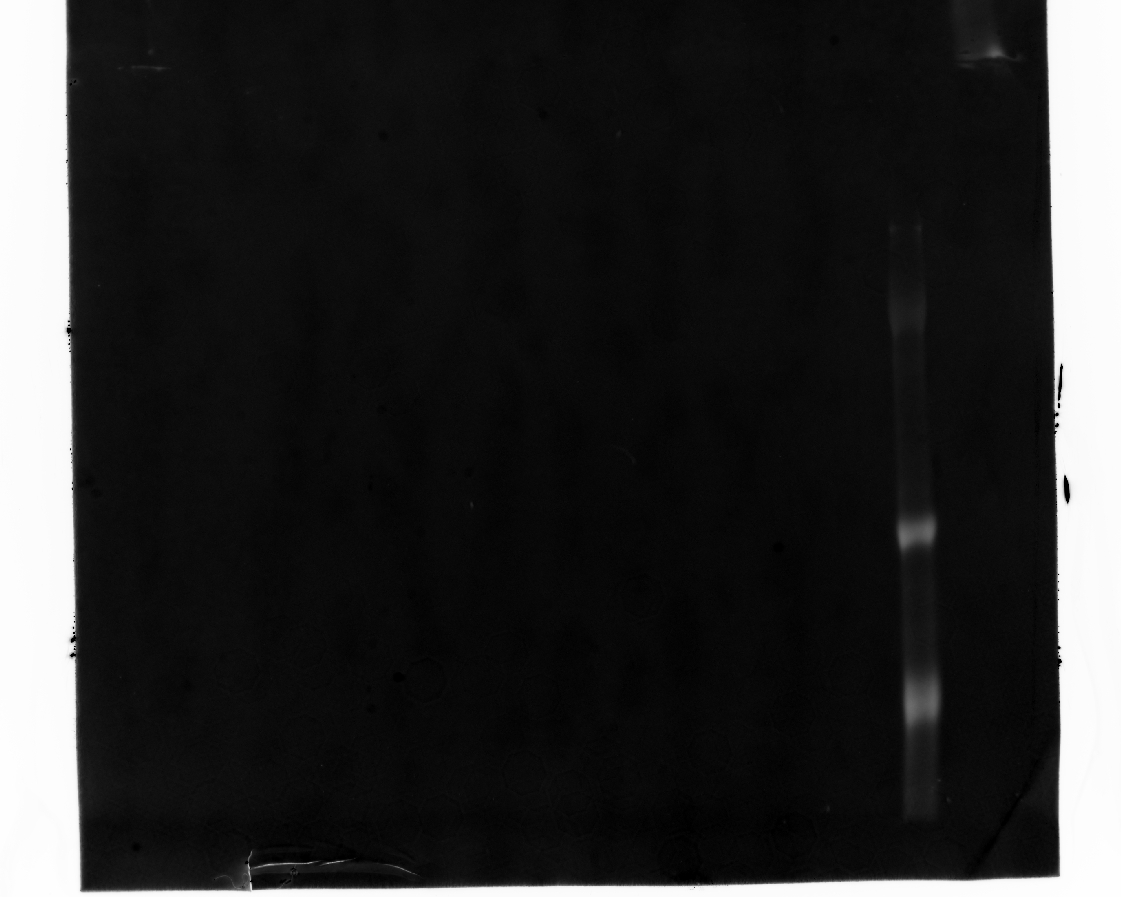

Supplement: S1 Data — (ZIP) [file pgen.1011059.s014.zip › SIdata/Figure 5 + S5 + S6/5 + S6 - RpoSLac/5+S6C_WB/20210218_RpoSLac/lmbchemidoc 2021-02-18 16h20m38s(Colorimetric).jpg]

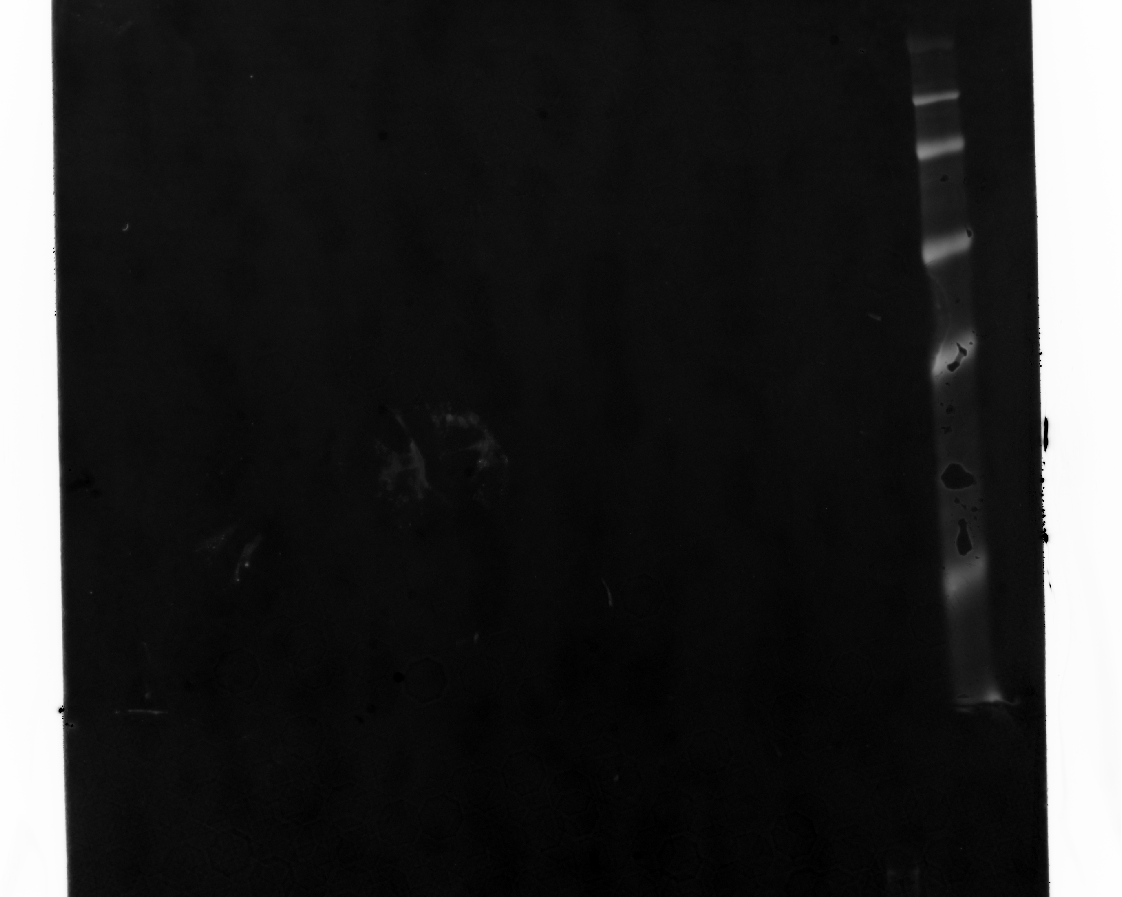

Supplement: S1 Data — (ZIP) [file pgen.1011059.s014.zip › SIdata/Figure 5 + S5 + S6/5 + S6 - RpoSLac/5+S6C_WB/20210218_RpoSLac/lmbchemidoc 2021-02-18 16h23m29s(Colorimetric).tif]

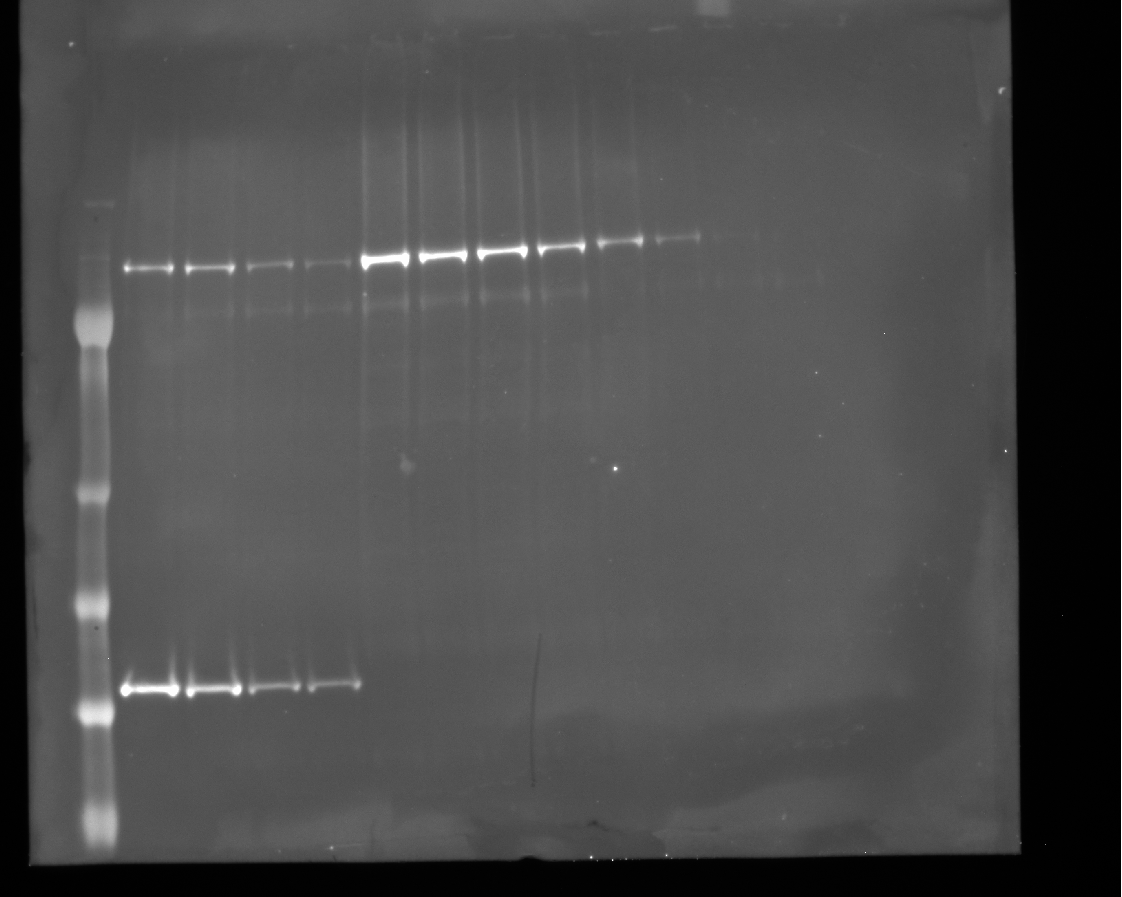

Supplement: S1 Data — (ZIP) [file pgen.1011059.s014.zip › SIdata/Figure 5 + S5 + S6/5 + S6 - RpoSLac/5+S6C_WB/20201221_RpoSLac recovery/lmbchemidoc 2020-12-21 18h58m19s(StarBright B700).tif]

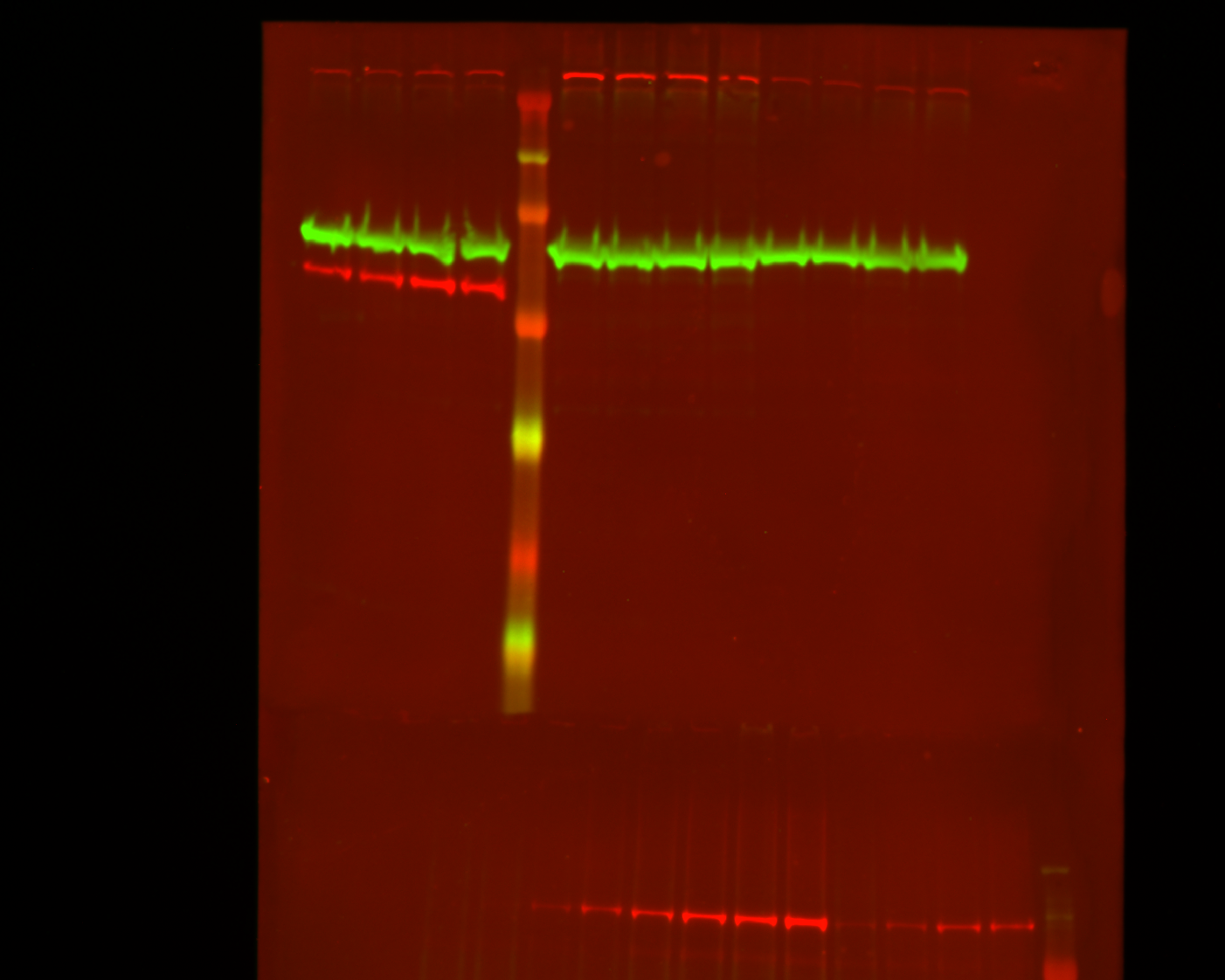

Supplement: S1 Data — (ZIP) [file pgen.1011059.s014.zip › SIdata/Figure 5 + S5 + S6/5 + S6 - RpoSLac/5+S6C_WB/20201221_RpoSLac recovery/lmbchemidoc 2020-12-21 18h55m38s(Composite).tif]

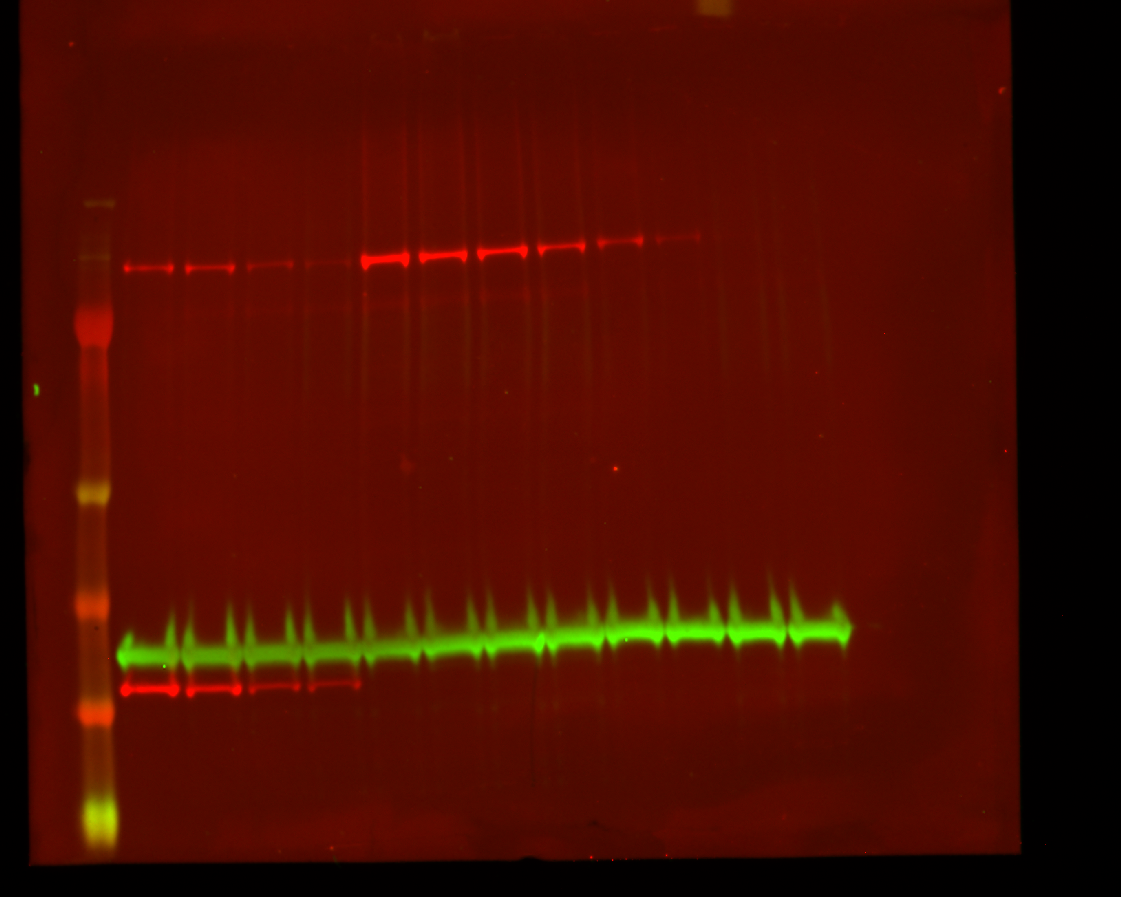

Supplement: S1 Data — (ZIP) [file pgen.1011059.s014.zip › SIdata/Figure 5 + S5 + S6/5 + S6 - RpoSLac/5+S6C_WB/20201221_RpoSLac recovery/lmbchemidoc 2020-12-21 18h58m19s(Composite).tif]

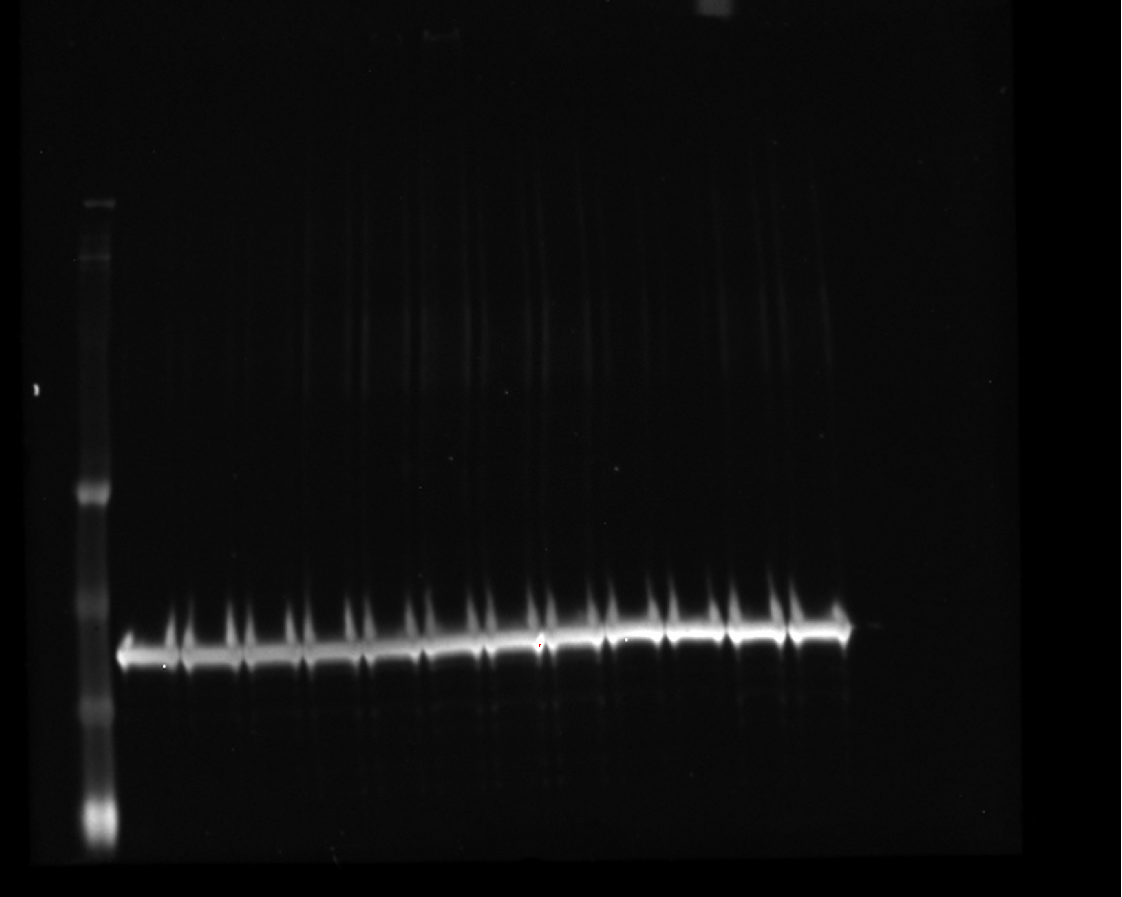

Supplement: S1 Data — (ZIP) [file pgen.1011059.s014.zip › SIdata/Figure 5 + S5 + S6/5 + S6 - RpoSLac/5+S6C_WB/20201221_RpoSLac recovery/lmbchemidoc 2020-12-21 18h58m19s(DyLight 800).tif]

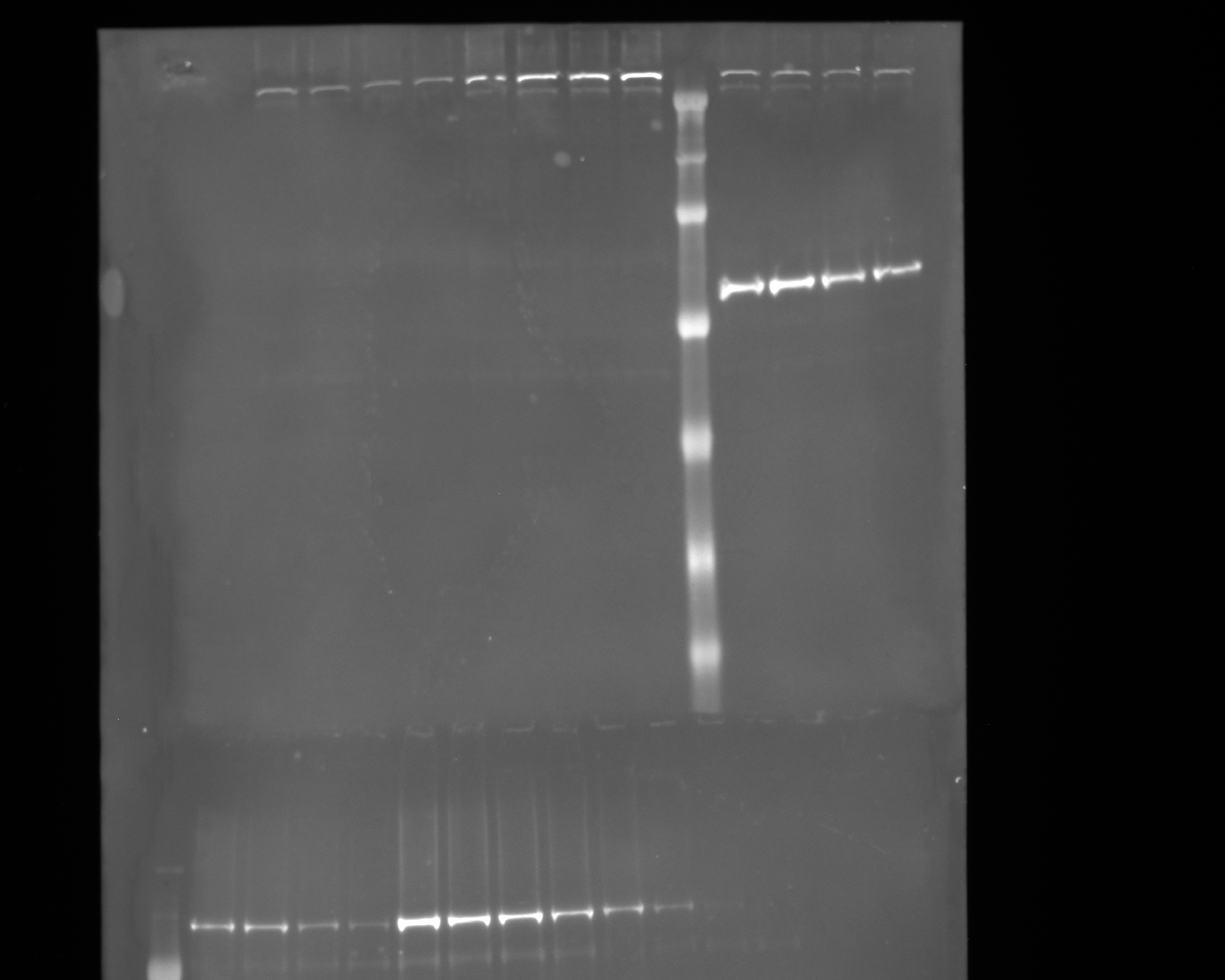

Supplement: S1 Data — (ZIP) [file pgen.1011059.s014.zip › SIdata/Figure 5 + S5 + S6/5 + S6 - RpoSLac/5+S6C_WB/20201221_RpoSLac recovery/lmbchemidoc 2020-12-21 18h55m38s(StarBright B700).tif]

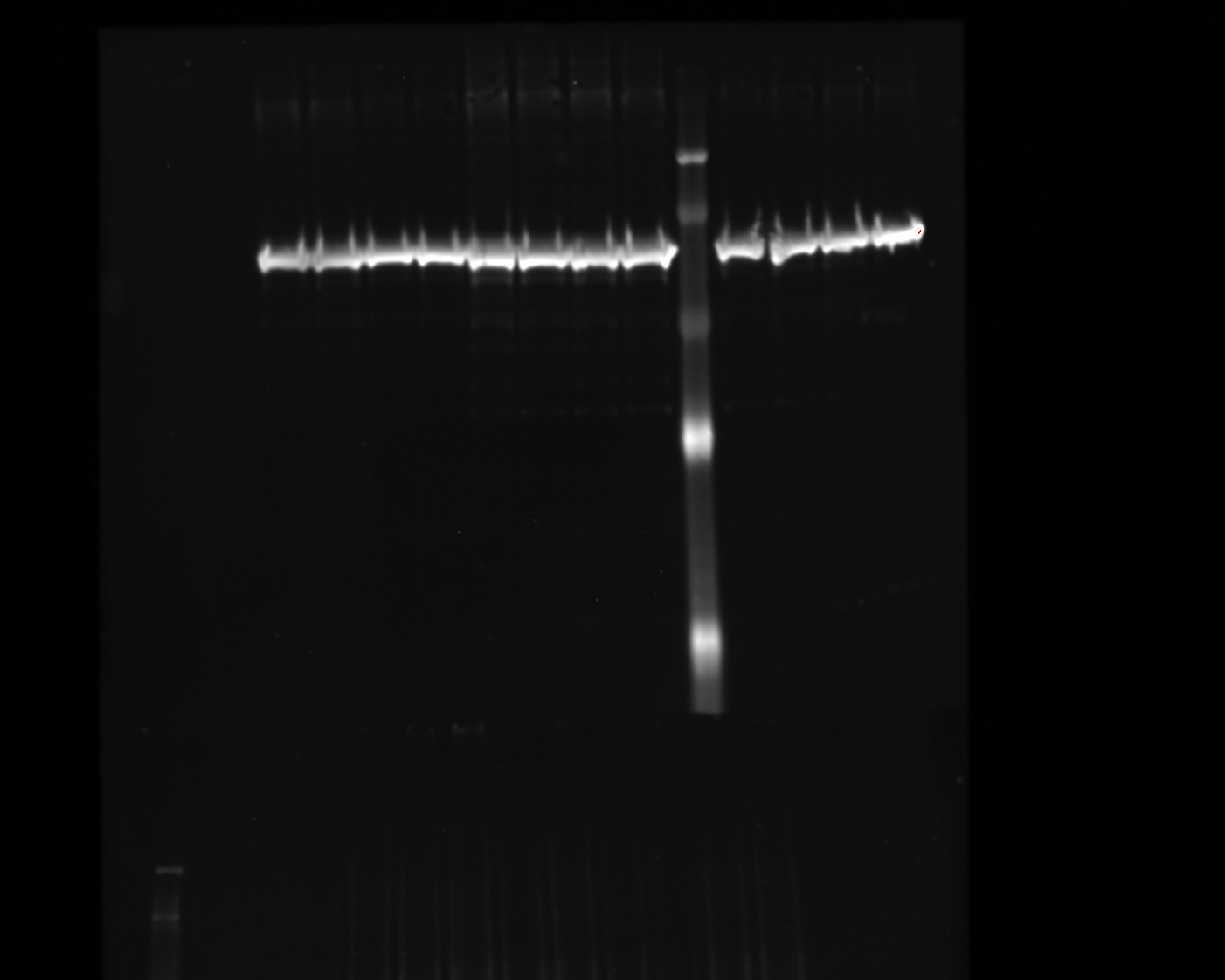

Supplement: S1 Data — (ZIP) [file pgen.1011059.s014.zip › SIdata/Figure 5 + S5 + S6/5 + S6 - RpoSLac/5+S6C_WB/20201221_RpoSLac recovery/lmbchemidoc 2020-12-21 18h55m38s(DyLight 800).tif]

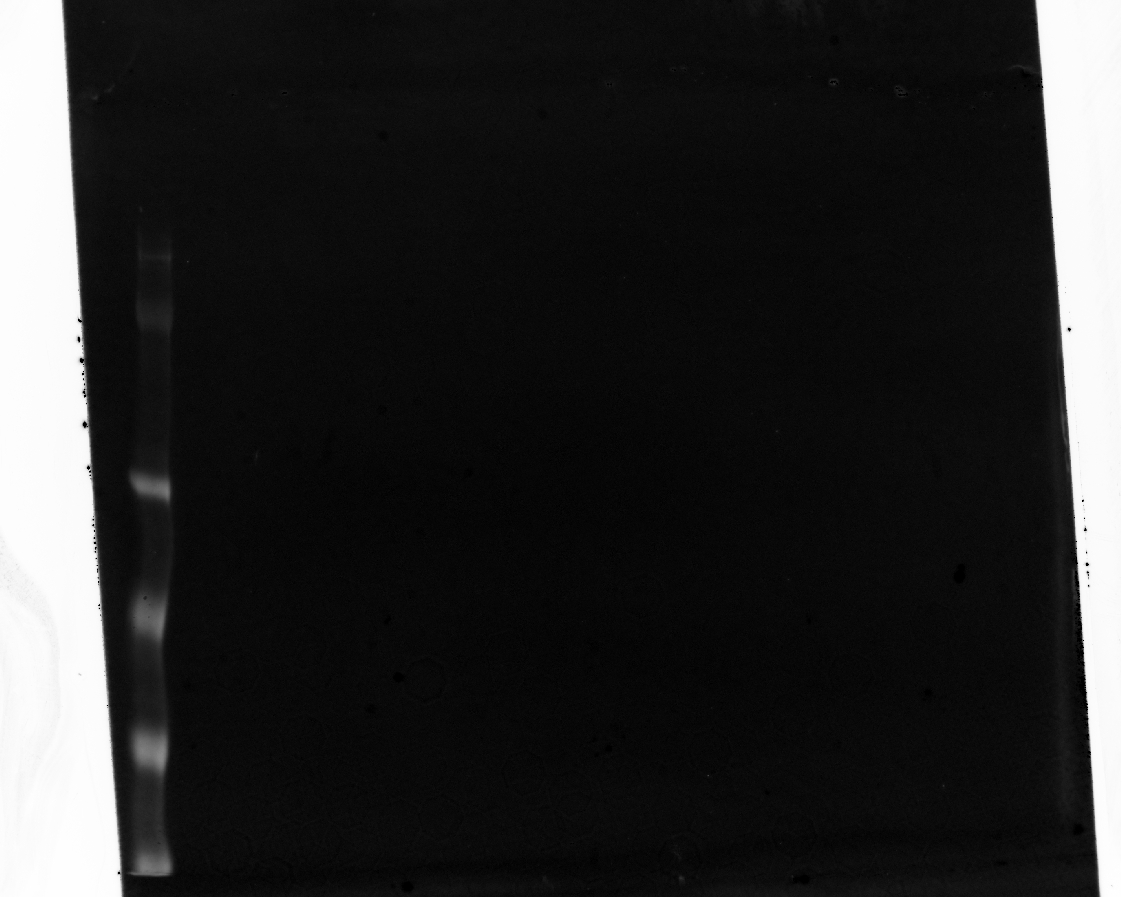

Supplement: S1 Data — (ZIP) [file pgen.1011059.s014.zip › SIdata/Figure 5 + S5 + S6/5 + S6 - RpoSLac/5+S6C_WB/20210217_RpoSLac/lmbchemidoc 2021-02-17 18h40m53s(Colorimetric).jpg]

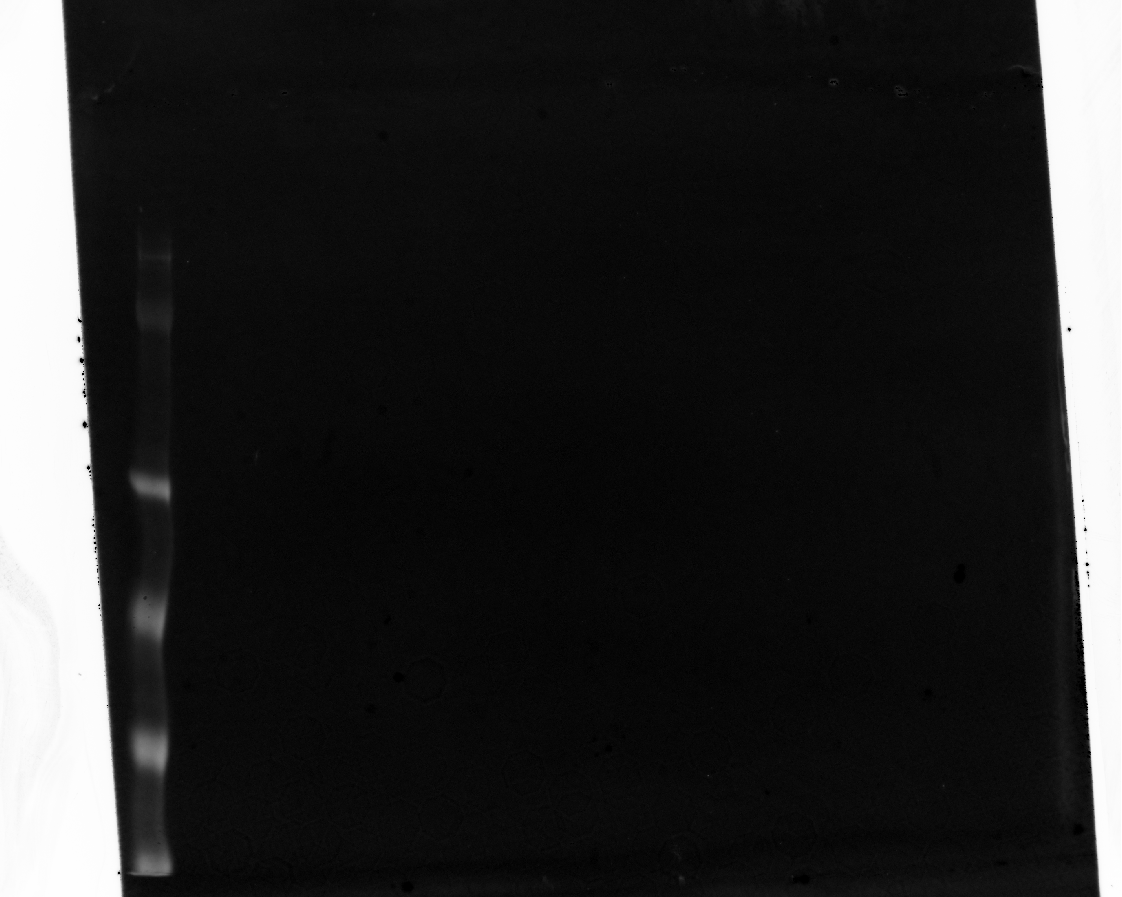

Supplement: S1 Data — (ZIP) [file pgen.1011059.s014.zip › SIdata/Figure 5 + S5 + S6/5 + S6 - RpoSLac/5+S6C_WB/20210217_RpoSLac/lmbchemidoc 2021-02-17 18h40m53s(Colorimetric).tif]

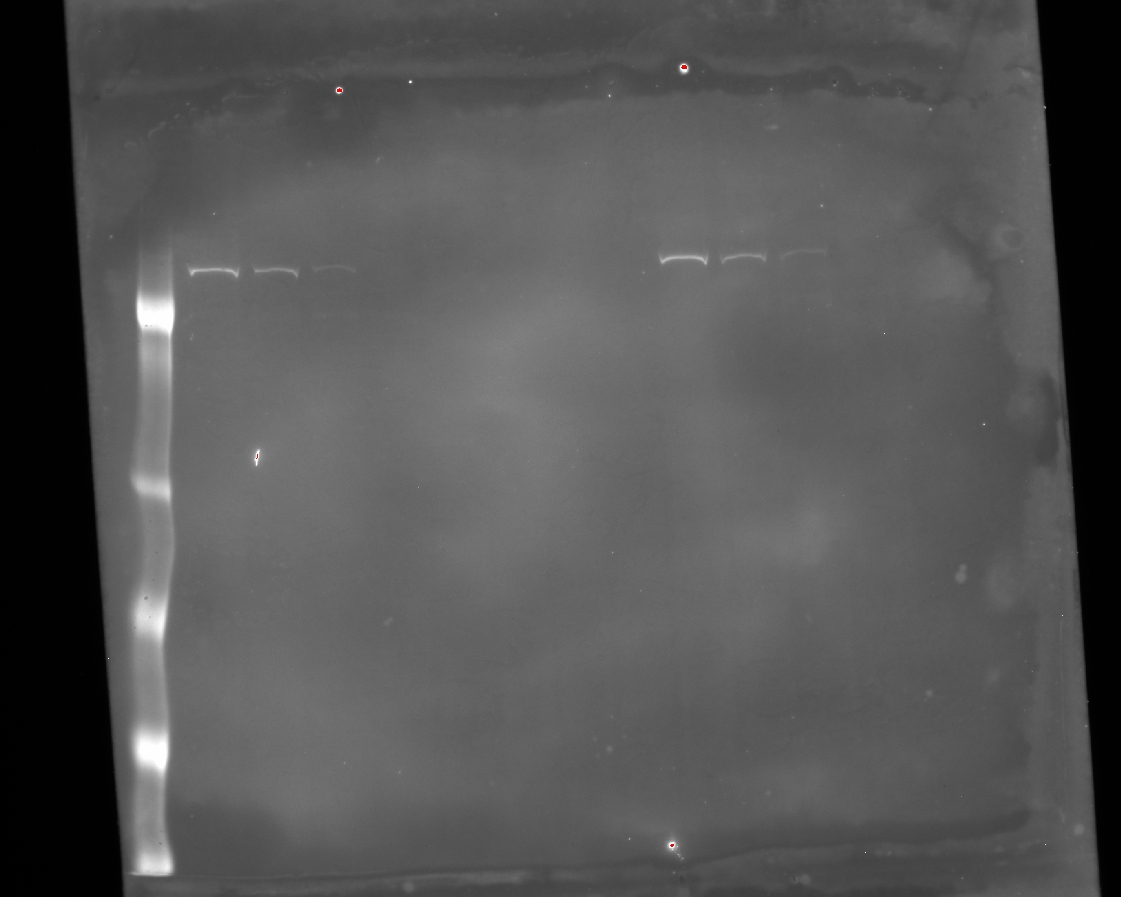

Supplement: S1 Data — (ZIP) [file pgen.1011059.s014.zip › SIdata/Figure 5 + S5 + S6/5 + S6 - RpoSLac/5+S6C_WB/20210217_RpoSLac/lmbchemidoc 2021-02-17 18h40m53s(StarBright B700).jpg]

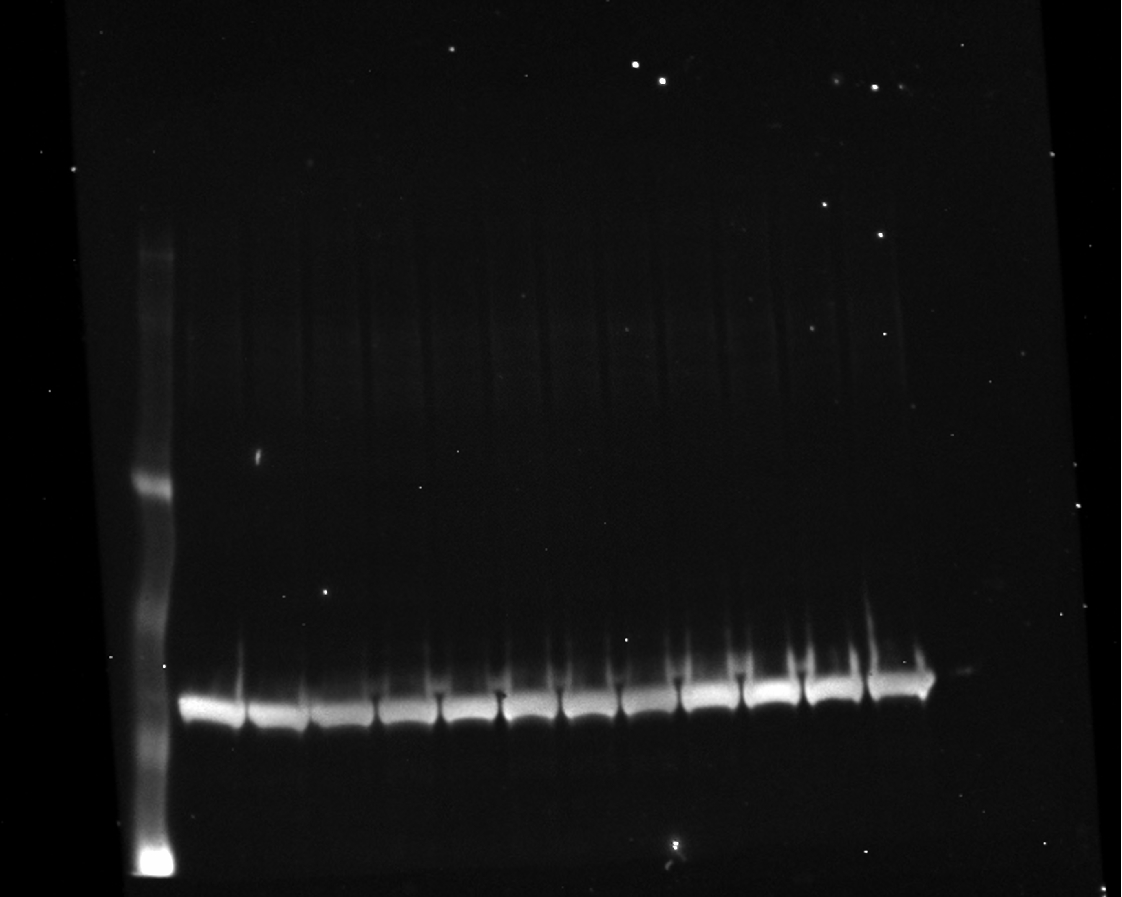

Supplement: S1 Data — (ZIP) [file pgen.1011059.s014.zip › SIdata/Figure 5 + S5 + S6/5 + S6 - RpoSLac/5+S6C_WB/20210217_RpoSLac/lmbchemidoc 2021-02-17 18h40m53s(DyLight 800).jpg]

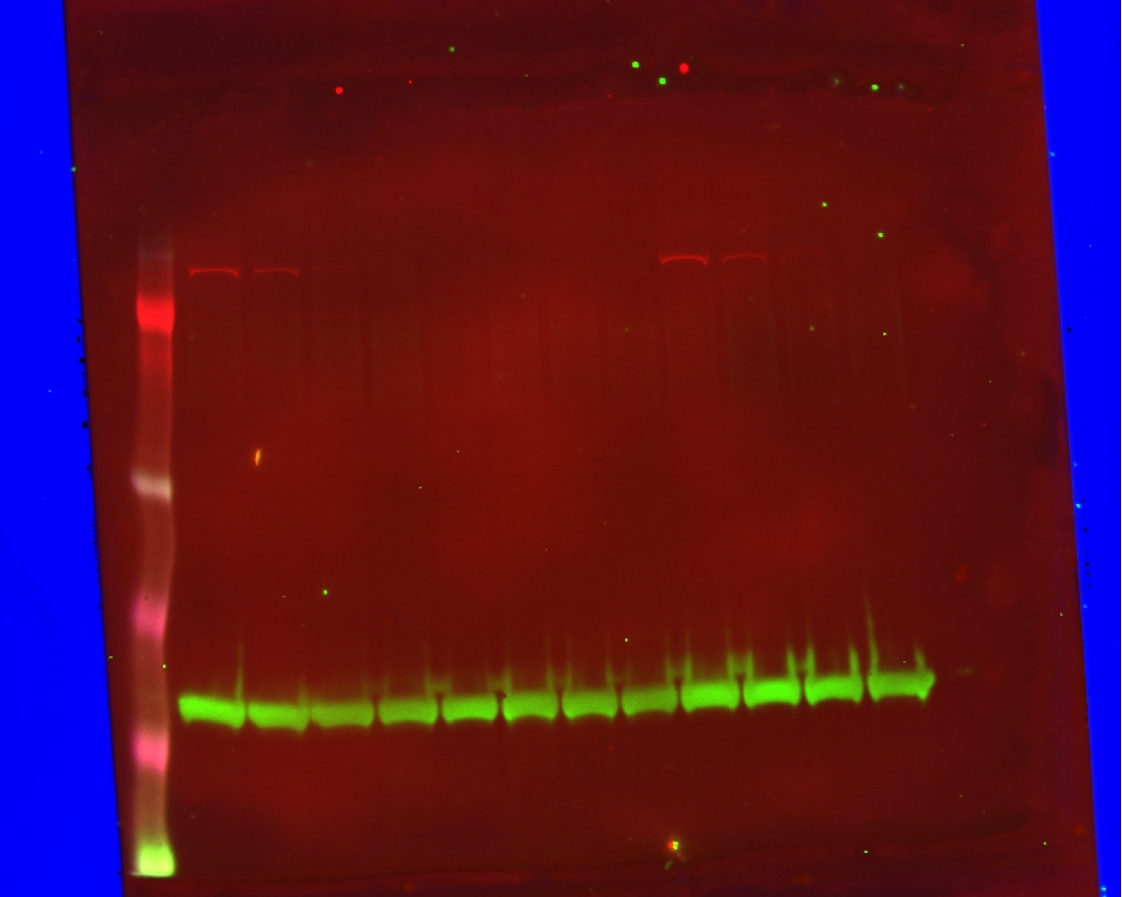

Supplement: S1 Data — (ZIP) [file pgen.1011059.s014.zip › SIdata/Figure 5 + S5 + S6/5 + S6 - RpoSLac/5+S6C_WB/20210217_RpoSLac/lmbchemidoc 2021-02-17 18h40m53s(Composite).jpg]

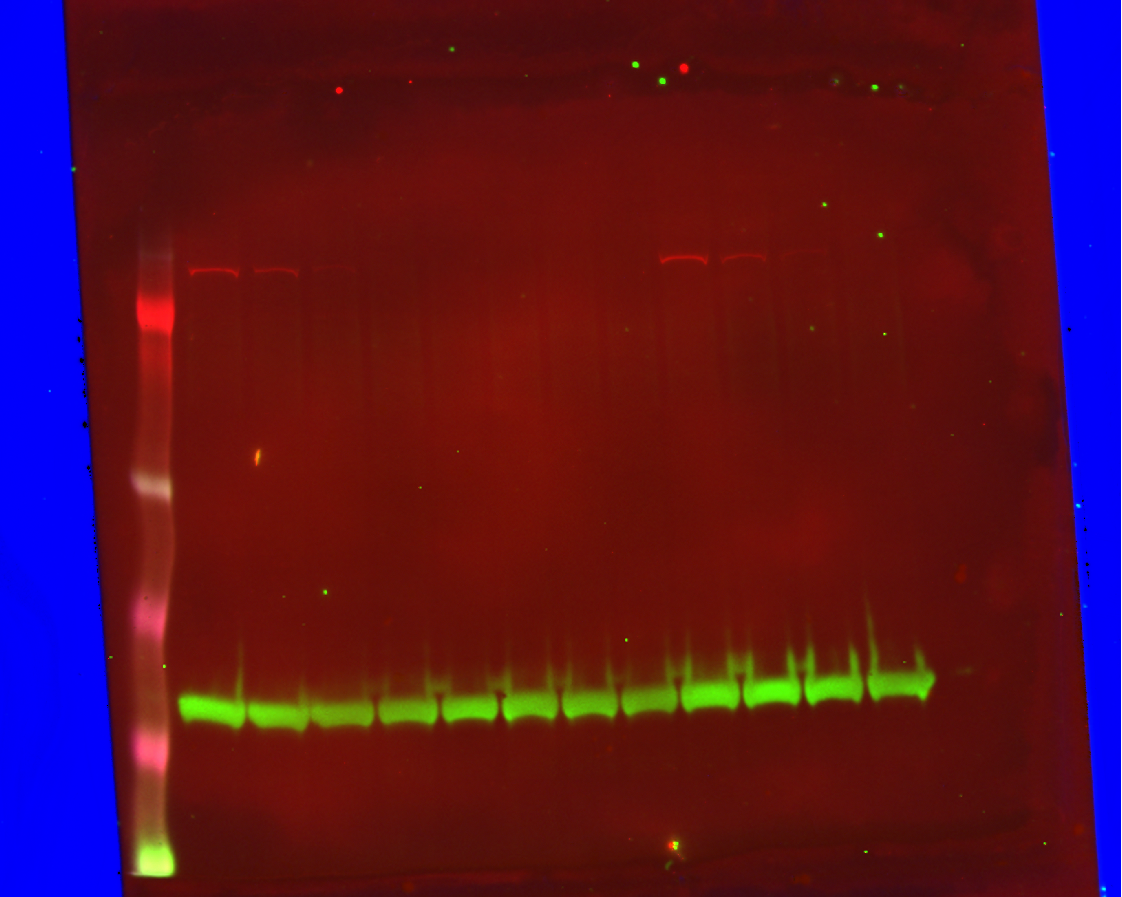

Supplement: S1 Data — (ZIP) [file pgen.1011059.s014.zip › SIdata/Figure 5 + S5 + S6/5 + S6 - RpoSLac/5+S6C_WB/20210217_RpoSLac/lmbchemidoc 2021-02-17 18h40m53s(Composite).tif]

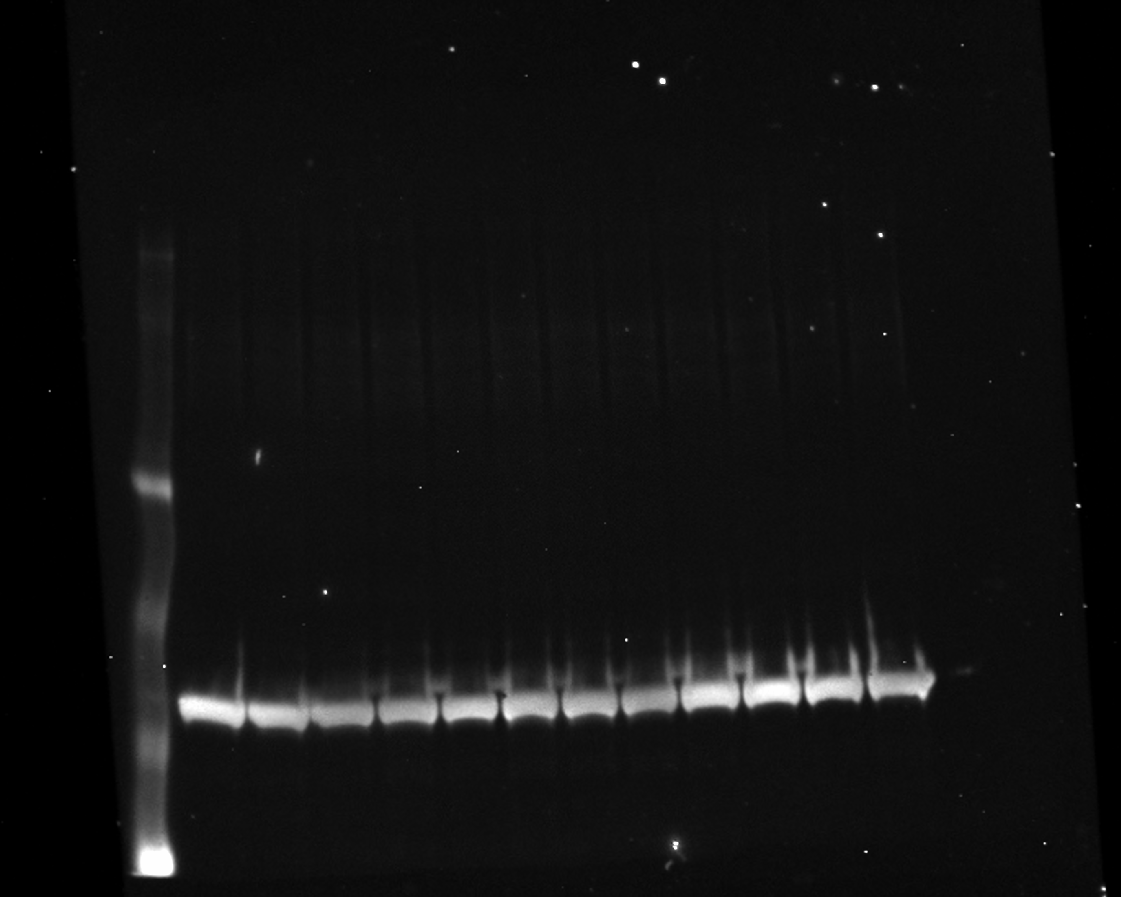

Supplement: S1 Data — (ZIP) [file pgen.1011059.s014.zip › SIdata/Figure 5 + S5 + S6/5 + S6 - RpoSLac/5+S6C_WB/20210217_RpoSLac/lmbchemidoc 2021-02-17 18h40m53s(DyLight 800).tif]

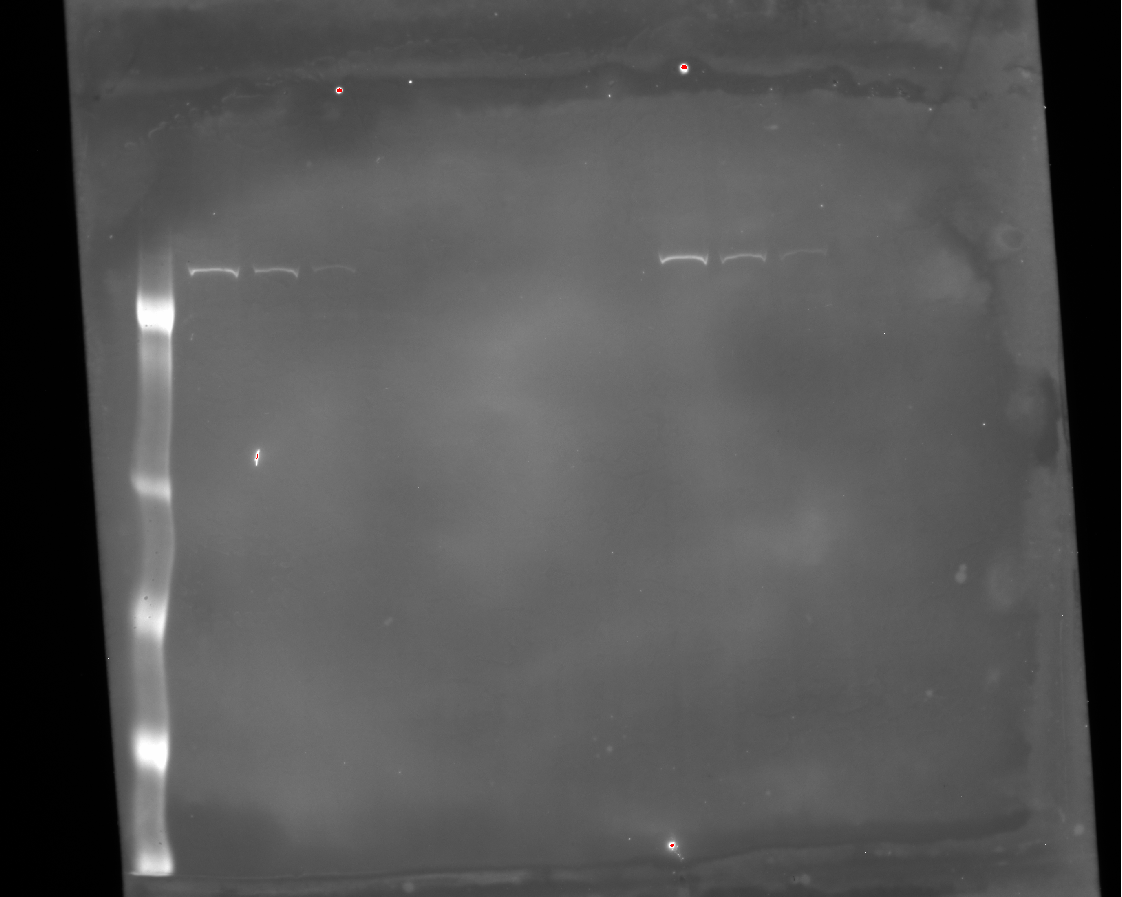

Supplement: S1 Data — (ZIP) [file pgen.1011059.s014.zip › SIdata/Figure 5 + S5 + S6/5 + S6 - RpoSLac/5+S6C_WB/20210217_RpoSLac/lmbchemidoc 2021-02-17 18h40m53s(StarBright B700).tif]
