# Supplementary material for: A negative feedback loop is critical for recovery of RpoS after stress in Escherichia coli
Source: PLoS Genet. 2024 Mar 11;20(3):e1011059. doi: 10.1371/journal.pgen.1011059 (PMC10957080; doi:10.1371/journal.pgen.1011059)
Supplement: Attachment — Submitted filename: Responses to reviewers Bouillet et al 1_8_24.docx [file pgen.1011059.s015.docx]

**To the Editor**: Many thanks to you and the reviewers for the rapid and very useful reviews of our manuscript. Here, we list our detailed responses to the reviewers. The following significant changes to the figures are noted: 1) New data, additional panels in Fig. 2, showing stationary phase stabilization and recovery in single, double, and triple ira mutants (supporting primary data in Fig. S1D) 2) Fig. 4E, split graph to show only recovery in Fig. 4E, during starvation in S3G (new panel). 3) Fig. S11 (Fitness correlations of iraP and crl) moved into main figure (now Fig. 9). 4) Addition of longer time points in Fig. S5D and addition of Table of RpoS half-lives in crl mutants added to Fig. S5. 5) deleted Fig. S10, not referenced in paper.

**Comments to the Authors:
Please note here if the review is uploaded as an attachment.**

**Reviewer #1:** The general stress sigma factor S (RpoS) of E.coli is an important master transcription factor at the center of a complex regulatory network orchestrating different stress responses, in addition to biofilm formation, motility and last but not least the transition into stationary phase. RpoS expression, activity and stability is controlled on many different levels, including regulatory proteolysis.
Here RpoS is targeted by an adaptor protein RssB for degradation by the ClpXP AAA+ protease complex. RssB is a response regulator whose expression and also activity can be controlled. RssB can interact with RpoS inducing a conformational change, which exposes a degron, targeting it for ClpXP degradation. Interestingly a number of so-called anti-adaptor proteins were identified, whose expression is induced by different stresses (e.g. Phosphate, Mg, DNA repair) and once synthesized they can interfere with the RssB activity, e.g. by sequestration, which results in a stress signal dependent stabilization and activation of RpoS.

In their manuscript "A negative feedback loop is critical for recovery of RpoS after stress in Escherichia coli" the authors observed that a deletion of the gene for anti-adaptor protein IraP (which is induced during phosphate starvation) plays a RpoS stabilizing role not only during the stress response but to a certain extent also during recovery from phosphate starvation stress response. In addition, the absence of IraP influences RpoS stability during escape from the other two tested stress conditions (glucose starvation or stationary phase) (Fig 2D).
Further experiments demonstrated that the recovery from phosphate starvation also depends on the negative feedback loop of RpoS dependent transcription of rssB and the presence of Crl which stabilizes the RnaPol/RpoS Holoenzyme, known to further facilitate this negative feedback loop. Both proteins, IraP and Crl, appear to support RpoS activity and stability in parallel processes.

General comments

-This is a very interesting and comprehensive manuscript entailing many well-designed experiments to further characterize, examine and investigate the molecular mechanisms controlling the escape from stress response pathways. This is interesting since thereby it can be investigated what happens when the conditions for a bacterial population are improving and the cells can for example restart growth and resume to replicate and divide again.

-It is interesting to note that the three tested more complex stress conditions all could include for example the synthesis of the (p)ppGpp alarmone as part of the stress response. Maybe the common regulatory effect detected for IraP during recovery from stress responses, might be caused by such a commonality in the stress responses, and in the case of (p)ppGpp, might also coordinate with the transcriptional and also translational restart upon lowered alarmone levels.

**Response**: We agree with the reviewer that ppGpp is the commonality between the 3 conditions tested, and this has been an attractive possibility that we continue to investigate. To do this properly, we have been working on trying to properly mimic the stress-dependent induction of IraP from an ectopic promoter to investigate recovery under conditions where ppGpp should not change. While these experiments are beyond the scope of this paper, initial results suggest that changes in ppGpp are not likely to explain recovery fully. No change has been made to the text at this point.

-It could be discussed how the interaction of IraP and possibly the two other anti-adaptor proteins with RssB help to integrate the different signals and how IraP and the other adaptor proteins interact with RssB to control this proteolytic switch, especially when it has to be reverted.

**Response**: We added a new experiment (Fig. 2E, F, with primary data in Fig. S1D) in which stabilization during stationary phase and recovery from stationary phase was evaluated in strains carrying various combinations of the three *ira* mutant alleles. The results suggest that only IraP plays a role in recovery from this condition. Each of the anti-adaptors interacts differently with RssB, and thus recovery may well be very different for each. An in-depth discussion of this is best saved for when we know more about recovery from IraM or IraD-promoted stabilization, but we have added text to results and discussion:

Results: “Given the somewhat unexpected role of IraP in recovery from glucose starvation and stationary phase, we further investigated whether the other two well-characterized anti-adaptors, IraD and IraM, also played roles in recovery, focusing on stationary phase. Single, double, and triple mutants of the three *ira* genes all still had stable RpoS during stationary phase (Fig. 2E; Fig. S1D). During recovery, IraP was the primary determinant of whether recovery was as it was in WT cells (half-life of around 4-5 minutes) or more rapid (2’, as seen in Fig. 2D). Thus, IraD and IraM did not play a significant role in either stabilization or recovery.

Discussion: “In stationary phase, at least two anti-adaptors, IraP and IraD, have been shown to be induced [30]. However, we find that even in a strain deleted for the genes for all three characterized anti-adaptors, *iraP*, *iraD* and *iraM*, RpoS is stabilized in stationary phase (Fig. 2E, Fig. S1D).

We found that IraP but not IraD or IraM, affected recovery from stationary phase (Fig. 2F). IraP, IraD and IraM are expressed under different conditions, are not structural homologs and each interact differently with RssB [9]. Whether IraD and IraM also allow rapid recovery after stresses under which they are expressed remains to be investigated.”

-Especially for the reasons given in line 241-247 I believe that the stability (degradation) of IraP should not be totally ruled out as a mechanism to explain its role in escape from the stress conditions.

**Response:** We agree with the reviewer that IraP degradation cannot be excluded and now acknowledge this explicitly: “Thus, unlike sRNAs, there is no evidence that IraP is used stoichiometrically or is degraded when RpoS does not need to be stabilized anymore, but further work will be necessary to fully rule out degradation or inactivation of IraP during recovery.”n addition, we continue to investigate whether we can tag the protein in other ways that do not interfere with recovery to allow us to directly follow it.

Minor comments

Somehow the references appear as if they could have been misplaced maybe upon reformatting. It would be good to systematically check them.

For example:
-Ref (28) (Pruteanu et al) in line 152 and line 460 should probably be (27) (Peterson et al)?
-Ref (29,33) in line 201 should probably be (28) (Pruteanu et al)?
Ref (25) in line 253 should probably be rather (24 (Typas et al) and maybe other references on the role of Crl in the feedback loop?
Ref (30,31) in line 460 Ref 31 (Becker et al 1999) describe the regulated proteolysis of RpoS via RssB and discuss the Bacillus competence regulatory proteolysis (including ComS which is also a kind of anti-adaptor protein) but at that time the Ira anti-adaptor proteins were not yet known.

Ref 45 in line 518 does not describe the website mentioned and described in this paragraph. The correct reference seems to be cited in the figure legend.

**Response**: Thank you for noticing these mistakes, which happened somewhere during reformatting and have now been corrected.

A typo in line 163

**Response**: Corrected

**Reviewer #2:** Numerous studies have investigated the induction of bacterial stress responses, yet the mechanisms governing bacterial recovery from stress are poorly understood. The authors asked how E. coli reverses the stabilization of the sigma factor RpoS in the recovery phase. They focused on two proteins, the anti-adaptor IraP and Crl, an activator of the RpoS-RNA polymerase holoenzyme. These proteins collectively contribute to a negative feedback loop that restores basal RpoS levels when the stress is gone. Despite these insights, the exact mechanism remains unsolved.
This is a solid study that will be of interest to microbiologists working on stress responses. The experimental design is clever, and the results support the conclusions. While the manuscript is carefully prepared and well written, there may be room for streamlining in various sections. For someone unfamiliar with the field, the information is very dense and rich in details. The Discussion spanning over more than eight pages appears somewhat lengthy. Trimming of detailed recapitulations of experimental results, for example on Crl mutants (page 24) or RssB phosphorylation (page 27), could enhance conciseness. Otherwise, I only have a few relatively minor comments.

**Response:** We shortened the discussion, moving the first paragraph into the introduction and the fitness browser discussion for IraP and Crl into the results, as suggested by reviewer #3, and shortening other sections. We did leave the short section on RssB phosphorylation, since our suggestion that RssB is likely fully phosphorylated during our growth conditions is somewhat unexpected and novel.

I understand why the study focusses on IraP. Given that there are some gaps in the overall understanding, I was wondering whether the authors tested if and how the other two RssB-binding anti-adaptors, IraD and IraM, contribute to the recovery process.

**Response:** In response to this comment, related to a comment from Reviewer 1, a new experiment was added (Fig. 2E, F, with primary data in Fig. S1D) in which stabilization during stationary phase and recovery from stationary phase was evaluated in strains carrying various combinations of the three ira mutant alleles. The results support IraP playing a major role during recovery from this stress. Some text describing this has been added to results:

“Given the somewhat unexpected role of IraP in recovery from glucose starvation and stationary phase, we further investigated whether the other two well-characterized anti-adaptors, IraD and IraM, also played roles in recovery, focusing on stationary phase. Single, double, and triple mutants of the three *ira* genes all still had stable RpoS during stationary phase (Fig. 2E; Fig. S1D). During recovery, IraP was the primary determinant of whether recovery was as it was in WT cells (half-life of around 4-5 minutes) or more rapid (2’, as seen in Fig. 2D). Thus, IraD and IraM did not play a significant role in either stabilization or recovery.

Discussion: “In stationary phase, at least two anti-adaptors, IraP and IraD, have been shown to be induced [30]. However, we find that even in a strain deleted for the genes for all three characterized anti-adaptors, *iraP*, *iraD* and *iraM*, RpoS is stabilized in stationary phase (Fig. 2E, Fig. S1D). We found that IraP but not IraD or IraM, affected recovery from stationary phase (Fig. 2F). IraP, IraD and IraM are expressed under different conditions, are not structural homologs and each interact differently with RssB [9]. Whether IraD and IraM also allow rapid recovery after stresses under which they are expressed remains to be investigated.”

Line 585: Please correct ItaP to IraP.

**Response**: Corrected

**Reviewer #3:** This paper is an important contribution to our understanding of bacterial stress responses. To date, the vast majority of work on bacterial stress responses focuses on how those responses are induced by a stress. This work focuses on the other half of that coin- how the response is turned off once a stressful condition ends. This is an important conceptual framing.

This paper focuses on the RpoS protein of E. coli, a well studied alternative sigma factor that accumulates in response to stress. Regulation of RpoS levels occurs at multiple levels, with regulated proteolysis playing an important role. Degradation of RpoS by the ClpXP protease requires the adaptor protein RssB. RssB's ability to interact with RpoS is regulated by small proteins called anti-adaptors, which were discovered in the senior author's lab. This paper demonstrates that active degradation of RpoS restarts upon relief from the stress, and that this restart involves a feedback loop with RssB as well as the regulator Crl. The authors are not able to determine the molecular mechanism kicking off this active degradation cycle, but they are able to eliminate some likely possibilities.

I have no concerns with the design or execution of the experiments. My only concerns with data interpretation have to do with a few places where it is difficult for me to assess the authors' interpretation of the data:

Line 245: I cannot see data in Figure 4E to support the claim that the SPA tag interfered with rapid recovery. In fact, I can't really see data for the iraP-SPA blots at all. I assume that they are hiding under the wild-type data, but I can't see which set of wild-type data.

**Response**: Fig. 4E was modified to make it more readable, removing the kinetics of the two strains during phosphate starvation and moving them to a new panel in supplemental Fig. S3 (S3G). This leaves only the phosphate recovery samples for the WT and *iraP*-SPA strains in this panel, hopefully making it clearer.

Line 290 - 293 / Figure S5: The authors interpret the data in Figure S5 to show that crl is important for recovery of degradation but that crl doesn't play a role during starvation itself. In the plots that are part of Figures S5C/D, it looks like crl mutants do have lower half-lives than wild-type. Looking at the blots, the effect of crl looks to be larger during recovery, but it is difficult for me to make that assessment based only on the blots. This argument would be stronger if there were graphs for parts A and B, and then the authors computed the half-lives for the various genotype/condition combinations.

**Response**: The graphs associated with S5A and B are shown in figure 5 A and B; we now clearly state that in the legend for Fig. S5. New sets of experiments were also performed, including a longer time point (40’) after antibiotic addition, now included in Fig S5D to allow a better comparison of the effect of Crl for RpoS stabilization during phosphate or glucose starvation. The results show that Crl doesn’t significantly impact RpoS stabilization in these conditions. A table summarizing the RpoS half-lives in the different strains and conditions was added (Fig S5E).

Beyond that, I have a few comments about the writing that I think will make the paper easier to read:

Line 114 and many other subsequent places: I found the "chase" designation very confusing because I believe the authors are using it in a nonstandard way. Protein turnover can be determined from a pulse-chase experiment, which the Oxford Dictionary of Biochemistry and Molecular Biology (2 ed.) describes as:

"a technique whereby cells growing in culture are exposed for a short period (pulse) to a radiolabelled molecule such as an amino acid and then transferred to a medium containing an excess of the nonlabelled substance for a longer period (chase). By removing samples at intervals the fate of intracellular components labelled during the pulse can be ascertained."

The experiments in this paper have no pulse of labeled molecules, so there is no chase with non-labeled molecules. Instead, all protein synthesis is stopped with the addition of an antibiotic, so the change in protein levels are due solely to degradation with no contribution of protein synthesis. I would suggest that the authors find some different nomenclature to distinguish between experiments that measure the net accumulation minus degradation vs. those that measure degradation only. Other studies from this lab that simply mark data as "minutes after Cm addition" were clear to me and I wonder if something describing which samples had chloramphenicol vs. which did not would be more straightforward.

**Response**: We now indicate, both in the text where first introduced and in the legend to Figure 1, the way in which we are using “chase” here. We have used this type of description for years to describe following stability of RNAs or proteins after antibiotic treatment. In the legend to Figure 1 we spell out one of the important differences. We have also tried to clarify throughout adding, “minutes after Cm addition” was added in the figure legends and the protocol used to follow RpoS degradation. We believe keeping the “chase” label is appropriate in current usage and important to distinguish experiments where antibiotic was added (reflecting rate of RpoS degradation) vs not (reflecting a combination of RpoS accumulation + degradation).

Addition to Fig. 1 legend: “Note that in this work, an antibiotic chase to block new protein synthesis is used to follow the half-life of proteins, rather than a radioactive or other label followed by a chase. One difference for an antibiotic chase, as compared to a pulse-chase, will be that the protein followed during the chase is not necessarily newly synthesized.”

Line 147-149: This statement is unclear to me. Are you saying that IraD plays a minor, not major role? Or are you saying that IraD plays a major role in the transition to stationary phase but a minor role during stationary phase? Or something else?

Response: Rephrased to clarify (new text: “IraD appears to contribute to increasing RpoS levels during the transition from late exponential to stationary phase but plays a minor role in stabilizing RpoS stability in this condition [30].”

Line 163: "bes" is a typo

**Response**: Corrected

Lines 424 - 447: This reads more like introduction than discussion. I think the discussion would be improved by immediately letting the reader know major experimental findings.

**Response**: This section was moved to the introduction as suggested.

Lines 514 - 526: I love the idea of using these data. A new analysis using existing data is still a new result, so this section should be in the results section, rather than in the discussion.

**Response**: The former figure S11 is now fig 10, and the text associated with it was moved to the results section; thank you for the suggestion. In fact, this very high correlation was a motivating observation early in this study.

**Have all data underlying the figures and results presented in the manuscript been provided?**
Large-scale datasets should be made available via a public repository as described in the *PLOS Genetics* [data availability policy](https://gcc02.safelinks.protection.outlook.com/?url=http%3A%2F%2Fjournals.plos.org%2Fplosgenetics%2Fs%2Fdata-availability&data=05%7C02%7Csophia.bouillet%40nih.gov%7Cec5b122dd8cd446afe7f08dbfae51f5f%7C14b77578977342d58507251ca2dc2b06%7C0%7C0%7C638379634365578608%7CUnknown%7CTWFpbGZsb3d8eyJWIjoiMC4wLjAwMDAiLCJQIjoiV2luMzIiLCJBTiI6Ik1haWwiLCJXVCI6Mn0%3D%7C1000%7C%7C%7C&sdata=GiaZ0ccFVu3F4si6TE%2Fnv8OmPGIjqSZd9Ws6JF5zpRI%3D&reserved=0), and numerical data that underlies graphs or summary statistics should be provided in spreadsheet form as supporting information.

Reviewer #1: Yes

Reviewer #2: Yes

Reviewer #3: Yes

PLOS authors have the option to publish the peer review history of their article ([what does this mean?](https://gcc02.safelinks.protection.outlook.com/?url=https%3A%2F%2Fjournals.plos.org%2Fplosgenetics%2Fs%2Feditorial-and-peer-review-process%23loc-peer-review-history&data=05%7C02%7Csophia.bouillet%40nih.gov%7Cec5b122dd8cd446afe7f08dbfae51f5f%7C14b77578977342d58507251ca2dc2b06%7C0%7C0%7C638379634365578608%7CUnknown%7CTWFpbGZsb3d8eyJWIjoiMC4wLjAwMDAiLCJQIjoiV2luMzIiLCJBTiI6Ik1haWwiLCJXVCI6Mn0%3D%7C1000%7C%7C%7C&sdata=mRF6ZVkrm7dYv5at0tiv6yHi2FkUHV%2BA0MfcAE%2F8VcQ%3D&reserved=0)). If published, this will include your full peer review and any attached files.

If you choose “no”, your identity will remain anonymous but your review may still be made public.

**Do you want your identity to be public for this peer review?** For information about this choice, including consent withdrawal, please see our [Privacy Policy](https://gcc02.safelinks.protection.outlook.com/?url=https%3A%2F%2Fwww.plos.org%2Fprivacy-policy&data=05%7C02%7Csophia.bouillet%40nih.gov%7Cec5b122dd8cd446afe7f08dbfae51f5f%7C14b77578977342d58507251ca2dc2b06%7C0%7C0%7C638379634365578608%7CUnknown%7CTWFpbGZsb3d8eyJWIjoiMC4wLjAwMDAiLCJQIjoiV2luMzIiLCJBTiI6Ik1haWwiLCJXVCI6Mn0%3D%7C1000%7C%7C%7C&sdata=cQmKlKM4WC3%2Bp7ahwPyNgMwtaMGo1eOnMlfOB7ovulk%3D&reserved=0).

Reviewer #1: No

Reviewer #2: No

Reviewer #3: No
